# Comparative Evaluation of Large Language Model Interfaces in Third Molar Surgery Complication Scenarios: Response Quality, Clinical Content, Potential Clinical Risk, Readability, and Externally Observable Response Latency—A Cross-Sectional Comparative Benchmark Study

**DOI:** 10.3390/jcm15197397

**Published:** 2026-09-23

**Authors:** İnci Rana Karaca, Esmanur Başer, Emre Ulubaş, Elif Betül Yıldırım

**Affiliations:** Department of Oral and Maxillofacial Surgery, Faculty of Dentistry, Gazi University, Ankara 06490, Türkiye

**Keywords:** large language models, artificial intelligence, third molar surgery, oral, cross-sectional studies, clinical decision-making, readability

## Abstract

**Background/Objectives:** Large language models (LLMs) are increasingly investigated in dentistry and oral and maxillofacial surgery, but comparative evidence regarding specialist-level response quality, clinical content, and potential safety concerns remains limited. This study compared four contemporary LLM interfaces in third molar surgery complication scenarios. **Methods:** Thirty open-ended specialist-level scenarios were submitted once to ChatGPT 5.4 Thinking, DeepSeek-V3.2, Gemini 3.1 Pro, and Microsoft Copilot (Work and Learning mode) in isolated single-turn sessions, yielding 120 responses. Two oral and maxillofacial surgeons, blinded to interface identity, independently assessed responses using a task-adapted five-point Global Quality Score (GQS). An additional post hoc criterion-referenced evaluation assessed diagnosis, management, escalation/referral, potentially harmful recommendations, safety-critical omissions, and potential clinical risk. Readability and externally observable response latency were also evaluated. **Results:** Among the sampled outputs, mean GQSs were 4.53 for Gemini, 4.40 for ChatGPT, 4.30 for DeepSeek, and 3.92 for Copilot (*p* < 0.001). Mean Clinical Content Scores were 4.80/5 for ChatGPT, 4.77 for Gemini, 4.75 for DeepSeek, and 3.90 for Copilot (*p* < 0.001). All responses were rated as recognizing the target complication. Within the 120 sampled outputs, one Copilot response met the study-specific criterion for a potentially harmful recommendation, and two Copilot responses met the predefined criteria for safety-critical omissions. Within this benchmark, the sampled Copilot outputs had higher study-specific potential clinical-risk scores than the sampled outputs from the other evaluated interfaces (*p* < 0.001). Readability and externally observable response latency also differed across the sampled interface outputs. **Conclusions:** In this single-generation benchmark, the sampled outputs showed different profiles in specialist-rated quality, clinical content, potential clinical risk, readability, and externally observable response latency under the specific interface configurations tested. These findings apply to the observed responses from the single synchronized testing session and should not be interpreted as establishing superiority of any underlying LLM, independent diagnostic accuracy, clinical equivalence, clinical safety, effectiveness, or readiness for autonomous decision support.

## 1. Introduction

Third molar removal is one of the most frequently performed procedures in oral and maxillofacial surgery and may be associated with a broad spectrum of intraoperative and postoperative complications [1,2,3]. The occurrence and severity of these complications are influenced by patient-related, radiographic, anatomical, and operative factors, including impaction characteristics, surgical difficulty, proximity to adjacent structures, and operator-related variables [4,5,6]. Common postoperative problems include pain, swelling, trismus, alveolar osteitis, infection, and hemorrhage, whereas less frequent but clinically important events include mandibular fracture, neurosensory disturbances, oroantral communication, and displacement-related complications [7,8]. These complications may affect postoperative quality of life and are also relevant to preoperative counseling and informed consent [9,10].

The clinical significance of third molar complications extends beyond their frequency because their urgency and management requirements vary considerably. Neurosensory disturbances involving the inferior alveolar or lingual nerve may persist after mandibular third molar surgery, and their occurrence is influenced by anatomical and radiographic risk factors [11,12,13]. Delayed postoperative infection and patient-related factors such as age further contribute to the heterogeneity of complication profiles [14,15]. The clinical burden is also reflected in prospective complication data. In a multicenter study of 1826 lower third molar extractions, postoperative complications occurred in 10.0% of cases; dry socket was reported in 3.2%, inferior alveolar nerve injury in 1.7%, wound infection in 1.4%, lingual nerve injury in 0.2%, and postoperative hemorrhage in 0.3% [6]. Accordingly, third molar complications provide an appropriate clinical stress test for AI systems because they range from relatively routine postoperative problems to less frequent but time-sensitive surgical and systemic emergencies, with substantial variation in urgency, management complexity, and escalation requirements. Successful responses therefore require more than factual knowledge retrieval; they must preserve clinically appropriate prioritization, identify when specialist referral or emergency escalation is required, and avoid potentially harmful recommendations.

Large language models (LLMs) are increasingly being investigated in dentistry for clinical knowledge assessment, question answering, patient information, and educational applications. However, fluent and well-structured responses should not be assumed to be accurate, complete, clinically appropriate, or safe. In impacted mandibular third molar assessment, artificial intelligence systems have demonstrated variable agreement with expert classification [16]. In a comparative evaluation of contemporary LLMs for traumatic dental injuries, ChatGPT-4o achieved a mean accuracy score of 4.74 ± 0.66 compared with 4.30 ± 1.01 for Gemini Advanced, while DeepSeek achieved a mean completeness score of 2.78 ± 0.46 compared with 2.56 ± 0.57 for Gemini Advanced [17]. In the specific context of third molar extraction, ChatGPT-generated patient information achieved a mean expert accuracy rating of 4.36/5, and 18 of 19 responses addressing AAOMS consensus content were closely aligned with those recommendations; however, readability remained relatively demanding (Flesch Reading Ease = 52; Flesch–Kincaid Grade Level = 10), and the responses did not provide citations or references [18]. More recent comparative work has additionally highlighted that aggregate performance measures may obscure clinically relevant error patterns, supporting evaluation approaches that consider not only overall performance but also the nature and potential consequences of individual errors [19].

Despite this growing literature, comparative evidence remains limited regarding specialist-level management of a heterogeneous spectrum of third molar surgery complications across contemporary publicly accessible LLM interfaces. In particular, it remains unclear whether differences among interfaces are evident not only in overall response quality but also in clinically consequential dimensions such as management completeness, appropriate escalation or referral, potentially harmful recommendations, and safety-critical omissions. The novelty of the present study lies in combining a matched, specialist-level benchmark of heterogeneous third molar complication scenarios with a multidimensional evaluation extending beyond global response quality. This approach is clinically relevant because a fluent or globally well-rated response may still omit an essential management step, fail to recommend appropriate escalation, or contain information with potentially important clinical consequences.

The aim of the present study was to compare four publicly accessible LLM interfaces across 30 matched specialist-level third molar complication scenarios with respect to specialist-rated response quality, readability, externally observable response latency, and the additional post hoc criterion-referenced clinical-content and potential clinical-risk outcomes.

The study was designed as an exploratory comparative benchmark rather than a confirmatory hypothesis-testing study. The prespecified comparative objectives were to examine whether specialist-rated response quality, readability, and externally observable response latency differed across the evaluated interfaces. Following completion of the original global quality assessment, additional criterion-referenced safety-oriented analyses were conducted post hoc to characterize clinical-content completeness and potential clinical risk; these analyses were therefore considered exploratory and hypothesis-generating.

## 2. Materials and Methods

### 2.1. Study Design and Scope

This cross-sectional comparative benchmark study evaluated four publicly accessible large language model (LLM) interfaces using a standardized set of complication-focused, open-ended clinical scenarios related to third molar surgery. Each benchmark item was submitted to all four interfaces, resulting in a matched repeated-measures design in which the benchmark item, rather than the individual response, represented the natural blocking unit for between-interface comparisons.

The original evaluation focused on specialist-rated response quality, readability, and externally observable response latency. Specialist-rated response quality, assessed using a task-adapted Global Quality Score (GQS), constituted the primary outcome, whereas readability and externally observable response latency were secondary outcomes. Following completion of the original global quality assessment, an additional post hoc criterion-referenced analysis was performed to provide a more clinically granular characterization of response content and potential safety concerns. This analysis separately evaluated diagnostic recognition, management completeness, appropriate escalation or referral, potentially harmful recommendations, safety-critical omissions, and potential clinical risk. These post hoc outcomes were exploratory and were not part of the original prespecified comparative objectives; neither the Clinical Content Score nor the study-specific potential clinical-risk framework was prospectively specified or independently validated before use.

The study was designed as a controlled, text-based benchmark of first turn performance across publicly accessible LLM interfaces and was not intended to establish clinical effectiveness, patient benefit, or the safety of any evaluated interface for autonomous clinical decision support. Reporting of the chatbot evaluation was informed by the Chatbot Assessment Reporting Tool (CHART), which provides recommendations for transparent reporting of studies evaluating generative AI-driven chatbot health advice [20].

### 2.2. Ethical Considerations

This study involved no patients, patient records, human tissue, biological specimens, or personally identifiable health information. The two expert evaluators assessed only anonymized AI-generated textual outputs and participated solely in their professional capacity as specialist assessors. No personal, behavioral, health-related, or identifiable information concerning the evaluators was collected or analyzed. According to the applicable institutional requirements, ethics committee approval and informed consent were not required for the present study.

### 2.3. Llm Interfaces, Access Conditions, and Version Reporting

The evaluated interfaces were ChatGPT 5.4 Thinking (OpenAI, San Francisco, CA, USA), DeepSeek-V3.2 in reasoning mode (DeepSeek AI, Beijing, China), Gemini 3.1 Pro in conversational mode (Google, Mountain View, CA, USA), and Microsoft Copilot in Work and Learning mode (Microsoft, Redmond, WA, USA). All four interfaces were accessed through their publicly available web interfaces using the same computer and internet connection. Testing was conducted during a synchronized morning session on 11 March 2026, beginning at approximately 07:00 local time in Türkiye (UTC+3). The interface/model and mode labels displayed by each interface on the testing date were recorded. Provider-side build identifiers, backend revisions, and other implementation details that were not exposed through the public interfaces could not be archived.

The four interfaces were selected to represent distinct publicly accessible, general-purpose LLM interfaces from different providers that offered user-facing configurations suitable for complex professional and knowledge-intensive queries at the time of testing. The purpose was to compare contemporary interfaces that could be accessed directly by end users without custom model deployment or application programming interface integration, rather than to provide an exhaustive comparison of all available LLMs or to isolate underlying model architectures.

For each benchmark item, the four interfaces were queried sequentially in a fixed order: ChatGPT, DeepSeek, Gemini, and Copilot. This sequence was applied separately to every benchmark question and was maintained unchanged throughout the testing session. The interface order was not randomized or counterbalanced. The fixed sequence was used to maintain procedural consistency across items; however, it does not eliminate the possibility of short-term temporal influences on externally observable response latency.

Mode selection was task-oriented rather than based on an assumption of technical equivalence across providers. For each platform, we selected the publicly accessible user-facing configuration that was considered most appropriate for complex, professional, knowledge-intensive queries at the time of testing. The aim was to evaluate each public interface under a configuration suitable for specialist-level clinical information generation rather than to equate backend computational settings, which were neither standardized nor transparently comparable across providers. The selected configuration was fixed before benchmark execution and maintained unchanged across all 30 items within each interface. No configuration was altered on the basis of observed response quality or study outcomes. Accordingly, these provider-specific modes should be regarded as interface–configuration combinations rather than equivalent computational modes.

Each benchmark question was submitted in a newly opened chat. Browser cache and previous conversation history were cleared between queries. No follow-up questions, clarification prompts, or regeneration requests were permitted. All prompts were entered in English. The ChatGPT memory feature was disabled. These procedures were selected to standardize first-turn performance and minimize contextual carry-over between benchmark items, although they do not reproduce iterative real-world conversations. Only the final fully visible response was retained for analysis; hidden or interface-specific reasoning traces were not considered part of the evaluated output. Retrieval, search augmentation, personalization, and provider-side contextualization could not be fully standardized across interfaces because these functions were not uniformly exposed or controllable through the public web interfaces. No external sources, geographic information, or supplemental contextual material were manually supplied by the investigators beyond the standardized benchmark prompts. Interface-level search or retrieval functions were not manually invoked by the investigators; where such functionality formed part of the provider-default interface behavior, the resulting visible output was retained as generated. Some archived Copilot responses displayed external source labels and geographically contextualized recommendations, including references to Ankara that were not present in the benchmark prompts. The specific provider-side mechanism responsible for this contextualization could not be determined from the public interface and was therefore not experimentally controlled.

The same standardized instruction preceded each benchmark question:

“Please answer the following question at a specialist level in oral and maxillofacial surgery, in a manner consistent with accepted scientific literature, established textbook knowledge, and sound clinical decision-making principles.” The standardized instruction did not specify a national jurisdiction, qualifying professional degree pathway, or jurisdiction-specific scope of oral and maxillofacial surgical practice.

### 2.4. Development of the Benchmark Question Set

The benchmark comprised 30 open-ended clinical scenarios covering postoperative, neurological, infectious, hemorrhagic, anatomical, technical, systemic, and emergency complications associated with mandibular and maxillary third molar surgery. The question pool was developed using established oral and maxillofacial surgery textbooks, including Peterson’s Principles of Oral and Maxillofacial Surgery [21], Oral and Maxillofacial Surgery [22], and Oral and Maxillofacial Surgery: Surgical Textbook and Atlas [23], together with contemporary peer-reviewed literature. Scenario selection was content-driven and intended to provide broad clinical coverage rather than to reproduce the epidemiologic frequency of complications encountered in practice. No prespecified quota or formal balancing scheme was used for common versus rare complications or for routine versus emergency presentations. Instead, the benchmark was assembled to include a heterogeneous range of clinically relevant problems spanning relatively common postoperative complications, less frequent anatomical or neurological events, and time-sensitive systemic or surgical emergencies, with emphasis on variation in management complexity and the need for escalation or referral. Accordingly, the benchmark should be regarded as a clinically heterogeneous test set rather than a prevalence-weighted or frequency-balanced sample of third molar complications. The benchmark size was content-driven rather than determined by an a priori statistical power calculation. Thirty scenarios were retained to provide broad coverage of clinically distinct complication types and management demands while preserving a standardized matched design in which every scenario could be evaluated across all four interfaces. The benchmark was not intended to constitute a psychometrically validated specialty examination.

The clinical content of the scenarios was grounded in the established oral and maxillofacial surgery textbooks and contemporary peer-reviewed literature described above. This source-based development was used to support the clinical relevance and content coverage of the benchmark. However, no formal content validity index, Delphi procedure, independent pretesting panel, or external psychometric validation of the 30-item scenario set was performed before interface testing. Accordingly, the benchmark should be regarded as a source-informed, specialist-developed clinical test set rather than as a formally externally content-validated instrument.

To characterize the clinical composition of the benchmark more explicitly, the 30 scenarios were additionally grouped post hoc into eight clinically oriented complication categories according to the principal management problem represented in each item. These comprised displacement/foreign-body/oroantral complications (6/30, 20.0%), mechanical/structural/technical injuries (5/30, 16.7%), infectious/deep-space complications (4/30, 13.3%), hemorrhagic/anticoagulation complications (3/30, 10.0%), neurologic complications (3/30, 10.0%), systemic/drug-related emergencies (3/30, 10.0%), osteonecrosis/impaired bone healing (3/30, 10.0%), and other local postoperative sequelae (3/30, 10.0%). Because some scenarios contained features relevant to more than one clinical domain, each item was assigned to a single category according to its predominant clinical management problem. This categorization was introduced for descriptive transparency and exploratory subgroup summarization and was not part of the original inferential analysis.

During the original GQS-based evaluation, these sources served as clinical knowledge anchors for topic selection and specialist judgment but did not constitute a formal item-by-item objective gold standard. For the subsequent post hoc criterion-referenced and safety-oriented evaluation, an item-specific clinical scoring framework was constructed from the same established knowledge base. For each of the 30 benchmark scenarios, the framework specified the expected diagnostic recognition, three essential management elements, an appropriate escalation or referral pathway, a potentially harmful or contraindicated recommendation, and a scenario-specific safety-critical omission.

The item-specific criterion-referenced framework was developed jointly by the author team after completion of the original GQS assessment. The two independent oral and maxillofacial surgery evaluators who subsequently performed the criterion-referenced rescoring were not involved in defining or developing the item-specific criteria. Thus, the framework was established after generation of the 120 responses; however, the content of those responses was not consulted during framework construction. Framework construction was based solely on the benchmark scenarios and the established oral and maxillofacial surgery textbooks and peer-reviewed literature described above. During framework development, interface identities remained concealed, and the framework developers did not review interface-level GQS summaries, between-interface comparisons, or statistical results from the original GQS analysis. The complete framework, including the expected diagnosis/recognition criterion, three essential management elements, escalation/referral criterion, potentially harmful or contraindicated recommendation, and safety-critical omission for each scenario, was finalized and locked before criterion-referenced rescoring of the 120 anonymized responses began. Any disagreements regarding whether a proposed management element should be considered essential were resolved by author consensus with reference to the predefined clinical sources. No item-specific criterion was modified after rescoring commenced.

The complete benchmark specification is provided in the Supplementary Materials. Supplementary Table S1 presents the exact verbatim wording of all 30 benchmark questions together with the standardized instruction that preceded each query. Supplementary Table S2 presents, for each of the 30 items, the represented complication/scenario, expected diagnosis or recognition criterion, three essential management elements, appropriate escalation/referral criterion, potentially harmful or contraindicated recommendation, and predefined safety-critical omission. These Supplementary Materials therefore permit item-level examination of both the clinical scenarios and the criteria used in the post hoc assessment.

The benchmark was primarily designed to evaluate complication management rather than independent diagnostic reasoning. The diagnostic inferential demand varied across scenarios: some items required recognition from clinical signs and symptoms, whereas others explicitly named the complication or provided strong diagnostic cues. Accordingly, the diagnosis/recognition domain was intended to assess whether the response appropriately recognized and addressed the target complication presented in the scenario, rather than to serve as a measure of independent diagnostic accuracy or diagnostic reasoning performance.

To further characterize the diagnostic information embedded within the benchmark, each scenario was descriptively categorized according to its degree of diagnostic cueing as explicit/near-explicit, strongly cued, or clinically inferential. Explicit/near-explicit items directly stated the target complication or anatomical event; strongly cued items provided a characteristic clinical constellation or highly specific precipitating factor that substantially narrowed the differential diagnosis; and clinically inferential items required comparatively greater integration of timing, symptoms, risk factors, or differential diagnoses. This descriptive classification was introduced solely to improve transparency regarding the diagnostic demands of the benchmark and was not used for scoring or inferential statistical analysis. Item-level classifications are provided in Supplementary Table S1.

### 2.5. Query Execution and Response Collection

Each of the 30 benchmark scenarios was submitted once to each of the four LLM interfaces, yielding a total of 120 responses. Each query was processed in an isolated chat instance, and only the first fully generated visible answer was retained. No response was regenerated, refined, or modified on the basis of its content. The outputs were archived without substantive editorial correction. Visible source labels, citations, retrieval-related annotations, and geographically contextualized wording were preserved as part of the original interface output and were not removed from the archived response set.

The complete interface-generated response set is provided in Supplementary File S1 and is reproduced as generated, including any inaccuracies, unsupported statements, automatically generated citations, or other characteristics of the original outputs. Inclusion of these archived responses should not be interpreted as independent validation or endorsement of their content. The overall study workflow, including prompt administration, response archiving, blinded assessment, criterion-referenced scoring, and statistical analysis, is summarized in Figure 1.

### 2.6. Anonymization and Blinded Expert Review

Before expert assessment, interface names, logos, interface-specific identifiers, and other direct branding cues were removed from the archived responses, and each response was assigned a neutral study code. The evaluators were therefore blinded to interface identity. However, because differences in writing style, formatting, response structure, and response length could provide indirect cues regarding interface origin, complete source masking could not be guaranteed.

Two independent board-certified oral and maxillofacial surgeons with 19 and 20 years of post-residency clinical experience evaluated the responses independently and without access to the other evaluator’s scores. During the original global quality assessment, responses were judged in relation to established oral and maxillofacial surgery textbook knowledge and contemporary peer-reviewed literature. These sources functioned as specialist-rating anchors rather than as a prespecified objective gold standard.

Following completion of the original GQS evaluation, the same anonymized response set was subjected to the additional post hoc criterion-referenced and safety-oriented assessment described below.

### 2.7. Specialist-Rated Response Quality

Overall response quality was assessed using a task-adapted five-point Global Quality Score (GQS) (Table 1). The GQS was originally introduced as a five-point global measure for evaluating the overall quality and usefulness of health information and was adapted in the present study for specialist-level LLM-generated clinical responses [24]. A score of 1 represented very poor information containing substantial inaccuracies or omissions and providing minimal practical value, whereas a score of 5 represented highly accurate, comprehensive, clearly structured, and practically useful information.

Each evaluator independently assigned a GQS to every response. The primary response-quality outcome was the mean of the two evaluator scores for each response. The GQS is an ordinal five-point rating scale. The mean of the two independent evaluator scores was used pragmatically as a response-level composite to summarize the two specialist judgments and to retain a single matched response-level outcome for each interface–item combination. This averaging was not intended to imply that the underlying GQS categories constituted an interval scale. Accordingly, between-interface inference was based on rank-based non-parametric procedures rather than parametric comparisons of means. Inter-evaluator agreement for the original GQS assessment was evaluated separately for each LLM interface using a two-way random-effects, absolute-agreement intraclass correlation coefficient for average measurements. Percentile-bootstrap 95% confidence intervals were calculated using 10,000 resamples.

The task-adapted GQS was used as a global specialist-rating instrument and was not independently validated as an objective measure of guideline concordance, clinical correctness, or safety for this benchmark. Accordingly, GQS results were interpreted as specialist-rated overall response quality rather than as direct evidence of clinical safety or effectiveness.

### 2.8. Post Hoc Criterion-Referenced Clinical Content and Safety Evaluation

Following completion of the original GQS assessment and finalization of the item-specific framework described in Section 2.4, an additional post hoc criterion-referenced evaluation was performed to provide a more clinically specific assessment of response content and potential safety concerns. The framework had been finalized and locked before rescoring began and was applied uniformly to all 120 anonymized responses without interface-specific modification. Each response was then independently evaluated by the two oral and maxillofacial surgery specialists using the five domains described below.

Diagnosis/recognition (A) was scored as 0 when the response failed to recognize or incorrectly characterized the target complication presented in the benchmark scenario and 1 when the target complication was appropriately recognized. This domain was not intended as a measure of independent diagnostic accuracy because the amount of diagnostic information provided varied across benchmark items. Management completeness (B) was scored from 0 to 3 according to the number and completeness of essential management elements addressed in the response. Escalation/referral (C) was scored as 0 when appropriate escalation or referral was absent or incorrect and 1 when an appropriate escalation or referral pathway was identified. Potentially harmful recommendation (D) was coded as 0 when absent and 1 when a recommendation capable of introducing clinically relevant harm was present. Safety-critical omission (E) was coded as 0 when absent and 1 when clinically important information whose omission could materially affect safe management was missing. Because each benchmark item contained three prespecified essential management elements, the management-completeness score was intended as a criterion-counting measure rather than a fine-grained global assessment of management quality. A score of 3 indicated that all three predefined essential management elements were adequately addressed; lower scores indicated that one or more of these elements were absent or insufficiently addressed. Accordingly, responses receiving the same management score could still differ in clinical nuance, depth, organization, or explanatory quality, which were not intended to be captured by this domain-specific subscore.

A criterion-referenced Clinical Content Score was calculated separately for each evaluator as the sum of domains A, B, and C, yielding a possible score from 0 to 5. Management completeness therefore contributed three of the five available points, reflecting the management-focused nature of the benchmark scenarios. This weighting reflected the structure of the item-specific framework, in which management was represented by three distinct essential clinical elements, whereas diagnosis/recognition and escalation/referral were each represented by a single binary criterion. Nevertheless, because this structure gives management greater influence on the aggregate Clinical Content Score, a post hoc sensitivity analysis was performed using equal maximum weighting of the three content domains. For this analysis, the management score was rescaled from 0–3 to 0–1, and an Equal-Weight Clinical Content Score was calculated as A + (B/3) + C (range, 0–3). The same matched Friedman and Holm-adjusted Wilcoxon framework used for the primary Clinical Content Score was applied to this sensitivity analysis. The mean of the two evaluator-specific Clinical Content Scores was subsequently used as the response-level score for between-interface comparisons and was additionally expressed as a percentage of the five-point maximum.

Potentially harmful recommendations and safety-critical omissions were intentionally analyzed as separate binary safety outcomes and were not incorporated into the Clinical Content Score. This approach was selected to prevent clinically consequential safety findings from being diluted or obscured within an aggregate content score.

For each benchmark scenario, Supplementary Table S2 specifies the expected diagnostic recognition, the three essential management elements, the appropriate escalation or referral pathway, the potentially harmful or contraindicated recommendation, and the predefined safety-critical omission used for the post hoc assessment. The framework was developed specifically for the present study and has not undergone independent external validation.

To improve the clinical interpretability of the aggregate scores, representative response excerpts were additionally selected post hoc for illustrative presentation. For each interface, examples were chosen to demonstrate both a higher-performing response that adequately addressed the relevant criterion-referenced domains and a lower-scoring or criterion-deficient response illustrating an observed omission or limitation. The examples were selected after scoring had been completed and did not influence any rating or statistical analysis. Excerpts were abridged only for length, without substantive alteration of the quoted wording; the complete unabridged responses remain available in Supplementary File S1. These examples were intended solely to illustrate the clinical meaning of the scoring framework and should not be interpreted as statistically representative of the full response distribution.

### 2.9. Study-Specific Potential Clinical-Risk Classification

In addition to the domain-specific criterion-referenced assessment, each response was evaluated using a four-level study-specific potential clinical-risk framework developed de novo for the present benchmark. The framework was intended to characterize the potential clinical consequences of inaccuracies or omissions if a generated response were followed without specialist correction. CHART informed the decision to assess potentially harmful or misleading chatbot-generated health information systematically and transparently [20], whereas World Health Organization patient-safety terminology provided only broad conceptual context for considering the possible consequences of errors and omissions [25]. Neither CHART nor the WHO framework provides or validates the four categories, score thresholds, or category definitions used in the present study, and no direct correspondence with an established WHO or other clinical safety scale is intended.

A score of 0 indicated no clinically relevant potential risk, defined as a clinically appropriate response without a meaningful error or omission expected to alter clinical management or patient safety. A score of 1 indicated low clinical potential risk, representing a minor omission, imprecision, or incomplete explanation that was unlikely to alter clinical management or cause patient harm. A score of 2 indicated moderate clinical potential risk, representing a clinically relevant inaccuracy or omission that could plausibly result in incomplete management, delayed referral, inappropriate monitoring, or suboptimal care but was unlikely by itself to cause immediate severe harm. A score of 3 indicated high clinical potential risk, representing a major omission or inaccurate recommendation that could plausibly result in serious patient harm if followed without specialist correction. Because this four-level classification was developed de novo and post hoc for the present benchmark, it was not considered a formally validated clinical-risk instrument. To strengthen its clinical grounding and reproducibility, the category definitions were based on the potential management consequences of inaccuracies or omissions, were accompanied by third-molar/OMFS-specific interpretive examples, and were applied independently to the anonymized responses by two experienced board-certified oral and maxillofacial surgeons. Inter-evaluator reproducibility was assessed using exact agreement and quadratic-weighted Cohen’s κ for the ordinal risk classifications. These agreement analyses were intended to quantify scoring consistency under the present framework and were not interpreted as evidence of construct, criterion, or external validity. Formal validation would require prospectively specified criteria, independent external assessment, and evaluation in additional and more heterogeneous benchmark samples.

Both evaluators independently assigned a clinical-risk score to each anonymized response. For inferential between-interface comparisons, the mean of the two evaluator-specific risk scores was used as the response-level clinical-risk score, while evaluator-level category distributions were retained for descriptive analysis. The complete definitions and third-molar/oral and maxillofacial surgery-specific examples are presented in Table 2.

Because none of the interface-generated recommendations was implemented in actual patient care, the risk categories represent potential clinical risk rather than observed patient harm.

### 2.10. Readability Assessment

Readability was assessed from the archived final-visible response texts using a uniform preprocessing protocol applied identically to all responses. The benchmark question, timing labels, interface metadata, URLs, standalone source labels, and non-linguistic list or Markdown symbols were removed before analysis, whereas clinical terminology, sentence content, and substantive answer text were preserved. Hidden or interface-specific reasoning content was not included.

To reduce sensitivity to list formatting, each non-empty paragraph or list item was treated as containing at least one sentence, while conventional punctuation was used to identify additional sentence boundaries. Readability was calculated using Python version 3.13.5 and the English (en_US) hyphenation dictionary implemented in Pyphen version 0.17.2 for syllable estimation. Five established readability measures were calculated: Flesch–Kincaid Grade Level, Gunning Fog Index, Coleman–Liau Index, Simple Measure of Gobbledygook, and Flesch Reading Ease Score. The intended readership for interpretation of the readability results was a professional oral and maxillofacial surgery audience, consistent with the standardized prompt requesting specialist-level responses. Readability was therefore treated as a secondary descriptive measure of surface linguistic complexity and potential reading burden rather than as a measure of patient-facing comprehensibility, clinical accuracy, communication quality, or clinical usefulness. The five readability indices were retained as overlapping, complementary measures rather than as independent readability outcomes. Their inclusion was intended primarily to assess whether the observed between-interface pattern was robust to the choice of readability formula. Flesch–Kincaid Grade Level and Flesch Reading Ease both rely on sentence length and syllabic word complexity and are therefore expected to show similar patterns; FRES was retained principally as an inversely scaled alternative representation rather than as an independent construct. The Gunning Fog Index additionally emphasizes complex-word frequency, SMOG places particular emphasis on polysyllabic-word burden, and the Coleman–Liau Index uses character and sentence counts rather than syllable estimation. Thus, although the measures share substantial conceptual and mathematical overlap, they provide partly different operationalizations of surface linguistic complexity. Concordance across the five indices was interpreted as robustness to formula choice rather than as five independent confirmations of a readability effect. None of these indices was interpreted as an OMFS-specific validated measure of professional clinical communication.

Interface-generated conversational additions that formed part of the final visible response text, including closing invitations such as ‘Would you like me to…’, were retained in the readability analysis because they were part of the user-visible linguistic output and were not treated as metadata or formatting artifacts.

### 2.11. Externally Observable Response Latency

Externally observable response latency was treated as a secondary interface-level outcome and was measured manually using a digital stopwatch from prompt submission until visible text generation ceased. All measurements were obtained using the same hardware and internet connection. Measurable elapsed durations were recorded in whole seconds. Because the interface-testing sequence was fixed rather than randomized or counterbalanced, externally observable response-latency comparisons may also have been influenced by order-related or short-term temporal variation during the testing session. Responses were classified as ‘immediate’ when visible response generation was completed too rapidly after prompt submission for a distinct elapsed duration to be reliably captured using the manual stopwatch procedure. These observations therefore represent values below the practical measurement resolution rather than literal zero-second durations. For the primary analysis, they were assigned a lower-bound numerical value of 0 s. Because this coding could influence the distribution and between-interface comparisons, an additional post hoc sensitivity analysis excluded every benchmark item containing an ‘immediate’ observation from all four interfaces, thereby preserving the matched-item structure. No alternative positive duration was imputed because the exact elapsed times of these observations were unknown. In the present study, externally observable response latency refers specifically to the total user-observable interval from prompt submission until visible response generation ceased and should not be interpreted as time to first token, internal computational latency, model-processing time, or computational efficiency.

### 2.12. Statistical Analysis

Statistical analyses were performed using IBM SPSS Statistics version 27.0 (IBM Corp., Armonk, NY, USA). Descriptive statistics included means, standard deviations, medians, interquartile ranges, minima, maxima, counts, and percentages as appropriate.

Because the same 30 benchmark items were evaluated across all four LLM interfaces, between-interface comparisons were treated as repeated, matched observations. For the original GQS outcome, the mean of the two evaluator scores was used as the response-level specialist-rated quality score. Overall between-interface differences were evaluated using the Friedman test, with Kendall’s W reported as the omnibus effect size. Because the GQS is ordinal and averaging the two evaluator ratings represents a pragmatic response-level summary, a post hoc sensitivity analysis was additionally performed using the original evaluator-specific GQSs without averaging. The complete between-interface analysis was repeated separately for each evaluator using the Friedman test, followed, when significant, by Holm-adjusted pairwise Wilcoxon signed-rank tests. This sensitivity analysis preserved the original 1–5 ordinal ratings and assessed whether the primary between-interface pattern depended on aggregation of the two evaluator scores. When a Friedman test was statistically significant, the six pairwise interface contrasts were evaluated using two-sided Wilcoxon signed-rank tests with Holm adjustment for multiple comparisons. Rank-biserial correlation (r_rb) was reported as the paired effect size. To express paired differences on the original measurement scale, mean paired differences, calculated as the first interface minus the second, were accompanied by percentile-bootstrap 95% confidence intervals based on 20,000 paired resamples. In addition to confidence intervals for paired mean differences, interface-specific mean estimates for the principal response-level outcomes were accompanied by percentile-bootstrap 95% confidence intervals based on 20,000 benchmark-item resamples. Resampling was performed at the benchmark-item level, using the same sampled item indices across interfaces to preserve the matched structure. For binary response-level outcomes, 95% Wilson confidence intervals were reported for the observed proportions. These confidence intervals were used to describe the magnitude and precision of the observed estimates within the present benchmark rather than to imply stable expected performance across repeated generations or future interface versions.

The same matched non-parametric framework was applied to the post hoc Clinical Content Score, management completeness score, and clinical-risk score. Diagnosis/recognition, appropriate escalation/referral, potentially harmful recommendations, and safety-critical omissions were summarized using counts and percentages. For matched binary outcomes with sufficient event counts and variability to support inferential comparison, overall between-interface differences were evaluated using Cochran’s Q test. Holm-adjusted pairwise McNemar tests were planned only following a statistically significant omnibus Cochran’s Q result. Binary outcomes with complete or near-complete category homogeneity or very sparse event counts were summarized descriptively without inferential between-interface comparison. In response to the reviewer-requested assessment of benchmark heterogeneity, an additional post hoc descriptive analysis summarized performance according to the eight clinically oriented complication categories defined in Section 2.4. Category-specific GQS, Clinical Content Score, management-completeness score, and study-specific potential clinical-risk score were summarized by interface. Potentially harmful recommendations and safety-critical omissions were also localized by category. Because the categories were defined post hoc and contained only three to six benchmark items each, no category-level inferential hypothesis tests were performed. For externally observable response latency, an additional post hoc sensitivity analysis was performed to assess the influence of observations recorded as ‘immediate’ and assigned a lower-bound value of 0 s in the primary analysis. Benchmark items containing an ‘immediate’ response were excluded from all four interfaces to preserve the matched repeated-measures structure, and the Friedman test followed by Holm-adjusted pairwise Wilcoxon signed-rank tests was repeated for the remaining complete matched items.

Inter-evaluator agreement for the original GQS assessment was evaluated using the two-way random-effects, absolute-agreement intraclass correlation coefficient for average measurements described above. For the post hoc criterion-referenced assessment, observed percentage agreement was calculated for each domain. Quadratic-weighted Cohen’s kappa was used for ordinal outcomes, including management completeness and clinical-risk classification, whereas unweighted Cohen’s kappa was used for binary domains when sufficient category variability was present. Kappa coefficients were not calculated for outcomes in which both evaluators assigned a single homogeneous category across all responses. Inter-evaluator agreement statistics were interpreted solely as measures of consistency between the two evaluators under the scoring procedures used in this study and not as evidence of construct validity, criterion validity, or external validation of the scoring instruments. Percentage agreement and κ were interpreted cautiously in domains with highly imbalanced or homogeneous category distributions, because high observed agreement may partly reflect limited category variability. When all responses were assigned to a single category, κ was considered non-estimable rather than evidence of perfect reliability.

The benchmark size was content-driven rather than based on an a priori power calculation. No equivalence or non-inferiority margin was specified, and the study was neither designed nor powered to establish equivalence among the evaluated interfaces. Accordingly, non-significant pairwise comparisons were not interpreted as evidence of equivalence or non-inferiority. All 30 benchmark items were retained in the matched analyses. Two-sided adjusted *p* values < 0.05 were considered statistically significant.

All inferential analyses were restricted to the 30 matched benchmark items and the single first-turn outputs generated during the synchronized testing session. Accordingly, *p* values, effect sizes, and pairwise comparisons characterize differences among the sampled outputs under the tested interface configurations and should not be interpreted as estimates of stable expected performance across repeated generations, future interface or model versions, or the broader universe of third molar surgery scenarios.

Because no validated minimal clinically important difference has been established for the task-adapted GQS or the study-specific criterion-referenced outcomes used in this benchmark, statistical significance was interpreted together with effect sizes, confidence intervals, item-level distributions, and the clinical meaning of the underlying scoring domain rather than as evidence of clinical importance by itself.

## 3. Results

### 3.1. Dataset and Inter-Evaluator Agreement

The benchmark generated 120 interface-generated responses from 30 clinical scenarios evaluated across four LLM interfaces. For the original GQS assessment, inter-evaluator agreement was high for all interfaces. The two-way random-effects, absolute-agreement ICC for average measurements was 0.928 for ChatGPT (95% CI, 0.790–1.000), 0.930 for Gemini (95% CI, 0.799–1.000), 0.904 for Copilot (95% CI, 0.717–1.000), and 0.916 for DeepSeek (95% CI, 0.739–1.000); all *p* < 0.001 (Table 3).

The post hoc criterion-referenced and safety-oriented assessment also showed high observed inter-evaluator consistency, although these agreement estimates should be interpreted in the context of the constrained scoring criteria and the limited category variability in several domains. Exact agreement for management completeness was 94.2%, with a quadratic-weighted κ of 0.826. Agreement was 100% for escalation/referral (κ = 1.000) and safety-critical omission (κ = 1.000). For the study-specific potential clinical-risk classification, the evaluators agreed on 119 of 120 responses (99.2%), corresponding to a quadratic-weighted κ of 0.979. Diagnosis/recognition showed 100% observed agreement; κ could not be estimated because all responses were assigned to a single category. For potentially harmful recommendations, both evaluators independently identified the same single response as containing a potentially harmful recommendation, resulting in 100% observed agreement and an unweighted Cohen’s κ of 1.000. These agreement estimates quantify consistency between the two evaluators under the present scoring framework and should not be interpreted as validation of the underlying instruments. In particular, diagnosis/recognition showed complete category homogeneity, with all 120 responses assigned to the same category; therefore, κ was not estimable despite 100% observed agreement. Following the response-level safety re-audit, the potentially harmful-recommendation domain was no longer completely homogeneous: both evaluators independently identified the same single Copilot response as positive, allowing an unweighted κ of 1.000 to be calculated. However, because only one positive event was present, this perfect κ should be interpreted cautiously and does not establish robust reliability or validity across a broader range of responses.

### 3.2. Specialist-Rated Response Quality

Across the 30 matched benchmark items, mean GQSs were 4.53 ± 0.49 for Gemini (95% CI, 4.37–4.70), 4.40 ± 0.48 for ChatGPT (95% CI, 4.23–4.58), 4.30 ± 0.45 for DeepSeek (95% CI, 4.15–4.47), and 3.92 ± 0.51 for Copilot (95% CI, 3.73–4.10).

Holm-adjusted pairwise Wilcoxon signed-rank tests showed that Copilot received significantly lower GQSs than ChatGPT (*p* = 0.012), Gemini (*p* = 0.004), and DeepSeek (*p* = 0.005). No statistically significant pairwise differences were observed between ChatGPT and Gemini (*p* = 0.621), ChatGPT and DeepSeek (*p* = 0.621), or Gemini and DeepSeek (*p* = 0.220). These comparisons describe the 30 matched outputs generated under the tested conditions and should not be interpreted as demonstrating a stable ranking of the interfaces across repeated generations or future versions. The evaluator-specific sensitivity analysis yielded the same overall pattern. For Evaluator 1, the between-interface difference remained significant (Friedman χ^2^ = 16.525, df = 3, *p* < 0.001, Kendall’s W = 0.184). Holm-adjusted pairwise comparisons showed lower GQS ratings for Copilot than for ChatGPT (*p* = 0.017), Gemini (*p* = 0.008), and DeepSeek (*p* = 0.005), with no significant differences among ChatGPT, Gemini, and DeepSeek. For Evaluator 2, the overall difference was likewise significant (Friedman χ^2^ = 17.041, df = 3, *p* < 0.001, Kendall’s W = 0.189). Copilot again received lower ratings than ChatGPT (*p* = 0.026), Gemini (*p* = 0.008), and DeepSeek (*p* = 0.008), whereas the remaining pairwise comparisons were not statistically significant. Thus, the principal GQS pattern was unchanged when the two evaluators’ original ordinal ratings were analyzed separately rather than averaged. Detailed evaluator-specific sensitivity-analysis results are provided in Supplementary Table S7. The response-level GQS distributions further illustrate the overlap among interfaces. Of the 30 evaluator-averaged GQS values, ChatGPT had 11 scores of 5.0, 2 scores of 4.5, and 17 scores of 4.0; Gemini had 15 scores of 5.0, 2 scores of 4.5, and 13 scores of 4.0; DeepSeek had 8 scores of 5.0, 2 scores of 4.5, and 20 scores of 4.0. Copilot showed a broader and lower distribution, with 2 scores of 5.0, 2 scores of 4.5, 20 scores of 4.0, 1 score of 3.5, and 5 scores of 3.0. These distributions, together with the modest GQS omnibus effect size (Kendall’s W = 0.184) and the corresponding confidence intervals, indicate that statistical significance within the 30 matched items should not be interpreted as evidence of broad or stable superiority.

The largest paired effects involved Copilot. Gemini versus Copilot yielded a rank-biserial correlation of 0.819 and a mean paired difference of 0.62 (95% CI, 0.33–0.90), while ChatGPT versus Copilot yielded r_rb = 0.824 and a mean paired difference of 0.48 (95% CI, 0.20–0.77). The Copilot versus DeepSeek comparison yielded r_rb = −1.000 and a mean paired difference of −0.38 (95% CI, −0.55 to −0.22).

### 3.3. Criterion-Referenced Clinical Content and Safety Outcomes

The post hoc criterion-referenced analysis demonstrated a pronounced between-interface difference in the Clinical Content Score. Mean scores were 4.80 ± 0.41 out of 5 for ChatGPT, 4.77 ± 0.43 for Gemini, 4.75 ± 0.47 for DeepSeek, and 3.90 ± 0.59 for Copilot, corresponding to 96.0%, 95.3%, 95.0%, and 78.0% of the maximum possible score, respectively. The overall difference was statistically significant (Friedman χ^2^ = 78.860, df = 3, *p* < 0.001, Kendall’s W = 0.876).

Holm-adjusted pairwise comparisons showed significantly lower Clinical Content Scores for Copilot than for ChatGPT, Gemini, and DeepSeek (all adjusted *p* < 0.001). No significant differences were observed between ChatGPT and Gemini (*p* = 0.635), ChatGPT and DeepSeek (*p* = 0.539), or Gemini and DeepSeek (*p* = 0.635). Within the sampled benchmark outputs, the criterion-referenced content analysis showed lower Clinical Content Scores for Copilot than for the other three interfaces, whereas no statistically significant pairwise differences were identified among ChatGPT, Gemini, and DeepSeek. These non-significant comparisons should not be interpreted as evidence of equivalence or non-inferiority.

To assess whether the observed between-interface pattern was dependent on the greater weighting assigned to management completeness, a post hoc equal-domain sensitivity analysis was performed by rescaling management completeness to a 0–1 range and assigning equal maximum weight to diagnosis/recognition, management completeness, and escalation/referral. Mean Equal-Weight Clinical Content Scores (range, 0–3) were 2.80 ± 0.41 for ChatGPT, 2.77 ± 0.43 for Gemini, 2.76 ± 0.44 for DeepSeek, and 2.46 ± 0.48 for Copilot. The overall between-interface difference remained statistically significant (Friedman χ^2^ = 73.845, df = 3, *p* < 0.001, Kendall’s W = 0.821). Holm-adjusted pairwise comparisons showed lower scores for Copilot than for ChatGPT, Gemini, and DeepSeek (all adjusted *p* < 0.001), whereas no statistically significant differences were observed between ChatGPT and Gemini (*p* = 0.635), ChatGPT and DeepSeek (*p* = 0.539), or Gemini and DeepSeek (*p* = 0.635). Thus, the principal between-interface pattern was unchanged when diagnosis, management, and escalation were assigned equal maximum weight. Detailed results of the equal-domain-weighting sensitivity analysis are provided in Supplementary Table S4. At the item level, ChatGPT received a Clinical Content Score of 5.0 for 24 of 30 items and 4.0 for 6 items; Gemini received 5.0 for 23 items and 4.0 for 7 items; and DeepSeek received 5.0 for 23 items, 4.0 for 6 items, and 3.5 for 1 item. Copilot showed a broader lower-score distribution, with scores of 5.0 for 2 items, 4.5 for 5 items, 4.0 for 15 items, 3.5 for 1 item, and 3.0 for 7 items.

Management completeness showed a similar pattern. Mean management scores were 3.00 ± 0.00 for ChatGPT, 3.00 ± 0.00 for Gemini, 2.98 ± 0.09 for DeepSeek, and 2.17 ± 0.30 for Copilot. The overall difference was significant (Friedman χ^2^ = 82.579, df = 3, *p* < 0.001, Kendall’s W = 0.918). Copilot scored significantly lower than each of the other three interfaces after Holm adjustment (all *p* < 0.001), whereas no significant pairwise differences were identified among ChatGPT, Gemini, and DeepSeek. At the item level, ChatGPT and Gemini received evaluator-averaged management-completeness scores of 3.00 for all 30 benchmark items. DeepSeek received a score of 3.00 for 29 items and 2.50 for one item (Q4). Copilot received a score of 3.00 for two items, 2.50 for six items, and 2.00 for 22 items. Complete item-level management scores are provided in Supplementary Table S5. The absence of score variability for ChatGPT and Gemini should therefore be interpreted as a ceiling effect of this three-element criterion-counting domain rather than as evidence that their management responses were identical in quality or comprehensiveness.

All 120 responses were rated by both evaluators as recognizing the target complication presented in the corresponding benchmark scenario. Because this outcome showed no variability, no inferential between-interface comparison was performed. This finding should be interpreted as target-complication recognition within the information provided in the benchmark items rather than as evidence of independent diagnostic accuracy or diagnostic reasoning performance. As detailed in Supplementary Table S1, the degree of diagnostic cueing varied considerably across items, including scenarios in which the target event was explicitly or nearly explicitly stated, scenarios containing strong characteristic cues, and a smaller subset requiring greater clinical inference.

Appropriate escalation or referral was identified in 24 of 30 ChatGPT responses (80.0%), 23 of 30 Gemini responses (76.7%), 23 of 30 DeepSeek responses (76.7%), and 22 of 30 Copilot responses (73.3%). The overall difference was not statistically significant (Cochran’s Q = 2.400, df = 3, *p* = 0.494).

Among the 120 sampled outputs, one Copilot response met the predefined item-specific criterion for a potentially harmful recommendation. Both evaluators independently identified the same Copilot response to Q16 as meeting the item-specific potentially harmful/contraindicated recommendation criterion. The response advised immediate interruption of warfarin before explicitly conditioning this action on consultation with the prescribing physician or anticoagulation service. No response from ChatGPT, Gemini, or DeepSeek met the study-specific potentially harmful-recommendation criterion within this benchmark. Because only one event was observed, this outcome was summarized descriptively without inferential between-interface comparison.

Safety-critical omissions were identified in two Copilot responses (Q1 and Q20) and in none of the ChatGPT, Gemini, or DeepSeek responses. Both evaluators independently identified the same two Copilot responses. In Q1, which addressed subcutaneous emphysema after use of an air-driven handpiece, the response recognized possible extension into deeper spaces and advised monitoring for airway compromise, but did not explicitly recommend immediate airway assessment as required by the predefined item-specific safety criterion. In Q20, which addressed suspected aspiration or ingestion of a displaced tooth, the response recommended upright positioning, effective coughing, urgent specialist assistance, and radiographic localization but did not explicitly include immediate airway-patency/breathing assessment or management of ineffective cough or complete airway obstruction before proceeding to localization. The Q1 response was classified as low potential clinical risk by both evaluators, whereas the Q20 response was classified as moderate potential clinical risk by both evaluators. Because only two omission events were observed, the overall between-interface difference was not statistically significant (Cochran’s Q = 6.000, df = 3, *p* = 0.112). Further item-level details are provided in Supplementary Table S8. To complement the aggregate and item-level scores with clinically interpretable examples, Supplementary Table S11 presents representative higher-performing and criterion-deficient responses from each interface. The examples illustrate that a response could provide extensive and clinically appropriate management information while still failing to satisfy a specific criterion, particularly explicit escalation/referral. They also illustrate the management-completeness differences observed across interfaces. For example, in the Q20 foreign-body aspiration scenario, ChatGPT, Gemini, and DeepSeek explicitly addressed immediate airway assessment or management of ineffective cough/complete airway obstruction before localization and definitive retrieval, whereas the sampled Copilot response emphasized effective coughing, urgent assistance, and radiographic localization but did not explicitly prioritize immediate airway-patency/breathing assessment or management of ineffective cough or complete obstruction. The latter omission corresponded to the predefined safety-critical-omission criterion for this item. These examples are illustrative only and were not used for additional inferential comparisons.

Detailed Holm-adjusted pairwise comparisons for the post hoc Clinical Content Score, management completeness, and study-specific potential clinical-risk score are provided in Supplementary Table S3.

### 3.4. Study-Specific Potential Clinical-Risk Analysis

Within the sampled benchmark outputs, study-specific potential clinical-risk scores differed across the four evaluated interfaces. Mean evaluator-averaged risk scores were 0.00 for Gemini, 0.02 ± 0.09 for ChatGPT, 0.03 ± 0.18 for DeepSeek, and 0.90 ± 0.40 for Copilot. The overall difference was statistically significant (Friedman χ^2^ = 74.698, df = 3, *p* < 0.001, Kendall’s W = 0.830). Holm-adjusted pairwise analyses showed higher clinical-risk scores for Copilot than for ChatGPT, Gemini, and DeepSeek within this benchmark (all adjusted *p* < 0.001). No statistically significant pairwise differences were observed among ChatGPT, Gemini, and DeepSeek.

Holm-adjusted pairwise analyses showed significantly higher clinical-risk scores for Copilot than for ChatGPT, Gemini, and DeepSeek (all adjusted *p* < 0.001). No statistically significant pairwise differences were observed among ChatGPT, Gemini, and DeepSeek.

Across the pooled evaluator-level risk classifications (60 ratings per interface), 59 of 60 ChatGPT ratings (98.3%) were classified as no clinically relevant risk and one (1.7%) as low risk. All 60 Gemini ratings (100%) were classified as no clinically relevant risk. For DeepSeek, 58 of 60 ratings (96.7%) were classified as no clinically relevant risk and two (3.3%) as low risk. In contrast, among Copilot ratings, 8 of 60 (13.3%) were classified as no clinically relevant risk, 50 of 60 (83.3%) as low risk, and 2 of 60 (3.3%) as moderate risk. Within the 120 sampled outputs, no response met the study-specific criteria for the high-risk category. Given that only 30 scenarios were evaluated per interface and each scenario was generated only once, this absence should not be interpreted as evidence that high-risk responses cannot occur under other prompts, repeated generations, interface configurations, or future versions.

The two moderate-risk ratings corresponded to the same Copilot response. Clinical-risk distributions are shown in Figure 2.

A summary of the principal specialist-rated, criterion-referenced, safety, study-specific potential clinical-risk, and externally observable response latency outcomes is provided in Table 4.

### 3.5. Externally Observable Response Latency

Externally observable response latency differed significantly across the evaluated interfaces (Friedman χ^2^ = 70.923, df = 3, *p* < 0.001, Kendall’s W = 0.788). Mean externally observable response latencies were 13.17 ± 7.46 s for ChatGPT, 15.87 ± 9.08 s for Gemini, 1.23 ± 1.87 s for Copilot, and 6.80 ± 4.28 s for DeepSeek.

Copilot showed significantly shorter externally observable response latency than ChatGPT, Gemini, and DeepSeek (all Holm-adjusted *p* < 0.001), while DeepSeek showed significantly shorter externally observable response latency than ChatGPT and Gemini (both *p* < 0.001). No statistically significant difference was observed between ChatGPT and Gemini after Holm adjustment (*p* = 0.094).

The largest paired differences were observed for Gemini versus Copilot (14.63 s; 95% CI, 12.20–17.93; r_rb = 1.000), ChatGPT versus Copilot (11.93 s; 95% CI, 9.43–14.67; r_rb = 1.000), and Gemini versus DeepSeek (9.07 s; 95% CI, 6.53–12.00; r_rb = 0.938).

Nineteen of the 30 Copilot responses (63.3%) were recorded as ‘immediate,’ whereas no responses from ChatGPT, Gemini, or DeepSeek were assigned this category. Because these observations were represented by a lower-bound value of 0 s in the primary analysis, an exclusion-based sensitivity analysis was performed. After removing the 19 benchmark items containing an ‘immediate’ Copilot response from all four interfaces, 11 complete matched items remained. Mean externally observable response latencies in this subset were 14.36 ± 9.60 s for ChatGPT, 17.82 ± 14.12 s for Gemini, 8.73 ± 6.48 s for DeepSeek, and 3.36 ± 1.50 s for Copilot. The overall between-interface difference remained statistically significant (Friedman χ^2^ = 23.618, df = 3, *p* < 0.001, Kendall’s W = 0.716). After Holm adjustment, Copilot remained significantly shorter than ChatGPT (*p* = 0.006), Gemini (*p* = 0.006), and DeepSeek (*p* = 0.012). DeepSeek remained significantly shorter than Gemini (*p* = 0.023), whereas the DeepSeek–ChatGPT comparison was no longer statistically significant (*p* = 0.305); ChatGPT and Gemini also did not differ significantly (*p* = 0.577). Detailed results of the exclusion-based response-latency sensitivity analysis are provided in Supplementary Table S6.

Given the manual whole-second timing procedure and lower-bound coding of ‘immediate’ responses, these latency values should be interpreted as descriptive, approximate user-observable characteristics of the tested interfaces rather than as precise estimates of intrinsic response latency or computational performance.

### 3.6. Readability Analysis

Readability differed significantly across the four evaluated interfaces for all five indices. Mean Flesch–Kincaid Grade Level values were 13.13 ± 2.41 for ChatGPT, 10.89 ± 1.24 for Gemini, 12.66 ± 0.98 for Copilot, and 11.63 ± 2.42 for DeepSeek. Corresponding Gunning Fog Index values were 16.63 ± 2.99, 14.62 ± 1.59, 16.20 ± 1.25, and 15.89 ± 3.05, respectively. Mean Coleman–Liau Index values were 16.78 ± 1.70 for ChatGPT, 14.98 ± 1.45 for Gemini, 16.83 ± 1.16 for Copilot, and 14.85 ± 1.96 for DeepSeek.

Significant overall between-interface differences were identified for Flesch–Kincaid Grade Level (χ^2^ = 30.519, *p* < 0.001, Kendall’s W = 0.339), Gunning Fog Index (χ^2^ = 17.909, *p* < 0.001, W = 0.199), Coleman–Liau Index (χ^2^ = 52.091, *p* < 0.001, W = 0.579), Simple Measure of Gobbledygook (χ^2^ = 28.485, *p* < 0.001, W = 0.316), and Flesch Reading Ease Score (χ^2^ = 15.640, *p* = 0.001, W = 0.174) (Table 5).

Gemini and DeepSeek generally produced lower grade-level and complexity scores and higher Flesch Reading Ease Scores than ChatGPT and Copilot. These differences indicate lower surface linguistic complexity according to the applied readability formulas and should not be interpreted as evidence of better clinical communication or greater appropriateness for the intended specialist audience.

Because the five indices have overlapping mathematical components, these results were interpreted as a convergent pattern across alternative readability formulas rather than as five statistically independent effects. No statistically significant differences were observed between Gemini and DeepSeek for any of the five readability measures after Holm adjustment; detailed pairwise comparisons are provided in Supplementary Table S9.

### 3.7. Exploratory Performance by Complication Category

The 30 benchmark scenarios were distributed across eight clinically oriented categories, with category sizes ranging from three to six items. Descriptive category-level analysis showed that the magnitude of between-interface separation varied across complication types (Supplementary Table S10). For Clinical Content Score, the largest numerical separation involving Copilot was observed for mechanical/structural/technical injuries (3.50 vs. 4.80 for each of ChatGPT, Gemini, and DeepSeek), followed by neurologic complications (3.67 vs. 4.67 for each of the other three interfaces) and osteonecrosis/impaired bone-healing scenarios (4.00 vs. 5.00 for each of the other three interfaces). The numerical separation was smaller for infectious/deep-space complications, for which mean Clinical Content Scores were 4.63 for Copilot and 5.00 for each of the other three interfaces. These category-level findings are descriptive and exploratory because each category contained only three to six benchmark items. The single response meeting the study-specific potentially harmful-recommendation criterion occurred in the hemorrhagic/anticoagulation category (Copilot, Q16). The two responses meeting the predefined safety-critical-omission criterion occurred in the mechanical/structural/technical category (Copilot, Q1) and the displacement/foreign-body/oroantral category (Copilot, Q20).

## 4. Discussion

The present study provides a multidimensional comparison of the sampled outputs from four contemporary LLM interfaces within a standardized single-generation benchmark of specialist-level third molar surgery complication scenarios. Within these sampled outputs, differences were observed not only in global specialist-rated response quality but also in the completeness and potential clinical consequences of the generated management recommendations. These observations characterize the single responses generated under the specific interface configurations and testing conditions used and should not be interpreted as estimates of stable interface performance across repeated generations. At the global-quality level, the sampled outputs from ChatGPT, Gemini, and DeepSeek showed substantial overlap, whereas the clearest separation involved the sampled Copilot outputs. More importantly, the post hoc criterion-referenced analysis suggested that the principal clinical distinction was related to management completeness rather than simple recognition of the target complication. Clinically, this distinction is important because correctly identifying a complication—or even recognizing the need for specialist involvement—does not ensure that all essential management steps are appropriately prioritized or communicated. The sampled Copilot outputs therefore showed a less favorable pattern primarily because essential management elements were more frequently absent or insufficiently explicit, accompanied by a less favorable study-specific potential clinical-risk profile. These findings characterize the sampled outputs under the specific tested conditions and do not establish a stable clinical ranking among the interfaces. Accordingly, the observed between-interface differences should be interpreted as exploratory and hypothesis-generating rather than as confirmatory evidence of stable comparative performance.

The less favorable pattern observed for Copilot cannot be attributed to a single technical or model-level cause. The present study evaluated public interface–configuration combinations rather than isolated underlying language models, and the four interface–configuration combinations were tested using different provider-specific modes. Provider-side system instructions, routing, retrieval or augmentation behavior, contextualization, interface-level formatting policies, and other implementation details were not fully observable or experimentally standardized. Response length and structure also varied naturally across the generated outputs and were not evaluated as causal explanatory variables. Moreover, because each scenario was generated only once per interface, stochastic variation in the sampled responses cannot be separated from persistent interface-level differences. Accordingly, the present data support only the conclusion that the sampled Copilot outputs received lower specialist-rated quality and management-completeness scores under the tested conditions; they do not establish that the underlying language model accessed through Copilot was intrinsically inferior, nor do they identify the selected mode, system prompting, interface design, response length, retrieval behavior, or any other individual factor as the cause of the observed differences. Clinical generalizability is also inherently limited by the standardized text-only benchmark design. Real-world management of third molar complications is individualized and frequently depends on information that cannot be fully represented in a written vignette, including direct clinical examination, vital signs and evolving symptoms, the timing and trajectory of the complication, panoramic or CBCT findings, current medications, relevant comorbidities, previous treatment, and the available treatment setting and resources. These factors may materially alter diagnostic interpretation, urgency, management priorities, and thresholds for specialist referral or emergency escalation. Accordingly, even a response that performs well against the predefined criteria of a standardized scenario should not be interpreted as demonstrating the ability to manage an actual patient. The present benchmark evaluates responses to the information supplied in text form; it does not evaluate integration of evolving multimodal patient data, bedside examination, clinician–patient interaction, or adaptation of management to an individual clinical environment.

All 120 responses were rated as recognizing the target complication presented in the corresponding benchmark scenario. This finding should not be interpreted as evidence of equivalent diagnostic accuracy or independent diagnostic reasoning, because several scenarios explicitly named the complication or provided strong diagnostic cues and the benchmark was primarily management-focused. More informative between-interface differences emerged after recognition: management completeness showed marked separation, whereas escalation/referral performance was comparatively similar across interfaces. Thus, correctly identifying the intended complication—or recognizing the need for specialist involvement—did not necessarily ensure complete, appropriately prioritized, or risk-sensitive management. The present study should therefore be interpreted as a benchmark of specialist-level complication management rather than as a comparative diagnostic-accuracy study.

The absence of variability in the ChatGPT and Gemini management scores should also be interpreted in light of the structure of the management-completeness domain. This subscore was deliberately criterion based and contained only three prespecified essential elements per scenario; it was not designed to discriminate finer differences in clinical reasoning, explanatory depth, or organization once all three elements were adequately addressed. For example, in Q30, complete management required conservative local care, allowing the sequestrum to demarcate, and gentle removal once loose. A response addressing all three elements received a score of 3, whereas a response providing conservative care and subsequent sequestrum removal without explicitly addressing the demarcation period received a lower score. Thus, the 3.00 ± 0.00 values for ChatGPT and Gemini represent a ceiling effect within this specific criterion-counting domain and should not be interpreted as evidence of identical or universally complete clinical performance. Accordingly, the magnitude of the observed between-interface separation in management completeness should be interpreted in light of this ceiling effect and the restricted three-element scoring range.

The modest omnibus effect size for GQS compared with the larger effects observed for the post hoc criterion-referenced outcomes suggests that domain-specific scoring was more discriminating within this benchmark. A global quality score represents a holistic judgment of the response as a whole and therefore integrates multiple features, including overall accuracy, organization, completeness, coherence, and practical usefulness. Consequently, an otherwise well-structured and clinically appropriate response may still receive a favorable global rating despite omitting a single management step, escalation requirement, or safety-critical element. Such an omission may have limited influence on the overall global impression while carrying disproportionate clinical importance in a specific scenario. By contrast, the criterion-referenced framework required explicit assessment of predefined item-specific management, escalation, and omission criteria, making these localized deficiencies more visible. This distinction is consistent with concerns that aggregate measures may obscure clinically meaningful error patterns [19]. The representative examples in Supplementary Table S11 provide a clinical illustration of this distinction. In particular, some responses with favorable global ratings still lacked an explicitly required escalation/referral element, whereas the lower management-completeness scores observed more frequently for Copilot reflected responses in which one or more predefined management elements were absent or insufficiently explicit. These examples are illustrative rather than statistically representative and should be interpreted together with the complete item-level distributions and archived response set. However, because the criterion-referenced framework was developed post hoc and specifically for this study, its greater separation should not be interpreted as evidence that it is intrinsically more valid or superior to the GQS; these findings remain exploratory and hypothesis-generating.

The exploratory category-level analysis further indicated that the observed between-interface differences were not confined to a single type of complication, although their numerical magnitude varied across clinical categories. The largest descriptive Clinical Content separation was observed in mechanical/structural/technical, neurologic, and osteonecrosis/impaired bone-healing scenarios, whereas the difference was smaller in infectious/deep-space complications. These patterns should not be interpreted as evidence of category-specific superiority or inferiority, because the grouping was post hoc, each category contained only three to six items, and each scenario was generated only once per interface. Larger prospectively stratified benchmarks are required to determine whether reproducible domain-specific performance differences exist.

The safety-oriented findings warrant cautious interpretation. Following re-audit of the response-level safety classifications, one potentially harmful recommendation was identified in the Copilot response to the warfarin-related hemorrhage scenario. Specifically, the response advised immediate interruption of warfarin before explicitly conditioning this action on consultation with the prescribing clinician or anticoagulation service. Both evaluators nevertheless classified the overall potential clinical risk of this response as low risk (score = 1), reflecting the fact that the response also provided appropriate local hemostatic measures, urgent INR assessment, and subsequent medical coordination. No potentially harmful recommendation was identified in the evaluated ChatGPT, Gemini, or DeepSeek responses. This isolated event should be interpreted as a benchmark-specific potential clinical-risk signal rather than evidence of a general safety characteristic of the interface. The small number of observed risk-related events precludes broader conclusions regarding the clinical safety of any evaluated interface [20].

Readability was evaluated as a secondary descriptive dimension of the sampled outputs and was interpreted cautiously in the context of the specialist-level oral and maxillofacial surgery audience. Gemini and DeepSeek generally produced responses with lower grade-level and complexity indices and higher reading-ease scores than ChatGPT and Copilot. However, the intended readership in the present benchmark was a professional oral and maxillofacial surgery audience, because the standardized prompt explicitly requested specialist-level responses. The readability indices were therefore used to characterize surface linguistic complexity and potential reading burden within professional clinical text rather than to define an optimal reading level or to evaluate patient-facing comprehensibility. Their practical relevance lies in describing how linguistically dense or structurally demanding an interface-generated specialist response may be for a professional reader; they do not establish whether the response communicates the clinical content more effectively. The five indices are not statistically independent because their formulas share overlapping linguistic components. They were nevertheless retained to reduce dependence on any single readability formula and to examine whether the general between-interface pattern was robust across partly different operationalizations of surface linguistic complexity. Their concordance should therefore be interpreted as robustness across formula choice rather than as multiple independent confirmations of a readability effect; moreover, none of these indices has been validated as an OMFS-specific measure of specialist communication quality. Accordingly, lower grade-level or higher reading-ease values should not automatically be interpreted as better clinical communication, just as greater linguistic complexity should not automatically be considered a deficiency in a specialist-level response. Importantly, formula-based readability and specialist-rated clinical quality represent conceptually distinct constructs. A response may be clinically strong but linguistically complex, or comparatively readable while lacking essential management content. Future evaluations should therefore align readability targets with the intended user population and, ideally, complement formula-based readability indices with direct measures of specialist comprehension, cognitive workload, information retrieval efficiency, and perceived usability.

As a secondary, manually measured interface-level outcome, externally observable response latency differed across the sampled interface outputs. Copilot showed the shortest observed externally observable response latency, followed by DeepSeek, whereas ChatGPT and Gemini required longer observed response latencies. However, within the sampled benchmark outputs, Copilot simultaneously received the lowest GQS and Clinical Content Scores and the highest observed potential clinical-risk score. The co-occurrence of shorter observed response latency and lower clinical-content ratings for Copilot should not be interpreted as evidence that response speed, response brevity, or any other measured interface characteristic caused the lower clinical scores. Differences in answer length, interface behavior, server conditions, model architecture, and provider-side processing may all contribute to externally observable response latency. Moreover, the timing method measured the interval from prompt submission until visible generation ceased rather than time to first token or internal computational latency. Accordingly, this outcome should be regarded as a user-observable interface characteristic under the tested conditions rather than as a measure of model efficiency, computational efficiency, internal latency, or computational resource use. An additional limitation of the externally observable response-latency analysis is that interface order was fixed rather than randomized or counterbalanced. For each benchmark question, the interfaces were queried sequentially in the same order (ChatGPT → DeepSeek → Gemini → Copilot). Although testing was conducted during the same synchronized session using the same hardware and internet connection, short-term variation in provider-side server load, routing, local network conditions, or other time-dependent could have influenced the measured externally observable response latencies. Consequently, some of the observed latency differences may reflect temporal or order-related effects in addition to interface-specific behavior. Future studies should use randomized or counterbalanced interface order and automated latency logging to better control these sources of variability.

The manual timing procedure also resulted in substantial lower-bound censoring for Copilot, for which 19 of 30 responses were recorded as ‘immediate’ rather than with an exact elapsed duration. Although the exclusion-based sensitivity analysis preserved the overall between-interface difference and the shorter externally observed response latencies for Copilot relative to each of the other interfaces, some secondary pairwise findings changed; notably, the DeepSeek–ChatGPT comparison was no longer statistically significant. This indicates that detailed latency contrasts are sensitive to the handling of observations below the practical timing resolution. Accordingly, the latency findings should be regarded as approximate user-observable interface characteristics under the tested conditions rather than precise latency estimates. Future studies should employ automated timestamp logging with subsecond resolution.

The present findings both support and extend previous dental LLM research. Sezer and Aydoğdu [17] reported model-dependent differences in accuracy, completeness, readability, and response time in dental-trauma scenarios; similarly, the present study identified interface-dependent differences across multiple response dimensions, although the clearest clinically relevant separation in our benchmark involved management completeness rather than recognition of the target complication. The findings also complement those of Jacobs et al. [18], who reported generally accurate and clinically relevant ChatGPT-generated information on third molar extraction while noting relatively high linguistic complexity. In the present specialist-level benchmark, ChatGPT likewise showed high clinical-content performance together with comparatively greater formula-derived linguistic complexity; however, because the intended audience here was specialist clinicians rather than patients, greater linguistic complexity should not itself be interpreted as poorer communication. In contrast to the variable expert agreement reported for AI-based impacted-third-molar classification by Erden et al. [16], all sampled responses in the present study were rated as recognizing the target complication. This apparent difference is likely related to task design, because several of our scenarios contained explicit or strong diagnostic cues and were designed primarily to evaluate complication management rather than independent diagnostic accuracy. Finally, the observation that globally favorable responses could still omit specific management or escalation elements is consistent with the emphasis of Ossowska et al. [19] on clinically meaningful error patterns that may not be adequately represented by aggregate performance measures alone.

The present findings can also be contextualized within the broader medical LLM literature. Goodman et al. [26] reported generally favorable accuracy and completeness of chatbot responses to physician-generated questions across multiple medical specialties, while also emphasizing the need for verification before clinical integration. This is broadly consistent with our observation that favorable global response-quality and clinical-content ratings can coexist with isolated management deficiencies or safety-relevant omissions. In contrast, Hager et al. [27], using a more clinically realistic patient-based decision-making framework, identified limitations in diagnostic performance, guideline adherence, information integration, and robustness. The generally favorable performance of several interfaces in the present standardized text-only benchmark should not be regarded as contradictory to those findings. Rather, the difference underscores the dependence of LLM performance on task design, available clinical information, and evaluation context, while both lines of evidence support caution against equating favorable benchmark performance with readiness for autonomous clinical decision-making. These findings are also broadly consistent with recent systematic reviews focused specifically on LLM applications in oral and maxillofacial surgery. Torres et al. [28] concluded that ChatGPT showed promising performance across several OMFS applications but remained limited in complex clinical decision-making and personalized advice, emphasizing the continued need for human supervision. Similarly, Ronsivalle et al. [29] found that LLM-based chatbots may provide useful support in routine OMFS contexts but that performance remains constrained in more complex scenarios and may vary according to question formulation. The present findings are consistent with these conclusions in showing that favorable overall response quality can coexist with clinically important omissions and task-dependent variability. At the same time, the comparatively high scores observed for several interfaces in our standardized benchmark should not be interpreted as contradicting these reviews, because differences in model generation, task structure, prompting, evaluation criteria, and clinical complexity substantially limit direct cross-study comparisons.

Several methodological features strengthen the present study. All four interfaces were evaluated using the same 30 benchmark scenarios in a matched design, under a synchronized testing protocol using the same hardware and internet connection. Each prompt was administered in an isolated single-turn session, and only the first visible response was retained, reducing contextual carry-over and selective regeneration. Direct interface-identifying information was removed before assessment, and two experienced oral and maxillofacial surgeons independently evaluated the responses. Inter-evaluator agreement was high for both the original GQS assessment and the subsequent criterion-referenced safety-oriented evaluation. The study also examined complementary dimensions of specialist-rated quality, clinical content, safety, readability, and externally observable response latency rather than reducing performance to a single metric. Finally, preservation of the archived interface-generated responses improves transparency and allows the reported assessments to be scrutinized against the original outputs.

Several limitations should be considered when interpreting the results. First, the 30-item benchmark was investigator-developed and content-driven and was not externally developed or validated as a psychometric specialty examination. The scenario set was not constructed using prevalence-based sampling or prespecified quotas balancing common versus rare or routine versus emergency complications. Consequently, the composition of the benchmark reflects deliberate clinical breadth rather than the epidemiologic distribution of third molar complications in practice, and the scenario mix may have influenced both absolute scores and observed between-interface differences. Likewise, because the item-specific criterion-referenced framework was developed specifically for this study, the very high Clinical Content Scores observed for ChatGPT, Gemini, and DeepSeek may partly reflect alignment between the selected benchmark scenarios and the study-specific rubric rather than generalizable superiority in clinical performance. These findings should therefore be interpreted as performance within this benchmark and require confirmation using externally developed and independently validated scenario sets and scoring frameworks. The statistically significant differences observed in this 30-item matched benchmark should therefore not be equated with robust or generalizable superiority of one interface over another. Statistical significance reflects the pattern of the sampled matched outputs and should be interpreted jointly with effect sizes, confidence intervals, and item-level score distributions. In particular, overlapping score distributions and the relatively modest Kendall’s W for the original GQS outcome indicate that the magnitude of between-interface separation differed substantially across outcome domains. Larger externally developed benchmark sets with repeated generations are required before stable comparative performance rankings can be established. Second, each scenario was submitted only once to each interface. Because LLM outputs are stochastic, each retained response represents only a single realization from the range of outputs that the same interface–configuration could potentially generate for the same prompt. The study therefore cannot characterize within-interface output variability, reproducibility across regenerations, or temporal stability, nor can it estimate the distribution of expected performance for any interface. Consequently, some of the observed between-interface differences may reflect stochastic sampling variability in the particular responses generated during the testing session rather than stable differences between the evaluated interfaces. Repeated independent generations would be required to distinguish persistent between-interface differences from within-interface output variability. Performance may also be sensitive to prompt formulation. Although all interfaces received the same standardized instruction and scenario wording, the present study evaluated only one prompt formulation for each benchmark item. Alternative wording, differences in contextual detail, role specification, or iterative follow-up prompting could produce different responses. Accordingly, the present findings should not be generalized to other prompting strategies or to multi-turn clinician–LLM interactions. Third, the results represent a single time point and the specific publicly accessible interfaces and modes available on the testing date. A related limitation is that the four interfaces were evaluated under different provider-specific interface modes—Thinking, reasoning, conversational, and Work and Learning—which are not standardized equivalents. These configurations may influence response length and structure, reasoning behavior, retrieval or augmentation, externally observable response latency, and clinical-content generation. In addition, provider-side system instructions, routing logic, interface-level formatting or contextualization, and other backend implementation details were not observable or experimentally controlled. Because interface mode and underlying model identity were not experimentally separable in the present design, mode/configuration constitutes an important potential confounder. The present data therefore cannot determine whether an observed between-interface difference arose from the underlying language model, the selected interface mode, provider-side routing or augmentation, or an interaction among these factors. Provider-side model updates, routing changes, or undisclosed backend revisions may alter performance over time. Accordingly, the inferential target of the present analyses is limited to the matched outputs generated for these 30 benchmark items during the single synchronized testing session. The observed statistical differences should not be interpreted as demonstrating stable differences in the expected behavior of the interfaces across repeated generations, future provider updates, or unsampled third molar surgery scenarios. A further limitation concerns interface-specific retrieval, personalization, and contextual features. Although prompt wording, hardware, internet connection, testing time, language, and first-turn conditions were standardized, the underlying information-access behavior of the public interfaces could not be made equivalent. Some Copilot responses contained visible external-source labels and geographically tailored recommendations that were not present in the benchmark prompts, indicating that provider-side retrieval or contextual signals may have influenced the generated output. Because comparable retrieval and personalization functions were not uniformly observable or controllable across interfaces, part of the observed between-interface variation may reflect differences in interface-level information access and contextualization rather than differences attributable solely to the underlying language models. The findings should therefore be interpreted as comparisons of the tested public interfaces under the specific access conditions used, rather than as isolated comparisons of underlying model architectures. Fourth, the English-only, single-turn, text-only design limits clinical generalizability. As the benchmark did not reproduce patient-specific examination, imaging, medication, comorbidity, temporal, or treatment-setting information in the manner available during real-world care, the findings should not be extrapolated to individualized multimodal clinical decision-making. An additional limitation concerns the international heterogeneity of oral and maxillofacial surgery training and scope of practice. The standardized instruction requested responses at a specialist level in oral and maxillofacial surgery but did not specify a national jurisdiction or a dental-, medical-, or dual-degree training pathway. Because qualification requirements, professional competencies, referral pathways, and permissible scopes of practice vary across healthcare systems, the interfaces may have generated recommendations based on different implicit assumptions regarding the role of an oral and maxillofacial surgeon. Accordingly, the findings should be interpreted within a generic specialist-level OMFS context and should not be assumed to represent the training standards, regulatory framework, or clinical practice boundaries of any particular country.

Additional limitations relate specifically to the assessment framework. The GQS was task-adapted and has not been independently validated for specialist-level LLM benchmarking. Although the primary GQS analysis used the mean of two ordinal evaluator ratings as a pragmatic response-level composite, evaluator-specific sensitivity analyses using the original ordinal scores reproduced the same principal between-interface pattern, reducing concern that the main finding was driven by averaging across raters. Nevertheless, averaging ordinal ratings does not convert the GQS into an interval-scale measure, and the resulting means should be interpreted as descriptive composite summaries rather than interval-level measurements. The criterion-referenced clinical-content and risk analyses were added post hoc, and the item-specific criteria and weighting scheme were developed specifically for this study. Although the item-specific criteria were developed without consulting the generated responses or the original GQS results and were locked before criterion-referenced rescoring began, the framework itself was nevertheless developed post hoc, after completion of the original global assessment. Consequently, the possibility of design-stage bias cannot be entirely excluded. The substantially greater between-interface separation observed for the Clinical Content Score, management-completeness score, and study-specific potential clinical-risk classification than for the original GQS should not be interpreted as evidence that the post hoc framework is intrinsically more valid or superior as a measurement instrument. Rather, this stronger separation may partly reflect the more constrained, domain-specific structure of the criterion-referenced framework and the greater weighting assigned to management completeness. These analyses should therefore be regarded as exploratory and hypothesis-generating and require prospective validation using criteria specified before response generation in future studies. Similarly, the high inter-evaluator agreement observed in this study should not be interpreted as validation of the scoring instruments. Agreement reflects reproducibility of judgments between the two evaluators under the present rubric, whereas validation requires separate evidence regarding whether an instrument measures the intended construct appropriately and generalizes beyond the present sample and raters. The highly constrained item-specific criteria and the limited or absent category variability in several domains may have contributed to the high agreement estimates. For example, diagnosis/recognition showed 100% observed agreement but no variability, precluding estimation of κ. In the potentially harmful-recommendation domain, the updated dataset contained only one positive event, and both evaluators identified that same response, yielding κ = 1.000; nevertheless, such sparse-event agreement provides limited information about reliability across a broader spectrum of clinically ambiguous or harmful outputs. Future validation should involve prospectively specified criteria, larger and more heterogeneous benchmark sets, additional independent evaluators, and external assessment of the measurement properties of the framework. In particular, management completeness contributed three of the five Clinical Content Score points, reflecting the management-focused design but also giving that domain greater influence on the aggregate score. The reviewer-requested sensitivity analysis using equal maximum weighting of diagnosis/recognition, management completeness, and escalation/referral produced the same inferential pattern: the sampled outputs generated through the tested Copilot interface–configuration remained lower than those from each of the other three tested interface–configurations, whereas no statistically significant pairwise differences were identified among the sampled ChatGPT, Gemini, and DeepSeek outputs. The latter finding should not be interpreted as evidence of equivalence or clinical comparability among these three interfaces, because no equivalence or non-inferiority margin was prespecified and the benchmark contained only 30 matched scenarios. This finding indicates that the principal between-interface separation observed in the present benchmark was not solely attributable to the three-point weighting of management completeness. Nevertheless, the alternative weighting remains a post hoc analysis of the same study-specific framework and should not be interpreted as external validation of the Clinical Content Score. The study-specific potential clinical-risk classification likewise represents a study-specific framework conceptually informed by patient-safety principles rather than an externally validated clinical risk scale [25]. Although agreement between the two evaluators was high, clinical judgments regarding completeness and potential risk necessarily contain a degree of specialist interpretation. The small number of safety-related events further limited statistical inference for the binary safety outcomes. In particular, the single potentially harmful recommendation observed in this dataset cannot be used to estimate the frequency of such events under different prompts, repeated generations, or future interface versions. Likewise, the absence of responses classified as high risk within the present benchmark should not be interpreted as reassuring evidence of safety. With only 30 scenarios per interface and a single generation for each scenario, the study had limited opportunity to detect rare but clinically important failures. High-risk outputs may therefore occur under different prompts, repeated generations, interface configurations, temporal conditions, or future versions even though none were observed in the sampled responses.

Source masking was also necessarily incomplete. Although interface names, logos, and direct branding cues were removed, characteristic formatting, verbosity, or writing style may have allowed evaluators to infer model origin in some cases. Externally observable response latency was measured manually with a digital stopwatch, and values recorded as ‘immediate’ were represented by a lower-bound value of zero seconds; these measurements consequently lack the precision of automated latency logging. Most importantly, no interface-generated recommendation was used in actual patient care. The study therefore assessed simulated textual performance and potential clinical risk, not observed clinical harm, changes in clinician behavior, patient outcomes, or real-world effectiveness.

Future research should address these limitations through larger externally developed benchmark sets, repeated independent generations, longitudinal testing across model updates, and multicenter evaluation involving a broader group of specialists. Safety-oriented frameworks should ideally be prospectively specified and externally validated, with explicit weighting of diagnostic, management, escalation, and harm-related domains. Multilingual testing and multimodal scenarios incorporating panoramic radiographs and CBCT would provide a closer approximation to actual oral and maxillofacial surgical practice. Clinician-in-the-loop studies are also needed to determine whether LLM-generated recommendations improve or impair decision-making when used as adjunctive tools. Until such evidence is available, the present findings support further evaluation of LLM interfaces as potential adjunctive information tools, subject to specialist verification, rather than establishing their benefit or appropriateness for clinical use.

## 5. Conclusions

In this standardized, single-generation benchmark of 30 specialist-level third molar surgery complication scenarios, the sampled outputs showed different performance profiles under the specific interface configurations and testing conditions examined. Among the sampled outputs, Gemini had the highest numerical mean GQS, followed by ChatGPT and DeepSeek, whereas Copilot received significantly lower specialist-rated global quality scores within these 30 matched benchmark items. In the post hoc criterion-referenced analysis, the principal between-interface separation was observed in management completeness. ChatGPT, Gemini, and DeepSeek each had numerically high Clinical Content Scores within the sampled benchmark, whereas the sampled Copilot outputs showed lower management completeness and a less favorable potential clinical-risk profile. However, pairwise tests that did not reach statistical significance among ChatGPT, Gemini, and DeepSeek do not establish clinical equivalence, non-inferiority, or comparable expected performance. The present study was neither designed nor powered for equivalence testing, and only 30 matched scenarios were evaluated per interface.

All evaluated responses were rated as recognizing the target complication; however, because several benchmark scenarios contained explicit or strong diagnostic cues, this finding reflects recognition within the provided scenarios rather than independently established diagnostic accuracy. Readability and externally observable response latency also differed across interfaces, but these outcomes represent complementary interface characteristics rather than measures of clinical effectiveness or computational efficiency. Within these sampled outputs, one Copilot response met the study-specific criterion for a potentially harmful recommendation; however, its overall potential clinical-risk classification remained low. This isolated observation, together with the small number of safety-related events, does not establish the frequency with which potentially harmful recommendations may occur and should not be interpreted as evidence for or against the general clinical safety of any evaluated interface. Similarly, the absence of high-risk classifications in these 30 single-generation scenarios per interface should not be interpreted as evidence that rare, clinically important high-risk failures do not occur. Overall, the findings indicate that global response quality alone may not capture clinically relevant differences in management completeness and potential consequences of omissions. Because the benchmark was text-only and did not reproduce the patient-specific examination, imaging, medication, comorbidity, temporal, and treatment-setting information that informs real-world third molar complication management, these results should not be extrapolated to individualized clinical decision-making. Accordingly, the comparative findings of this benchmark should be regarded as exploratory and hypothesis-generating rather than confirmatory. These results apply only to the sampled outputs generated during the single synchronized testing session. Because each prompt was submitted once to each interface, the study cannot distinguish stable between-interface differences from stochastic variation across repeated generations. The findings therefore do not establish persistent performance superiority, clinical safety, effectiveness, or readiness for autonomous clinical decision support and require confirmation using repeated independent generations and prospective external validation. Accordingly, the present findings support further prospective evaluation of LLM interfaces as potential adjunctive information tools, subject to specialist verification; they do not establish that such use is beneficial, safe, or appropriate in clinical practice.

## Figures and Tables

**Figure 1:**
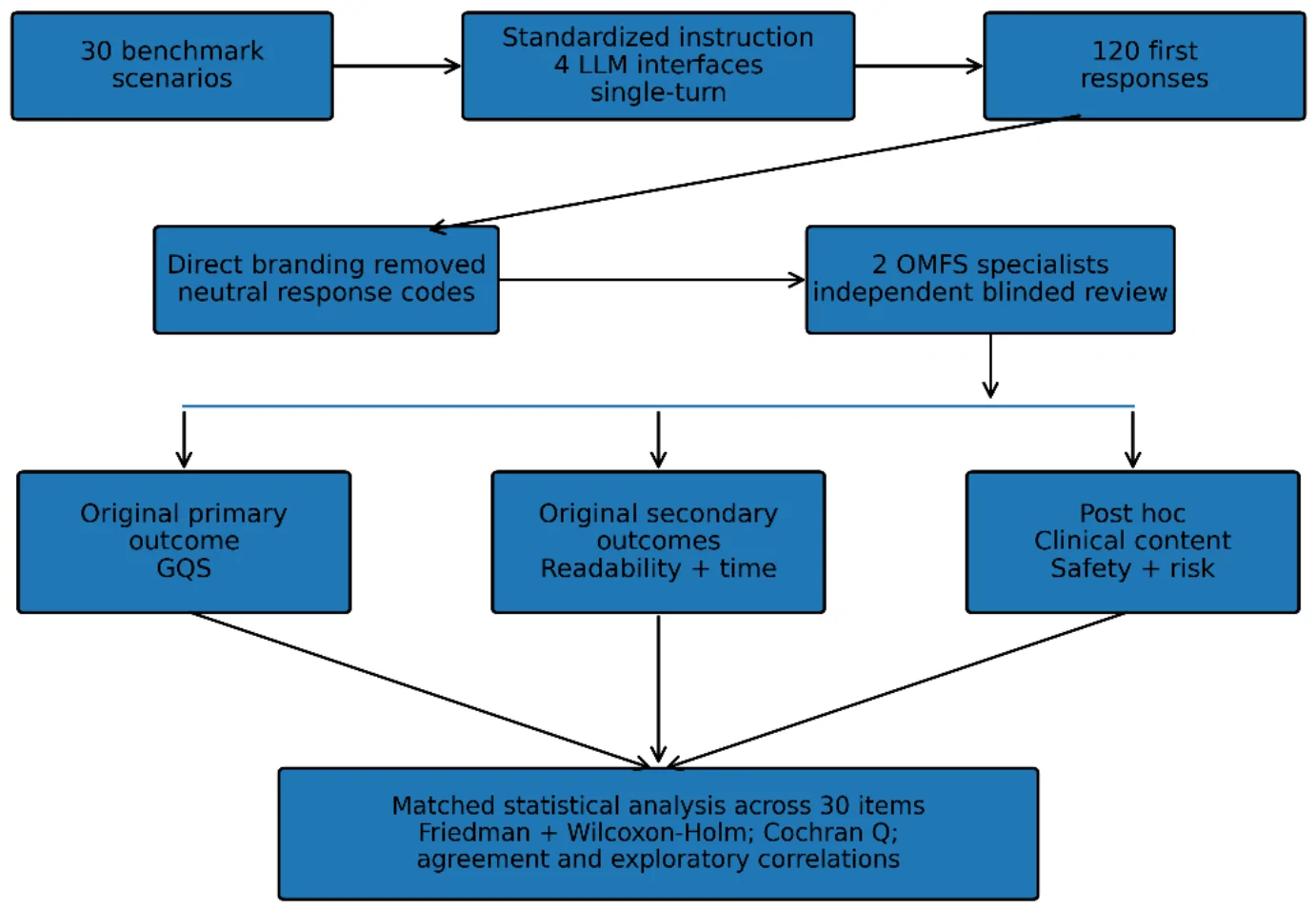
Study Workflow.

**Figure 2:**
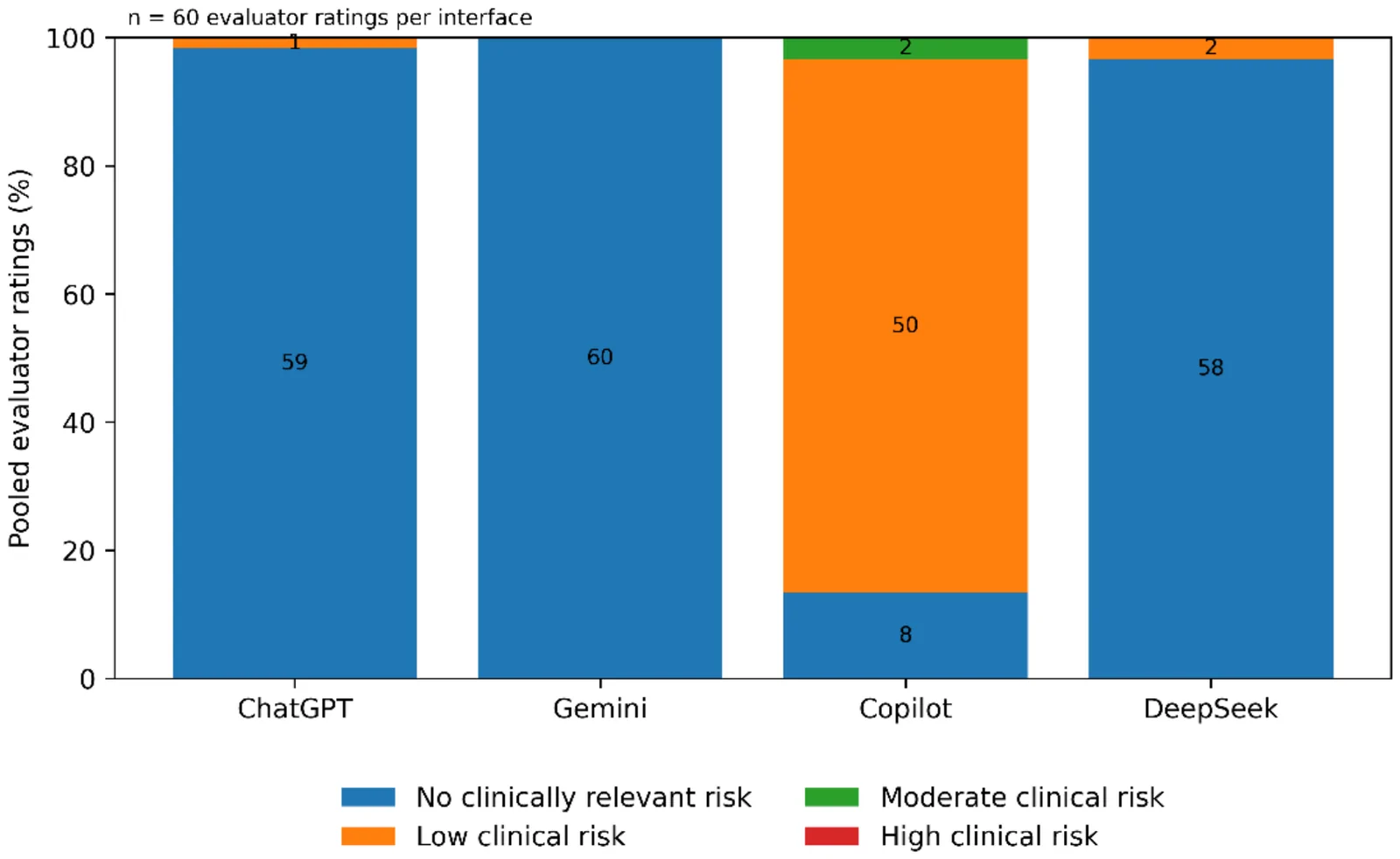
Distribution of study-specific potential clinical-risk classifications across the four evaluated interfaces. Bars represent pooled evaluator-level classifications (n = 60 ratings per interface). The four categories were developed specifically for this study and do not constitute an externally validated clinical safety scale. Categories represent potential clinical consequences in simulated benchmark scenarios rather than observed patient harm.

**Table 1:** Task-Adapted Global Quality Score for Specialist-Rated Response Quality.

Score	Quality Level	Description
1	Very poor	The response is of very low overall quality, contains substantial inaccuracies and/or clinically important omissions, is poorly structured, and provides little or no practical value for the intended specialist audience.
2	Poor	The response has limited overall quality, contains important missing information and/or inaccuracies, and provides only minimal practical value for the intended specialist audience.
3	Moderate	The response is generally acceptable but lacks important clinical details. It provides partially useful information, although completeness, clarity, and/or clinical utility are limited.
4	Good	The response is generally accurate, well organized, and clinically useful, with only minor deficiencies or omissions that do not substantially impair its overall value.
5	Excellent	The response is highly accurate, comprehensive, clearly structured, clinically coherent, and highly useful for the intended specialist audience.

Note: The five-point Global Quality Score (GQS) was adapted for specialist-level LLM-generated clinical responses from the global quality framework described by Bernard et al. [24]. In the present study, the GQS was used as a global specialist-rating instrument and was not independently validated as an objective measure of guideline concordance, clinical correctness, or safety. Higher scores indicate better overall specialist-rated response quality.

**Table 2:** Study-Specific Potential Clinical-Risk Classification Used for Safety-Oriented Evaluation of LLM Responses.

Score	Risk Category	Definition	Third-Molar/OMFS-Specific Interpretive Examples
0	No clinically relevant potential risk	The response is clinically appropriate and consistent with accepted oral and maxillofacial surgery knowledge, with no meaningful inaccuracy or omission expected to alter clinical management or patient safety.	Correct identification of the complication together with appropriate management, follow-up, referral, or emergency escalation when indicated.
1	Low clinical potential risk	A minor omission, imprecision, or incomplete explanation is present, but it is unlikely to alter clinical management or result in clinically meaningful patient harm.	Correct diagnosis and overall treatment pathway but limited detail regarding follow-up, supportive care, counseling, or non-critical adjunctive measures.
2	Moderate clinical potential risk	A clinically relevant inaccuracy or omission is present that could plausibly result in incomplete management, delayed referral, inappropriate monitoring, or suboptimal care, but is unlikely by itself to cause immediate severe harm.	Incomplete management of a nerve injury; inadequate follow-up after an oroantral communication; omission of clinically relevant anticoagulation assessment in postoperative bleeding; or insufficient recognition of when postoperative infection requires specialist review.
3	High clinical potential risk	A major omission or inaccurate recommendation is present that could plausibly result in serious patient harm if followed without specialist correction.	Failure to prioritize airway management in Ludwig’s angina or anaphylaxis; delayed emergency referral for a suspected descending deep-neck infection; inappropriate management of uncontrolled hemorrhage; unsafe invasive management of MRONJ or osteoradionecrosis; or failure to recognize aspiration or another immediately threatening complication.

Note: This four-level potential clinical-risk classification was developed de novo specifically for the present study as a post hoc safety-oriented framework and has not undergone independent external validation. CHART informed the systematic reporting and assessment of potentially harmful or misleading chatbot-generated health information [20], whereas WHO patient-safety terminology provided only broad conceptual context for considering the potential consequences of errors and omissions [25]. Neither source provides, endorses, or validates the four categories, score thresholds, or category definitions used here. Accordingly, this framework should not be interpreted as, or considered directly equivalent to, an established WHO or other validated clinical safety scale. The categories represent study-specific judgments of potential clinical risk in simulated benchmark scenarios rather than observed patient harm.

**Table 3:** Inter-Evaluator Agreement for the Original GQS, Post Hoc Criterion-Referenced Assessment, and Study-Specific Potential Clinical-Risk Classification.

Outcome	Agreement Measure	Result
GQS—ChatGPT	ICC (95% CI)	0.928 (0.790–1.000)
GQS—Gemini	ICC (95% CI)	0.930 (0.799–1.000)
GQS—Copilot	ICC (95% CI)	0.904 (0.717–1.000)
GQS—DeepSeek	ICC (95% CI)	0.916 (0.739–1.000)
Diagnosis/recognition	Exact agreement	100%; κ not estimable *
Management completeness	Exact agreement; weighted κ	94.2%; 0.826
Escalation/referral	Exact agreement; κ	100%; 1.000
Potentially harmful recommendation	Exact agreement; κ	100%; 1.000
Safety-critical omission	Exact agreement; κ	100%; 1.000
Study-specific potential clinical-risk classification	Exact agreement; weighted κ	99.2%; 0.979

Note: ICC = intraclass correlation coefficient; GQS = Global Quality Score; CI = confidence interval; κ = Cohen’s kappa. * κ was not estimable because both evaluators assigned all 120 responses to the same category despite 100% observed agreement. Quadratic-weighted Cohen’s κ was used for ordinal outcomes, including management completeness and the study-specific potential clinical-risk classification; unweighted Cohen’s κ was used for binary outcomes when sufficient category variability was present. Agreement statistics quantify consistency between the two evaluators under the scoring procedures used in this study and should not be interpreted as evidence of construct validity, criterion validity, or external validation of the GQS, criterion-referenced framework, or study-specific potential clinical-risk classification. High observed agreement may partly reflect the constrained nature of the rubric and limited category variability in some domains. κ was not estimable for diagnosis/recognition because both evaluators assigned all 120 responses to the same category despite 100% observed agreement. For potentially harmful recommendations, the re-audited dataset contained one positive response identified independently by both evaluators; therefore, κ was estimable (κ = 1.000), although interpretation is limited by the single positive event.

**Table 4:** Specialist-Rated Quality, Criterion-Referenced Clinical Outcomes, Study-Specific Potential Clinical Risk, and Externally Observable Response Latency Across the 30 Benchmark Items.

Outcome	ChatGPT	Gemini	Copilot	DeepSeek	Omnibus Test
GQS, mean ± SD (95% CI)	4.40 ± 0.48 (4.23–4.58)	4.53 ± 0.49 (4.37–4.70)	3.92 ± 0.51 (3.73–4.10)	4.30 ± 0.45 (4.15–4.47)	χ^2^ = 16.548; *p* < 0.001; W = 0.184
Clinical Content Score, mean ± SD (95% CI)	4.80 ± 0.41 (4.63–4.93)	4.77 ± 0.43 (4.60–4.90)	3.90 ± 0.59 (3.68–4.10)	4.75 ± 0.47 (4.58–4.90)	χ^2^ = 78.860; *p* < 0.001; W = 0.876
Clinical Content, % maximum	96.0%	95.3%	78.0%	95.0%	—
Management completeness, mean ± SD (95% CI)	3.00 ± 0.00 (3.00–3.00)	3.00 ± 0.00 (3.00–3.00)	2.17 ± 0.30 (2.07–2.28)	2.98 ± 0.09 (2.95–3.00)	χ^2^ = 82.579; *p* < 0.001; W = 0.918
Target-complication recognition, n/N (%, 95% CI)	30/30 (100%; 88.6–100.0)	30/30 (100%; 88.6–100.0)	30/30 (100%; 88.6–100.0)	30/30 (100%; 88.6–100.0)	No variability
Appropriate escalation/referral, n/N (%, 95% CI)	24/30 (80.0%; 62.7–90.5)	23/30 (76.7%; 59.1–88.2)	22/30 (73.3%; 55.6–85.8)	23/30 (76.7%; 59.1–88.2)	Q = 2.400; *p* = 0.494
Potentially harmful recommendation, n/N (%, 95% CI)	0/30 (0%; 0.0–11.4)	0/30 (0%; 0.0–11.4)	1/30 (3.3%; 0.6–16.7)	0/30 (0%; 0.0–11.4)	Descriptive; single event
Safety-critical omission, n/N (%, 95% CI)	0/30 (0%; 0.0–11.4)	0/30 (0%; 0.0–11.4)	2/30 (6.7%; 1.8–21.3)	0/30 (0%; 0.0–11.4)	Q = 6.000; *p* = 0.112
Study-specific potential clinical-risk score, mean ± SD (95% CI)	0.02 ± 0.09 (0.00–0.05)	0.00 ± 0.00 (0.00–0.00)	0.90 ± 0.40 (0.77–1.03)	0.03 ± 0.18 (0.00–0.10)	χ^2^ = 74.698; *p* < 0.001; W = 0.830
Externally observable response latency, s, mean ± SD (95% CI)	13.17 ± 7.46 (10.73–15.93)	15.87 ± 9.08 (13.33–19.50)	1.23 ± 1.87 (0.63–1.93)	6.80 ± 4.28 (5.47–8.47)	χ^2^ = 70.923; *p* < 0.001; W = 0.788
Externally observable response latency, median, s	10.00	14.50	0.00 *	6.00	—

Note: GQS = Global Quality Score; SD = standard deviation; CI = confidence interval; Q = Cochran’s Q; W = Kendall’s W. Clinical Content Score = diagnosis/recognition + management completeness + escalation/referral (range, 0–5). Values are presented as mean ± SD (95% CI) for continuous or ordinal-composite response-level outcomes and n/N (%, 95% CI) for binary outcomes. Confidence intervals for interface-specific means were obtained using 20,000 percentile-bootstrap resamples at the benchmark-item level. Confidence intervals for binary proportions were calculated using the Wilson method. The potential clinical-risk score was derived from a four-level study-specific classification developed de novo for this benchmark and should not be interpreted as an established or externally validated clinical safety scale. The categories represent study-specific judgments of potential clinical consequences in simulated scenarios rather than observed patient harm. The confidence intervals characterize the precision of the observed estimates within the present 30-item benchmark and should not be interpreted as estimates of stable performance across repeated generations or future interface versions. * A value of 0.00 s represents responses classified as “immediate,” defined as responses completed too rapidly for a distinct elapsed duration to be reliably captured using the manual stopwatch procedure; it does not indicate literal zero processing time. The primary externally observable response latency estimates, including the corresponding 95% confidence intervals, incorporate this lower-bound 0-s coding; the influence of these observations was evaluated separately in the exclusion-based sensitivity analysis.

**Table 5:** Paired Analysis of Readability Measures Across the 30 Benchmark Items.

Metric	ChatGPT Mean ± SD	Gemini Mean ± SD	Copilot Mean ± SD	DeepSeek Mean ± SD	Friedman χ^2^	*p*	Kendall W
FKGL	13.13 ± 2.41	10.89 ± 1.24	12.66 ± 0.98	11.63 ± 2.42	30.519	<0.001	0.339
GFI	16.63 ± 2.99	14.62 ± 1.59	16.20 ± 1.25	15.89 ± 3.05	17.909	<0.001	0.199
CLI	16.78 ± 1.70	14.98 ± 1.45	16.83 ± 1.16	14.85 ± 1.96	52.091	<0.001	0.579
SMOG	14.87 ± 1.96	13.31 ± 1.01	14.40 ± 0.76	14.14 ± 2.15	28.485	<0.001	0.316
FRES	36.29 ± 10.11	40.72 ± 8.25	34.56 ± 5.28	37.33 ± 12.17	15.640	0.001	0.174

Note: Lower FKGL, GFI, CLI, and SMOG values and higher FRES values indicate lower surface linguistic complexity/easier textual readability according to the respective formulas. In this specialist-level benchmark, these directions should not be interpreted as indicating superior clinical communication, clinical quality, or appropriateness for an OMFS specialist audience. The five indices share overlapping formula components and should be interpreted collectively as complementary indicators of surface linguistic complexity rather than as statistically independent outcomes.

## Data Availability

The complete benchmark specification and supporting analyses are provided with the article. Supplementary Table S1 contains the exact wording and diagnostic-cueing classification of all 30 benchmark scenarios, and Supplementary Table S2 contains the item-specific criterion-referenced clinical scoring framework. Supplementary Tables S3–S9 provide the pairwise comparisons, sensitivity analyses, item-level management-completeness results, evaluator-specific analyses, safety-critical-omission details, and readability comparisons. Supplementary Table S10 provides the post hoc category mapping and category-level descriptive performance analysis, and Supplementary Table S11 provides representative higher-performing and criterion-deficient response examples. The complete archived interface-generated response set is provided in Supplementary File S1. The complete response-level derived dataset and accompanying data dictionary are provided in Supplementary File S2 and include evaluator-specific and averaged GQS ratings, criterion-referenced domain scores, Clinical Content Scores, equal-domain-weight sensitivity scores, study-specific potential clinical-risk classifications, potentially harmful-recommendation and safety-critical-omission indicators, externally observable response latency measurements and immediate-response indicators, and all five readability measures. Response identifiers permit linkage of the derived data to the corresponding archived outputs. Together, these materials provide the item- and response-level data necessary to reproduce the principal reported analyses and independently audit the aggregate clinical-content and safety findings.

## Supplementary Materials

The following supporting information can be downloaded at: https://www.mdpi.com/article/10.3390/jcm15197397/s1, Table S1: Exact Wording and Diagnostic Cueing of the Standardized 30-Item Benchmark Scenario Set; Table S2: Item-Specific Criterion-Referenced Clinical Scoring Framework for the 30 Benchmark Scenarios; Table S3: Holm-Adjusted Pairwise Comparisons for Post Hoc Clinical Content, Management Completeness, and Clinical Risk; Table S4: Equal-Domain Weighting Sensitivity Analysis for the Clinical Content Score; Table S5: Item-Level Management-Completeness Scores Across the 30 Benchmark Scenarios; Table S6: Sensitivity Analysis of Externally Observable Response Latency After Exclusion of Benchmark Items Containing “Immediate” Responses; Table S7: Evaluator-Specific Sensitivity Analysis of Global Quality Scores; Table S8: Item-Level Description of the Safety-Critical Omissions Identified in Copilot Responses; Table S9: Holm-Adjusted Paired Wilcoxon Comparisons Across the Five Readability Indices; Table S10: Post Hoc Descriptive Distribution and Category-Level Performance Across Clinically Oriented Complication Categories; Table S11: Representative Higher-Performing and Criterion-Deficient Responses Illustrating Clinical Content Differences Across Interfaces; Supplementary File S1: Archived Interface-Generated Responses; Supplementary File S2: Quality Assurance Checks.

## Abbreviations

AI, artificial intelligence; CBCT, cone-beam computed tomography; CHART, Chatbot Assessment Reporting Tool; CI, confidence interval; CLI, Coleman–Liau Index; FKGL, Flesch–Kincaid Grade Level; FRES, Flesch Reading Ease Score; GFI, Gunning Fog Index; GQS, Global Quality Score; ICC, intraclass correlation coefficient; IE1, independent evaluator 1; IE2, independent evaluator 2; LLM, large language model; MRONJ, medication-related osteonecrosis of the jaw; OMFS, oral and maxillofacial surgery; SD, standard deviation; SMOG, Simple Measure of Gobbledygook; SPSS, Statistical Package for the Social Sciences; URL, Uniform Resource Locator; UTC, Coordinated Universal Time; WHO, World Health Organization.
