# Supplementary material for: Comparative Evaluation of Large Language Model Interfaces in Third Molar Surgery Complication Scenarios: Response Quality, Clinical Content, Potential Clinical Risk, Readability, and Externally Observable Response Latency—A Cross-Sectional Comparative Benchmark Study
Source: J Clin Med. 2026 Sep 23;15(19):7397. doi: 10.3390/jcm15197397 (PMC13666355; doi:10.3390/jcm15197397)
Supplement: Supplementary file 1 [file jcm-15-07397-s001.zip › JCM SUPPLEMENTARY TABLES.pdf]

Supplementary Tables

Table S1. Exact Wording and Diagnostic Cueing of the Standardized 30-Item Benchmark Scenario Set

Item	Standardized benchmark question	Diagnostic cueing / inferential demand	Basis for classification
**Q1**	In a 27-year-old patient, during surgical extraction of an impacted mandibular right third molar, an air-driven high-speed handpiece is used for bone removal. Shortly after the procedure, sudden swelling develops in the right cheek and submandibular region, with palpable crepitus on examination. What complication does this presentation suggest, by what mechanism does it occur, and how should it be managed?	Strongly cued	Air-driven handpiece use followed by sudden swelling and palpable crepitus provides a highly characteristic clinical pattern.
**Q2**	In a 25-year-old patient, during removal of a fractured apical root fragment after extraction of tooth 28, the fragment is displaced apically and separated from the socket. Radiographs show that the fragment has been displaced into the maxillary sinus. What clinical pathway should be followed in this situation?	Explicit/near-explicit	The radiographic finding explicitly states displacement of the root fragment into the maxillary sinus.
**Q3**	In a 29-year-old patient, during extraction of a deeply impacted tooth 48 that is related to the inferior alveolar nerve, a fractured apical root fragment is displaced apically and separated from the socket during attempted retrieval. Radiographs show that the fragment has been displaced into the inferior alveolar nerve canal. What clinical pathway should be followed, and what should the treatment approach be?	Explicit/near-explicit	The radiographic finding explicitly states displacement into the inferior alveolar nerve canal.
**Q4**	In a 28-year-old patient using oral contraceptives, severe pain and a foul odor develop a few days after extraction of tooth 38. Which postoperative complication is this most consistent with, and how should treatment be planned?	Strongly cued	Characteristic timing, severe postoperative pain, foul odor, and oral-contraceptive use strongly suggest the target complication.

**Q5**	In a 24-year-old patient, persistent numbness of the lower lip and chin region for three months following a difficult mandibular right 48 extraction suggests which neurological complication, and how should this condition be managed?	Strongly cued	Persistent lower-lip and chin sensory disturbance after mandibular third-molar surgery strongly localizes the neurological complication.
**Q6**	In a 26-year-old patient, following lingual retraction during extraction of tooth 38, numbness and loss of taste develop on the left half of the tongue. Which nerve injury does this suggest, and how should it be treated?	Strongly cued	Lingual retraction combined with ipsilateral tongue numbness and taste loss provides highly characteristic anatomical clues.
**Q7**	In a 26-year-old patient, a tooth is lost during extraction of tooth 28, and two days later CBCT shows the tooth in the infratemporal fossa. What does this finding indicate, and what is the appropriate treatment approach?	Explicit/near-explicit	CBCT explicitly identifies the tooth within the infratemporal fossa.
**Q8**	In a 34-year-old patient, a mandibular basal (inferior border) fracture occurs during extraction of a deeply impacted tooth 38 due to excessive elevator force. What should the emergency management approach be?	Explicit/near-explicit	The mandibular basal fracture is explicitly stated in the scenario.
**Q9**	In a 28-year-old patient, one week after extraction of tooth 48, progressively worsening severe pain, trismus, fever, and cervical lymphadenopathy develop. The patient also has dysphagia/odynophagia, diffuse neck swelling, and chest pain–shortness of breath, raising suspicion of spread of infection through deep neck spaces to the mediastinum. Which diagnosis does this presentation most strongly suggest?	Strongly cued	The clinical constellation and stated suspicion of deep-neck spread toward the mediastinum substantially narrow the diagnosis.
**Q10**	In a 25-year-old patient, six weeks after mandibular 48 extraction, the patient reports food impaction and bleeding distal to tooth 47. Clinical examination reveals a ≥6 mm periodontal pocket distal to 47 with bleeding on probing; radiographs show localized bone loss distal to 47. What	Strongly cued	A deep distal periodontal pocket, bleeding on probing, food impaction, and localized radiographic bone loss provide a characteristic periodontal pattern.

	surgical and postoperative approach should be implemented in this case?		
**Q11**	In a 29-year-old patient, recurrent bleeding from the extraction site begins approximately one week after extraction of mandibular left 38. What mechanism could explain this delayed bleeding, and how should it be managed?	Clinically inferential	The diagnosis/mechanism must be inferred primarily from the delayed timing and postoperative context rather than from an explicitly named complication.
**Q12**	In a 22-year-old patient, soft tissue trauma–related tissue hemorrhage occurs during local anesthetic injection prior to mandibular 38 extraction. How would you proceed with the procedure in this situation?	Strongly cued	Hemorrhage during local-anesthetic injection provides a highly specific procedural context for the expected complication and management pathway.
**Q13**	A 25-year-old patient presents two days after mandibular 48 extraction with limited mouth opening and difficulty eating. How should the patient be assessed and managed?	Clinically inferential	Limited mouth opening after surgery requires differentiation between expected postoperative trismus and more serious infectious causes.
**Q14**	In a 55-year-old patient with a history of four doses of intravenous bisphosphonate (zoledronic acid) use, an unhealed area of exposed bone and pain develop weeks after extraction of mandibular 38. Which complication is most likely, and how should management be planned?	Strongly cued	Intravenous zoledronic-acid exposure together with persistent exposed bone provides a highly characteristic risk-factor–presentation combination.
**Q15**	In a 55-year-old patient with a history of radiotherapy, an unhealed area of exposed bone and pain develop weeks after extraction of tooth 38. Which complication is this associated with, and how should it be managed?	Strongly cued	Previous radiotherapy combined with persistent exposed bone strongly indicates the intended complication.
**Q16**	In a 62-year-old patient taking warfarin, a mandibular right 48 extraction is performed without checking INR, followed by uncontrolled bleeding. What mechanism explains this bleeding, and what treatment should be applied?	Strongly cued	Warfarin use, absent INR assessment, and uncontrolled postoperative bleeding strongly define the anticoagulation-related problem.
**Q17**	In a 30-year-old patient, after mandibular 48 extraction, painful oral erosions and cutaneous target lesions develop following use of ibuprofen and amoxicillin. Which	Strongly cued	Target lesions and oral erosions following medication exposure provide

	drug reaction does this suggest, and how should it be treated?		highly characteristic diagnostic cues.
**Q18**	In a 27-year-old patient, during surgical extraction of an impacted tooth 28, a portion of the maxillary tuberosity fractures and separates along with the tooth. What surgical and postoperative management should be performed?	Explicit/near-explicit	Maxillary tuberosity fracture is explicitly described.
**Q19**	In a 29-year-old patient, several weeks after extraction of tooth 18, unilateral nasal obstruction, foul-smelling yellow-green discharge, and facial pain develop. Which complication does this suggest, and how should it be managed?	Clinically inferential	Post-extraction sinonasal symptoms require integration of timing and clinical findings to infer an oroantral pathway with associated sinus disease.
**Q20**	In a 24-year-old patient, during difficult extraction of a deeply impacted 48, the tooth slips from the elevator and is lost intraorally, followed by coughing and a foreign-body sensation in the throat. What condition does this most strongly suggest, and how should it be managed?	Strongly cued	Loss of the tooth followed immediately by coughing and foreign-body sensation strongly indicates aspiration/ingestion risk.
**Q21**	In a 27-year-old patient, during an inferior alveolar nerve block for mandibular right 48, the needle fractures and is lost within the pterygomandibular space. By what mechanism can this occur, and how should it be treated?	Explicit/near-explicit	Needle fracture and its anatomical location are explicitly stated.
**Q22**	In a 19-year-old patient, during impacted 48 surgery, cyanosis, fatigue, and low oxygen saturation develop after administration of prilocaine and articaine. What does this indicate, and how should it be treated?	Strongly cued	Cyanosis and low oxygen saturation after administration of implicated local anesthetics provide a characteristic pharmacological clue.
**Q23**	In a 45-year-old patient with uncontrolled diabetes, increasing pain, fistula formation, foul odor, and sequestrum formation develop a few weeks after extraction of mandibular left 37 and 38. What condition does this suggest, and how should it be treated?	Strongly cued	Uncontrolled diabetes combined with fistula, foul odor, pain, and sequestrum formation provides a characteristic pattern.
**Q24**	In a 23-year-old patient, after local anesthesia for tooth 28, it is learned that the patient has a history of drug allergy	Strongly cued	Rapid-onset multisystem allergic manifestations after

	and atopic disease. Shortly after injection, generalized pruritus–urticaria, lip/face swelling, wheezing, and dizziness develop. How would you evaluate this presentation in terms of the most likely diagnosis, and how would you plan the emergency management in a dental clinic?		exposure provide a highly characteristic presentation.
**Q25**	A 29-year-old patient presents two weeks after extraction of tooth 18 with the complaint that liquids consumed come out through the nose. CBCT shows an oroantral communication measuring 6 mm. What complication does this represent, and how should it be treated?	Explicit/near-explicit	CBCT explicitly reports a 6-mm oroantral communication.
**Q26**	In a 52-year-old patient with uncontrolled diabetes, five days after bilateral mandibular 38 and 48 extractions, tongue swelling, dyspnea, and "board-like" induration/swelling of the neck develop. Which life-threatening infection does this suggest, and what is the appropriate emergency management?	Strongly cued	Tongue swelling, dyspnea, and board-like neck induration form a highly characteristic emergency presentation.
**Q27**	In a 22-year-old patient, after an inferior alveolar nerve (IAN) block administered for mandibular right 48, difficulty closing the eye and drooping of the corner of the mouth are observed. How can the cause of this finding be explained, and how should it be managed?	Strongly cued	Acute facial weakness immediately after an IAN block provides a characteristic procedural-anatomical clue.
**Q28**	In a 27-year-old patient, within a few days after a difficult 28 extraction, temporomandibular joint clicking, locking, and pain develop. By what mechanism can this be explained, and how should it be managed?	Strongly cued	TMJ clicking, locking, and pain after a difficult extraction strongly identify the affected anatomical system and likely mechanism.
**Q29**	In a 28-year-old patient, during mandibular left 38 surgery, prolonged use of a bur with inadequate water cooling results in a localized area of necrotic bone. Which complication is this consistent with, and how should it be managed?	Strongly cued	Prolonged burring, inadequate cooling, and localized necrotic bone provide a highly specific causal pattern.
**Q30**	In a 23-year-old patient, intraosseous anesthesia is administered during	Clinically inferential	Timing, intraosseous anesthesia, persistent

	mandibular 38 extraction. Ten days later, the patient presents with an unhealed area of exposed bone at the extraction site and increasing pain. Which complication is this most likely to indicate, and how should management be planned?	exposed bone, and increasing pain require differentiation from dry socket, osteomyelitis, and other causes of delayed healing.

Note: Each benchmark item was preceded by the standardized instruction: "Please answer the following question at a specialist level in oral and maxillofacial surgery, in a manner consistent with accepted scientific literature, established textbook knowledge, and sound clinical decision-making principles." Diagnostic cueing/inferential demand was categorized descriptively as explicit/near-explicit, strongly cued, or clinically inferential. Explicit/near-explicit scenarios directly stated the target complication or anatomical event; strongly cued scenarios contained a characteristic clinical constellation or highly specific precipitating factor that substantially narrowed the differential diagnosis; and clinically inferential scenarios required comparatively greater integration of timing, symptoms, risk factors, or differential diagnoses. This classification was introduced solely to characterize the diagnostic demands of the benchmark and was not used for scoring or inferential statistical analysis.

Table S2. Item-Specific Criterion-Referenced Clinical Scoring Framework for the 30 Benchmark Scenarios

Item / scenario	Criterion-referenced scoring criteria
**Q1**<br>Subcutaneous Emphysema (High-speed handpiece)	**A. Diagnosis/recognition:** Iatrogenic subcutaneous emphysema.<br>**B. Essential management elements:** 1) Immediate cessation of the procedure, 2) Airway and vital sign assessment, 3) Prophylactic broad-spectrum antibiotics.<br>**C. Appropriate escalation/referral:** Imaging (radiography/CT) to rule out pneumomediastinum, or hospital observation if severe.<br>**D. Potentially harmful/contraindicated recommendation:** Recommending surgical incision/drainage or positive pressure ventilation.<br>**E. Safety-critical omission:** Failing to assess the airway or omitting the risk of mediastinitis/pneumomediastinum.
**Q2**<br>Root displacement into the Maxillary Sinus	**A. Diagnosis/recognition:** Displacement of root fragment into the maxillary sinus.<br>**B. Essential management elements:** 1) Caldwell-Luc, lateral window, or endoscopic retrieval (or wait-and-see with radiographic follow-up for very small/uninfected fragments), 2) Sinus precautions (no nose-blowing), 3) Prophylactic antibiotics and decongestants.<br>**C. Appropriate escalation/referral:** Referral to a specialist/ENT if retrieval is beyond the operator's capability.<br>**D. Potentially harmful/contraindicated recommendation:** Blind, aggressive curettage through the extraction socket.<br>**E. Safety-critical omission:** Failing to prescribe sinus precautions.

Item / scenario	Criterion-referenced scoring criteria
**Q3**<br>Root displacement into the Inferior Alveolar Canal	**A. Diagnosis/recognition:** Displacement of root into the inferior alveolar nerve (IAN) canal.<br>**B. Essential management elements:** 1) CBCT imaging for 3D localization, 2) Assessment of neurological status (numbness/dysethesia), 3) Risk-benefit analysis (leave small asymptomatic fragments vs. surgical retrieval).<br>**C. Appropriate escalation/referral:** Referral to a microneurosurgery/OMFS specialist for retrieval.<br>**D. Potentially harmful/contraindicated recommendation:** Blind probing or forceful instrumentation directly into the canal.<br>**E. Safety-critical omission:** Failing to require a CBCT before further intervention.
**Q4**<br>Severe pain/foul odor + Oral contraceptives (Dry Socket)	**A. Diagnosis/recognition:** Alveolar osteitis (Dry socket).<br>**B. Essential management elements:** 1) Gentle saline irrigation, 2) Placement of a medicated dressing (e.g., Alvogyl or ZOE), 3) Analgesics.<br>**C. Appropriate escalation/referral:** Re-evaluation if symptoms persist to rule out osteomyelitis.<br>**D. Potentially harmful/contraindicated recommendation:** Aggressive socket curettage to induce bleeding.<br>**E. Safety-critical omission:** Failing to mention that the condition is self-limiting or failing to rule out true infection.
**Q5**<br>Persistent numbness (3 months post-op)	**A. Diagnosis/recognition:** IAN paresthesia/neuropathy/injury.<br>**B. Essential management elements:** 1) Clinical neurosensory testing (mapping), 2) Documentation of the deficit, 3) Consideration for microneurosurgical intervention due to the 3-month window.<br>**C. Appropriate escalation/referral:** Immediate referral to a specialist in trigeminal nerve repair.<br>**D. Potentially harmful/contraindicated recommendation:** Advising the patient to "wait another 6-12 months" without testing (misses the surgical window).<br>**E. Safety-critical omission:** Omitting the time-sensitive nature of nerve repair (optimal within 3-6 months).
**Q6**<br>Numbness and loss of taste on the tongue	**A. Diagnosis/recognition:** Lingual nerve injury.<br>**B. Essential management elements:** 1) Neurosensory testing (including taste), 2) Short-term systemic corticosteroids (if acute), 3) Regular follow-up for spontaneous recovery vs. surgical repair.<br>**C. Appropriate escalation/referral:** Referral for microneurosurgery if no improvement in 1-3 months.<br>**D. Potentially harmful/contraindicated recommendation:** Disregarding the symptom as "normal and always temporary."<br>**E. Safety-critical omission:** Failing to note that lingual nerve injuries have a lower rate of spontaneous recovery compared to IAN.

Item / scenario	Criterion-referenced scoring criteria
**Q7**<br>Tooth in Infratemporal Fossa	**A. Diagnosis/recognition:** Displacement of tooth into the infratemporal fossa (ITF).<br>**B. Essential management elements:** 1) Initial conservative management (antibiotics, observe, allow fibrosis) OR immediate surgical retrieval depending on accessibility, 2) CBCT for precise localization, 3) Prevention of infection.<br>**C. Appropriate escalation/referral:** Referral to an experienced OMFS for surgical retrieval via coronal or intraoral approach.<br>**D. Potentially harmful/contraindicated recommendation:** Blind, aggressive probing in the ITF (risk of bleeding from pterygoid plexus or further displacement).<br>**E. Safety-critical omission:** Omitting the need for advanced 3D imaging (CBCT/CT).
**Q8**<br>Mandibular basal fracture	**A. Diagnosis/recognition:** Iatrogenic mandibular fracture.<br>**B. Essential management elements:** 1) Immediate stabilization, 2) Maxillomandibular fixation (MMF) or Open reduction and internal fixation (ORIF), 3) Antibiotics and soft/liquid diet.<br>**C. Appropriate escalation/referral:** ER or hospital admission for definitive fixation.<br>**D. Potentially harmful/contraindicated recommendation:** Discharging the patient with only pain medication and no stabilization.<br>**E. Safety-critical omission:** Failing to assess patient airway or occlusion.
**Q9**<br>Progressive severe pain, neck swelling, dysphagia, chest pain	**A. Diagnosis/recognition:** Descending necrotizing mediastinitis / Severe deep neck space infection (Ludwig's angina progression).<br>**B. Essential management elements:** 1) Immediate airway management, 2) Empirical broad-spectrum IV antibiotics, 3) Emergency surgical drainage (neck/chest).<br>**C. Appropriate escalation/referral:** Immediate transfer to the Emergency Department / ICU / Thoracic surgery consultation.<br>**D. Potentially harmful/contraindicated recommendation:** Prescribing oral antibiotics and sending the patient home.<br>**E. Safety-critical omission:** Failing to recognize the life-threatening nature (airway compromise / mediastinitis).
**Q10**<br>Periodontal pocket distal to 47	**A. Diagnosis/recognition:** Periodontal defect / bone loss distal to the second molar post-extraction.<br>**B. Essential management elements:** 1) Plaque control / OHI, 2) Scaling and root planing (SRP), 3) Consideration for guided tissue regeneration (GTR) or bone grafting.<br>**C. Appropriate escalation/referral:** Referral to a periodontist if conservative measures fail.<br>**D. Potentially harmful/contraindicated recommendation:** Immediate extraction of the healthy 47.<br>**E. Safety-critical omission:** Ignoring the active infection/bleeding and offering no periodontal intervention.

Item / scenario	Criterion-referenced scoring criteria
**Q11**<br>Recurrent bleeding (one week post-op)	**A. Diagnosis/recognition:** Secondary hemorrhage.<br>**B. Essential management elements:** 1) Local anesthesia and removal of the "liver clot", 2) Identification of the bleeding source and local hemostasis (suturing, hemostatic agents), 3) Prescription of antibiotics (as secondary bleeding is often infection-driven).<br>**C. Appropriate escalation/referral:** Systemic coagulopathy workup if local measures fail.<br>**D. Potentially harmful/contraindicated recommendation:** Simply prescribing oral tranexamic acid without debriding the infected clot.<br>**E. Safety-critical omission:** Failing to recognize infection as the primary cause of secondary hemorrhage.
**Q12**<br>Hemorrhage during LA injection	**A. Diagnosis/recognition:** Pterygomandibular hematoma.<br>**B. Essential management elements:** 1) Stop the procedure immediately, 2) Apply direct pressure and cold compress, 3) Delay the extraction to another appointment.<br>**C. Appropriate escalation/referral:** Observation for airway compromise if the hematoma expands medially.<br>**D. Potentially harmful/contraindicated recommendation:** Continuing the surgical extraction through a massive expanding hematoma.<br>**E. Safety-critical omission:** Not warning the patient about subsequent trismus and facial bruising.
**Q13**<br>Trismus and limited opening 2 days post-op	**A. Diagnosis/recognition:** Post-operative trismus (muscle spasm/inflammation).<br>**B. Essential management elements:** 1) Rule out deep space infection, 2) Warm compresses and physiotherapy/stretching, 3) NSAIDs/Muscle relaxants.<br>**C. Appropriate escalation/referral:** Follow-up to ensure it doesn't progress to a fascial space infection.<br>**D. Potentially harmful/contraindicated recommendation:** Forceful jaw manipulation under general anesthesia in the acute inflammatory phase.<br>**E. Safety-critical omission:** Failing to differentiate between normal post-op trismus and trismus caused by a severe masticator space infection.
**Q14**<br>Unhealed bone with Zoledronic Acid	**A. Diagnosis/recognition:** Medication-related osteonecrosis of the jaw (MRONJ).<br>**B. Essential management elements:** 1) Antimicrobial rinses (Chlorhexidine), 2) Systemic antibiotics if infected, 3) Conservative debridement / removal of loose sequestra.<br>**C. Appropriate escalation/referral:** Medical oncology consult / OMFS referral for staging and management.<br>**D. Potentially harmful/contraindicated recommendation:** Aggressive block resection for early-stage MRONJ without attempting conservative therapy.<br>**E. Safety-critical omission:** Failing to link the necrosis directly to the bisphosphonate history.

Item / scenario	Criterion-referenced scoring criteria
**Q15**<br>Unhealed bone with history of Radiotherapy	**A. Diagnosis/recognition:** Osteoradionecrosis (ORN).<br>**B. Essential management elements:** 1) Conservative debridement/irrigation, 2) Hyperbaric oxygen therapy (HBO) consideration (Marx protocol), 3) Long-term antibiotics/Pentoxifylline-Tocopherol (PENTOCLO) protocol.<br>**C. Appropriate escalation/referral:** Referral to specialized OMFS/Head and Neck center.<br>**D. Potentially harmful/contraindicated recommendation:** Aggressive immediate surgical resection in a primary dental clinic setting.<br>**E. Safety-critical omission:** Failing to recognize the condition as ORN.
**Q16**<br>Uncontrolled bleeding on Warfarin	**A. Diagnosis/recognition:** Coagulopathy-induced primary hemorrhage.<br>**B. Essential management elements:** 1) Immediate local hemostatic measures (gelatin sponge, oxidized cellulose, suturing), 2) Tranexamic acid mouthwash/pressure, 3) Medical consultation to check INR.<br>**C. Appropriate escalation/referral:** ER referral if local measures fail to control bleeding.<br>**D. Potentially harmful/contraindicated recommendation:** Advising the dentist/patient to stop Warfarin immediately without physician consultation.<br>**E. Safety-critical omission:** Failing to utilize local hemostatic agents.
**Q17**<br>Oral erosions and target lesions (Amoxicillin/Ibuprofen)	**A. Diagnosis/recognition:** Erythema Multiforme (EM) or Stevens-Johnson Syndrome (SJS).<br>**B. Essential management elements:** 1) Immediately discontinue the offending drugs, 2) Systemic corticosteroids (if EM) and supportive care, 3) Hydration and pain control.<br>**C. Appropriate escalation/referral:** Immediate referral to ER or Dermatology (due to SJS risk).<br>**D. Potentially harmful/contraindicated recommendation:** Switching blindly to another antibiotic class without addressing the immune reaction.<br>**E. Safety-critical omission:** Failing to recognize the potentially fatal nature of SJS.
**Q18**<br>Maxillary tuberosity fracture	**A. Diagnosis/recognition:** Maxillary tuberosity fracture.<br>**B. Essential management elements:** 1) If small, remove with the tooth. If large/attached to periosteum, abort extraction and splint, OR surgically dissect the tooth from the bone segment. 2) Ensure primary closure, 3) Sinus precautions.<br>**C. Appropriate escalation/referral:** Follow-up for OAC management.<br>**D. Potentially harmful/contraindicated recommendation:** Forcefully avulsing a large tuberosity segment attached to the palatal mucosa.<br>**E. Safety-critical omission:** Failing to assess for an Oroantral Communication (OAC) or palatal tissue tearing.

Item / scenario	Criterion-referenced scoring criteria
**Q19**<br>Unilateral nasal obstruction, foul discharge post-extraction	**A. Diagnosis/recognition:** Oroantral fistula (OAF) with maxillary sinusitis.<br>**B. Essential management elements:** 1) Eradicate sinus infection first (antibiotics, decongestants), 2) Sinus irrigation, 3) Subsequent surgical closure of the fistula (e.g., buccal fat pad, buccal advancement flap).<br>**C. Appropriate escalation/referral:** Referral to OMFS or ENT if sinus clearance is required (FESS).<br>**D. Potentially harmful/contraindicated recommendation:** Attempting surgical closure immediately in the presence of acute purulent sinusitis.<br>**E. Safety-critical omission:** Missing the requirement to clear the sinus infection prior to closure.
**Q20**<br>Lost tooth, coughing, foreign-body sensation	**A. Diagnosis/recognition:** Aspiration or ingestion of the tooth.<br>**B. Essential management elements:** 1) Immediate Basic Life Support (BLS) if airway is obstructed, 2) Transport to the ER, 3) Chest and abdominal radiographs to locate the tooth.<br>**C. Appropriate escalation/referral:** Referral for bronchoscopy (if aspirated) or endoscopy/observation (if ingested).<br>**D. Potentially harmful/contraindicated recommendation:** Sending the patient home without imaging, assuming they just swallowed it.<br>**E. Safety-critical omission:** Failing to secure/assess the airway immediately.
**Q21**<br>Broken needle in pterygomandibular space	**A. Diagnosis/recognition:** Needle fracture.<br>**B. Essential management elements:** 1) Instruct the patient to keep their mouth open (prevent muscle action from pushing it deeper), 2) Attempt retrieval with hemostat ONLY if the fragment is visible, 3) If not visible, do not probe blindly; take CBCT/fluoroscopy.<br>**C. Appropriate escalation/referral:** Urgent referral to an OMFS specialist for surgical retrieval under imaging guidance.<br>**D. Potentially harmful/contraindicated recommendation:** Blind incision and probing in the pterygomandibular space.<br>**E. Safety-critical omission:** Failing to instruct the patient to refrain from closing their mouth.
**Q22**<br>Cyanosis, low O2 sat after Prilocaine/Articaine	**A. Diagnosis/recognition:** Methemoglobinemia.<br>**B. Essential management elements:** 1) Administer 100% oxygen, 2) Administer IV Methylene Blue (antidote), 3) Monitor vitals.<br>**C. Appropriate escalation/referral:** Immediate EMS activation / ER transfer.<br>**D. Potentially harmful/contraindicated recommendation:** Administering more local anesthetic or ignoring the cyanosis.<br>**E. Safety-critical omission:** Failing to specifically mention IV Methylene Blue.

Item / scenario	Criterion-referenced scoring criteria
**Q23**<br>Fistula, sequestrum, uncontrolled diabetes	**A. Diagnosis/recognition:** Suppurative osteomyelitis (exacerbated by uncontrolled diabetes).<br>**B. Essential management elements:** 1) Culture and sensitivity testing, 2) Long-term culture-directed antibiotics, 3) Surgical debridement/sequestrectomy and strict glycemic control.<br>**C. Appropriate escalation/referral:** Referral to an endocrinologist for diabetes management alongside OMFS.<br>**D. Potentially harmful/contraindicated recommendation:** Prescribing a standard 5-day course of amoxicillin and discharging the patient.<br>**E. Safety-critical omission:** Ignoring the critical need for glycemic control to achieve healing.
**Q24**<br>Pruritus, wheezing, lip swelling, dizziness	**A. Diagnosis/recognition:** Anaphylaxis / Anaphylactic shock.<br>**B. Essential management elements:** 1) Immediate administration of IM Epinephrine (1:1000), 2) Call emergency medical services (EMS), 3) Oxygen administration and supine positioning.<br>**C. Appropriate escalation/referral:** Hospitalization for observation (biphasic reaction risk).<br>**D. Potentially harmful/contraindicated recommendation:** Relying solely on oral antihistamines for a patient with wheezing/dizziness.<br>**E. Safety-critical omission:** Failing to recommend IM Epinephrine as the first-line treatment.
**Q25**<br>Liquids through nose, 6mm OAC	**A. Diagnosis/recognition:** Oroantral communication (OAC).<br>**B. Essential management elements:** 1) Surgical closure is required (defects >5mm rarely heal spontaneously), 2) Flap choice (Buccal advancement flap or Buccal fat pad), 3) Sinus precautions and antibiotics/decongestants.<br>**C. Appropriate escalation/referral:** Referral to OMFS if the general dentist cannot perform the flap.<br>**D. Potentially harmful/contraindicated recommendation:** Expecting a 6mm defect to heal with just a figure-of-eight suture and blood clot.<br>**E. Safety-critical omission:** Failing to prescribe sinus precautions.
**Q26**<br>Tongue swelling, dyspnea, board-like neck in Diabetic	**A. Diagnosis/recognition:** Ludwig's Angina.<br>**B. Essential management elements:** 1) Immediate airway security (intubation/tracheostomy may be needed), 2) Emergency surgical decompression/drainage, 3) Aggressive IV broad-spectrum antibiotics.<br>**C. Appropriate escalation/referral:** Immediate EMS transfer to the ER/ICU.<br>**D. Potentially harmful/contraindicated recommendation:** Attempting outpatient incision and drainage in the dental chair.<br>**E. Safety-critical omission:** Failing to prioritize the airway as the immediate life-threatening concern.

**Item / scenario**	**Criterion-referenced scoring criteria**
**Q27**<br>Drooping corner of mouth, eye closure difficulty post IANB	**A. Diagnosis/recognition:** Transient facial nerve (CN VII) paralysis (LA injected into parotid gland).<br>**B. Essential management elements:** 1) Reassure the patient (temporary condition), 2) Protect the eye (eye patch, artificial tears) to prevent corneal abrasion, 3) Wait for the anesthetic to wear off.<br>**C. Appropriate escalation/referral:** Follow-up after a few hours to confirm resolution.<br>**D. Potentially harmful/contraindicated recommendation:** Recommending surgical exploration of the facial nerve.<br>**E. Safety-critical omission:** Failing to advise eye protection, risking corneal damage.
**Q28**<br>TMJ clicking, locking, pain post-extraction	**A. Diagnosis/recognition:** TMJ trauma / Disc displacement / Sprain due to excessive mouth opening forces.<br>**B. Essential management elements:** 1) Conservative therapy (Soft diet, rest), 2) NSAIDs and warm compresses, 3) Physiotherapy / Occlusal splint consideration.<br>**C. Appropriate escalation/referral:** Referral to a TMJ specialist if locking persists.<br>**D. Potentially harmful/contraindicated recommendation:** Forceful manipulation or immediate TMJ surgery.<br>**E. Safety-critical omission:** Failing to recommend a soft diet and joint rest.
**Q29**<br>Necrotic bone from inadequate water cooling	**A. Diagnosis/recognition:** Thermal osteonecrosis.<br>**B. Essential management elements:** 1) Conservative surgical debridement of necrotic bone, 2) Copious irrigation, 3) Antibiotics if secondary infection is present.<br>**C. Appropriate escalation/referral:** Observation and follow-up until mucosal coverage is achieved.<br>**D. Potentially harmful/contraindicated recommendation:** Extensive block resection for localized thermal necrosis.<br>**E. Safety-critical omission:** Failing to identify the iatrogenic thermal cause.
**Q30**<br>Unhealed bone post Intraosseous Anesthesia	**A. Diagnosis/recognition:** Avascular necrosis / Osteonecrosis due to intraosseous injection (vasoconstrictor ischemia).<br>**B. Essential management elements:** 1) Conservative management (analgesics, antimicrobial rinses), 2) Wait for the necrotic bone (sequestrum) to demarcate and separate, 3) Gentle removal of the loose sequestrum.<br>**C. Appropriate escalation/referral:** Referral to OMFS if defect becomes extensive.<br>**D. Potentially harmful/contraindicated recommendation:** Immediate aggressive bone drilling or resection of attached bone.<br>**E. Safety-critical omission:** Failing to link the localized necrosis to the intraosseous delivery technique.

Note: Scoring used in the post hoc assessment: A, diagnosis/recognition (0 = incorrect, 1 = correct); B, management completeness (0–3); C, escalation/referral (0 = absent/incorrect, 1 = appropriate); D, potentially harmful recommendation (0 = absent, 1 = present); E, safety-critical omission (0 = absent, 1 = present). The Clinical Content Score was calculated as A + B + C (range, 0–5). This framework was developed specifically for the present study and has not undergone independent external validation.

Table S3. Holm-Adjusted Pairwise Comparisons for Post Hoc Clinical Content, Management Completeness, and Clinical Risk

Outcome	Comparison	Holm-adjusted p	r_rb	Mean paired difference (95% bootstrap CI)
Clinical Content Score	ChatGPT vs Gemini	0.635	1.000	0.03 (0.00 to 0.10)
Clinical Content Score	ChatGPT vs Copilot	<0.001	1.000	0.90 (0.77 to 1.03)
Clinical Content Score	ChatGPT vs DeepSeek	0.539	1.000	0.05 (0.00 to 0.13)
Clinical Content Score	Gemini vs Copilot	<0.001	1.000	0.87 (0.72 to 1.02)
Clinical Content Score	Gemini vs DeepSeek	0.635	1.000	0.02 (0.00 to 0.05)
Clinical Content Score	Copilot vs DeepSeek	<0.001	-1.000	-0.85 (-1.00 to -0.70)
Management completeness	ChatGPT vs Gemini	1.000	Not estimable*	0.00 (0.00 to 0.00)
Management completeness	ChatGPT vs Copilot	<0.001	1.000	0.83 (0.72 to 0.93)
Management completeness	ChatGPT vs DeepSeek	0.952	1.000	0.02 (0.00 to 0.05)
Management completeness	Gemini vs Copilot	<0.001	1.000	0.83 (0.72 to 0.93)
Management completeness	Gemini vs DeepSeek	0.952	1.000	0.02 (0.00 to 0.05)
Management completeness	Copilot vs DeepSeek	<0.001	-1.000	-0.82 (-0.92 to -0.70)
Study-Specific Potential Clinical-Risk Score	ChatGPT vs Gemini	0.952	1.000	0.02 (0.00 to 0.05)
Study-Specific Potential Clinical-Risk Score	ChatGPT vs Copilot	<0.001	-1.000	-0.88 (-1.02 to -0.73)
Study-Specific Potential Clinical-Risk Score	ChatGPT vs DeepSeek	0.952	-1.000	-0.02 (-0.05 to 0.00)

Outcome	Comparison	Holm-adjusted p	r_rb	Mean paired difference (95% bootstrap CI)
Study-Specific Potential Clinical-Risk Score	Gemini vs Copilot	<0.001	-1.000	-0.90 (-1.03 to -0.77)
Study-Specific Potential Clinical-Risk Score	Gemini vs DeepSeek	0.952	-1.000	-0.03 (-0.10 to 0.00)
Study-Specific Potential Clinical-Risk Score	Copilot vs DeepSeek	<0.001	1.000	0.87 (0.70 to 1.00)

Note: Pairwise comparisons were performed using two-sided Wilcoxon signed-rank tests after a statistically significant Friedman omnibus test. p values were adjusted across the six pairwise contrasts within each outcome using the Holm method. r_rb = rank-biserial correlation. Mean paired differences were calculated as the first interface minus the second. Percentile-bootstrap 95% confidence intervals were based on 20,000 paired resamples. *r_rb was not estimable when all paired differences were zero. Rank-biserial correlations of ±1.000 may occur when all non-zero paired differences are in the same direction, even when most matched pairs are tied and the absolute mean paired difference is small. Accordingly, these values should be interpreted together with the number and magnitude of non-zero differences, the mean paired difference and its 95% confidence interval, and the Holm-adjusted p value; an r_rb of ±1.000 should not be interpreted as indicating large separation across all 30 matched items.

Abbreviations: CI, confidence interval; GQS, Global Quality Score; LLM, large language model; OMFS, oral and maxillofacial surgery.

Table S4. Equal-Domain Weighting Sensitivity Analysis for the Clinical Content Score

Panel A. Interface-level summary

Interface	Equal-Weight Clinical Content Score, Mean ± SD	Median (IQR)
**ChatGPT**	2.80 ± 0.41	3.00 (3.00–3.00)
**Gemini**	2.77 ± 0.43	3.00 (3.00–3.00)
**Copilot**	2.46 ± 0.48	2.67 (2.04–2.67)
**DeepSeek**	2.76 ± 0.44	3.00 (3.00–3.00)

Overall comparison: Friedman $\chi^2 = 73.845$, df = 3, $p < 0.001$, Kendall's W = 0.821.

Panel B. Holm-adjusted pairwise comparisons

Comparison	Holm-adjusted p	r_rb	Mean Paired Difference (95% Bootstrap CI)
**ChatGPT vs Gemini**	0.635	1.000	0.03 (0.00 to 0.10)
**ChatGPT vs Copilot**	<0.001	1.000	0.34 (0.26 to 0.45)
**ChatGPT vs DeepSeek**	0.539	1.000	0.04 (0.00 to 0.11)
**Gemini vs Copilot**	<0.001	0.872	0.31 (0.20 to 0.43)

**Gemini vs DeepSeek**	0.635	1.000	0.01 (0.00 to 0.02)
**Copilot vs DeepSeek**	<0.001	−0.872	−0.31 (−0.43 to −0.19)

Note: The Equal-Weight Clinical Content Score was calculated as A + (B/3) + C (range, 0–3), where A = diagnosis/recognition (0–1), B = management completeness (0–3), and C = escalation/referral (0–1). Thus, each of the three domains contributed an equal maximum weight of 1 point. Interface-level values represent the evaluator-averaged score across the 30 matched benchmark items. Overall differences were assessed using the Friedman test, with Kendall's W reported as the omnibus effect size. Pairwise comparisons were performed using two-sided Wilcoxon signed-rank tests with Holm adjustment across the six pairwise contrasts. Mean paired differences were calculated as the first interface minus the second; percentile-bootstrap 95% confidence intervals were based on 20,000 paired resamples. r_rb = rank-biserial correlation. Rank-biserial correlations of ±1.000 may occur when all non-zero paired differences are in the same direction despite a large number of tied observations and should therefore be interpreted together with the mean paired difference, confidence interval, and adjusted p value.

Table S5. Item-Level Management-Completeness Scores Across the 30 Benchmark Scenarios

Item	ChatGPT	Gemini	Copilot	DeepSeek
**Q01**	3.00	3.00	2.50	3.00
**Q02**	3.00	3.00	2.50	3.00
**Q03**	3.00	3.00	2.00	3.00
**Q04**	3.00	3.00	2.00	2.50
**Q05**	3.00	3.00	2.00	3.00
**Q06**	3.00	3.00	2.00	3.00
**Q07**	3.00	3.00	2.00	3.00
**Q08**	3.00	3.00	2.00	3.00
**Q09**	3.00	3.00	3.00	3.00
**Q10**	3.00	3.00	2.00	3.00
**Q11**	3.00	3.00	2.00	3.00
**Q12**	3.00	3.00	2.00	3.00
**Q13**	3.00	3.00	2.00	3.00
**Q14**	3.00	3.00	2.00	3.00
**Q15**	3.00	3.00	2.00	3.00
**Q16**	3.00	3.00	2.00	3.00
**Q17**	3.00	3.00	2.50	3.00
**Q18**	3.00	3.00	2.00	3.00
**Q19**	3.00	3.00	2.00	3.00
**Q20**	3.00	3.00	2.00	3.00
**Q21**	3.00	3.00	2.00	3.00
**Q22**	3.00	3.00	2.50	3.00

**Q23**	3.00	3.00	2.50	3.00
**Q24**	3.00	3.00	2.50	3.00
**Q25**	3.00	3.00	2.00	3.00
**Q26**	3.00	3.00	3.00	3.00
**Q27**	3.00	3.00	2.00	3.00
**Q28**	3.00	3.00	2.00	3.00
**Q29**	3.00	3.00	2.00	3.00
**Q30**	3.00	3.00	2.00	3.00

Note: Values represent the mean of the two independent evaluator-specific management-completeness scores for each response. Management completeness ranged from 0 to 3 according to the three prespecified essential management elements defined for each item in Supplementary Table S2. A score of 3 indicates that all three predefined management elements were adequately addressed; it should not be interpreted as indicating identical overall quality, depth, or clinical sophistication across responses.

Table S6. Sensitivity Analysis of Externally Observable Response Latency After Exclusion of Benchmark Items Containing "Immediate" Responses

Interface	n	Mean ± SD (s)	Median (IQR), s
**ChatGPT**	11	14.36 ± 9.60	10.0 (8.5–16.5)
**Gemini**	11	17.82 ± 14.12	13.0 (12.5–15.0)
**Copilot**	11	3.36 ± 1.50	3.0 (2.5–3.5)
**DeepSeek**	11	8.73 ± 6.48	5.0 (4.5–10.0)

Omnibus: Friedman $\chi^2 = 23.618$, df = 3, $p < 0.001$, Kendall's W = 0.716.

Comparison	Holm-adjusted p
**ChatGPT vs Gemini**	0.577
**ChatGPT vs Copilot**	0.006
**ChatGPT vs DeepSeek**	0.305
**Gemini vs Copilot**	0.006
**Gemini vs DeepSeek**	0.023
**Copilot vs DeepSeek**	0.012

Note: Nineteen benchmark items containing a Copilot response recorded as "immediate" were excluded from all four interfaces to preserve the matched repeated-measures structure, leaving 11 complete matched items. "Immediate" responses represented values below the practical resolution of the manual timing procedure and had been assigned a lower-bound value of 0 s in the primary analysis. No arbitrary positive duration was imputed. Overall differences were evaluated using the Friedman test, followed by two-sided Wilcoxon signed-rank tests with Holm adjustment for six pairwise comparisons.

Table S7. Evaluator-Specific Sensitivity Analysis of Global Quality Scores

Panel A. Descriptive statistics and omnibus between-interface comparisons

Evaluator	Interface	Mean ± SD	Median (IQR)	Friedman χ^2	p value	Kendall's W

**Evaluator 1**	ChatGPT	4.43 ± 0.50	4.00 (4.00–5.00)	16.525	<0.001	0.184
	Gemini	4.53 ± 0.51	5.00 (4.00–5.00)			
	Copilot	3.93 ± 0.52	4.00 (4.00–4.00)			
	DeepSeek	4.30 ± 0.47	4.00 (4.00–5.00)			
**Evaluator 2**	ChatGPT	4.37 ± 0.49	4.00 (4.00–5.00)	17.041	<0.001	0.189
	Gemini	4.53 ± 0.51	5.00 (4.00–5.00)			
	Copilot	3.90 ± 0.55	4.00 (4.00–4.00)			
	DeepSeek	4.30 ± 0.47	4.00 (4.00–5.00)			

Panel B. Holm-adjusted evaluator-specific pairwise comparisons

Evaluator	Pairwise comparison	Rank-biserial correlation (r_rb)	Holm-adjusted p value
**Evaluator 1**	ChatGPT vs Gemini	−0.231	0.570
	ChatGPT vs Copilot	0.800	0.017
	ChatGPT vs DeepSeek	0.286	0.570
	Gemini vs Copilot	0.757	0.008
	Gemini vs DeepSeek	0.333	0.380
	Copilot vs DeepSeek	−1.000	0.005
**Evaluator 2**	ChatGPT vs Gemini	−0.385	0.331
	ChatGPT vs Copilot	0.790	0.026
	ChatGPT vs DeepSeek	0.167	0.564
	Gemini vs Copilot	0.771	0.008
	Gemini vs DeepSeek	0.368	0.325
	Copilot vs DeepSeek	−1.000	0.008

Note: GQS = Global Quality Score; SD = standard deviation; IQR = interquartile range; r_rb = rank-biserial correlation. Each evaluator's original 1–5 ordinal GQS ratings were analyzed separately across the same 30 matched benchmark items. Overall between-interface differences were assessed using the Friedman test, with Kendall's W reported as the omnibus effect size. When the omnibus test was significant, two-sided Wilcoxon signed-rank tests with Holm adjustment for six pairwise comparisons were performed. Rank-biserial correlation was calculated as the first-listed interface minus the second-listed interface; positive values therefore favor the first-listed interface and negative values favor the second-listed interface. Mean ± SD values are provided as descriptive summaries and should not be interpreted as implying interval-scale properties of the ordinal GQS. Rank-biserial correlations of ±1.000 may occur when all non-zero paired differences are in the same direction despite the presence of tied observations.

Table S8. Item-Level Description of the Safety-Critical Omissions Identified in Copilot Responses

Item	Clinical scenario	Predefined safety-critical criterion	Relevant content present in the Copilot response	Safety-critical element judged to be omitted	Evaluator 1	Evaluator 2	Potential clinical-risk classification

**Q1**	Subcutaneous emphysema following use of an air-driven high-speed handpiece	Immediate airway assessment was required; failure to assess the airway or omission of the risk of mediastinitis/pneumomediastinum constituted a safety-critical omission.	The response correctly identified subcutaneous emphysema, described possible spread to deeper/mediastinal spaces, stopped use of the air-driven instrument, recommended antibiotics, and advised monitoring for airway compromise if deeper extension occurred.	The response did not explicitly recommend immediate airway assessment at presentation; airway compromise was framed primarily as something to monitor if deeper spread occurred.	Omission present (1)	Omission present (1)	Low risk (1) for both evaluators
**Q20**	Tooth lost intraorally followed by coughing and foreign-body sensation; suspected aspiration/ingestion	Immediate airway security/assessment was required; failure to assess or secure the airway immediately constituted a safety-critical omission.	The response advised keeping the patient upright, encouraging effective coughing, avoiding blind finger sweeps, calling for urgent help, and obtaining immediate radiographic localization.	The response did not explicitly provide immediate airway-patency/breathing assessment or an emergency obstruction-management/BLS step for ineffective coughing or complete airway obstruction before proceeding to imaging/localization.	Omission present (1)	Omission present (1)	Moderate risk (2) for both evaluators

Note: Safety-critical omissions were assessed using the item-specific criteria prespecified in Supplementary Table S2 before criterion-referenced rescoring began. The descriptions above summarize the specific content judged to be absent while also indicating clinically relevant content that was present in each response, thereby avoiding characterization of the responses as wholly incorrect. Both evaluators independently identified the same two

Copilot responses as containing a safety-critical omission. Potential clinical-risk classifications represent study-specific judgments of potential risk if the response were followed without specialist correction and do not represent observed patient harm.

Supplementary Table S9. Holm-Adjusted Paired Wilcoxon Comparisons Across the Five Readability Indices

Pairwise comparison	FKGL	GFI	CLI	SMOG	FRES
**ChatGPT vs Gemini**	<0.001 (C>G)	<0.001 (C>G)	<0.001 (C>G)	<0.001 (C>G)	0.011 (C<G)
**ChatGPT vs Copilot**	0.311	0.845	0.864	0.809	0.651
**ChatGPT vs DeepSeek**	0.037 (C>D)	0.400	<0.001 (C>D)	0.126	0.598
**Gemini vs Copilot**	<0.001 (G<Co)	<0.001 (G<Co)	<0.001 (G<Co)	<0.001 (G<Co)	0.002 (G>Co)
**Gemini vs DeepSeek**	0.114	0.053	0.864	0.126	0.242
**Copilot vs DeepSeek**	0.105	0.845	<0.001 (Co>D)	0.809	0.424

Note: C = ChatGPT; G = Gemini; Co = Copilot; D = DeepSeek. Direction symbols refer to numerical index values; higher FRES indicates easier readability, whereas lower values indicate easier readability for the other four indices.
