# Supplementary material for: Comparative Evaluation of Large Language Model Interfaces in Third Molar Surgery Complication Scenarios: Response Quality, Clinical Content, Potential Clinical Risk, Readability, and Externally Observable Response Latency—A Cross-Sectional Comparative Benchmark Study
Source: J Clin Med. 2026 Sep 23;15(19):7397. doi: 10.3390/jcm15197397 (PMC13666355; doi:10.3390/jcm15197397)
Supplement: Supplementary file 1 [file jcm-15-07397-s001.zip › Supplementary File S1.pdf]

Supplementary File S1

Archived Interface-Generated Responses

DEEPSEEK QUESTIONS AND ANSWERS:

1. In a 27-year-old patient, during surgical extraction of an impacted mandibular right third molar, an air-driven high-speed handpiece is used for bone removal. Shortly after the procedure, sudden swelling develops in the right cheek and submandibular region, with palpable crepitus on examination. What complication does this presentation suggest, by what mechanism does it occur, and how should it be managed?

This presentation is pathognomonic for **cervicofacial subcutaneous emphysema (surgical emphysema)**—a well-documented iatrogenic complication in oral and maxillofacial surgery. The triad of acute onset, rapid extraoral swelling, and palpable crepitus immediately following the use of an air-driven handpiece renders the diagnosis unequivocal.
It is critical to distinguish this from an acute expanding hematoma (which would be firm, non-crepitant, and often bluish) or an early-onset gas-forming necrotizing fasciitis (which would present with systemic toxicity, fever, and severe pain out of proportion to the exam, typically hours to days later, not immediately upon procedure completion).
Mechanism of Occurrence (Biomechanics and Anatomy)
The pathophysiology is multifactorial, rooted in the physics of high-speed turbine handpieces and the regional fascial architecture:
The Air Source: Air-driven handpieces operate at driving pressures of 2–3 bar (approximately 30–45 psi). The exhaust air is directed out through the burr shaft or the front exhaust ports. During osteotomy for an impacted mandibular third molar, the surgical field is submerged in blood and irrigation. If the handpiece is held with excessive pressure against the bone, or if the burr becomes temporarily bound in the osteotomy trough, the exhaust ports become obstructed by bone or soft tissue. This forces the compressed air directly into the cancellous marrow spaces (intraosseous emphysema) or, more commonly, through the surgical defect in the lingual cortical plate.
The Fascial Pathway: The mandibular third molar region is a fascial crossroads. The air stream breaches the lingual periosteum and penetrates the sublingual and submandibular spaces, which communicate freely with the parapharyngeal and deep cervical spaces. Because the investing layer of the deep cervical fascia attaches to the hyoid bone inferiorly, the air preferentially dissects in a cephalad direction (into the buccal, masseteric, and pterygoid spaces—hence the cheek swelling) but also tracks inferiorly along the carotid sheath and the loose areolar tissue of the submandibular triangle (hence the submandibular swelling). The crepitus is the physical palpation of this interstitial air dissecting along fascial planes and distending the connective tissue septae.
Textbook Correlate (Fonseca, Vol. 1 - Principles of Surgery): The primary etiological factor is the failure to maintain adequate irrigation such that the water coolant does not fully envelop the burr tip, combined with the inherently high exhaust pressure of the turbine, which can exceed normal tissue turgor pressure (typically 15–20 mmHg) by a factor of over 100.
Management Protocol (Evidence-Based, Stepwise)
This is almost always a self-limiting, benign condition if managed correctly. The primary goals are to prevent secondary infection, monitor for airway compromise, and accelerate nitrogen resorption. Intervention is entirely medical—never surgical.
Phase 1: Immediate Assessment (The "Rule-Out" Phase)
Auscultation and Palpation: Carefully palpate the extent of crepitus from the malar region down to the clavicular notch. Auscultate the precordium and neck for a "Hamman's crunch" (mediastinal crunch) to rule out pneumomediastinum—which, while rare, is a documented sequela if the air tracks into the superior mediastinum.
Vital Signs: Ensure the patient is normotensive, afebrile, and maintaining an oxygen saturation > 96% on room air.
Imaging: While a standard panoramic radiograph may show radiolucent striations in the soft tissues, a CBCT or cervical CT is the gold standard to definitively delineate the extent of air dissemination and to rule out an unrecognized pneumothorax or pneumomediastinum, especially if crepitus extends below the sternal notch.
Phase 2: Definitive Medical Management (The "Standard of Care")
High-Flow Supplemental Oxygen (100% O_2 via non-rebreather mask):
Mechanism: Subcutaneous air is roughly 78% nitrogen. Nitrogen has a very low

solubility coefficient. By increasing the patient's arterial oxygen tension (FiO_2 to 1.0), you create a steep partial-pressure gradient. This increases the rate of nitrogen absorption into the capillaries and venous system by a factor of 4 to 6 times, reducing the clinical resolution time from 5–7 days down to 24–48 hours.

Aggressive Prophylactic Broad-Spectrum Antibiotics:

Rationale: The dissecting air creates a non-vascularized potential space that can serve as a culture medium for oral anaerobes. Secondary infection of an emphysematous space can rapidly progress to mediastinitis or necrotizing fasciitis, which carries a high mortality.

Regimen: Amoxicillin-Clavulanate 875/125 mg BID (or Clindamycin 600 mg IV/PO QID if penicillin-allergic) plus Metronidazole 500 mg TID for a full 7-day course to cover the polymicrobial oral flora.

Strict Valsalva Avoidance:

Verbally and literally enforce an absolute prohibition on nose-blowing, sneezing with a closed mouth, smoking, and the use of straws for 10–14 days to prevent further positive intranasal/intraoral pressure from exacerbating the dissection.

Supportive Analgesia and Monitoring:

Prescribe NSAIDs (e.g., Ibuprofen 600 mg TID) for inflammatory pain. Admit the patient for 24-hour inpatient observation if there is any extension into the parapharyngeal space or if the patient has a short, obese neck, which predisposes to occult airway edema. Otherwise, schedule a telephonic check-in at 12 hours and a clinical follow-up at 48 hours.

Absolute Contraindications and Pitfalls to Avoid

DO NOT perform an incision and drainage, place a negative-pressure drain, or attempt to "milk" the air out. The skin barrier is intact; violating it introduces exogenous pathogens into a sterile dissected space and will not evacuate the air effectively, as it is trapped within fascial septae.

Prognosis and Follow-Up

With high-flow oxygen and appropriate antibiotics, the crepitus will markedly diminish within 24 hours and completely resolve within 3 to 5 days. A 2-week postoperative review is standard to confirm complete resolution and to evaluate the extraction site for normal bony healing.

Final Clinical Pearl: This complication is entirely preventable. In contemporary OMFS practice, for all impacted mandibular third molar osteotomies, I strongly advocate for the exclusive use of piezoelectric ultrasonic bone surgery or surgical electric handpieces (with internal irrigation and no exhaust air), which completely eliminate this iatrogenic risk. In my formal surgical dictation, I would document this event as an "acute iatrogenic cervicofacial surgical emphysema" and note that the patient was managed expectantly with supplemental oxygen, prophylactic antibiotics, and strict Valsalva precautions without further incident.

2. In a 25-year-old patient, during removal of a fractured apical root fragment after extraction of tooth 28, the fragment is displaced apically and separated from the socket. Radiographs show that the fragment has been displaced into the maxillary sinus. What clinical pathway should be followed in this situation?

• Based on the clinical scenario you described (25-year-old patient, fractured apical root fragment of tooth 28 displaced into the maxillary sinus), here is the recommended clinical pathway:

• Immediate Actions (At the Time of Displacement)

• Do NOT attempt blind probing or retrieval through the socket. This risks pushing the fragment deeper, damaging the sinus membrane further, or introducing infection.

• Assess the situation:

o Confirm the displacement with a perioperative radiograph (e.g., periapical, occlusal, or cone-beam CT if immediately available).

o Evaluate the size of the fragment (apical third of a root is typically small).

o Assess the integrity of the sinus membrane: Note if there is a clear communication (oro-antral communication/OAC) or if the membrane is perforated but the opening may be small.

• Initial management of the socket:

o Gentle irrigation with sterile saline.

o Do not curette the socket aggressively.

o Consider placing a small collagen plug or absorbable gelatin sponge into the socket apex to promote clotting and potentially act as a barrier. Avoid packing material deeply into the sinus.

o Suture the gingival margins of the socket loosely to stabilize the clot, but not tightly

enough to trap infection.
• Postoperative Management & Decision Pathway
• The key decision is whether to retrieve the fragment immediately or manage it conservatively. This depends on multiple factors.
• Pathway A: Conservative/Expectant Management (Often First-Line for Small, Apical Fragments)
• This is a valid and frequently recommended approach for a small, sterile, non-mobile apical fragment in a healthy sinus.
• Rationale: The fragment may become encased in the sinus mucosa, remain asymptomatic, and not cause complications. Unnecessary surgery to retrieve it carries its own risks.
• Actions:
o Prescribe appropriate medications:
▪ Antibiotics: A course of broad-spectrum antibiotics (e.g., amoxicillin-clavulanate) is standard to prevent acute sinusitis.
▪ Nasal decongestants: Use of a nasal spray (e.g., oxymetazoline) for 3-5 days to promote sinus drainage and ostium patency.
▪ Analgesics.
o Give detailed patient instructions:
▪ No nose blowing, sneeze with mouth open, avoid sucking on straws, avoid strenuous activity for 7-10 days (to prevent dislodging the clot and creating a permanent OAC).
▪ Report any signs of sinusitis immediately (facial pain/pressure, purulent nasal discharge, fever, foul taste).
o Close Follow-up: Monitor clinically for 1-2 weeks for signs of infection or OAC formation.
• Pathway B: Surgical Retrieval (Indicated in Specific Scenarios)
• Retrieval is typically planned as an elective procedure after initial inflammation subsides (unless acute infection mandates it).
• Indications for Surgical Retrieval:
• The fragment is large or contains a foreign body (e.g., post core).
• The patient develops acute or chronic maxillary sinusitis that does not resolve with antibiotics.
• The fragment is mobile within the sinus (seen on radiographs) and acts as a nidus for infection.
• The fragment is positioned in a way that obstructs the sinus ostium.
• The oro-antral communication (OAC) is large (>4-5mm) and will require surgical closure regardless; retrieval can be combined.
• Patient preference after being informed of risks/benefits.
• Surgical Retrieval Options:
• Caldwell-Luc Procedure:
o The classic approach. An intraoral incision in the maxillary vestibule, creation of a bony window in the anterior maxillary wall, and retrieval of the fragment under direct vision.
o Allows for inspection of the sinus, removal of diseased mucosa, and closure of any OAC.
o More invasive; risk of infraorbital nerve injury, postoperative swelling.
• Endoscopic Sinus Surgery (Functional Endoscopic Sinus Surgery - FESS):
o Often the preferred modern approach. Performed by an ENT surgeon.
o The fragment is retrieved via the natural or enlarged middle meatal ostium using an endoscope.
o Minimally invasive, better preserves sinus physiology, faster recovery.
o Requires collaboration with an ENT specialist.
• Combined Approach: In some cases, a small intraoral flap may be raised to gently probe the socket/sinus floor, and the fragment may be irrigated out or retrieved if easily accessible, sometimes guided by endoscopy.
• Recommended Clinical Pathway Summary
• Immediate: Confirm radiographically, avoid blind probing, manage socket conservatively, prescribe antibiotics/decongestants.
• Initial Decision Point (First 24-48 hours): Based on fragment size and initial presentation, decide on primary intent (conservative vs. planned surgical retrieval). For a small apical fragment, start with conservative management.
• Short-Term (1-2 week follow-up):

o If patient remains asymptomatic, continue monitoring. Obtain a CBCT scan if not already done to precisely localize the fragment and assess sinus health. Long-term radiographic monitoring may be advised.
o If patient develops symptoms of sinusitis, initiate more aggressive treatment and likely plan for surgical retrieval.
• Long-Term: If the fragment remains asymptomatic, it can often be left in situ indefinitely with periodic observation. The patient should be informed of its presence and told to report any future sinus issues.
• Critical Step: Documentation and Referral
• Document thoroughly the event, discussion with the patient, and the treatment plan.
• Referral to an oral and maxillofacial surgeon or an ENT surgeon is strongly recommended for co-management, especially if surgical retrieval is considered. They can best assess the need for and approach to retrieval.
• In essence, the pathway shifts from immediate prevention of infection, to a trial of conservative management for a small fragment, with a low threshold for specialist referral and planned surgical retrieval if complications arise.
3. In a 29-year-old patient, during extraction of a deeply impacted tooth 48 that is related to the inferior alveolar nerve, a fractured apical root fragment is displaced apically and separated from the socket during attempted retrieval. Radiographs show that the fragment has been displaced into the inferior alveolar nerve canal. What clinical pathway should be followed, and what should the treatment approach be?
• This is a complex and significant complication. The primary goals in managing this situation are to prevent or manage neurosensory deficits and avoid infection. Here's a structured clinical pathway and treatment approach:
• Immediate Clinical Pathway (At the Time of the Procedure)
• Immediate Cessation & Assessment:
o Stop all attempts at retrieval from within the socket. Do NOT attempt to blindly explore or "fish" for the fragment in the canal, as this will almost certainly worsen nerve injury.
o Document the event clearly in the operative notes, including the pre-operative risk, the event of displacement, and immediate findings.
o Inform the patient and/or family about the complication in a clear, factual, and compassionate manner. This is a critical step for informed consent for subsequent management.
• Immediate Radiographic Confirmation:
o Obtain a CBCT scan immediately, if available in the clinic/hospital. A periapical or panoramic film can confirm displacement, but a CBCT is essential to determine the exact 3D location of the fragment within the canal, its relationship to the neurovascular bundle, and its size. This will guide all future decision-making.
• Immediate Medical Management:
o Antibiotics: Prescribe a course of broad-spectrum antibiotics (e.g., amoxicillin/clavulanic acid or clindamycin if penicillin-allergic) to prevent infection of the fragment within the canal, which could lead to osteomyelitis or neuritis.
o Corticosteroids: Consider a short, tapering course of oral corticosteroids (e.g., dexamethasone) to reduce acute inflammatory edema around the nerve, potentially limiting initial neuropraxia.
o Analgesia: Prescribe appropriate analgesia (NSAIDs are first-line).
o Antiviral Prophylaxis: In some protocols, antiviral medication (e.g., valacyclovir) is considered if there is significant nerve manipulation, to prevent reactivation of latent herpes simplex virus, which can complicate neural healing.
• Baseline Neurosensory Testing (NST):
o Before the local anesthetic wears off, explain to the patient that they will need to monitor sensation.
o As soon as anesthesia subsides (typically the next day), perform and document a detailed baseline neurosensory examination. This includes:
▪ Light touch (cotton wisp)
▪ Two-point discrimination
▪ Static/sharp touch discrimination (blunt vs. sharp)
▪ Directional sense (brush stroke)
▪ Thermal discrimination

o Use a standardized form/map to document the precise area of altered sensation (lower lip, chin, gingiva, teeth). Photographs can be helpful.
• Definitive Treatment Approach & Decision Making
• The decision is between Conservative Monitoring and Surgical Retrieval. There is no universal consensus, and the decision must be individualized based on the factors below.
• Option 1: Conservative/Non-Surgical Management (Often Preferred Initially)
• This is the first-line approach in many cases, especially if the fragment is small, non-infected, and the nerve deficit is minimal.
• Indications:
o Small fragment (< 2-3 mm), with smooth, non-irritating edges.
o Fragment is located within the canal, not causing compression or significant disruption of the nerve bundle.
o Patient has mild hypoesthesia or paresthesia (not anesthesia dolorosa or severe pain).
o No signs of infection.
• Management:
o Continue antibiotics to complete a full course.
o Serial Clinical and Radiographic Monitoring: Follow-up at 1 week, 1 month, 3 months, 6 months, and 1 year.
o Repeat Neurosensory Testing at each visit to document recovery, stabilization, or deterioration.
o Repeat CBCT at 3-6 months to ensure fragment remains quiescent (no infection, no migration).
o Referral to Microsurgeon: Even if monitoring conservatively, an early consultation with an oral and maxillofacial surgeon experienced in nerve repair is wise for co-management and baseline assessment.
• Option 2: Surgical Retrieval (Exploration and Removal)
• This approach carries a significant risk of causing further nerve damage but may be necessary.
• Indications:
o Infection: Signs of acute or chronic infection (pain, swelling, purulence) related to the fragment.
o Severe or Progressive Neurologic Symptoms:
▪ Anesthesia (complete loss of sensation) - suggests severe disruption.
▪ Painful paresthesia/dysesthesia (burning, shocking pain) - suggests active neuritis or compression.
▪ Progressive worsening of neurosensory deficit over time.
o Large fragment (> 4-5 mm) with sharp edges, likely causing ongoing compression.
o Fragment is mobile or displaced further on follow-up imaging.
o Patient's choice after understanding the risks of persistent numbness vs. risks of surgery.
• Surgical Procedure:
o Performed by a Specialist: Must be done by an oral and maxillofacial surgeon with expertise in nerve repair, ideally with microsurgical training.
o Approach: Requires a decompression and exploration of the inferior alveolar nerve canal via a buccal corticotomy in the mandibular ramus/body region (not via the extraction socket).
o Technique: Use of the operating microscope or high-power loupes, micro-instruments, and continuous intraoperative nerve monitoring.
o Goal: Carefully remove the fragment while preserving the nerve bundle. If the nerve is severed or severely damaged, primary neurorrhaphy (nerve repair) or cable grafting (using a donor nerve like the great auricular) may be required at the same time.
• Summary of Recommended Pathway
• Immediate: Stop, CBCT, inform patient, start antibiotics/steroids, baseline NST.
• Refer: Early referral to an oral and maxillofacial surgeon with microneurosurgery experience.
• Decide (Shared Decision-Making):
o If asymptomatic or mild symptoms → Conservative monitoring with serial NST and CBCT.
o If infection, severe/worsening pain, or profound deficit → Surgical exploration and retrieval by a specialist.

• Long-term: Monitor for recovery (which can take up to 18-24 months). If dense anesthesia or painful dysesthesia persists beyond 3-6 months with no improvement, surgical exploration and possible nerve repair/grafting should be strongly considered, as outcomes diminish with time.
• Key Medicolegal Principle: Meticulous documentation of every step—the complication, the discussion with the patient, the findings, and all treatment decisions—is absolutely critical.

4. In a 28-year-old patient using oral contraceptives, severe pain and a foul odor develop a few days after extraction of tooth 38. Which postoperative complication is this most consistent with, and how should treatment be planned?

• Based on the clinical presentation, this is most consistent with alveolar osteitis (dry socket).
• Here's the reasoning and treatment plan:
• 1. Why this fits alveolar osteitis
• Timing: Onset "a few days after extraction" matches the typical presentation of alveolar osteitis (post-op days 2–4).
• Symptoms: Severe pain (often disproportionate to clinical findings) and foul odor are classic features.
• Risk factors:
o Oral contraceptives → increased estrogen → possible impaired healing or higher fibrinolytic activity in the socket.
o Tooth 38 (mandibular third molar) → lower molars, especially impacted third molars, are at highest risk.
• Other postoperative complications (e.g., infection, hematoma, fracture) usually present differently (swelling, fever, purulence, visible bone exposure not always required for diagnosis of dry socket; the key is painful, empty socket with clot loss).
• 2. Treatment plan
• A. Diagnosis confirmation:
• Clinical exam: visual and gentle probing to check for exposed bone, necrotic clot debris, empty socket.
• Exclude other causes: radiograph if bony fragments or infection suspected.
• B. Management:
• Analgesia:
o NSAIDs (ibuprofen, etc.) often needed, possibly opioid-containing analgesics if pain severe.
• Local socket care:
o Gentle irrigation with warm saline to remove debris.
o Medicated dressing placement (e.g., zinc oxide-eugenol, Alvogyl, or similar dry socket paste) to provide analgesia and protect socket. Dressing may be changed every 24–48 hours until symptoms subside (usually 3–7 days).
• Supportive advice:
o Avoid smoking, vigorous rinsing, straw use.
o Maintain oral hygiene with gentle rinsing (e.g., chlorhexidine 0.12% after 24 hours post-dressing).
• Follow-up: Usually resolves in 7–10 days with symptomatic treatment; ensure no secondary infection develops.
• C. Preventive consideration for future extractions:
• For patients on oral contraceptives, schedule extraction during the placebo pill week (lowest estrogen) to reduce risk.
• Use atraumatic surgery, consider topical antifibrinolytic agents or socket dressings prophylactically in high-risk cases.
• If signs of true infection (fever, swelling, purulence, lymphadenopathy) were present, antibiotic therapy would be considered, but alveolar osteitis is primarily a localized post-extraction healing disturbance without significant bacterial infection initially.

5. In a 24-year-old patient, persistent numbness of the lower lip and chin region for three months following a difficult mandibular right 48 extraction suggests which neurological complication, and how should this condition be managed?

• Based on the information provided, the most likely neurological complication is inferior alveolar nerve (IAN) injury, which has resulted in neuropathic pain and/or persistent anesthesia, paresthesia, or dysesthesia (in this case, described as numbness) in the distribution of the mental nerve (the terminal branch of the IAN).
• Detailed Explanation:

• Anatomy & Cause: The mandibular right third molar (tooth 48) is in close proximity to the inferior alveolar canal. A "difficult extraction" (often involving sectioning, significant bone removal, or elevation) can directly traumatize the IAN through:
o Stretching (neuropraxia): Most common, often reversible.
o Crushing or partial severing (axonotmesis): More severe, recovery is slower and may be incomplete.
o Complete transection (neurotmesis): Most severe, spontaneous recovery is unlikely.
o Other mechanisms include post-operative inflammation, hematoma compression, or bone fragments impinging on the nerve.
• Presentation: Persistent numbness (anesthesia) of the lower lip and chin (mental nerve distribution) for three months indicates a sensory deficit that has lasted beyond the expected resolution time for temporary injury (which is typically days to a few weeks).
• Management Strategy:
• Management is guided by the principle of moving from conservative to interventional options, with timing being critical.
• 1. Immediate and Early Management (First 24-72 hours):
• Confirm Diagnosis: A thorough clinical neurosensory exam should map the exact area of deficit and test for different sensory modalities (light touch, pinprick, two-point discrimination).
• Imaging: A CBCT scan is the gold standard to assess the relationship between the extraction socket and the inferior alveolar canal, and to rule out obvious causes like a retained root fragment or a displaced implant/bone fragment compressing the nerve.
• Pharmacotherapy: Initiate a course of high-dose corticosteroids (e.g., methylprednisolone) to reduce inflammation around the nerve, especially if started within 72 hours. NSAIDs may also be used.
• Patient Counseling: Document the deficit thoroughly and inform the patient about the nature of the injury, prognosis, and management plan. This is crucial for medicolegal reasons and for setting realistic expectations.
• 2. Subacute Management (Weeks 1-12):
• Medical Management for Neuropathic Symptoms: If the numbness is bothersome or evolves into painful paresthesia/dysesthesia (burning, tingling, pain), neuropathic pain medications may be considered (e.g., gabapentin, pregabalin, tricyclic antidepressants like amitriptyline). A referral to a pain specialist may be appropriate.
• Monitoring & Re-evaluation: Perform regular neurosensory exams (e.g., every 4-6 weeks) to document any signs of spontaneous recovery.
• Adjuvant Therapy: Some evidence supports the use of high-dose Vitamin B complex (especially B12) and alpha-lipoic acid to support nerve regeneration. Low-level laser therapy (LLLT) may have a role, though evidence is mixed.
• 3. Management After 3 Months (Current Situation - Persistent Deficit):
• Since the deficit has persisted for 3 months with no improvement, it is now classified as a persistent sensory disturbance. The likelihood of full spontaneous recovery decreases significantly after this point.
• Re-evaluation: Repeat CBCT if not done initially. Perform a detailed, quantitative neurosensory test.
• Referral: The patient should be referred to an oral and maxillofacial surgeon with specific expertise in microneurosurgery.
• Microsurgical Intervention (Nerve Repair/Decompression):
o Indication: Considered if there is no improvement after 3-4 months, especially if imaging shows a clear mechanical cause (e.g., visible transection on MRI, compression by an implant or bone spicule).
o Procedure: Involves surgical exploration of the nerve, removal of any compressing material (decompression), and if a discontinuity is found, microsurgical repair (neurorrhaphy) or grafting.
o Timing: Optimal results from microsurgery are typically achieved if performed between 3-6 months post-injury, before irreversible degeneration occurs.
• Summary of Recommendations for this 24-year-old Patient:
• Immediate Steps:
o Conduct a detailed neurosensory examination and document findings.
o Obtain a CBCT scan to visualize the nerve canal in relation to the extraction site.
o Consider referral to an oral and maxillofacial surgeon for evaluation.
• Given the 3-month duration:

o The window for conservative/medical management alone has largely passed.
o Surgical consultation for possible microsurgical exploration and repair is now the primary consideration. The young age of the patient favors a more proactive approach, as healing potential is better and the long-term impact of the deficit is significant.
• Ongoing Care:
o Provide psychological support, as persistent numbness can significantly impact quality of life.
o Discuss all options, including the risks and benefits of surgery versus continued observation.
• Disclaimer: This information is for educational purposes. Clinical decisions must be made by the treating clinician based on a full evaluation of the patient.

6. In a 26-year-old patient, following lingual retraction during extraction of tooth 38, numbness and loss of taste develop on the left half of the tongue. Which nerve injury does this suggest, and how should it be treated?

• This presentation suggests injury to the lingual nerve, a branch of the mandibular division (V3) of the trigeminal nerve (CN V).
• Why the Lingual Nerve?
• Anatomy: During the extraction of a mandibular third molar (tooth 38), the lingual nerve is vulnerable as it lies close to the lingual cortical plate, often within the soft tissue of the lingual flap.
• Functions affected:
o Sensory: Provides general sensation (touch, pain, temperature) to the anterior two-thirds of the tongue (hence the numbness).
o Special sensory (taste): Carries taste fibers from the anterior two-thirds of the tongue via the chorda tympani, which is a branch of the facial nerve (CN VII) that joins the lingual nerve. Injury explains the loss of taste.
• Likely Mechanism
• Lingual retraction during surgery can cause:
• Compression/stretching (neuropraxia)
• Contusion or partial tear (axonotmesis)
• Complete transection (neurotmesis) – less common but possible
• Treatment & Management
• Immediate (at time of discovery):
• Document the deficit clearly.
• If a suture or instrument is visibly compressing the nerve, consider immediate release (if feasible and safe).
• Postoperative Management:
• Confirm diagnosis – Detailed neurosensory testing (light touch, two-point discrimination, taste assessment).
• Imaging – Consider a CBCT to evaluate the socket/lingual plate if bony impingement or fragment is suspected.
• Medication:
o High-dose corticosteroids (e.g., methylprednisolone tapered course) started early may reduce inflammation and edema.
o Some protocols include NSAIDs and vitamin B complex (neurotropic support).
• Monitor – Most cases are neuropraxic and recover spontaneously within days to weeks. Follow-up at 1, 4, 8, 12, and 24 weeks with neurosensory charting.
• Referral:
o If no improvement after 8–12 weeks, refer to an oral and maxillofacial surgeon or microneurosurgeon with experience in nerve repair.
o Consider microsurgical exploration and repair (neurolysis, neurorrhaphy, or nerve grafting) if severe dysfunction persists beyond 3–4 months, especially if transection was likely.
• Supportive care:
o Advise patient on tongue protection from accidental biting/burns due to numbness.
o Taste loss often recovers earlier than sensation.
• Prognosis
• Neuropraxia: Full recovery likely within weeks.
• Axonotmesis: Slow recovery over months (1–2 mm/day regeneration).
• Neurotmesis: Poor recovery without surgical intervention.
• Given the patient's age, healing potential is generally good, but early recognition and

appropriate management optimize outcomes.

7. In a 26-year-old patient, a tooth is lost during extraction of tooth 28, and two days later CBCT shows the tooth in the infratemporal fossa. What does this finding indicate, and what is the appropriate treatment approach?

• This clinical scenario indicates that tooth 28 (maxillary left first premolar) has been displaced into the infratemporal fossa during extraction.
• What this finding indicates:
• Mechanism of displacement:
• The infratemporal fossa lies posterior and superior to the maxillary tuberosity, bounded laterally by the ramus of the mandible, medially by the lateral pterygoid plate, and superiorly by the greater wing of the sphenoid. During extraction of upper posterior teeth, especially if there is:
o Excessive apical or distobuccal force,
o Poorly developed or fractured tuberosity,
o Pneumatized maxillary sinus,
• the tooth can be pushed through the thin posterior maxillary wall into the infratemporal space.
• Risk factors:
o Long roots directed posteriorly,
o Use of elevators in wrong direction,
o Poor surgical access/visualization,
o Anatomical variations (large sinus, thin bone).
• Potential complications if left untreated:
o Infection/abscess formation,
o Trismus due to involvement of pterygoid muscles or inflammation,
o Injury to neurovascular structures (inferior alveolar nerve, internal maxillary artery, pterygoid venous plexus),
o Foreign body reaction,
o Possible migration deeper into spaces.
• Appropriate treatment approach:
• Immediate management (at time of loss):
o If recognized immediately, avoid blind probing (risk of pushing tooth deeper or causing bleeding).
o Obtain imaging (CBCT ideally) to localize precisely.
• In this case (CBCT at 2 days):
o Assess: Exact 3D position relative to key structures (lateral pterygoid plate, pterygoid muscles, maxillary artery, mandibular nerve).
o Signs of infection: Check for fever, trismus, pain, swelling.
o Antibiotics: Prophylactic or therapeutic antibiotics (amoxicillin/clavulanate or clindamycin if penicillin-allergic) to prevent infection.
• Surgical retrieval:
o Approach: Usually via intraoral approach under general anesthesia.
o Technique:
• a. Extend incision from tuberosity area, possibly with a coronoidectomy or osteotomy of the lateral tuberosity/posterior maxilla for access.
• b. Sometimes, a hemicoronal or endoscopic-assisted approach may be considered if tooth is high and lateral.
o Consider imaging guidance (intraoperative navigation if available) due to complex anatomy.
• If asymptomatic and deeply embedded in asymptomatic patient?
o Some surgeons may opt for close observation if retrieval poses high risk (e.g., encased in dense fibrous tissue, far from vital structures, asymptomatic), but generally removal is recommended due to risk of late complications.
• Postoperative:
o Analgesics, antibiotics, soft diet.
o Monitor for bleeding, infection, nerve injury, trismus.
o Physical therapy if trismus persists.
• Summary:
• CBCT showing tooth 28 in the infratemporal fossa indicates an iatrogenic displacement requiring surgical retrieval via an appropriate approach (usually intraoral with possible tuberosity osteotomy), guided by precise 3D imaging and possibly intraoperative navigation. Observation is rarely advised given risks of infection, trismus, or vascular

injury over time.

8. In a 34-year-old patient, a mandibular basal (inferior border) fracture occurs during extraction of a deeply impacted tooth 38 due to excessive elevator force. What should the emergency management approach be?

• This scenario describes a iatrogenic mandibular fracture during a surgical dental procedure. The emergency management approach should be systematic, prioritizing patient stabilization, accurate diagnosis, and appropriate referral.
• Here is the recommended step-by-step emergency management approach:
• Immediate On-Site Actions (In the Dental Office/Surgery)
• Stop the Procedure Immediately: Discontinue any further attempts at tooth extraction.
• Stabilize the Patient & Reassure: Keep the patient in a seated or semi-reclined position. Explain what has happened calmly to reduce anxiety.
• Assess Airway, Breathing, and Circulation (ABC):
o This is paramount. A mandibular fracture, especially a bilateral one, can compromise the airway due to posterior displacement of the tongue or swelling. In this unilateral inferior border fracture, the airway is less likely to be immediately compromised, but it must be checked.
o Ask the patient to speak. Listen for clear speech or signs of obstruction (gurgling, stridor).
• Control Hemorrhage: Apply direct pressure with gauze to the extraction socket if there is bleeding. Significant bleeding from the fracture site itself is less common with an inferior border fracture but should be assessed.
• Immobilize the Jaw (Temporary Stabilization):
o Instruct the patient not to move their jaw.
o Apply a Barton bandage or a simple circumferential bandage (e.g., using a gauze roll or elastic bandage) under the mandible and over the top of the head. The goal is to support the jaw, not to reduce the fracture. This minimizes pain and prevents further displacement.
• Document Neurological Status: Briefly check for neurosensory deficit of the inferior alveolar nerve (numbness of lower lip and chin on the affected side), as the fracture line likely involves the mandibular canal. Document this finding.
• Analgesia: Administer appropriate pain relief (e.g., NSAIDs if not contraindicated, or other analgesics). Avoid sedatives that might depress respiration.
• Imaging: If available in the clinic (e.g., CBCT), obtain an urgent scan to fully assess the fracture location, displacement, comminution, and relationship to tooth roots and the inferior alveolar canal. At a minimum, take a panoramic radiograph (OPG) and a posteroanterior (PA) view of the mandible. Do not discharge the patient without imaging.
• Definitive Management (Requires Immediate Referral/Transfer)
• Emergency Referral: This is a maxillofacial surgery emergency. The patient must be immediately referred to a hospital with an oral & maxillofacial surgery department.
o Call for an ambulance if the patient shows any signs of airway distress, significant bleeding, or if the transfer via private vehicle is not immediate and safe.
o Contact the on-call maxillofacial surgeon directly to alert them and transmit any available images.
• Provide Documentation: Send all relevant records, including the preoperative consent, notes on the procedure, the emergency actions taken, and the radiographic images, with the patient.
• Definitive Hospital-Based Treatment (Managed by Maxillofacial Surgeons)
• Comprehensive Assessment: Clinical exam, detailed imaging (often CT scan for superior bony detail).
• Treatment Planning: The approach depends on fracture characteristics:
o Non-displaced or minimally displaced stable fracture: May be managed conservatively with soft diet, analgesics, and close observation, or with closed reduction and intermaxillary fixation (IMF) for a few weeks.
o Displaced or unstable fracture: Will likely require open reduction and internal fixation (ORIF). This involves surgical exposure of the fracture, anatomical reduction, and fixation using titanium plates and screws. The deeply impacted tooth 38 is typically removed during the same procedure if it is in the line of fracture or deemed non-salvageable.
• Antibiotics: Prophylactic antibiotics are usually administered due to the compound (open)

nature of the fracture (communication with the mouth via the socket).
• Tetanus Prophylaxis: Consideration based on patient's vaccination history.
• Key Points in This Specific Scenario:
• Age (34): The patient has good healing potential. The bone is mature but not osteoporotic.
• Fracture Type (Inferior Border/Basal): This is often a favorable fracture, but its stability depends on the direction and displacement. It still requires formal assessment.
• Cause (Excessive Elevator Force): Highlights the need for careful surgical technique, proper bone removal, and consideration for surgical sectioning of the tooth in deeply impacted cases to avoid excessive force.
• Medicolegal Duty: Thorough documentation of the event, informed consent about risks (including fracture) prior to surgery, and immediate, appropriate management are crucial.
• In summary, the core emergency approach is: 1) Stabilize the patient and the jaw, 2) Obtain imaging, and 3) Arrange immediate transfer to a maxillofacial surgery unit for definitive care.

9. In a 28-year-old patient, one week after extraction of tooth 48, progressively worsening severe pain, trismus, fever, and cervical lymphadenopathy develop. The patient also has dysphagia/odynophagia, diffuse neck swelling, and chest pain–shortness of breath, raising suspicion of spread of infection through deep neck spaces to the mediastinum. Which diagnosis does this presentation most strongly suggest?

• Based on the clinical presentation, the diagnosis this most strongly suggests is Ludwig's Angina with probable descending necrotizing mediastinitis.
• Here's the breakdown of why:
• Timeline and Origin: The symptoms began one week after the extraction of tooth 48 (the lower right third molar). This is a classic origin point for deep neck infections, as the roots of mandibular molars often involve the submandibular space.
• Key Clinical Features of Ludwig's Angina:
o Severe pain, trismus, and dysphagia/odynophagia: Direct results of a rapidly spreading cellulitis in the submandibular and sublingual spaces.
o Diffuse, firm "brawny" neck swelling: Bilateral swelling of the submandibular region, often described as giving a "wood-like" feel and potentially elevating the tongue.
o Fever and cervical lymphadenopathy: Signs of systemic infection.
• The Critical, Life-Threatening Progression:
o Spread through deep neck spaces: The infection from the submandibular space can track posteriorly along the fascial planes into the lateral pharyngeal space, and then further down.
o Chest pain and shortness of breath: These are alarm symptoms indicating the infection has likely descended along the retropharyngeal or danger space (prevertebral space) into the mediastinum, causing mediastinitis.
o This complication is known as descending necrotizing mediastinitis and carries a very high mortality rate.
• Why Other Diagnoses Are Less Likely:
• Simple dry socket (alveolar osteitis): Would cause localized pain at the extraction site but not the systemic symptoms, trismus, or diffuse neck swelling.
• Peritonsillar abscess: Typically presents with severe sore throat, trismus, and a "hot potato" voice, but the swelling is unilateral and centered on the tonsillar pillar, not originating from a recent dental procedure.
• Acute suppurative parotitis: Swelling would be centered in the parotid region (pre-auricular and along the angle of the mandible), not primarily submandibular.
• In summary: The progression from a mandibular molar extraction → severe submandibular cellulitis with trismus and dysphagia (classic Ludwig's Angina) → signs of mediastinal involvement (chest pain, shortness of breath) makes Ludwig's Angina with descending necrotizing mediastinitis the most compelling and urgent diagnosis. This is a true medical and surgical emergency requiring immediate airway management, IV antibiotics, and surgical drainage of both the neck and mediastinum.

10. In a 25-year-old patient, six weeks after mandibular 48 extraction, the patient reports food impaction and bleeding distal to tooth 47. Clinical examination reveals a ≥6 mm periodontal pocket distal to 47 with bleeding on probing; radiographs show localized bone loss distal to 47. What surgical and postoperative approach should be implemented in this case?

• Based on the clinical scenario, the findings are consistent with a periodontal defect distal to tooth 47 following extraction of the mandibular 48 (impacted third molar), likely a

distal-angular bony defect or possibly a three-wall infrabony defect (depending on radiograph and intraoperative findings).

- Here's a systematic surgical and postoperative approach:
- 1. Pre-surgical phase
- Confirm diagnosis: Periapical radiograph + possible CBCT to assess morphology of bone loss (vertical defect depth, number of walls, relation to furcation, root anatomy).
- Initial therapy: Full-mouth scaling and root planing, oral hygiene instruction to reduce inflammation before surgery.
- Patient factors: 25 years old → good healing potential, but smoking history (if any) should be addressed.
- 2. Surgical approach
- Flap design:

o An envelope or triangular mucoperiosteal flap from distal of 46 extending distal to 47, preserving as much keratinized tissue as possible.
o Possibly a distal wedge incision if there is redundant tissue.

- Debridement:

o Remove granulation tissue, curette socket remnants from extraction site, and thoroughly root plan distal surface of 47.

- Defect evaluation:

o Measure probing depth intraoperatively after debridement; assess number of bony walls.
o In young patients, if ≥3 walls remain, guided tissue regeneration (GTR) is often the first choice for predictable bone fill.

- Bone graft & membrane:

o Fill defect with bone graft material (xenograft/allograft preferred for space maintenance).
o Place a resorbable collagen membrane (barrier membrane for GTR) to cover the graft and defect, ensuring membrane stability.
o If defect is wide but shallow with 2 walls, consider simpler approach: open flap debridement + graft without membrane, but regeneration outcome is better with membrane in deep narrow defects.

- Closure: Primary closure with non-resorbable (e.g., PTFE) or resorbable sutures; ensure flap is tension-free and covers the membrane.
- 3. Postoperative care
- Antibiotics: Perioperative antibiotic coverage (e.g., amoxicillin 500 mg TID for 5–7 days) if significant infection preoperatively.
- Analgesics & chlorhexidine:

o Prescribe analgesics (NSAIDs) for pain.
o Chlorhexidine 0.12% mouth rinse twice daily for 2 weeks (no brushing/flossing near surgical site initially).

- Suture removal: At 10–14 days.
- Oral hygiene: Reinforce gentle brushing and interdental cleaning (avoid distal of 47 initially; after 2–3 weeks, use soft picks).
- Diet: Soft diet for 1–2 weeks to avoid food impaction.
- Recall: Post-op check at 1 week, 1 month, then periodontal maintenance schedule.

o Evaluate healing at 6 months with probing and radiograph to assess bone fill.

- 4. Long-term management
- Regular periodontal maintenance every 3–6 months.
- Possible need for occlusal adjustment if traumatic occlusion on 47.
- If regeneration fails or defect anatomy unfavorable, consider distal crown lengthening or root resection in future only if restorative needs dictate.
- Rationale
- Primary etiology: Post-extraction healing complications (likely residual debris/poor clot, leading to localized periodontitis).
- Goal: Regeneration of periodontal attachment and bone, not just pocket reduction, given patient's age.
- Prognosis: Good with proper defect management and oral hygiene.
- Summary answer:
- Perform open flap debridement, bone grafting, and guided tissue regeneration (GTR) with a resorbable membrane, followed by meticulous postoperative care including antibiotics, chlorhexidine rinses, soft diet, and staged reintroduction of oral hygiene. Regular periodontal follow-up is essential.

11. In a 29-year-old patient, recurrent bleeding from the extraction site begins approximately one week after extraction of mandibular left 38. What mechanism could explain this delayed bleeding, and how should it be managed?
• This is an important clinical scenario. Let's break it down systematically.
• Likely Mechanism: Secondary Hemorrhage (Reactionary Hemorrhage)
• The timing—approximately one week post-extraction—points strongly to secondary hemorrhage. This is delayed bleeding that occurs 24 hours to several days (often 5–10 days) after the initial procedure, and it has a different pathophysiology than immediate or reactionary (within 24 hours) bleeding.
• Primary Mechanisms at Play:
• Premature Dislodgement of the Blood Clot & Infection (Localized Alveolar Osteitis/"Dry Socket" with Hemorrhagic Component): This is a very common cause. The initial stable clot lyses or is dislodged, exposing the bone and vessels in the socket. The subsequent inflammatory process, often with bacterial infection (fibrinolytic activity), can erode into a small blood vessel within the bony walls.
• Wound Breakdown Over a Vessel: The initial clot may have sealed a small nutrient vessel in the bone (e.g., a branch of the inferior alveolar artery within the socket, or more commonly, a smaller intraosseous vessel). As the clot organizes and granulation tissue forms, premature breakdown can open this vessel.
• Underlying Local Infection (Suppuration): A localized socket infection can cause vasculitis (inflammation of vessel walls) and subsequent rupture of the vessel.
• Systemic Factors Unmasked by Healing: While less common, a systemic predisposition (e.g., undiagnosed mild coagulopathy, platelet disorder, or medication effect like NSAIDs/aspirin) may become apparent during the healing phase when the fragile clot or early granulation tissue is vulnerable.
• Important Note for Mandibular Third Molars: The 38 region has a rich vascular supply (inferior alveolar artery, lingual artery branches). The bony walls, especially if the tooth was deeply impacted, may contain small but persistent vessels that are prone to this delayed bleeding.
• Management
• Management follows a structured approach: Assess, Localize, Control, and Prevent Recurrence.
• 1. Initial Assessment & History (Critical First Step)
• Vital Signs: Check blood pressure, heart rate. Look for signs of hypovolemia or shock (tachycardia, hypotension) though rare in isolated dental bleeding.
• Medical History: Quickly re-evaluate:
o Any history of bleeding disorders (personal or familial)?
o Current medications (anticoagulants, antiplatelets, NSAIDs, herbal supplements like ginseng, garlic)?
o Recent trauma to the area?
o Signs of infection (pain, foul taste, swelling, fever)?
• 2. Local Anesthesia & Examination
• Administer local anesthetic with a vasoconstrictor (e.g., lidocaine with epinephrine). This provides pain control, reduces patient anxiety (which can elevate BP and worsen bleeding), and provides temporary hemostasis via vasoconstriction.
• Gently irrigate the socket with saline to remove loose clots and debris.
• Visualize and Localize the Bleeding Source:
o Is it generalized ooze from the entire socket? (Suggests clot breakdown/infection)
o Is it a pulsatile, brisk bleed from a specific pinpoint in the bone? (Suggests a small arterial vessel)
• 3. Local Hemostatic Measures
• Curottage & Pocket Placement: Gently curette the socket walls to stimulate fresh bleeding and formation of a new clot. This is often effective for generalized ooze.
• Packing with Hemostatic Agent: Place a resorbable hemostatic agent directly into the socket under pressure. Excellent options include:
o Oxidized Regenerated Cellulose (Surgicel)
o Gelatin Sponge (Gelfoam)
o Collagen-based sponges (CollaPlug, CollaTape)
o Tranexamic Acid-soaked gauze (antifibrinolytic) is particularly useful.
• Suture Over the Pack: Crucial step. Place a figure-of-eight or horizontal mattress suture over the socket to secure the hemostatic pack, protect the new clot, and physically oppose tissues. Use non-resorbable (e.g., silk) or long-lasting resorbable sutures for better

stability.
• For a Visible Vessel: If a specific bleeding vessel is seen in bone, consider electrocautery or bone wax for mechanical occlusion (if available and you are trained).
• 4. Systemic Measures & Adjuncts
• Tranexamic Acid Mouthwash: Prescribe 5% tranexamic acid mouthwash (hold over the area for 2-3 minutes, 4 times daily, for 2-3 days). Do not rinse vigorously.
• Antibiotics: If signs of infection are present (purulence, severe pain, erythema), prescribe a course of antibiotics (e.g., amoxicillin or clindamycin if penicillin-allergic). This treats the underlying cause.
• Analgesia: Prescribe appropriate pain relief. Avoid NSAIDs (like ibuprofen) at this stage due to their antiplatelet effect. Acetaminophen/paracetamol is preferred.
• Medical Consultation: If bleeding is severe, recurrent despite local measures, or a systemic cause is strongly suspected, consult the patient's physician or a hematologist. Blood work (CBC, coagulation profile) may be indicated.
• 5. Post-Operative Instructions
• Rest: Absolute rest, head elevated for 24-48 hours.
• Diet: Soft, cool diet. Avoid hot liquids, alcohol, and vigorous rinsing for 48 hours.
• Avoid Disruption: No spitting, sucking (e.g., through a straw), or smoking.
• Follow-up: See the patient in 24-48 hours to assess the socket and remove or adjust sutures if needed.
• Summary for the 29-year-old patient:
• The delayed bleeding is most likely secondary hemorrhage due to localized clot breakdown and infection, possibly from a small intraosseous vessel. Management involves thorough socket debridement under local anesthesia, placement of a resorbable hemostatic agent, and secure suturing to protect the new clot, combined with tranexamic acid mouthwash and antibiotics if infection is present. A careful review for underlying systemic factors remains prudent.

12. In a 22-year-old patient, soft tissue trauma–related tissue hemorrhage occurs during local anesthetic injection prior to mandibular 38 extraction. How would you proceed with the procedure in this situation?

• This is a common complication. Here is a step-by-step protocol for managing this situation in a 22-year-old patient:
• Immediate Actions (Within Seconds)
• Stop the Injection Immediately: Do not inject any more anesthetic.
• Maintain Pressure: Leave the needle in place for 10-15 seconds, then withdraw it. Immediately apply firm, direct, and continuous pressure to the area of bleeding (lateral to the injection site, in the medial aspect of the ramus/pterygomandibular space) for a minimum of 2-3 minutes. Use gauze and digital pressure.
• Reassure the Patient: Explain what happened in a calm manner. "We've had a little bit of bleeding into the tissues from the injection. This is a known possibility, and I'm applying pressure to control it."
• Assessment and Management (Next 5-10 Minutes)
• Evaluate the Hemorrhage: After applying pressure, assess the extent.
o Small, Localized Hematoma: If it's minor with minimal swelling and no trismus, proceed with the procedure after documenting.
o Significant Hematoma with Rapid Swelling: This indicates bleeding into the potential spaces of the pterygomandibular/infratemporal region.
▪ Continue Pressure: Apply cold compress externally (ice pack on the cheek/angle of mandible) to help vasoconstrict and limit spread.
▪ Monitor Airway: This is the primary concern. Ask the patient about breathing or swallowing difficulty. Observe for any signs of airway compromise (dysphonia, dysphagia, stridor), which would be a medical emergency.
▪ Assess Trismus: Check for any limitation in mouth opening due to muscle involvement.
• Decision Point: To Proceed or Postpone the Extraction?
• The decision is based on the severity of the hematoma, patient comfort, and surgical access.
• Proceed with Extraction (Minor Hematoma):
o If swelling is minimal, the patient is comfortable, and you have adequate anesthesia and surgical access, you can proceed with the extraction of tooth 38.
o Crucial: You must now use extreme caution with any subsequent injections (e.g.,

for buccal infiltration). Avoid the hematoma area. Consider alternative techniques (e.g., intraligamentary injection) if needed.
o Inform the patient that post-operative swelling and bruising will be more significant.
• Postpone the Extraction (Significant Hematoma):
o This is the safer and often recommended choice, especially if there is:
▪ Rapidly increasing swelling.
▪ Any trismus limiting access (< 20-25mm opening).
▪ Patient discomfort or anxiety.
▪ Concern about visualization or compromised surgical field.
o Rationale: The hematoma creates tissue distortion, increases the risk of further bleeding during surgery, and makes the procedure more traumatic. The local anesthetic may also be less effective due to diffusion.
• Post-Hemorrhage Instructions & Follow-up
• Post-Operative Instructions (Given whether you proceed or postpone):
o Ice: Apply ice packs (20 min on, 20 min off) for the first 24-48 hours.
o Analgesics: Prescribe/Recommend analgesics (avoid NSAIDs like ibuprofen for 24-48 hrs if concerned about bleeding; acetaminophen is preferred). Absolutely no aspirin.
o Soft Diet & Warm Salt Rinses: After 24 hours, gentle warm salt water rinses can promote resolution.
o Head Elevation: Sleep with head elevated.
o Warning Signs: Provide clear instructions to return immediately if they experience difficulty breathing, swallowing, significant increase in swelling, fever, or severe pain.
• Documentation:
o Meticulously document in the chart: the incident, the anesthetic used (including vasoconstrictor), the amount injected, your immediate management (pressure applied, duration), the patient's response, your assessment of the hematoma size and location, and your decision (to proceed or postpone) with justification.
o Take a clinical photo for the record if possible.
• Follow-up:
o If you postponed, reschedule the extraction in 7-14 days, once the hematoma has resolved and trismus (if any) has subsided.
o For a significant hematoma, consider a phone call follow-up the next day.
• Summary of Key Principles:
• Primary Goal: Stop the active bleeding with immediate, firm pressure.
• Primary Concern: Airway patency. Monitor closely.
• Key Decision Factor: Surgical Access. Trismus is a strong indicator to postpone.
• Communication: Keep the patient informed and reassured throughout.
• Documentation: Complete and accurate records are essential.
• For this 22-year-old patient, given the tissue resilience and healing capacity, if the hematoma is controlled and minor, proceeding can be considered. However, if there is any doubt about access or control, postponing is the most prudent and standard professional course of action.

13. A 25-year-old patient presents two days after mandibular 48 extraction with limited mouth opening and difficulty eating. How should the patient be assessed and managed?

• This patient is presenting with post-operative trismus following mandibular third molar extraction (tooth 48). The timing (2 days post-op) is consistent with the onset of inflammatory or infectious complications. My assessment and management approach would be as follows:
• 1. Immediate Assessment
• A. History (Focus on):
• Pain: Severity, character (throbbing vs. constant), and location (localized vs. radiating).
• Mouth Opening: Progressive worsening since surgery? Can they take liquids?
• Systemic Symptoms: Fever, chills, malaise, dysphagia, difficulty breathing.
• Oral Hygiene: Post-op care performed (e.g., saltwater rinses).
• Medications: Current analgesics, antibiotics (if prescribed), and compliance.
• B. Clinical Examination:
• Extra-oral:
o Swelling & Redness: Assess face, submandibular, and neck regions.
o Palpation: Check for tenderness, warmth, and lymphadenopathy.

o Measure Trismus: Inter-incisal distance at maximal opening (normal ~35-45mm; <20-25mm is significant trismus).
• Intra-oral (carefully, may be limited):
o Extraction Site: Check for alveolar osteitis (dry socket) — exposed bone, foul odor, necrotic clot.
o Signs of Infection: Purulent discharge, erythema, swollen flap.
o Hematoma/Edema: In surrounding tissues.
o Check for Pus: Apply gentle pressure on the buccal and lingual aspects of the extraction site.
o Muscle Tenderness: Palpate masseter, temporalis, and medial pterygoid muscles.
• C. Investigations (if indicated):
• Radiograph: A new panoramic or periapical X-ray if a retained root fragment or bony sequestrum is suspected.
• Blood Tests: Only if systemic infection is suspected (CBC, CRP).
• 2. Differential Diagnoses
• Post-operative inflammatory edema with muscle trismus (most common).
• Alveolar osteitis (dry socket).
• Localized infection (e.g., pericoronitis exacerbation, socket infection).
• Submasseteric space infection (deep space infection).
• Hematoma formation.
• Trauma to medial pterygoid/masseter muscles during injection or surgery.
• Temporomandibular joint (TMJ) irritation from prolonged opening.
• 3. Management
• A. Immediate/Primary Management:
• Analgesia: NSAIDs (e.g., ibuprofen) for inflammation and pain, unless contraindicated. Consider adding acetaminophen for breakthrough pain.
• Muscle Relaxants: A short course of a muscle relaxant (e.g., diazepam low dose at night) can help if muscle spasm is a major component.
• Antibiotics: Only if signs of infection are present (purulence, spreading cellulitis, systemic symptoms). Amoxicillin with clavulanic acid or clindamycin (if penicillin-allergic) is typical.
• Dry Socket Management: If diagnosed, irrigate gently with warm saline, place a sedative dressing (e.g., Alvogyl or zinc oxide/eugenol pack) to relieve pain.
• Encourage Mouth Opening: Gentle, passive stretching exercises (e.g., opening and closing within comfort limits, several times a day). No forceful stretching.
• B. Supportive & Adjunctive Measures:
• Moist Heat Application: Apply to the affected side of the face for 20 minutes, several times daily to reduce muscle spasm and improve blood flow.
• Soft Diet & Hydration.
• Oral Hygiene Instructions: Warm saltwater rinses (gently, without vigorous swishing) starting 24 hours post-op, after meals.
• C. Follow-up:
• Re-evaluate in 24-48 hours to assess response to treatment.
• If trismus worsens or systemic symptoms develop, consider imaging (CT scan) to rule out a deep space infection requiring incision and drainage.
• 4. When to Refer/Emergency Considerations
• Immediate referral to oral & maxillofacial surgery if:
o Signs of airway compromise (dyspnea, stridor, voice changes).
o Severe dysphagia or inability to handle secretions.
o Rapidly spreading cellulitis or signs of Ludwig's angina (bilateral submandibular swelling, tongue elevation).
o Suspected deep space infection (e.g., submasseteric, lateral pharyngeal).
o Trismus progressively worsening despite initial management.
• 5. Patient Education
• Explain that some trismus is common after lower third molar surgery, peaking at 2-3 days and usually resolving over 1-2 weeks.
• Reinforce the importance of gentle stretching exercises.
• Advise to return immediately if fever, difficulty breathing/swallowing, or severe worsening occurs.
• In summary: The initial step is a thorough history and clinical exam to distinguish between simple inflammatory trismus and a more serious infective complication. Management is directed at the underlying cause, with aggressive follow-up to ensure resolution and to

identify early any progression to deep space infection.

14. In a 55-year-old patient with a history of four doses of intravenous bisphosphonate (zoledronic acid) use, an unhealed area of exposed bone and pain develop weeks after extraction of mandibular 38. Which complication is most likely, and how should management be planned?

• This presentation is highly concerning for medication-related osteonecrosis of the jaw (MRONJ), which, given the history, is specifically bisphosphonate-related osteonecrosis of the jaw (BRONJ).
• Most Likely Complication: Stage 1 or early Stage 2 MRONJ.
• The key features aligning with MRONJ are:
• Antiresorptive Exposure: Four doses of intravenous zoledronic acid (a potent bisphosphonate) is a well-established major risk factor.
• Precipitating Event: Dental extraction (tooth #38) is the most common trigger.
• Classic Findings:
o Exposed bone in the jaw that has persisted for more than 8 weeks.
o Pain in the affected region.
• Location: The mandible is more commonly affected than the maxilla.
• Management Plan
• Management is staged based on the severity (AAOMS Staging System) and focuses on controlling symptoms, preventing progression, and treating infection. The following outlines a comprehensive approach:
• 1. Immediate Assessment & Confirmation:
• Detailed History & Exam: Confirm drug history (bisphosphonate type, duration, last dose), rule out other causes (e.g., radiation necrosis, osteomyelitis).
• Imaging:
o Panoramic Radiograph (OPG): To assess bone changes (e.g., osteolysis, sclerosis, sequestration) and rule out residual root fragments.
o Cone Beam CT (CBCT): Gold standard for evaluating extent of bony involvement, detecting early changes, and surgical planning. Shows classic features like osteolysis, sequestrum formation, and periosteal reaction.
• Culture & Sensitivity: If purulence is present, obtain a sample to guide antibiotic therapy.
• 2. Conservative Management (Mainstay for Stage 1 & 2):
• Oral Rinses: 0.12% Chlorhexidine gluconate mouth rinse twice daily. This is crucial for local debridement and infection control.
• Antibiotic Therapy: Penicillin-based therapy (e.g., Amoxicillin) or Clindamycin (if penicillin-allergic) is typically used for 1-2 weeks to manage secondary infection and pain. Therapy may be pulsed/intermittent based on symptoms.
• Analgesia: Manage pain with appropriate analgesics (e.g., NSAIDs, opioids if severe).
• Minor Debridement: Gentle removal of loose, superficial sequestra in the office under local anesthesia and chlorhexidine irrigation may be beneficial.
• Tobacco & Alcohol Cessation: Critical counseling point.
• Close Follow-up: Re-evaluate in 2-4 weeks to assess response.
• 3. Surgical Management (Considered for Progressive Disease or Stage 3):
• Indications: Failed conservative therapy, progression to Stage 2/3 (pain + infection + necrosis extending beyond alveolar bone, pathologic fracture, extra-oral fistula), or large areas of necrotic bone.
• Procedure: Conservative surgical debridement/resection (sequestrectomy) with primary mucosal closure, aiming to remove the necrotic bone while preserving viable tissue. *Aggressive resection is generally avoided* unless absolutely necessary.
• Important: The patient's prescribing physician (e.g., oncologist, endocrinologist) must be consulted before any surgery to discuss risks/benefits and the possibility of a "drug holiday." The decision to temporarily suspend bisphosphonate therapy is complex and depends on the underlying condition (e.g., cancer with bone metastases vs. osteoporosis).
• 4. Adjunctive Therapies & Multidisciplinary Care:
• Multidisciplinary Team is essential. Coordinate care with:
o Oral & Maxillofacial Surgeon (lead for local management).
o Treating Physician/Oncologist (manages systemic condition and antiresorptive therapy).
o Dentist/Prosthodontist (for oral hygiene and prosthetic adjustments).
• Possible Adjuncts: Pentoxifylline & Tocopherol (Vitamin E) regimen has shown promise in some studies to reduce bone exposure and pain. Low-level laser therapy (photobiomodulation) may aid in symptom relief and healing.

• Long-term Oral Maintenance: Meticulous oral hygiene, regular dental check-ups, and preventative care to avoid future dental extractions.
• 5. Patient Education & Prevention for Future:
• Emphasize the lifelong risk of MRONJ with any future invasive dental procedures.
• Stress the importance of optimal oral hygiene and regular dental evaluations.
• Any future dental surgery requires prophylactic planning (antibiotics, chlorhexidine) and full discussion with the care team.
• In summary: For this 55-year-old patient, the most likely diagnosis is MRONJ secondary to IV zoledronic acid. Initial management should be conservative (chlorhexidine rinses, culture-directed antibiotics, pain control, minor debridement) with
immediate referral to an oral and maxillofacial surgeon and communication with the physician managing the bisphosphonate therapy. More invasive surgery is reserved for cases that fail to respond or progress.

15. In a 55-year-old patient with a history of radiotherapy, an unhealed area of exposed bone and pain develop weeks after extraction of tooth 38. Which complication is this associated with, and how should it be managed?

• Based on the clinical presentation you've described, this is highly suggestive of osteoradionecrosis (ORN) of the mandible.
• Summary of the Case and Complication
• Patient Profile: 55-year-old with a history of radiotherapy (almost certainly for head and neck cancer).
• Trigger: Dental extraction (tooth 38, the lower left third molar).
• Timeframe: Symptoms develop weeks after extraction (a typical latent period).
• Key Symptoms:
o Unhealed area of exposed bone (for more than 3-4 weeks).
o Pain.
o The bone is likely visible in the socket and may be necrotic.
• This classic triad—prior radiotherapy, a traumatic event (extraction), and non-healing exposed bone—is diagnostic for ORN.
• Pathophysiology
• Radiation therapy damages the blood vessels (causing hypovascularity) and bone cells (osteocytes) in the irradiated field. This impairs the bone's ability to repair itself and resist infection. A subsequent insult, like a dental extraction, can lead to necrosis as the bone cannot mount an adequate healing response.
• Management of Osteoradionecrosis
• Management is staged, starting with conservative measures and escalating based on severity and response. The goals are to control symptoms, prevent progression, and manage infection.
• 1. Conservative/Medical Management (Initial approach for early-stage ORN):
• Oral Hygiene & Local Wound Care: Gentle irrigation with saline or chlorhexidine rinses. Debridement of superficial sequestra in the clinic.
• Antibiotics: Used to treat secondary infection (e.g., penicillin-based or clindamycin). They treat the superimposed infection but do not reverse the necrosis itself.
• Analgesia: For pain control.
• Pentoxifylline and Tocopherol (Vitamin E) (PENTO Protocol): This is a cornerstone of modern medical management. Pentoxifylline improves blood flow and reduces fibrosis, while tocopherol acts as an antioxidant. This combination has been shown to promote healing in some cases of ORN.
• Ultrasound Therapy (Low-intensity pulsed ultrasound): May stimulate bone healing in some cases.
• 2. Hyperbaric Oxygen Therapy (HBOT):
• Traditionally used, but its role is now controversial. It aims to increase tissue oxygenation and enhance angiogenesis.
• Current guidelines (e.g., from the M.D. Anderson Cancer Center) suggest it is not recommended as a primary treatment due to mixed evidence and risk of failure. It may be considered in refractory cases or as part of a combined protocol, but it is no longer the first-line standard.
• 3. Surgical Management:
• Indicated for advanced disease (severe pain, pathologic fracture, orocutaneous fistula), or when conservative treatment fails.
• Conservative surgery: Sequestrectomy (removal of the dead bone fragment) and local debridement.

• Radical surgery: Segmental resection with immediate reconstruction using a vascularized free flap (e.g., fibula free flap). This is the definitive treatment for advanced ORN, as it removes all necrotic, hypoxic bone and replaces it with healthy, well-vascularized tissue, which is crucial in an irradiated field.
• 4. Prevention (The Most Critical Step):
• This case underscores the paramount importance of pre-radiation dental evaluation.
• Ideally, all non-restorable teeth in the radiation field should be extracted at least 2-3 weeks before radiotherapy begins, allowing for primary healing in a non-irradiated environment.
• Meticulous lifelong oral care and regular dental follow-ups are essential post-radiation. Extractions should be avoided if possible; if necessary, they should be performed with minimal trauma, possibly with prophylactic antibiotics, and ideally in consultation with an oral oncologist or oral surgeon experienced in managing irradiated patients.
• Conclusion
• The patient is likely suffering from osteoradionecrosis of the mandible. Management should begin with:
• A detailed clinical and imaging assessment (likely a CBCT or CT scan) to determine the extent of bone involvement.
• Initiation of conservative medical management, notably the PENTO protocol (Pentoxifylline & Tocopherol), along with local wound care and antibiotics for infection.
• Referral to a multidisciplinary team specializing in head and neck oncology, typically involving an oral and maxillofacial surgeon, a radiation oncologist, and a medical oncologist. Surgical resection with vascularized free flap reconstruction is the definitive treatment for advanced or non-responsive cases.

16. In a 62-year-old patient taking warfarin, a mandibular right 48 extraction is performed without checking INR, followed by uncontrolled bleeding. What mechanism explains this bleeding, and what treatment should be applied?

• This scenario describes a high-risk situation with a clear etiology and specific management needs.
• Mechanism of Bleeding
• The bleeding is primarily due to the pharmacological effect of warfarin.
• Warfarin's Action: Warfarin is a vitamin K antagonist. It inhibits the synthesis of the active forms of vitamin K-dependent clotting factors (II, VII, IX, and X) in the liver.
• INR Significance: The International Normalized Ratio (INR) is the standardized measure of the extrinsic and common coagulation pathways. It is the essential tool for monitoring warfarin's anticoagulant effect. Not checking it prior to an invasive procedure is a critical error.
• Consequence: Without knowing the INR, the extraction was likely performed while the patient was in a therapeutic or potentially supratherapeutic anticoagulated state. This impaired coagulation system cannot form a stable clot at the surgical site, leading to uncontrolled, persistent bleeding.
• Underlying Patient Factor: The tooth involved (mandibular right 48, or lower right third molar) often has a deep, bony socket with a rich vascular supply, which can complicate bleeding control even in non-anticoagulated patients.
• Treatment (Immediate Local & Supportive Measures)
• The goal is to achieve local hemostasis without prematurely reversing the systemic anticoagulation, which could pose a thrombotic risk. Reversal is considered only for life-threatening bleeding.
• Step 1: Initial Assessment & Stabilization
• Assess vital signs (for signs of hypovolemia).
• Initiate IV access, consider fluid resuscitation if bleeding is severe.
• Draw blood for immediate INR, CBC, and type & screen (in case transfusion is needed).
• Step 2: Aggressive Local Hemostatic Measures These are the first-line and most critical interventions:
• Mechanical Pressure: Pack the socket with gauze soaked in tranexamic acid (an antifibrinolytic) and have the patient bite down firmly for 30-60 minutes.
• Topical Hemostatic Agents: After gentle debridement, pack the socket with a hemostatic agent:
o Absorbable gelatin sponge (Gelfoam®) soaked in tranexamic acid or saline.
o Oxidized cellulose (Surgicel®).
o Collagen-based sponges (CollaPlug®, CollaTape®).
o Fibrin sealant or hemostatic putty if available.
• Suturing: Place multiple, interrupted, non-resorbable sutures (e.g., 3-0 or 4-0 silk)

under tension to achieve primary closure over the packed socket. This compresses the socket and stabilizes the clot/hemostatic material. A "figure-of-eight" suture across the socket is often very effective.
• Re-evaluate: Apply sustained pressure again after suturing. If bleeding persists through the sutures, pack more agent and resuture.
• Step 3: Pharmacological Adjuncts
• Tranexamic Acid: In addition to topical use, administer systemically (e.g., 1g orally every 8 hours for 3-5 days) to inhibit clot breakdown.
• Warfarin Management (In Consultation with Physician/Cardiologist):
o Do NOT stop warfarin abruptly. The decision to hold or adjust the dose depends on the INR result and the patient's thromboembolic risk (e.g., mechanical heart valve, atrial fibrillation).
o For most cases of localized oral bleeding, achieving local control allows the patient to continue their usual warfarin dose. The INR may be checked and a single dose may be held or reduced, but full reversal is rarely indicated.
• Step 4: Systemic Reversal (Only for Severe, Uncontrollable Bleeding) If the above measures fail and bleeding is life-threatening:
• Vitamin K (Phytonadione): 1-5 mg IV or orally (IV works faster but carries anaphylaxis risk). This provides reversal over 6-24 hours.
• Prothrombin Complex Concentrate (PCC): This is the fastest and preferred agent for emergency reversal (works within 15 minutes). It contains concentrated factors II, VII, IX, and X. The dose is INR-guided.
• Fresh Frozen Plasma (FFP): An alternative if PCC is unavailable, but requires larger volumes, carries transfusion risks, and acts more slowly.
• Step 5: Post-operative Instructions & Monitoring
• Strict Instructions: Soft diet, cold compresses, avoid rinsing/spitting/straws for 24-48 hours, strict avoidance of NSAIDs.
• Close Follow-up: Re-evaluate in 24-48 hours. Provide detailed instructions on signs of re-bleeding.
• Communication: Immediately consult or refer to the patient's physician/cardiologist and/or a hospital emergency department for coordinated management of anticoagulation. A hematology consult may also be warranted.
• Key Takeaway
• The bleeding is a direct result of an unmonitored warfarin effect. The cornerstone of treatment is local surgical hemostasis (mechanical packing, hemostatic agents, and secure suturing) combined with tranexamic acid. Systemic reversal of anticoagulation is reserved for severe, refractory cases and must be done in consultation with a physician to balance bleeding and thrombotic risks. This patient requires urgent management and specialist coordination.

17. In a 30-year-old patient, after mandibular 48 extraction, painful oral erosions and cutaneous target lesions develop following use of ibuprofen and amoxicillin. Which drug reaction does this suggest, and how should it be treated?

• Based on the presentation you've described, this strongly suggests Stevens-Johnson Syndrome (SJS) or Toxic Epidermal Necrolysis (TEN), which is a severe, life-threatening drug reaction.
• Here's the breakdown:
• 1. Diagnosis: Stevens-Johnson Syndrome / Toxic Epidermal Necrolysis (SJS/TEN)
• Typical Triggers: The onset following the use of ibuprofen (an NSAID) and amoxicillin (an antibiotic) is classic. Both are well-known, common triggers for SJS/TEN.
• Key Symptoms:
o Painful oral erosions: Severe mucosal involvement (mouth, sometimes eyes, genitals) is a hallmark.
o Cutaneous target lesions: These are not the classic "targets" of erythema multiforme (which are raised and paler in the center). In SJS/TEN, the lesions are typically atypical target lesions (with only two zones of color or poorly defined) that quickly progress to blisters and widespread epidermal detachment.
o Temporal Association: The reaction typically begins 1-3 weeks after starting the culprit drug.
• 2. Immediate Actions & Treatment
• This is a medical emergency. The patient requires immediate hospitalization, typically in an Intensive Care Unit (ICU) or a specialized Burn Unit.
• Treatment Principles:

• Immediate Drug Withdrawal: Stop ibuprofen, amoxicillin, and ALL other non-essential medications immediately. Identifying and removing the culprit is the single most important step.
• Supportive Care (The Mainstay of Therapy): This is analogous to treating a major burn.
o Fluid & Electrolyte Management: Careful IV fluid resuscitation to prevent hypovolemia, while avoiding fluid overload.
o Wound Care: Sterile handling, protective non-adhesive dressings for denuded skin.
o Pain Management: Severe pain requires strong IV analgesia (often opioids).
o Nutritional Support: Nasogastric or parenteral nutrition due to severe oral involvement.
o Infection Prophylaxis: Aseptic technique and monitoring for sepsis (the leading cause of death). Prophylactic systemic antibiotics are controversial and not routinely given unless there is clear evidence of infection.
o Ophthalmic & Mucosal Care: Critical to prevent long-term sequelae. Frequent ophthalmology consultations and mucosal care (e.g., mouth rinses).
• Specific Medical Therapies (Controversial, but often considered):
o Systemic Corticosteroids: Their use is debated. High-dose pulses early in the disease may halt progression but carry risks of infection and sepsis. Many centers have moved away from them.
o Intravenous Immunoglobulin (IVIG): Often used as it may block the Fas-Fas ligand mediated apoptosis that causes keratinocyte death.
o Anti-TNF agents (e.g., Etanercept): Emerging as a promising therapy that may be more effective than IVIG.
o Cyclosporine: Some protocols use it for its immunosuppressive and anti-apoptotic effects.
• 3. What it is NOT (Differential Diagnosis)
• Erythema Multiforme Major: Can look similar with targets and oral erosions, but is often milder, frequently associated with herpes simplex or *Mycoplasma* infections (not drugs), and has less widespread skin detachment.
• Acute Generalized Exanthematous Pustulosis (AGEP): Presents with widespread sterile pustules, not target lesions or severe erosions.
• Drug Reaction with Eosinophilia and Systemic Symptoms (DRESS): Presents with a diffuse rash, high fever, lymphadenopathy, and internal organ involvement (hepatitis, nephritis), typically 2-8 weeks after drug start. The "target lesions" and rapid progression to erosions are less characteristic of DRESS.
• Summary of Key Points:
• Diagnosis: SJS/TEN triggered by ibuprofen, amoxicillin, or both.
• Action: Immediate emergency hospitalization (ICU/Burn Unit).
• Treatment Cornerstones: 1) Immediate cessation of all suspect drugs, 2) Aggressive supportive care, 3) Consideration of specific immunomodulatory therapy (IVIG, etanercept) in consultation with a dermatologist and intensive care specialist.
• Disclaimer: *This information is for educational purposes. The management of SJS/TEN is complex and must be carried out by a specialized medical team.*

18. In a 27-year-old patient, during surgical extraction of an impacted tooth 28, a portion of the maxillary tuberosity fractures and separates along with the tooth. What surgical and postoperative management should be performed?

• Based on the scenario you've described, here is the surgical and postoperative management that should be performed. This situation is a fracture of the maxillary tuberosity during extraction of an impacted third molar (tooth 28, likely using the FDI numbering system, which is the left maxillary third molar).
• Immediate Surgical Management (Intraoperative)
• The primary goals are to preserve vital structures (especially the maxillary sinus), achieve hemostasis, and stabilize the bone fragment to allow healing.
• Immediate Assessment & Decision:
o Evaluate the Fracture: Determine the size of the bony fragment, its relationship to the impacted tooth, and its soft tissue attachment (pedicle). A fragment with intact periosteal attachment has a much better blood supply and chance of survival.
o Check for Sinus Communication: Gently probe the area to see if the fracture has created an oroantral communication (OAC) into the maxillary sinus. Palpate the posterior wall of the maxillary tuberosity. A significant fracture can compromise

the sinus floor.
o Assess Fragment Viability: If the fragment is large, well-vascularized, and can be repositioned, preservation is the goal.
• Surgical Repair Strategy:
o If the Fragment is Viable and Stable:
▪ Reposition and Stabilize: Carefully reposition the bony fragment into its anatomical position.
▪ Suture Stabilization: Use interrupted or horizontal mattress sutures to re-approximate the gingiva and periosteum over the bone, providing soft tissue stabilization. A "figure-of-eight" suture over the socket can help.
▪ Consider Splinting: For a very large or mobile fragment, a small bone screw or a suturing technique that anchors to more stable bone (e.g., the zygomatic buttress) may be necessary. In some cases, a prefabricated or acrylic splint may be used to stabilize the area.
o If the Fragment is Non-Viable (detached, small, or necrotic):
▪ Remove the Fragment: Gently detach and remove it. This is often necessary if it is completely detached from soft tissue.
▪ Manage the Resultant Defect: The key is to achieve primary closure over the bony defect. This may require:
• Mobilization of a Flap: A buccal advancement flap or a palatal flap may be needed to achieve tension-free closure over the socket and any potential sinus communication.
• Socket Debridement: Carefully curette the socket to remove debris but preserve healthy bone.
• Management of Potential Oroantral Communication (OAC):
o This is a critical step. If an OAC is present or suspected:
▪ Size Assessment: If smaller than 2-3mm, it may close spontaneously with careful soft tissue approximation and a blood clot.
▪ Larger Communication: Requires active management to prevent an oroantral fistula (OAF). This typically involves:
• Creating a Buccal Advancement Flap: The most common method. A periosteal releasing incision allows the buccal flap to be advanced over the communication without tension.
• Consider a Palatal Rotational Flap: For larger defects or if the buccal tissue is inadequate.
▪ Suture with Care: Use resorbable sutures (e.g., 4-0 or 5-0 Vicryl) to achieve a watertight, primary closure.
• Hemostasis: Achieve hemostasis with pressure, local hemostatic agents (e.g., gelatin sponge, oxidized cellulose), and secure suturing.
• Immediate Postoperative Management
• Immediate Instructions:
o Bite on Gauze: Apply firm, continuous pressure on a gauze pack for 45-60 minutes to stabilize the clot and fragment.
o Avoid Disruption: STRICTLY PROHIBIT nose blowing, sneezing with a closed mouth, sucking through a straw, or spitting for at least 7-10 days. These actions create negative pressure in the sinus, which can disrupt the healing site and open a communication. Advise to sneeze with the mouth open.
o Medications:
▪ Analgesics: Prescribe appropriate pain medication (e.g., NSAIDs).
▪ Antibiotics: Mandatory. Prescribe a broad-spectrum antibiotic (e.g., amoxicillin-clavulanate or clindamycin if allergic) for 5-7 days to prevent sinus infection and osteitis.
▪ Decongestants/Nasal Spray: Prescribe a nasal decongestant spray (e.g., oxymetazoline) to use for 3-5 days. This reduces sinus edema and congestion, promoting drainage away from the surgical site.
• Postoperative Follow-up and Home Care
• Diet:
o Soft, Cool Diet for the first week. Avoid hot foods/liquids initially.
o Chew on the opposite side.
o Maintain good hydration.
• Oral Hygiene:
o Avoid rinsing or brushing the surgical site for the first 24 hours.

o After 24 hours, begin gentle rinsing with warm salt water or a prescribed chlorhexidine mouthwash after meals to keep the area clean.
o Brush other teeth normally, carefully avoiding the surgical site.
• Activity:
o Avoid strenuous activity and heavy lifting for at least one week to minimize bleeding and edema risk.
• Monitoring and Management of Complications
• Signs of Infection or Sinusitis: Instruct the patient to watch for and report:
o Persistent or foul-tasting nasal discharge (especially unilateral).
o Fever, increased pain/swelling after 3-4 days.
o Air/fluid passing from the nose to the mouth.
• Signs of Failed Healing/Oroantral Fistula (OAF):
o Persistent communication or a "whistling" sound when breathing through the nose while pinching it.
o Recurrent sinus infections.
• Follow-up:
o Schedule a 24-48 hour post-op check to assess healing, soft tissue integrity, and signs of infection.
o A 1-week follow-up is crucial to evaluate for any persistent OAC.
o If an OAC persists beyond 2-3 weeks, referral to an oral and maxillofacial surgeon for definitive closure (e.g., with a buccal fat pad graft or other more advanced techniques) is likely necessary.
• In summary, the key principles are: Immediate stabilization of the viable fragment or its removal, meticulous closure to prevent an oroantral fistula, aggressive antibiotic and decongestant therapy, and strict patient compliance to avoid negative sinus pressure. Given the complexity, this complication often warrants a prompt consultation with or referral to an oral and maxillofacial surgeon at the time of the event, if the operating dentist is not a specialist.

19. In a 29-year-old patient, several weeks after extraction of tooth 18, unilateral nasal obstruction, foul-smelling yellow-green discharge, and facial pain develop. Which complication does this suggest, and how should it be managed?

• Based on the presentation you've described, this is highly suggestive of acute maxillary sinusitis of odontogenic origin, specifically a complication following the extraction of tooth 18 (mandibular left second molar).
However, there is an important anatomical point to clarify:
• Tooth 18 (using the Universal numbering system) is the mandibular left second molar, which is in the lower jaw.
• Odontogenic sinusitis more commonly arises from maxillary teeth (premolars and molars), because their roots are in close relation to the maxillary sinus floor.
• If the history truly states "extraction of tooth 18" and symptoms are of maxillary sinusitis, this could be due to:
o Possible confusion in tooth numbering — perhaps the extracted tooth was actually a maxillary molar (e.g., tooth 14 or 15 if on the left side in universal numbering).
o Rarely, a mandibular infection could spread to the maxillary sinus indirectly, but much less likely.
Given the classic symptoms unilateral nasal obstruction, foul-smelling yellow-green discharge, and facial pain occurring weeks after extraction, the most likely diagnosis is:
Oro-antral communication (OAC) leading to chronic oroantral fistula (OAF) with secondary acute maxillary sinusitis.
Pathophysiology:
During extraction of a maxillary posterior tooth (especially first or second molar), the thin bone between the tooth root and the sinus floor may be perforated, creating an oroantral communication. If not recognized/closed initially, it can persist → bacteria and oral fluids enter the sinus → sinus infection, often with foul discharge due to anaerobic oral flora.
Management Approach:
1. Immediate assessment:
a. Clinical exam: Check for OAF by having patient pinch nose and gently blow while observing the extraction site for air/fluid passage. b. Nasal endoscopy: To assess middle meatus, look for purulent discharge.
c. Imaging: Cone-beam CT (CBCT) or CT of sinuses to confirm sinusitis, assess size of OAC/OAF, and evaluate for foreign bodies (e.g., displaced root fragment, bone spicule) or sinus polyps.
2. Initial management of acute infection:
a. Antibiotics: Broad-spectrum covering oral anaerobes and sinus pathogens (e.g.,

amoxicillin-clavulanate or clindamycin + third-gen cephalosporin if penicillin allergic).
b. Nasal decongestants (oxymetazoline short-term) and saline nasal irrigation to improve sinus drainage. c. Analgesics for facial pain.
3. Definitive management of OAC/OAF:
a. If acute sinusitis is present, treat infection first before surgical closure of fistula. b. Surgical closure of oroantral fistula:
i. Small recent OAC may close spontaneously with help of a palatal or buccal advancement flap. ii. Established OAF (>3–4 weeks) requires surgical closure (e.g., buccal fat pad flap, buccal advancement flap, palatal rotational flap). c. Concurrent sinus surgery: If sinusitis is refractory or there is mucosal disease, may need Caldwell-Luc approach or endoscopic middle meatal antrostomy to clean the sinus and improve drainage before or during fistula repair.
4. Remove any antral foreign body if seen on imaging.
Final answer:
Complication: Oroantral fistula with secondary acute odontogenic maxillary sinusitis.
Management: Control acute sinusitis with antibiotics/decongestants, then surgical closure of fistula ± endoscopic sinus surgery for sinus drainage.

20. In a 24-year-old patient, during difficult extraction of a deeply impacted 48, the tooth slips from the elevator and is lost intraorally, followed by coughing and a foreign-body sensation in the throat. What condition does this most strongly suggest, and how should it be managed?

• Based on the clinical scenario, this most strongly suggests aspiration or ingestion of the tooth into the respiratory or digestive tract.
• Most Likely Condition:
• Aspiration of the tooth into the airway is the primary and most urgent concern, given the sequence of events:
• Loss of the tooth intraorally → likely fell into the oropharynx.
• Coughing → reflex response to airway irritation.
• Foreign-body sensation in the throat → suggestive of material passing through or lodged in the laryngopharynx or upper esophagus.
• The coughing and foreign-body sensation point strongly toward entry into the larynx or trachea (aspiration), though swallowing (ingestion) is also possible.
• Immediate Management:
• 1. Immediate Assessment & Stabilization:
• Keep the patient upright if coughing spontaneously; do not interfere if they are effectively coughing.
• If coughing is ineffective or if signs of airway obstruction develop (stridor, cyanosis, severe dyspnea), follow airway obstruction protocols (Heimlich maneuver/abdominal thrusts if conscious; CPR if unconscious).
• 2. Do NOT attempt blind finger sweeps in the mouth, as this may push the object deeper.
• 3. Urgent Radiographic Localization:
• First-line imaging: Chest X-ray (PA and lateral) and Abdominal X-ray (KUB) to locate the tooth.
• A tooth is radiopaque and should be visible.
• If in the chest (aspiration): Usually seen in the right main bronchus (more vertical). Bronchoscopy for removal is mandatory due to risk of pneumonia, atelectasis, or lung abscess.
• If in the abdomen (ingestion): Usually passes through the GI tract unevently. Monitor with serial X-rays if needed. Surgery is rarely required unless signs of perforation or obstruction develop.
• 4. Referral/Specialist Involvement:
• Aspiration confirmed/suspected: Urgent referral to pulmonology or thoracic surgery for bronchoscopy.
• Ingestion confirmed: Refer to general surgery/GI if tooth is lodged in esophagus (requires endoscopic removal) or if it fails to pass.
• If initial X-rays are negative but symptoms persist: Consider CT scan for better localization.
• 5. Documentation & Follow-up:
• Document the event thoroughly in the medical record.
• Inform the patient and family.
• Provide clear discharge instructions (if sent home after ingestion) to report any abdominal pain, fever, or difficulty breathing immediately.
• Key Takeaway:

• Given the coughing and foreign-body sensation, aspiration is the leading concern until proven otherwise. The management is urgent imaging to locate the tooth, followed by appropriate endoscopic retrieval if in the airway or esophagus, with careful monitoring if in the stomach/bowels.

21. In a 27-year-old patient, during an inferior alveolar nerve block for mandibular right 48, the needle fractures and is lost within the pterygomandibular space. By what mechanism can this occur, and how should it be treated?

• This is a serious but fortunately rare complication. Let's break down the mechanism and management.
• 1. Mechanism of Needle Fracture and Loss
• The needle most likely fractures and is retained in the pterygomandibular space due to:
• Sudden unexpected movement by the patient: If the patient jerks or closes their mouth forcefully while the needle is inserted, the hub can bend or break at the shaft-hub junction.
• Pre-existing needle damage: Reuse of disposable needles or repeated bending/sterilization can cause metal fatigue.
• Improper insertion technique: Inserting the needle up to the hub increases the risk of breakage at its weakest point (the hub-shaft junction) if the patient moves.
• Deflective maneuver against hard tissue: If the needle is deflected off the medial surface of the ramus, lateral pterygoid plate, or other bony structures, and the dentist attempts to redirect without withdrawing slightly, undue stress can cause fracture.
• Manufacturing defect: Rare, but possible.
• In an inferior alveolar nerve block for the mandibular right third molar (48), the needle is inserted into the pterygomandibular space medial to the ramus and lateral to the medial pterygoid muscle. If it breaks, the fragment will be embedded in the loose connective tissue, muscles (medial pterygoid, maybe part of lateral pterygoid), or near neurovascular structures (inferior alveolar nerve, lingual nerve, inferior alveolar vessels).
• 2. Immediate Management at the Time of Incident
• Stay calm and instruct the patient not to move.
• Do not remove the syringe if the hub is still attached — it can help locate the fragment.
• If the needle is completely lost from sight, do not attempt blind exploration with instruments; this may push it deeper or cause injury.
• Keep the mouth open with a bite block to prevent further movement.
• Reassure the patient and explain what happened.
• 3. Radiographic Localization
• The priority is to locate the fragment before attempting removal.
• First radiograph: Usually a lateral oblique view of the mandible can show the needle in the pterygomandibular space.
• More precise imaging: CT scan (axial and coronal views) is the gold standard — it shows the exact 3D position relative to vital structures (inferior alveolar nerve, blood vessels, muscles, parotid gland).
• Panoramic radiograph may help but is less precise for depth localization.
• 4. Surgical Removal
• Refer urgently to an oral and maxillofacial surgeon.
• Removal is typically done under general anesthesia in a hospital setting.
• Using the CT images for guidance, the surgeon will plan an intraoral (or rarely extraoral) approach.
• The fragment is approached through the pterygomandibular space via an incision in the retromolar region / medial to the ramus, sometimes with blunt dissection along the original needle path.
• Intraoperative fluoroscopy or C-arm imaging may be used to guide removal.
• Care is taken to avoid damaging the inferior alveolar nerve, lingual nerve, and maxillary artery branches.
• 5. Possible Complications If Left Untreated
• Migration of the needle fragment (rare due to tissue encapsulation, but possible with muscle movement).
• Infection or abscess formation.
• Chronic pain or neuropathy due to irritation of nerves.
• Risk of penetration into deeper spaces (infratemporal fossa, parapharyngeal space).
• Psychological distress to the patient.
• 6. Prevention
• Use new, disposable needles for each patient.
• Do not insert the needle to the hub — leave at least 5–10 mm exposed.

• Warn the patient before injection to avoid sudden movement.
• Avoid bending the needle intentionally.
• Use longer needles (e.g., 35 mm) for inferior alveolar blocks to avoid full insertion.
• Summary of Treatment Plan
• Immediate stabilization of patient and needle if partially visible.
• Radiographic localization with CT scan.
• Referral to oral and maxillofacial surgeon.
• Surgical removal under general anesthesia with image guidance.
• Postoperative monitoring for infection or nerve injury.
• Documentation and incident reporting per clinical guidelines.
• Counseling and follow-up for the patient.
• In this 27-year-old patient, the prognosis is generally good if the fragment is promptly and properly removed.

22. In a 19-year-old patient, during impacted 48 surgery, cyanosis, fatigue, and low oxygen saturation develop after administration of prilocaine and articaine. What does this indicate, and how should it be treated?

• Based on the description, this scenario is highly concerning for methemoglobinemia, a known potential adverse reaction to prilocaine (and, to a lesser extent, articaine).
• What This Indicates:
• The combination of cyanosis unresponsive to oxygen, fatigue, and low oxygen saturation (SpO2) appearing shortly after administration of prilocaine (a classic culprit) strongly points to drug-induced methemoglobinemia.
• Mechanism: Prilocaine (and its metabolite ortho-toluidine) can oxidize the iron in hemoglobin from the ferrous (Fe^{2}) state to the ferric (Fe^{3}) state, forming methemoglobin.
+ + Methemoglobin cannot bind and transport oxygen effectively.
• Key Clinical Sign: The patient will appear cyanotic (often a slate-gray or bluish color) and have a low SpO2 reading (typically around 85%, but can be lower), but this hypoxemia does NOT improve with supplemental oxygen. This is a critical distinguishing feature from respiratory depression or pulmonary issues.
• Fatigue is a direct result of impaired oxygen delivery to tissues.
• How This Should Be Treated:
• Immediate Actions in the Dental/Surgical Setting:
• Recognize and Stop the Insult: Immediately stop administration of any further local anesthetic.
• Secure the Airway and Support Breathing: Ensure a patent airway. Administer 100% oxygen via a non-rebreather mask. While oxygen alone will not reverse methemoglobinemia, it maximizes the oxygen-carrying capacity of the remaining normal hemoglobin and aids in tissue oxygenation.
• Call for Emergency Medical Services (EMS): This is a medical emergency requiring hospital management. Transport to an emergency department is mandatory.
• Monitor: Continuously monitor vital signs, oxygen saturation (via pulse oximetry, recognizing its limitations in this condition), and heart rhythm.
• Definitive Treatment in the Hospital (Emergency Department):
• The cornerstone of treatment for significant symptomatic methemoglobinemia is the administration of methylene blue.
• Confirmation: Diagnosis is confirmed by co-oximetry on an arterial blood gas (ABG) sample. Standard pulse oximeters are unreliable in methemoglobinemia and will show a falsely low SpO2. Co-oximetry directly measures the percentage of methemoglobin (MetHb%).
• Methylene Blue Administration:
o Indication: Usually given for symptomatic patients or with MetHb >20-30% (lower threshold for patients with comorbidities like anemia or cardiac disease). A 19-year-old with symptoms clearly meets the criteria.
o Dose: 1-2 mg/kg of a 1% solution, given by slow IV push over 5 minutes.
o Mechanism: Acts as a co-factor to accelerate the enzymatic reduction of methemoglobin back to normal hemoglobin.
o Response: Improvement in cyanosis and symptoms is usually rapid (within minutes to an hour).
• Supportive Care: Continue oxygen. IV fluids. Monitor for recurrence, as the effect of methylene blue can be transient.
• Contraindications & Cautions: Methylene blue is contraindicated in patients with known G6PD deficiency, as it can cause severe hemolysis. In G6PD-deficient patients,

alternative treatments like ascorbic acid (vitamin C) or even exchange transfusion in life-threatening cases may be considered.
• Summary for the Clinician:
• You are likely seeing prilocaine-induced methemoglobinemia. The urgent priorities are: 1) Maintain airway and give 100% O2, 2) Activate EMS for immediate transfer to a hospital, and 3) Inform the receiving emergency department of your strong suspicion for methemoglobinemia following prilocaine administration, so they can prepare for co-oximetry and methylene blue.
• Disclaimer: *This information is for educational purposes. Actual management must be carried out by qualified healthcare professionals on the scene and in a hospital setting.*

23. In a 45-year-old patient with uncontrolled diabetes, increasing pain, fistula formation, foul odor, and sequestrum formation develop a few weeks after extraction of mandibular left 37 and 38. What condition does this suggest, and how should it be treated?

• Based on the clinical presentation, this suggests osteomyelitis of the mandible.
• Here's a breakdown of the reasoning and the appropriate management:
• Why This is Osteomyelitis
• Patient Profile: A 45-year-old with uncontrolled diabetes is at high risk for impaired wound healing and infection due to microvascular complications and immune dysfunction.
• Precipitating Event: Recent extraction of mandibular molars (37, 38) provides a portal for infection into the bone.
• Key Clinical Features:
o Increasing pain beyond normal post-extraction healing.
o Fistula formation (a sinus tract draining pus).
o Foul odor (indicative of anaerobic infection and necrotic tissue).
o Sequestrum formation (a piece of dead, separated bone)—a pathognomonic sign of osteomyelitis.
o The timeline of "a few weeks" aligns with subacute or chronic osteomyelitis development.
• Recommended Treatment Approach
• Management must be aggressive and multidisciplinary, given the uncontrolled diabetes and signs of chronic infection.
• 1. Immediate Diagnostic Work-Up
• Imaging:
o Panoramic radiograph (OPG) to assess bone destruction, sclerosis, and sequestrum.
o CBCT for detailed 3D evaluation of bone involvement, sequestra, and periosteal reaction.
o Consider MRI if soft tissue extension or early marrow changes are suspected.
• Lab Tests:
o CBC, ESR, CRP (will be elevated).
o Blood glucose/HbA1c to quantify diabetic control.
o Microbiology: Deep culture and sensitivity from fistula/debridement specimen (aerobic/anaerobic, including *Staphylococcus aureus*, *Streptococcus*, anaerobes like *Prevotella* and *Fusobacterium*).
• 2. Medical Management
• Optimize Diabetes Control: Crucial first step. Coordinate with physician/endocrinologist for strict glycemic control (insulin regimen likely needed).
• Long-term Antibiotics: Based on culture results. Empiric therapy (while awaiting cultures) often requires broad-spectrum coverage:
o First-line: A penicillin/beta-lactamase inhibitor (e.g., amoxicillin-clavulanate) plus metronidazole (for anaerobes).
o Penicillin-allergic or MRSA risk: Clindamycin, or vancomycin/linezolid in consultation.
o Duration: Typically 4–6 weeks of IV therapy initially, possibly transitioning to oral based on response. Chronic cases may need months.
• Analgesia: Appropriate pain management.
• 3. Surgical Management (Essential)
• Sequestrectomy & Surgical Debridement: Removal of all necrotic bone (sequestrum) and infected soft tissue until bleeding bone margins are reached.
• Saucerization: Removal of overlying bone to expose the infected area, allowing drainage

and preventing pus accumulation.
• Decortication: In refractory cases, removal of the affected cortical bone plate.
• Fistula Tract Excision: Remove the entire sinus tract.
• Ensure Adequate Drainage.
• 4. Adjunctive Therapies
• Hyperbaric Oxygen Therapy (HBOT): Consider if available, especially for refractory cases. Improves oxygenation, enhances neutrophil function, and promotes angiogenesis in hypoxic tissue.
• Local Antibiotic Delivery: Antibiotic-impregnated beads (e.g., gentamicin-PMMA) may be placed in the surgical site.
• 5. Long-term Follow-Up
• Regular clinical and radiographic review for resolution or recurrence.
• Maintenance of good glycemic control and oral hygiene.
• Possible need for reconstructive surgery (e.g., bone grafting) after infection is fully eradicated (often delayed for 6–12 months).
• Conclusion
• The patient is presenting with chronic osteomyelitis of the mandible, likely secondary to odontogenic infection in an immunocompromised host (uncontrolled diabetes).
Treatment is dual:
• Control the systemic disease (aggressive diabetic management).
• Aggressively treat the local infection with combined surgical debridement and long-term culture-guided antibiotics.
• Failure to control diabetes will severely compromise treatment success. Immediate referral to an oral & maxillofacial surgeon and coordination with the patient's physician are mandatory.

24. In a 23-year-old patient, after local anesthesia for tooth 28, it is learned that the patient has a history of drug allergy and atopic disease . Shortly after injection, generalized pruritus–urticaria, lip/face swelling, wheezing, and dizziness develop. How would you evaluate this presentation in terms of the most likely diagnosis, and how would you plan the emergency management in a dental clinic?

• Evaluation of Presentation:
• Given the clinical scenario – a young adult with known drug allergy/atopic history, with onset of generalized pruritus, urticaria, facial/lip angioedema, wheezing (lower respiratory involvement), and dizziness (suggestive of hypotension or systemic reaction) within minutes of a local anesthetic injection – this is highly consistent with a Type I IgE-mediated anaphylactic reaction.
• This is a medical emergency. The constellation of symptoms involving the skin (urticaria), respiratory system (wheezing, potential for upper airway edema from lip/face swelling), and possible cardiovascular system (dizziness) meets criteria for anaphylaxis. The rapid onset after a potential allergen exposure (local anesthetic or possibly another component, like a preservative) is classic.
• Emergency Management Plan in a Dental Clinic:
• Immediate action is required following the basic principle: Airway, Breathing, Circulation, Disability, Exposure (ABCDE).
• 1. Immediate Actions:
• Stop all dental treatment.
• Call for help. Activate the clinic's emergency protocol. Have another team member call emergency medical services (EMS / ambulance) immediately. State "suspected anaphylaxis."
• Position the patient: Place the patient supine (flat on their back). If the patient is experiencing significant breathing difficulty or is vomiting, they can be placed in a position of comfort, often sitting up slightly. Do not have them sit upright if they are dizzy or lightheaded, as this can worsen hypotension.
• Administer Epinephrine (Adrenaline) – FIRST LINE, MOST CRITICAL STEP.
o Drug: Epinephrine 1:1000 (1 mg/mL) solution.
o Dose: 0.3 - 0.5 mg intramuscularly. For a 23-year-old adult, 0.3 mg (0.3 mL) is a standard initial dose.
o Site: Mid-outer thigh (vastus lateralis) is preferred. It can be given through clothing.
o Rationale: Epinephrine reverses vasodilation, reduces edema, improves blood pressure, and relieves bronchospasm. It is life-saving. Delay is dangerous.
• 2. Supportive Measures & Secondary Medications:

• Airway & Breathing:
o Administer high-flow oxygen (10-15 L/min via non-rebreather mask).
o Monitor closely for progression of upper airway swelling. Prepare for possible advanced airway management by EMS.
o For bronchospasm (wheezing): Consider an inhaled beta-2 agonist like Salbutamol (Albuterol) via a spacer/mask if available and the patient can use it.
• Circulation:
o Establish IV access if trained personnel and equipment are available. Begin an IV crystalloid fluid bolus (e.g., 500-1000 mL normal saline) if hypotension is present or suspected (dizziness).
o Monitor vital signs (pulse, blood pressure) continuously.
• Adjunctive Medications (after epinephrine):
o Antihistamines: Administer an H1-antihistamine (e.g., Diphenhydramine 25-50 mg IM or slow IV) and consider an H2-antihistamine (e.g., Ranitidine 50 mg IV or Famotidine 20 mg IV).
o Corticosteroids: Administer a steroid to potentially reduce late-phase reactions (e.g., Hydrocortisone 200 mg IM/IV or Methylprednisolone 80-125 mg IV). This is secondary to epinephrine.
• 3. Monitoring & Preparation for Transfer:
• Reassess frequently. Symptoms may recur (biphasic reaction). Be prepared to give a second dose of epinephrine after 5-15 minutes if there is no improvement or if symptoms worsen.
• Keep the patient warm.
• Do not discharge the patient. All patients with anaphylaxis require emergency department evaluation and observation for several hours (typically 4-6 hours minimum, sometimes longer).
• Prepare to hand over to the EMS team with details: suspected trigger (local anesthetic for tooth 28), time of onset, all medications given (time and dose of epinephrine is critical), and the patient's response.
• 4. Post-Emergency Considerations (After Stabilization & Transfer):
• Documentation: Record the event meticulously in the patient's chart, including the sequence of symptoms, all interventions, and EMS activation.
• Allergist Referral: The patient must be referred to an allergist/immunologist for confirmatory testing to identify the specific causative agent (e.g., the local anesthetic itself, methylparaben preservative, or possibly sulfites in the vasoconstrictor). Do not simply assume it was the "caine" drug without testing. Alternative safe local anesthetics for future procedures can be identified.
• Medic Alert & Prescription: The patient should be prescribed an epinephrine auto-injector and advised to wear a medical alert bracelet.
• Key Points for the Dental Team:
• Anaphylaxis is a clinical diagnosis. Do not wait for full symptom progression.
• Epinephrine is the first and most important drug. There are no absolute contraindications to its use in anaphylaxis.
• The differential diagnosis includes vasovagal syncope (which typically presents with pallor, bradycardia, and recovery upon lying flat, and lacks urticaria, wheezing, or pruritus) and a panic attack. However, the presence of multi-system involvement (skin + respiratory +/- cardiovascular) decisively points to anaphylaxis.
• All dental clinics must have a stocked emergency kit, regularly checked, with staff trained in drills for managing anaphylaxis.

25. A 29-year-old patient presents two weeks after extraction of tooth 18 with the complaint that liquids consumed come out through the nose. CBCT shows an oroantral communication measuring 6 mm. What complication does this represent, and how should it be treated?

This presentation represents a persistent oroantral communication (OAC), likely evolving into an oroantral fistula (OAF) following extraction of the maxillary right third molar (18). The passage of liquids from the oral cavity into the nose is a characteristic clinical finding. Because the defect has persisted for two weeks and measures 6 mm, spontaneous closure is unlikely; communications larger than approximately 5 mm generally require surgical management. Treatment should first include assessment of the maxillary sinus for infection or sinusitis, using the clinical findings and CBCT. If sinus infection is present, it should be controlled before or concurrently with definitive closure, with appropriate medical therapy and restoration of sinus drainage. The margins of a persistent epithelialized tract should be excised before closure.

For a 6-mm posterior maxillary defect, definitive treatment is usually tension-free surgical closure with a well-vascularized local flap. A buccal advancement (Rehrmann) flap is a standard and predictable option for a defect of this size. Depending on the location, tissue availability, and need to preserve vestibular depth, a palatal rotational flap or buccal fat pad flap may also be considered. Postoperatively, the patient should receive sinus precautions: avoidance of nose blowing, sneezing with the mouth closed, smoking, use of straws, or other activities that increase sinonasal pressure. Analgesia and appropriate nasal management may be prescribed, while systemic antibiotics are indicated when sinus infection is present rather than solely because an OAC exists. Thus, the specialist diagnosis is a persistent 6-mm oroantral communication/OAF following extraction of tooth 18, requiring evaluation and treatment of any sinus pathology followed by tension-free surgical closure, most commonly with a buccal advancement flap.

26. In a 52-year-old patient with uncontrolled diabetes, five days after bilateral mandibular 38 and 48 extractions, tongue swelling, dyspnea, and "board-like" induration/swelling of the neck develop. Which life-threatening infection does this suggest, and what is the appropriate emergency management?

• Based on the clinical presentation, the most likely life-threatening infection is Ludwig's Angina.
• Here is the breakdown of the reasoning and the appropriate emergency management:
• Suggested Life-Threatening Infection: Ludwig's Angina
• Pathophysiology: This is a rapidly spreading, diffuse cellulitis of the submandibular, sublingual, and submental spaces (bilateral), not a confined abscess. The "board-like" induration is a classic descriptor.
• Key Clues in the Vignette:
o Source: Recent bilateral mandibular extractions (especially of third molars, which are in close proximity to the submandibular space).
o Host: Patient with uncontrolled diabetes (impaired immune response and wound healing).
o Time Course: Five days post-procedure (typical incubation period).
o Findings:
▪ Tongue swelling and elevation: Due to involvement of the sublingual space.
▪ Dyspnea: The most critical concern, caused by posterior and superior displacement of the tongue, leading to potential airway obstruction.
▪ "Board-like" induration/swelling of the neck: Characteristic of the brawny, non-fluctuant cellulitis spreading through fascial planes.
• Appropriate Emergency Management
• The primary threat is airway obstruction. Management follows a strict sequence:
• Immediate Airway Assessment and Securing the Airway (ABSOLUTE PRIORITY):
o Do not delay. Anticipate a difficult airway due to trismus, swelling, and anatomical distortion.
o Awake fiberoptic nasotracheal intubation is often the preferred method, as it allows the patient to maintain their own airway until the endotracheal tube is placed.
o Alternatives include video laryngoscopy or awake tracheostomy. Orotracheal intubation may be impossible and risky.
o Do not sedate or paralyze the patient before the airway is secured, as this can precipitate complete obstruction.
• Supportive Measures:
o High-flow oxygen while preparing for intubation.
o Establish IV access for fluids and medications.
o Monitor vitals closely (oxygen saturation, heart rate, blood pressure).
• Medical Management:
o Empiric, Broad-Spectrum IV Antibiotics: Must cover mixed oral flora (gram-positive, gram-negative, and anaerobes). A typical regimen includes:
▪ Ampicillin-Sulbactam OR
▪ Clindamycin plus a third-generation cephalosporin (e.g., ceftriaxone) OR
▪ Piperacillin-Tazobactam.
▪ Add vancomycin or linezolid if MRSA is suspected.
o Corticosteroids: IV dexamethasone may be used to reduce inflammatory swelling, but only after the airway is secured.
• Surgical Management:

o Emergent Surgical Consultation (Oral & Maxillofacial Surgery, ENT).
o Incision and Drainage: While Ludwig's angina is primarily a cellulitis, surgical exploration is often necessary to release pressure, drain any collections, and obtain cultures. It is not typically a single, drainable abscess.
• Adjunctive Care:
o Control of the patient's diabetes (IV insulin drip).
o Pain management.
o ICU admission for monitoring.
• In summary, for this patient: The diagnosis is Ludwig's Angina. The immediate, life-saving intervention is securing the airway via awake fiberoptic intubation or tracheostomy, followed by high-dose IV antibiotics and surgical consultation.
27. In a 22-year-old patient, after an inferior alveolar nerve (IAN) block administered for mandibular right 48, difficulty closing the eye and drooping of the corner of the mouth are observed. How can the cause of this finding be explained, and how should it be managed?
• Excellent question. This is a classic presentation of an uncommon complication following an inferior alveolar nerve (IAN) block. Let's break it down.
• Explanation of the Cause
• The findings—difficulty closing the eye and drooping of the corner of the mouth—are indicative of a temporary facial nerve (CN VII) palsy.
• Here is the anatomical explanation:
• Target of the Injection: The standard IAN block targets the inferior alveolar nerve just before it enters the mandibular foramen, which is located on the medial surface of the mandibular ramus. The needle pathway is typically posterior and medial to the mandible.
• Anatomical Variation (Key Factor): In some individuals, the parotid gland and the facial nerve trunk or its branches extend more anteriorly and inferiorly than usual. The facial nerve runs within the parotid gland.
• Mechanism of the Complication: If the parotid capsule is penetrated by the dental needle (which is traveling in a posterior-superior direction for the IAN block), local anesthetic can be deposited within the parotid parenchyma. The anesthetic then diffuses and bathes the facial nerve fibers, causing a temporary chemical neuropraxia (blockade of nerve conduction).
• Resulting Symptoms: The facial nerve controls the muscles of facial expression, including:
o Orbicularis oculi: Closing the eyelid (patient cannot close the eye tightly).
o Orbicularis oris & zygomaticus muscles: Elevating the corner of the mouth and smiling (resulting in drooping).
o Other potential signs include loss of forehead wrinkling, inability to puff out the cheek, and flattening of the nasolabial fold on the same side as the injection. Taste sensation (chorda tympani branch) and lacrimation may rarely be affected, but motor symptoms are most prominent.
• In summary: The cause is unintentional deposition of local anesthetic (likely lidocaine or articaine) into the parotid gland, leading to a transient facial nerve block. This is not a stroke (which would be unilateral upper *and* lower face weakness) and is almost always a benign, self-resolving condition.
• Management
• Management is entirely supportive and expectant, as the effects will wear off as the local anesthetic is metabolized.
• Immediate Reassurance (Most Important Step):
o Calmly and clearly explain to the patient what has happened. Tell them this is a known, rare, and temporary complication of the injection, similar to the numbing they feel in their lip, but affecting a different nerve.
o Emphasize that it is NOT a stroke and that normal function will return completely, usually within the duration of the dental anesthetic (2-4 hours). In rare cases, effects may linger for several more hours, but full recovery is expected within 24 hours at most.
• Eye Care (Critical Intervention):
o The inability to close the eye (lagophthalmos) is the most significant risk, as it can lead to corneal drying, irritation, or injury.
o Instill lubricating eye drops (artificial tears).
o Apply an eye patch or tape the eyelids shut (gently) if the procedure will continue and the patient cannot blink. Instruct the patient to manually blink and use drops frequently if no patch is applied.

o Advise the patient to keep the eye moist and protected until the blink reflex returns.
• Monitor and Wait:
o There is no antidote or specific treatment needed. The procedure can often continue as planned, provided the patient is comfortable and the eye is protected.
o Monitor the patient until signs of recovery begin.
• Documentation:
o Meticulously document the event in the patient's chart:
▪ The procedure (mandibular right IAN block for tooth 48).
▪ The unexpected symptoms observed.
▪ The explanation given to the patient (transient facial nerve effect).
▪ The management provided (reassurance, eye care).
▪ The patient's response and understanding.
• Follow-up:
o Inform the patient that if any unusual symptoms persist beyond 24 hours (which is exceedingly rare with modern local anesthetics), they should contact you. Almost invariably, they will call a few hours later to report that everything is back to normal.
• What NOT to do: Do not administer any further medications or injections. Do not attempt to "aspirate" or neutralize the anesthetic. Avoid causing undue alarm.
• Prognosis: Excellent. Complete resolution is the universal outcome as the local anesthetic effect subsides.

28. In a 27-year-old patient, within a few days after a difficult 28 extraction, temporomandibular joint clicking, locking, and pain develop. By what mechanism can this be explained, and how should it be managed?

• This scenario describes a classic presentation of acute temporomandibular joint (TMJ) dysfunction following a difficult lower molar extraction, likely due to trauma to the masticatory system.
• Mechanism (Explanation)
• Excessive and Prolonged Mouth Opening: A difficult surgical extraction often requires the patient to keep their mouth wide open for an extended period, far beyond the normal physiological range. This can lead to:
o Stretching and strain of the TMJ capsule, ligaments (e.g., temporomandibular ligament), and retrodiscal tissues.
o Subluxation or displacement of the articular disc. The condyle may translate excessively forward, and the disc may not recapture properly upon closing, leading to clicking (disc reduction) and locking (disc displacement without reduction).
o Muscle fatigue and spasm in the muscles of mastication (masseter, temporalis, lateral pterygoid), contributing to pain and dysfunction.
• Direct Trauma or Pressure: The surgeon may have applied significant force or pressure on the mandible (e.g., using a hand or instrument for counter-pressure) during the extraction, potentially causing microtrauma to the joint or altering joint mechanics.
• Altered Occlusion: Post-extraction, especially if there is swelling or if the opposing tooth now has no contact, there can be a subtle change in the patient's bite (occlusal alteration). This forces the masticatory system to find a new, often strained, rest position and path of closure, stressing the TMJ.
• Stress and Parafunctional Habits: The pain and anxiety associated with the procedure can lead to clenching or bruxism, further overloading the already traumatized joint and muscles.
• In summary: The primary mechanism is macro-trauma from hyperextension during the procedure, leading to acute disc displacement, capsulitis/synovitis, and myofascial pain, manifesting as clicking (disc displacement with reduction), intermittent locking, and pain.
• Management
• Management is conservative and supportive in this acute phase, as the condition often resolves with time and minimal intervention.
• Immediate/Initial Management (First 1-2 Weeks):
• Patient Education and Reassurance:
o Explain the likely cause (trauma from prolonged opening) and that it is a common, often self-limiting condition.
o Advise rest for the jaw: Soft diet, avoid wide yawning, singing, and chewing gum.
o Teach self-management techniques: Apply moist heat to the joint and muscle area

for 15-20 minutes several times a day to relax muscles and improve blood flow.
• Pharmacological Management:
o NSAIDs (e.g., Ibuprofen, Naproxen): First-line for pain and inflammation in the joint (synovitis/capsulitis).
o Muscle Relaxants (e.g., Cyclobenzaprine): Short-term use if significant muscle spasm is present.
o Analgesics (e.g., Acetaminophen) for pain control if NSAIDs are contraindicated.
• Occlusal Evaluation:
o Check for any obvious premature contact or shift in the bite. A minor adjustment (selective occlusal grinding) may be considered if a clear, sharp deflective contact is identified, but this is usually deferred until the acute swelling subsides.
• Physical Therapy Modalities:
o Gentle jaw exercises (after acute pain subsides) to restore normal range of motion. A physical therapist can teach specific techniques.
o Ultrasound or low-level laser therapy may help reduce pain and inflammation.
• If Symptoms Persist (>2-4 weeks):
• Occlusal Appliance Therapy: A well-adjusted stabilization splint (full-coverage, hard acrylic) is the gold standard dental intervention. It helps by:
o Distributing occlusal forces evenly.
o Reducing loading on the joint.
o Decreasing muscle activity.
o Providing a stable, reproducible jaw position.
• Referral to a Specialist:
o Oral and Maxillofacial Surgeon or Orofacial Pain/TMD Specialist for further evaluation if locking becomes persistent (closed lock) or pain is severe.
o Advanced imaging (MRI) may be requested to confirm disc position and rule out other pathology.
o In cases of persistent, painful disc displacement without reduction, interventions like arthrocentesis (joint lavage) can be highly effective to reduce inflammation and release adhesions.
• Management of Chronic Factors: Address any underlying parafunctional habits, stress, or postural issues.
• Key Takeaway:
• For this 27-year-old patient, the onset is clearly acute and trauma-induced. The prognosis is generally good. Management should start with rest, reassurance, anti-inflammatories, and soft diet. Most cases will resolve with these measures. If symptoms do not improve within a few weeks, progression to occlusal splint therapy and specialist referral is appropriate. Avoid invasive or irreversible treatments (e.g., major occlusal rehabilitation, surgery) in this initial acute phase.

29. In a 28-year-old patient, during mandibular left 38 surgery, prolonged use of a bur with inadequate water cooling results in a localized area of necrotic bone. Which complication is this consistent with, and how should it be managed?
• Based on the clinical scenario you've described, this is consistent with surgical bone necrosis due to thermal osteonecrosis.
• Explanation
• Mechanism: Prolonged, high-speed drilling without adequate water irrigation generates significant frictional heat. Bone is a poor conductor of heat, and temperatures exceeding 47°C (116.6°F) for one minute can cause irreversible protein denaturation, cell death (osteocytes), and necrosis of the bone.
• Localization: The necrotic bone acts as a non-viable, avascular sequestrum within the osteotomy site (e.g., socket, bone around the roots of tooth #38). This creates a focus for infection, impairs healing, and can lead to persistent pain, delayed healing, or a dry socket-like picture that doesn't resolve.
• Management
• Management is primarily conservative and supportive, aimed at removing the necrotic focus and promoting healing of the viable surrounding bone.
• 1. Initial/Intraoperative Recognition:
• Stop the procedure in the affected area.
• Copiously irrigate with sterile saline or cooled irrigant to lower the temperature.
• Gently debride any visibly charred, loose, or non-bleeding bone fragments with a curette or bur using copious irrigation.
• 2. Postoperative Management:

• Antibiotics: Prescribe a course of antibiotics (e.g., amoxicillin/clavulanate or clindamycin if penicillin-allergic) to prevent secondary infection of the necrotic bone.
• Analgesics: Provide adequate pain management (NSAIDs +/- adjuncts).
• Antiseptic Rinses: Instruct the patient to use chlorhexidine mouthwash (0.12%) twice daily to maintain oral hygiene and reduce microbial load.
• Close Monitoring: Schedule frequent follow-up appointments (e.g., weekly) to assess healing.
• 3. Management of Persistent Necrosis/Sequestrum:
• If the area fails to heal, becomes painful, or a sequestrum (a loose piece of dead bone) becomes evident:
• Minor Sequestrectomy: Under local anesthesia, gently elevate the overlying soft tissue and remove the loose necrotic bone fragment with a curette or forceps.
• Irrigation: Again, copiously irrigate the site.
• Primary Closure or Healing by Secondary Intention: The wound may be loosely sutured or allowed to granulate, depending on size.
• 4. Important Adjunctive Measures & Prevention:
• Patient Counseling: Explain the cause and management plan. Reinforce the importance of follow-up.
• Emphasize Prevention for Future Procedures: This complication underscores the critical importance of proper surgical technique:
o Always use sharp, new burs.
o Use copious, continuous sterile water/saline irrigation during any bone cutting.
o Use a light, intermittent "brush-stroke" technique with the bur, avoiding prolonged, continuous pressure on one spot.
o Use high-speed handpieces with integrated spray coolant.
• Differential Diagnosis to Consider
• Dry Socket (Alveolar Osteitis): More common, presents with severe pain 2-4 days post-op, but bone is typically *not* necrotic; the socket is empty and lacks a blood clot. Management is focused on pain control and irrigation.
• Osteomyelitis: A more severe, spreading infection of the bone marrow. Thermal necrosis can be a precipitating factor, but true osteomyelitis involves more systemic signs, radiographic changes (moth-eaten appearance), and requires more aggressive, long-term antibiotic therapy and surgical debridement.
• Nerve Injury: Prolonged heat can also damage the inferior alveolar nerve, leading to paresthesia.
• Summary
• The complication is focal thermal osteonecrosis. Management is conservative debridement, antibiotics, antiseptic rinses, and close monitoring, with minor surgical intervention (sequestrectomy) if a fragment persists. The key lesson is prevention through proper surgical technique with adequate cooling.

30. In a 23-year-old patient, intraosseous anesthesia is administered during mandibular 38 extraction. Ten days later, the patient presents with an unhealed area of exposed bone at the extraction site and increasing pain. Which complication is this most likely to indicate, and how should management be planned?

• Based on the clinical presentation you've described, the most likely complication is Osteomyelitis of the mandible.
• Here's a detailed breakdown:
• Most Likely Complication: Osteomyelitis
• Why this fits the scenario:
• Timeline: Symptoms appearing 10 days post-extraction with worsening pain and failure to heal align with the subacute to chronic onset of osteomyelitis, rather than a simple dry socket (alveolar osteitis), which typically presents with severe pain 2-4 days post-extraction.
• Key Finding: The presence of an "unhealed area of exposed bone" that is not just a socket but an area of necrotic, exposed bone is highly suggestive of osteomyelitis. In dry socket, the bone is exposed but usually limited to the socket itself and the pain is often relieved by medicated dressings.
• Precipitating Factor: Intraosseous anesthesia involves directly penetrating the cortical bone to deposit anesthetic into the medullary space. This can introduce oral flora directly into the bone, acting as a potential nidus for infection. It also creates a direct pathway for bacteria from the contaminated extraction site into the deeper bone.
• Symptoms: "Increasing pain" beyond the normal post-operative period is a hallmark of bone infection, not merely a healing complication.

• Differential Diagnosis to Consider (but less likely):
• Dry Socket (Alveolar Osteitis): Pain peaks earlier (day 2-4), the exposed bone is typically within the socket, and there's usually no purulent discharge or systemic symptoms. The direct bone inoculation from the IO anesthesia makes simple dry socket less likely as the sole issue here.
• Foreign Body Reaction or Necrosis: Possible but less common; infection is a more frequent sequela.
• Fracture: Would present with different mechanics of pain (mobility, movement) and typically sooner.
• Management Plan
• Management should be aggressive and systematic to prevent progression to chronic osteomyelitis, pathological fracture, or systemic sepsis.
• 1. Immediate Assessment & Diagnosis:
• Clinical Exam: Thorough extra-oral and intra-oral exam for swelling, fistula, purulence, lymphadenopathy, and neurosensory deficits (e.g., inferior alveolar nerve involvement).
• Imaging:
o Cone Beam CT (CBCT) or CT Scan: The imaging gold standard. Essential to evaluate the extent of bone involvement, identify sequestra (necrotic bone fragments), involucrum (new bone formation), and rule out a pathological fracture. It provides far more detail than a panoramic radiograph.
o Panoramic Radiograph: May show initial changes like mottled radiolucency, but is often inadequate for full assessment in early osteomyelitis.
• Laboratory Tests: CBC with differential (look for leukocytosis, elevated neutrophils), ESR, and CRP (markers of inflammation/infection).
• Microbiology: Crucial. Obtain a deep bone biopsy or aspirate of purulent material (if present) for culture and sensitivity before starting antibiotics. This guides targeted antimicrobial therapy, as mandibular osteomyelitis is often polymicrobial (mixed aerobic/anaerobic oral flora).
• 2. Medical Management:
• Empiric Antibiotics: Start broad-spectrum IV antibiotics immediately after obtaining cultures. A common regimen covers anaerobes and gram-positive/negative bacteria (e.g., Clindamycin or Penicillin + Metronidazole, or Amoxicillin-Clavulanate). Adjust based on culture and sensitivity results.
• Analgesia: Manage pain with appropriate analgesics (often requiring opioids in the acute phase).
• Supportive Care: Ensure hydration and nutrition.
• 3. Surgical Management:
• Incision & Drainage: If there is a fluctuant abscess or purulent collection, it must be drained.
• Sequestrectomy & Debridement: This is the cornerstone of surgical treatment. All necrotic, non-vital bone (sequestrum) and grossly infected soft tissue must be surgically removed until bleeding, viable bone margins are reached. The intraosseous anesthesia tract likely needs to be explored and debrided.
• Wound Management: The wound may be left open to drain or loosely closed over a drain, depending on the extent.
• 4. Long-Term Follow-up:
• Extended Antibiotics: Transition from IV to oral antibiotics based on clinical response, typically for a prolonged course (4-8 weeks or more).
• Serial Imaging: Follow-up with CBCT/CT or radiographs to monitor bone healing and resolution of the infection.
• Reconstruction: If a significant bone defect remains after infection is fully eradicated, bone grafting may be considered in the future.
• Summary
• This presentation strongly points to osteomyelitis of the mandible secondary to intraosseous anesthesia and subsequent extraction. Management requires a combined medical-surgical approach: urgent imaging (CT/CBCT), deep culture, initiation of IV antibiotics, and prompt surgical debridement of necrotic bone, followed by a prolonged course of culture-directed antibiotics and close monitoring. Early and definitive intervention is key to preventing chronic, debilitating disease.

CHATGPT QUESTIONS AND ANSWERS:

1.In a 27-year-old patient, during surgical extraction of an impacted mandibular right third molar, an air-driven high-speed handpiece is used for bone removal. Shortly after the procedure, sudden swelling develops in the right cheek and submandibular region, with palpable crepitus on examination. What complication does this presentation suggest, by what mechanism does it occur, and how should it be managed?

This presentation is characteristic of subcutaneous emphysema associated with the use of an air-driven high-speed handpiece during mandibular third-molar surgery.
The complication occurs when compressed air is forced through the surgical wound into the loose connective tissues and fascial spaces. During bone removal, the air turbine may introduce pressurized air beneath the mucoperiosteal flap. The air can then dissect along the buccal, submandibular, sublingual, or deeper cervical fascial planes. The characteristic clinical finding is rapid-onset swelling accompanied by palpable crepitus, which helps distinguish emphysema from hematoma, edema, or allergic swelling. In more extensive cases, air may track into the neck and mediastinum, producing pneumomediastinum.
Management begins with immediate cessation of the air-driven instrument and assessment of the airway and the extent of emphysema. The patient should be evaluated for dysphagia, dyspnea, voice changes, neck swelling, chest pain, or respiratory distress. Limited cervicofacial emphysema without systemic symptoms is generally managed conservatively with observation, analgesia, and postoperative instructions. Because pressurized air introduced through an oral surgical wound may carry oral microorganisms into deeper tissue planes, broad-spectrum antibiotic coverage is commonly recommended.
The patient should be instructed to avoid activities that increase intraoral or intrathoracic pressure, such as vigorous nose blowing, forceful coughing, or straining. Most uncomplicated cases resolve spontaneously as the air is resorbed, typically within several days.
If swelling extends into the neck, respiratory symptoms occur, or pneumomediastinum is suspected, urgent hospital assessment and CT imaging of the neck and chest are indicated, with airway monitoring and appropriate medical or surgical consultation.
Diagnosis: Iatrogenic cervicofacial subcutaneous emphysema caused by pressurized air from an air-driven high-speed handpiece.
Mechanism: Forced entry of compressed air through the surgical wound into fascial tissue planes.
Treatment: Stop the air source, assess and monitor the airway, provide conservative care and antibiotic coverage, and obtain urgent imaging/hospital management if deep cervical or mediastinal extension is suspected.

2. In a 25-year-old patient, during removal of a fractured apical root fragment after extraction of tooth 28, the fragment is displaced apically and separated from the socket. Radiographs show that the fragment has been displaced into the maxillary sinus. What clinical pathway should be followed in this situation?

- Here's the clinical pathway generally recommended when a root fragment is displaced into the maxillary sinus during extraction (tooth 28 / upper left first premolar):
- 1) Immediate chairside actions (same appointment)
- Stop further blind instrumentation in the socket (probing often pushes the fragment deeper/posteriorly and enlarges any oroantral communication).
- Explain and document the complication, symptoms to watch for, and the next steps.
- Assess for an oroantral communication (OAC) clinically (gentle inspection; avoid aggressive Valsalva that can force contamination into the sinus). Untreated OAC can lead to sinusitis and later fistula formation. (PMC)
- Give sinus precautions immediately: no nose blowing, sneeze with mouth open, avoid straws/smoking, avoid forceful spitting, avoid pressure changes (diving/air travel if possible) until managed. (baos.org.uk)
- Analgesia as needed.
- Imaging:
  - o If you only have 2D films: take periapicals/occlusal + panoramic to confirm location.
  - o Ideally obtain CBCT to precisely localize the fragment and plan the retrieval route (especially to see whether it's on the sinus floor vs migrated). (jomos.org)
- 2) Decide: "Immediate transalveolar retrieval" vs "planned surgical retrieval"
- A) Try immediate transalveolar retrieval only if all are true

• The fragment is clearly accessible through the socket (e.g., sitting at/near the sinus floor, visible or easily reached),
• You can obtain good access and vision (adequate flap/retraction),
• You can retrieve it without blind upward pressure.
• If it's not readily accessible, don't persist—move to planned retrieval.
• B) If the fragment is truly inside the sinus (not accessible) → planned retrieval is recommended
• Most authors recommend removing displaced dental foreign bodies even if asymptomatic, because retained fragments can cause maxillary sinusitis or other sinonasal complications over time. (PMC)
• Timing: Retrieval is often advised early (ideally within days) before mucosal swelling/infection makes removal harder (exact timing depends on symptoms, fragment position, and local service availability). (PMC)
• 3) Choose the retrieval approach (OMFS ± ENT)
• The approach depends on fragment size, exact location, sinus condition, and local expertise:
• Option 1 — Endoscopic transnasal removal (FESS / ESS)
• Often favored when:
• The fragment has migrated posteriorly/superiorly or near the natural ostium,
• There is concurrent sinus disease needing treatment,
• ENT access is available.
• Option 2 — Intraoral antrostomy / (Modified) Caldwell–Luc approach
• Common OMFS route when:
• The fragment is in the sinus and not retrievable transalveolarly,
• A direct intraoral window offers predictable access.
• Case series and reviews describe the Caldwell–Luc / modified intraoral antrostomy as a reliable technique for retrieving displaced roots from the maxillary sinus. (PubMed)
• *(In practice, many teams use an intraoral approach with endoscopic assistance to reduce morbidity and improve visualization.) (jomos.org)
• 4) Manage the oroantral communication (if present)
• Small OACs may close spontaneously, but larger defects usually need immediate closure (e.g., buccal advancement flap, palatal flap, buccal fat pad—choice depends on size/location and surgeon preference). (PMC)
• Goal: prevent chronic contamination → sinusitis → epithelialized oroantral fistula. (PMC)
• 5) Medical support (commonly used adjuncts)
• Exact prescribing varies by guideline/local protocol and patient factors, but commonly:
• Antibiotic coverage when there is OAC and/or sinus contamination risk (e.g., amoxicillin–clavulanate; alternatives if allergic per local guidance),
• Nasal decongestant ± saline spray/irrigation,
• Continue sinus precautions and arrange close follow-up. (baos.org.uk)
• 6) Follow-up and red flags
• Arrange OMFS/ENT follow-up to monitor for:
• Facial pain/pressure, purulent nasal discharge, fever,
• Unilateral foul taste/odor, fluid passage nose↔mouth,
• Persistent OAC signs (air/fluid leakage). (PMC)
• Bottom line:
• Once radiographs confirm a root fragment has entered the maxillary sinus, the safe pathway is stop blind attempts, localize precisely (preferably CBCT), refer/plan retrieval (endoscopic transnasal or intraoral antrostomy/Caldwell–Luc depending on position), and close any oroantral communication with appropriate sinus precautions and follow-up. (PMC)

3. In a 29-year-old patient, during extraction of a deeply impacted tooth 48 that is related to the inferior alveolar nerve, a fractured apical root fragment is displaced apically and separated from the socket during attempted retrieval. Radiographs show that the fragment has been displaced into the inferior alveolar nerve canal. What clinical pathway should be followed, and what should the treatment approach be?

• I'll outline a practical OMFS decision pathway for an apical root fragment displaced into the inferior alveolar canal—what to do immediately, what imaging is needed, when to refer/explore surgically, and how to manage the nerve injury risk.
• When a root fragment is displaced into the inferior alveolar nerve (IAN) canal, the

priority shifts from “get the fragment out” to preventing iatrogenic nerve injury and managing it like a foreign body within/against a major sensory nerve. The safest pathway is stop → localize precisely (CBCT) → specialist (OMFS) decision: early controlled retrieval vs deliberate retention with close follow-up.

• 1) Immediate intra-op / same-visit actions (what to do right now)
• Stop blind retrieval attempts.
• “Chasing” the fragment with elevators/burs is the #1 way to convert a neurapraxia risk into axonotmesis/neurotmesis.
• Document and examine neurosensory status immediately (before LA fully wears off if possible, then repeat once it does):
o Light touch, pinprick, two-point discrimination, direction, thermal if you do it routinely
o Map distribution (lower lip/chin/teeth/gingiva on that side)
• Inform the patient clearly (displacement into the canal, nerve-injury risk, need for advanced imaging and OMFS management). Document the discussion.
• Do not enlarge the socket aggressively “hoping it will pop out.” Irrigate gently, achieve hemostasis, and close (primary closure if feasible).
• Analgesia and consider short-course corticosteroid (common OMFS practice to reduce edema around the nerve).
• Antibiotics: not mandatory purely because it’s in the canal, but many clinicians prescribe if there was significant manipulation, contamination, or high infection risk (use local protocols).
• 2) Imaging (mandatory next step)
• Obtain CBCT (or equivalent 3D imaging) to determine:
o Exact position of the fragment (within canal vs cortical perforation into canal)
o Proximity to the mental foramen, canal wall integrity, and whether the fragment is mobile
o The safest surgical corridor (buccal window vs limited troughing, etc.)
• Plain radiographs confirm “in the canal,” but CBCT guides whether retrieval is feasible without excessive nerve manipulation.
• 3) Decision point: retrieve vs retain (how you choose)
• Because the fragment is in the IAN canal, early specialist review is strongly indicated. The decision usually hinges on:
• Retrieval is generally favored when:
• Any neurosensory deficit is present or developing (paresthesia, anesthesia, dysesthesia)
• Fragment is large, sharp, or clearly compressing the canal contents
• Fragment appears mobile or likely to migrate
• There is infection, persistent pain, or a high risk of future infection
• CBCT shows it can be accessed with controlled exposure (minimizing nerve trauma)
• Deliberate retention (leave it) can be considered when:
• Patient has normal sensation
• Fragment is small, stable, and deep such that retrieval would require significant canal opening / nerve handling
• CBCT suggests retrieval risk to the nerve is higher than the benefit
• If you retain: it must be an intentional plan, not “we couldn’t get it.” That means informed consent + scheduled neurosensory follow-up + repeat imaging if symptoms arise.
• Practical OMFS bias: A fragment *within the canal* often pushes the team toward early controlled management (sometimes retrieval, sometimes observation), but not blind attempts.
• 4) If retrieval is chosen: recommended treatment approach (OMFS)
• This should be performed by an OMFS comfortable with IAN exposure (and ideally microsurgical principles).
• Goals: remove the fragment with minimal traction/compression on the nerve; decompress if needed; preserve canal contents.
• Typical steps (conceptual):
• Intraoral approach with a buccal cortical window / controlled osteotomy guided by CBCT (often piezosurgery to reduce soft-tissue injury risk)
• Identify the canal region; gentle exposure only as much as needed
• Atraumatic removal of the fragment (micro-instruments, minimal suction near nerve, avoid grasping that “tugs” the nerve)

• If nerve is visibly compressed: decompression / external neurolysis may be performed depending on findings and expertise
• Achieve hemostasis without packing that compresses the canal aggressively
• Close; consider adjuncts per surgeon preference (e.g., anti-edema regimen)
• Timing: If retrieval is indicated (especially with symptoms), many OMFS teams aim for early intervention (often within days) rather than waiting weeks—because fibrosis/inflammation can make later removal harder and nerve recovery less predictable.
• 5) Post-op pathway and follow-up (regardless of retrieve vs retain)
• Standardized neurosensory follow-up (documented): baseline → 1 week → 1 month → 3 months (or per protocol).
• If neuropathic symptoms occur: manage pain appropriately (avoid undertreating dysesthesia), and reassess imaging if worsening.
• Red flags requiring urgent reassessment:
o Increasing numbness, painful dysesthesia, anesthesia
o Progressive pain/swelling/infection signs
• If a significant sensory deficit persists (especially with dysesthesia) beyond the expected recovery window, consider referral to a nerve injury/microsurgery pathway. Many surgeons consider exploration/repair decisions within the first ~3 months if severe and not improving, but the exact threshold depends on the type of deficit and exam findings.
• Bottom line (what you'd write as the clinical pathway)
• Stop retrieval attempts immediately → document neurosensory status → CBCT → urgent OMFS referral/assessment.
• Treatment is CBCT-guided decision:
o Early controlled surgical retrieval/decompression if symptomatic, large/mobile, compressive, infected, or safely accessible; OR
o Deliberate retention with strict neurosensory follow-up if asymptomatic and retrieval risk is higher than benefit.
• If you want, I can format this as a clean "algorithm box" (flowchart style) you can paste into notes or a case discussion, and a separate paragraph for the informed-consent wording.

4. In a 28-year-old patient using oral contraceptives, severe pain and a foul odor develop a few days after extraction of tooth 38. Which postoperative complication is this most consistent with, and how should treatment be planned?

• This presentation is most consistent with alveolar osteitis (dry socket, postextraction alveolitis) after mandibular third molar extraction. The timing—2 to 4 days after removal of 38—together with severe throbbing pain and foul odor/halitosis, is classic. Use of oral contraceptives is a recognized risk factor, likely through increased fibrinolysis and loss of the socket clot.

Management should be palliative and local, not surgical. The patient should be examined to confirm a socket with partial/complete clot loss, exposed bone, and debris, while also excluding a true postoperative infection such as cellulitis or osteomyelitis. Established alveolar osteitis is treated primarily by gentle intra-alveolar irrigation to remove food debris and necrotic material, because symptom relief—not acceleration of healing—is the main objective. Socket curettage is not recommended, as it may further expose bone and worsen pain.

After irrigation, pain control is planned with NSAID-based analgesia unless contraindicated, and many OMFS protocols add a short-term obtundant intra-alveolar dressing such as a eugenol-containing dressing for severe pain, with reassessment and dressing change/removal as needed over the next 24 to 48 hours. Topical or long-acting local anesthetic may be used for immediate relief. Dressings should not be left indefinitely because they can delay healing or act as a foreign body.

Systemic antibiotics are not routinely indicated for uncomplicated dry socket, even if there is malodor, because alveolar osteitis is not primarily a spreading infection. Antibiotics are reserved only if there are signs of true infection beyond the socket, such as fever, diffuse swelling, progressive trismus, purulence, lymphangitic spread, or cellulitis.

So, in specialist terms: this is postextraction alveolar osteitis of 38, and treatment should be local debridement by irrigation, analgesia, optional short-term sedative dressing, close review, and no routine antibiotics unless superimposed infection is present.

5. In a 24-year-old patient, persistent numbness of the lower lip and chin region for three months following a difficult mandibular right 48 extraction suggests which neurological complication, and how should this condition be managed?

• Persistent numbness of the lower lip and chin (mental region) for 3 months after a difficult mandibular right 48 extraction most strongly suggests an inferior alveolar nerve (IAN) injury (i.e., mental nerve territory paresthesia/anesthesia). Clinically this is "IAN neuropathy" ranging from neuropraxia (conduction block) to

axonotmesis/neurotmesis depending on severity.
• How to manage it (practical OMFS approach)
• 1) Confirm and document the deficit
• Full neurosensory exam and mapping: light touch, pin-prick, two-point discrimination, directional brush, thermal; grade severity (e.g., MRCS scale).
• Ask about improvement trend (any recovery over time is a good prognostic sign) and whether symptoms are pure numbness vs dysesthesia/neuropathic pain (burning, electric shocks).
• 2) Identify potentially reversible causes
• Review operative details (difficulty, root fracture, excessive elevation, bleeding/hematoma, proximity to canal).
• Obtain imaging (often CBCT) if not already done to look for:
o retained root/tooth fragment in/near canal,
o bony spicules, compression,
o displaced material, or other canal insults.
• 3) Early conservative care (still appropriate at 3 months if improving)
• Observation with scheduled re-evaluation (e.g., every 4–6 weeks) if there is *clear, progressive sensory return*.
• If unpleasant sensations or neuropathic pain: consider neuropathic pain agents (e.g., gabapentin/pregabalin, TCAs) and/or topical options as appropriate.
• Patient counseling: recovery can take months; emphasize careful lip care (avoid biting/thermal injury).
• 4) Escalate when recovery is not happening
• At 3 months, you should refer to an OMFS unit experienced in trigeminal nerve injuries / microsurgery now, especially if:
• there is no objective improvement, or
• there is significant dysesthesia, or
• imaging suggests a mechanical issue.
• 5) Surgical options (time-sensitive)
• If there is minimal/no improvement by ~3–6 months, and findings suggest significant injury, consider:
• Surgical exploration ± decompression (if compression/entrapment suspected),
• External/internal neurolysis (selected cases),
• Microsurgical repair (neurorrhaphy) or nerve grafting if discontinuity is suspected/confirmed.
• Key point: The “window” where surgery is most likely to help is generally within the first several months—so persistent numbness at 3 months is the moment to formalize assessment + refer, not just “wait indefinitely.”
• If you want, tell me whether there has been *any* improvement since week 1–2 and whether there’s pain/burning vs pure numbness—those two details strongly change the urgency and the expected prognosis.

6. In a 26-year-old patient, following lingual retraction during extraction of tooth 38, numbness and loss of taste develop on the left half of the tongue. Which nerve injury does this suggest, and how should it be treated?

• This presentation strongly suggests an injury to the lingual nerve on the left side (often including its chorda tympani taste fibers), because the lingual nerve supplies general sensation to the anterior 2/3 of the tongue and carries taste from that same region via chorda tympani.
• What nerve injury is most likely?
• Left lingual nerve injury (traction/compression or laceration from lingual retraction during mandibular third molar surgery).
• How to treat / manage it (practical approach)
• Management depends on whether this is likely a temporary conduction block (neuropraxia) vs partial/complete transection.
• 1) Immediate steps (same day / first visit after surgery)
• Document neurosensory status carefully: light touch, pin-prick, two-point discrimination, temperature, and map the numb area; note taste complaint.
• Explain prognosis: many lingual nerve injuries after 38 removal are temporary and improve over weeks to a few months.
• Avoid further trauma to the tongue (hot foods, biting).
• 2) Early conservative treatment (first days–weeks)
• Observation + scheduled follow-up is the mainstay for suspected neuropraxia/traction

injury.
• Symptomatic care:
o Analgesics/anti-inflammatory meds as needed.
o If there is neuropathic pain/dysesthesia (burning, electric shocks), consider neuropathic agents (e.g., gabapentin/pregabalin) under appropriate prescribing/monitoring.
• Many clinicians consider a short early course of corticosteroids (best if started very early) for suspected compression/traction injury—practice varies, and it's not a substitute for timely referral if severe/persistent.
• 3) Follow-up timeline (key decision points)
• Reassess at 1–2 weeks, then 4–6 weeks, then ~3 months with the same neurosensory tests.
• Improvement by 4–8 weeks is a good sign and usually supports continued observation.
• 4) When to refer / consider surgery
• Refer to an OMFS unit with microsurgical experience if:
• Complete anesthesia (no sensation) or significant taste deficit with no improvement, or
• A transection is suspected (sharp "cut" mechanism, severe persistent deficit), or
• Deficit persists and is functionally significant beyond ~2–3 months.
• Surgical options (case-dependent): exploration, decompression, neurorrhaphy, nerve grafting.
• Best outcomes are generally reported when indicated repairs are done early (often within ~3 months; up to 3–6 months in selected cases), rather than waiting too long.
• 5) If transection was clearly seen intraoperatively
• Immediate or very early repair (same day/within days) is preferred when feasible.
• Bottom line: This is most consistent with a left lingual nerve injury from retraction. Start with documentation + reassurance + close follow-up, treat symptoms, and refer early if the deficit is complete or not improving—because the window for best surgical outcomes is time-sensitive.

7. In a 26-year-old patient, a tooth is lost during extraction of tooth 28, and two days later CBCT shows the tooth in the infratemporal fossa. What does this finding indicate, and what is the appropriate treatment approach?

CBCT showing the extracted tooth sitting in the infratemporal fossa means this is an iatrogenic displacement of tooth 28 (usually through a fractured/perforated posterior maxillary tuberosity/periosteum during elevation), with migration into the infratemporal space over the next 1–2 days. This is a *true "lost tooth"* in a deep anatomic space (near the pterygoid plexus/maxillary artery and branches of V3), so blind intraoral digging is contraindicated.
• Appropriate treatment approach
• Do not attempt further blind retrieval (high risk of pushing it deeper and causing bleeding/nerve injury).
• Refer urgently to OMFS (this is not a routine dental retrieval).
• Plan retrieval based on symptoms + position on CBCT:
o If symptomatic (progressive pain, trismus, swelling, fever, dysphagia, neurologic symptoms) or CBCT shows it is accessible:
• → Early surgical removal (ideally by an experienced OMFS; often under GA).
• Common approach is a wide intraoral posterior maxillary/vestibular flap with careful blunt dissection toward the infratemporal space; many centers use image guidance / endoscopic assistance to minimize morbidity.
o If asymptomatic and very deep/unfavorable position (high risk to retrieve immediately):
• → Some surgeons choose short delay (≈2–4 weeks) to allow fibrous encapsulation, then remove with better control (still OMFS-level surgery). This is *case-dependent* and requires close follow-up.
• Supportive care while awaiting surgery/decision
o Analgesia, soft diet, limit wide opening.
o Antibiotics are commonly prescribed (because this is a contaminated foreign body in deep tissues), plus antiseptic mouth rinse.
o Repeat imaging if symptoms change or before delayed retrieval.
• Key point: The CBCT finding indicates a displaced tooth into the infratemporal fossa, and the correct management is OMFS-led, image-guided/controlled surgical retrieval (often early), or carefully selected delayed retrieval if deep and asymptomatic—but *never blind exploration*.

8. In a 34-year-old patient, a mandibular basal (inferior border) fracture occurs during extraction of a deeply impacted tooth 38 due to excessive elevator force. What should the emergency management approach be?

• I'll outline a practical emergency (same-visit) management pathway for an iatrogenic mandibular basal/inferior border fracture that occurs during a difficult 38 extraction—what to do immediately, what to avoid, and how to hand off for definitive fixation.
• Emergency management (chairside / ED)
• 1) Stop the extraction immediately
• Do not continue elevating or "fight through" the case.
• If the tooth is partially mobilized, don't attempt forceful delivery—this can displace segments and worsen the fracture.
• 2) Primary survey: airway–bleeding–neuro status
• Assess airway and swallowing (rare but critical if floor-of-mouth hematoma/bleeding).
• Control bleeding with pressure, suction, hemostatic agents as needed.
• Quick exam:
o Occlusion change, step deformity, mobility/crepitus at inferior border
o Trismus, pain on function
o Inferior alveolar / mental nerve symptoms (lip/chin numbness)
o Check for lacerations/open communication (socket makes this *functionally "open/contaminated"* in many cases).
• 3) Stabilize the mandible
• Goal: reduce motion → reduce pain/bleeding and prevent displacement.
• Apply a Barton bandage / supportive jaw wrap (temporary external stabilization).
• In a facility with OMFS capability: consider temporary IMF (intermaxillary fixation) *only if trained and appropriate*; avoid if aspiration risk, vomiting risk, poor airway, intoxication, etc.
• Give clear instructions: no chewing, minimal talking.
• 4) Analgesia + infection prophylaxis
• Pain control: NSAID ± opioid as required.
• Because the fracture occurred via an extraction site, manage as contaminated:
o Start antibiotics covering oral flora (e.g., amoxicillin-clavulanate; clindamycin if penicillin-allergic—local protocols apply).
o Chlorhexidine mouth rinse.
o Update tetanus status if indicated (especially if there's a mucosal laceration/"open" fracture concern).
• 5) Imaging urgently
• Obtain panoramic radiograph immediately if available.
• CT/CBCT is preferred to define:
o fracture line (displacement/comminution)
o relation to inferior alveolar canal
o whether the tooth/root fragment lies in the fracture line or has displaced.
• 6) Definitive referral and plan
• This is typically an urgent OMFS/maxillofacial trauma referral the same day.
• Definitive treatment depends on displacement and occlusion:
• Displaced basal/inferior border fractures → usually ORIF (open reduction internal fixation) with plates/screws (often along inferior border) ± short-term IMF.
• Non- or minimally displaced, stable occlusion (less common in basal fractures) → possible closed treatment (IMF/soft diet) under close follow-up.
• 7) What to do about the tooth (key practical point)
• Do not persist with traumatic extraction.
• Whether to remove the tooth at the time of fixation depends on:
o tooth/root in the fracture line
o contamination, infection, mobility
o access during ORIF
• Commonly: extraction is completed atraumatically during ORIF *if* it interferes with reduction/stability or is clearly within the fracture line; otherwise it may be left temporarily to avoid further fragmentation—this is a surgeon decision based on imaging and stability.
• "Do not" list (avoids worsening the fracture)
• Don't continue aggressive elevators or attempt blind retrieval of fragments.
• Don't send home without stabilization + imaging + urgent OMFS referral if occlusion is altered or mobility is present.

• Don't assume it's a "crack" only—basal fractures can displace with function.
•
• Discharge instructions if transfer/definitive care is pending
• Soft/liquid diet, no chewing.
• Jaw support kept in place.
• Strict return precautions: increasing swelling, dysphagia, dyspnea, uncontrolled bleeding, fever, worsening numbness, inability to occlude.
• If you want, tell me whether occlusion is altered and whether the fracture looks displaced —I can map the likely definitive pathway (closed vs ORIF) more specifically.

9. In a 28-year-old patient, one week after extraction of tooth 48, progressively worsening severe pain, trismus, fever, and cervical lymphadenopathy develop. The patient also has dysphagia/odynophagia, diffuse neck swelling, and chest pain–shortness of breath, raising suspicion of spread of infection through deep neck spaces to the mediastinum. Which diagnosis does this presentation most strongly suggest?

• This presentation is most consistent with an odontogenic deep neck space infection complicated by descending necrotizing mediastinitis (DNM) — i.e., infection tracking from the submandibular/parapharyngeal–retropharyngeal pathways into the mediastinum, producing neck sepsis + chest pain and dyspnea.

10. In a 25-year-old patient, six weeks after mandibular 48 extraction, the patient reports food impaction and bleeding distal to tooth 47. Clinical examination reveals a ≥6 mm periodontal pocket distal to 47 with bleeding on probing; radiographs show localized bone loss distal to 47. What surgical and postoperative approach should be implemented in this case?

• You're describing a post–third molar distal periodontal defect on the mandibular second molar (47) (deep pocket ≥6 mm with BOP + localized distal bone loss) that has persisted into the "early periodontal healing" window at ~6 weeks. At this point, food impaction + bleeding usually means the site needs surgical access for thorough debridement and recontouring/regeneration, not just rinses.
• Surgical approach (what to do)
• 1) Re-evaluate and rule out local causes
• Check for retained bony sequestrum, granulation tissue, enamel/cementum damage on distal 47, overhanging restorations, or a foreign body.
• Assess the defect morphology (2-wall / 3-wall vertical vs shallow crater) clinically and on radiograph; CBCT can help if the anatomy is unclear or if you suspect a residual fragment.
• 2) Periodontal access surgery (core step)
• Full-thickness mucoperiosteal flap distal/buccal (often with a small lingual reflection only if needed) to expose:
• Distal root surface of 47
• Extraction site/distal osseous crater
• Then perform:
• Thorough degranulation + irrigation
• Root surface debridement / planing of distal 47
• Remove any sequestra or residual pathology
• 3) Choose the corrective procedure based on defect type
• A) If the distal defect is shallow or mostly an irregular bony ledge/crater (non-contained):
• Distal wedge procedure (excision of distal tissue wedge) + osteoplasty/ostectomy to recontour the distal bone and eliminate the plaque trap.
• Aim for a cleansable distal anatomy and pocket reduction.
• B) If there is a vertical, more contained defect (especially 2–3 wall component) where regeneration is realistic:
• Regenerative surgery (preferred):
o Bone graft (autograft/allograft/xenograft) ±
o Guided tissue regeneration (membrane) and/or
o Biologics (e.g., enamel matrix derivative) depending on your protocol.
• Achieve primary closure without tension.
• 4) Flap closure
• Position flap to allow stable closure (often slightly coronal/at CEJ level depending on goals).
• Interrupted/vertical mattress sutures for tight adaptation and to minimize food impaction.
• Postoperative approach (what to prescribe and how to follow)

• Meds & hygiene
• Chlorhexidine 0.12–0.2% rinse (or gel locally) 2×/day for 1–2 weeks.
• Analgesics/NSAIDs as needed.
• Antibiotics: not mandatory for every case; consider if there was significant suppuration, extensive regeneration with biomaterials, systemic risk factors, or clinician preference.
• Mechanical plaque control
• No brushing directly on the wound area for ~1 week; then very gentle brushing as tolerated.
• Once soft tissues allow (often after suture removal), start targeted interdental cleaning distal to 47:
o Superfloss/interdental brush if embrasure permits
o Consider a mono-tuft brush aimed distal to 47
• Diet & function
• Soft diet, avoid packing fibrous foods into the distal area.
• Avoid vigorous spitting/suction early; keep the site clean with gentle rinsing.
• Follow-up schedule
• 1 week: evaluate soft tissue, remove sutures, reinforce hygiene.
• 4–6 weeks after surgery: reassess pocket depth and BOP.
• 3–6 months: radiographic review if regenerative therapy was done; long-term maintenance.
• Key practical point
• At 6 weeks post-extraction with ≥6 mm pocket + BOP + radiographic bone loss, the most predictable pathway is open flap debridement with either:
• distal wedge + osseous recontouring (if non-contained/shallow), or
• regeneration (GTR/graft/biologics) if the defect morphology supports it.
• If you want, tell me whether the bone loss looks more like a vertical defect or a broad crater, and whether there's suppuration—I'll map it to the most appropriate option (resective vs regenerative) and flap design.

11. In a 29-year-old patient, recurrent bleeding from the extraction site begins approximately one week after extraction of mandibular left 38. What mechanism could explain this delayed bleeding, and how should it be managed?

• This pattern—bleeding that starts/restarts ~1 week after an extraction—most classically fits secondary (delayed) hemorrhage, usually from an infected socket.
• Likely mechanism (why it happens "late")
• Around days 5–10, a socket can become secondarily infected or develop localized inflammation.
• Bacteria + inflammation can trigger fibrinolysis (breakdown of the clot) and tissue sloughing, and can erode a small vessel in the socket wall → the clot "gives way" and bleeding recurs.
• (Contrast: primary hemorrhage is immediate; reactionary is within hours. A week later is typically *secondary* and infection-related.)
• Management (practical OMFS approach)
• 1) Immediate assessment
• Check airway/aspiration risk, hemodynamic status (BP/HR), estimate blood loss.
• Quick history: anticoagulants/antiplatelets, herbal supplements, liver disease, prior bleeding issues.
• 2) Local control (first-line)
• Suction + inspect the socket (good light, retract well).
• Irrigate gently (saline) and remove loose debris/clot if it's unstable/infected.
• Local anesthesia with epinephrine (if not contraindicated) to reduce bleeding and allow exploration.
• Direct pressure: bite on gauze for 20–30 minutes (no checking every 2 minutes).
• If still bleeding: place a local hemostatic into the socket (choose what you have):
o oxidized regenerated cellulose / gelatin sponge / collagen plug
o consider topical tranexamic acid (TXA-soaked gauze if available)
• Figure-of-eight suture over the socket to secure the pack.
• 3) Treat the cause (if socket looks infected)
• If there are signs of infection (fetid odor, purulence, increasing pain/swelling, granulation/slough):
o Irrigation + debridement as needed
o Antibiotics if clinically infected/cellulitis/systemic signs (regimen per local protocol)
o Analgesia and hygiene instructions (avoid vigorous rinsing for 24 h after

hemostasis)
• 4) Escalate when indicated
• Persistent bleeding despite good local measures, brisk pulsatile bleeding, expanding swelling, or recurrent significant episodes:
o send for CBC + coagulation tests (and medication review)
o consider arterial source (uncommon but important) → hospital management, possible ligation/embolization.
• 5) Discharge instructions (to prevent re-bleed)
• No smoking/alcohol, no spitting/sucking through straw, soft/cool foods initially.
• If it restarts: bite on firm gauze for 30 minutes continuously; if it doesn't stop or there's dizziness/weakness → urgent care.
• If you want, tell me whether the socket has increasing pain/foul taste, and whether the patient is on anticoagulants/aspirin—those two details help prioritize "infection-driven secondary hemorrhage" vs "systemic/medication-related bleeding."

12. In a 22-year-old patient, soft tissue trauma–related tissue hemorrhage occurs during local anesthetic injection prior to mandibular 38 extraction. How would you proceed with the procedure in this situation?

• I'd treat this as an injection-related hematoma/soft-tissue bleed. The key decision is whether it's small and stable (you can usually continue) or rapidly expanding / floor-of-mouth involvement (you should stop).
• 1) Immediate actions (right away)
• Stop the injection and withdraw the needle.
• Direct pressure with gauze over the puncture site for at least 2–5 minutes (longer if still oozing).
• Reassess: size, firmness, rate of enlargement, pain, and function (mouth opening, swallowing, voice).
• 2) Decide whether to proceed
• Proceed with the extraction (most common) if:
• Swelling/bleeding is localized, not enlarging, and you can achieve adequate anesthesia.
• No signs of airway/space compromise (see below).
• Postpone/abort and manage urgently if any red flags:
• Rapidly expanding swelling, especially in the floor of mouth/submandibular region
• Tongue elevation, dysphagia, drooling, muffled voice, dyspnea/stridor
• Significant trismus developing quickly
In these cases:
➡️
stop the procedure, keep the patient upright, activate emergency pathway, and prioritize airway (urgent OMFS/ED).
• 3) If you proceed: how to continue safely
• After pressure control, use cold compress externally (ice pack) for 10–15 min intervals.
• Avoid reinjecting into the traumatized/bleeding area.
• If anesthesia is inadequate:
o Re-attempt block only if necessary with excellent technique: aspirate in two planes, inject slowly, minimal redirections.
o Consider an alternative approach (e.g., different block technique or supplemental infiltration away from the hematoma) rather than multiple passes in the same region.
• During extraction: be gentle with soft tissues; consider that swelling/bruising may worsen.
• 4) Post-event instructions & follow-up (even if you finish the extraction)
• Explain this is usually self-limiting bruising/swelling.
• Ice for first 24 hours, then warm compresses from 24–48 hours onward to speed resorption.
• Analgesia (e.g., NSAID/acetaminophen as appropriate).
• Warn to return urgently if: swelling increases rapidly, difficulty swallowing/breathing, fever, worsening trismus, or uncontrolled bleeding.
• Document the event (site, size, progression, management).
• Bottom line: control bleeding with pressure, assess stability. If it's small and not expanding, you can usually continue with the extraction using cautious anesthetic technique and good aftercare. If there are floor-of-mouth/airway red flags or rapid expansion, stop and escalate immediately.

13. A 25-year-old patient presents two days after mandibular 48 extraction with limited mouth opening and difficulty eating. How should the patient be assessed and managed?

• Two days after a mandibular 48 extraction, trismus (limited mouth opening) is common from postoperative inflammation and medial pterygoid/masticator muscle spasm—but you must rule out early odontogenic infection or a developing space infection.
1) Assess (what to check immediately)
A. Rapid "red flag" screen (if any → urgent escalation)
Ask and examine for:
• Fever, rigors, systemic malaise
• Progressively increasing swelling, especially submandibular/sublingual
• Dysphagia, odynophagia, drooling, muffled voice
• Dyspnea/stridor (airway concern)
• Trismus severe (e.g., MIO < ~20 mm) or rapidly worsening
• Toxic appearance, dehydration, inability to take oral fluids
B. History focused on likely causes
• Onset: immediate vs gradual worsening after day 1
• Pain pattern: improving vs escalating
• Medications taken and effect (NSAIDs?)
• Any foul taste/drainage
• Trauma during surgery: long/difficult extraction, heavy flap retraction, injection pain (medial pterygoid irritation)
• Medical risks: smoking, diabetes/immunosuppression
C. Exam
• Vitals (temp, HR)
• Measure maximum interincisal opening (MIO) and note deviation
• Extraoral: swelling, firmness, tenderness, lymph nodes
• Intraoral: socket appearance (clot, debris), erythema, purulence, fluctuance
• Check occlusion and mandibular border tenderness (rule out rare fracture if suspicious)
2) Management (based on findings)
If it looks like uncomplicated early postoperative trismus (most common)
Typical picture: mild–moderate trismus + expected soreness/swelling, no fever, no spreading swelling, no pus.
• Analgesia/anti-inflammatory: NSAID (if safe) ± acetaminophen on a regular schedule for 48–72h
• Warm compresses (from 24–48h postop onward) + gentle massage
• Jaw physiotherapy: frequent gentle opening exercises (short sessions, many times/day; avoid forcing)
• Soft diet, hydration; avoid hard chewing
• Socket care: saline or chlorhexidine rinses (if you routinely use them) and good oral hygiene
• Review in 24–48h to ensure opening and symptoms are improving
If infection is suspected (worsening pain/swelling, fever, pus, increasing trismus, bad taste)
• Irrigate the socket, remove debris; obtain drainage if present
• Start antibiotics when clinical infection is present (choice depends on local protocol/allergy; commonly amoxicillin-clavulanate or penicillin + metronidazole; clindamycin if true penicillin allergy)
• Close follow-up in 24 hours
• If fluctuance/collection → incision and drainage (don't rely on antibiotics alone)
If deep space infection/airway risk is suspected (red flags)
• Urgent hospital assessment
• Contrast CT of neck/mandible region if space infection is suspected
• IV antibiotics, surgical drainage as indicated
• Airway monitoring/airway plan if submandibular/sublingual involvement
Practical "decision rule" you can use
• Stable, afebrile, swelling not spreading, no pus → conservative trismus care + reassess soon.
• Any systemic signs, spreading swelling, pus, rapidly worsening trismus, swallowing/voice/breathing symptoms → treat as infection/space infection and escalate.
If you tell me: MIO in mm, presence/absence of fever and swelling location (buccal vs submandibular/sublingual), and whether there's pus/foul taste, I can map it to the most likely diagnosis and the safest pathway.

14. In a 55-year-old patient with a history of four doses of intravenous bisphosphonate (zoledronic acid) use, an unhealed area of exposed bone and pain develop weeks after extraction of mandibular 38. Which complication is most likely, and how should management be planned?

• I'll identify the most likely diagnosis given IV zoledronic acid exposure after extraction, then lay out a practical, stepwise OMFS management plan (assessment, staging, imaging, meds, surgery thresholds, and follow-up).
• Most likely complication: medication-related osteonecrosis of the jaw (MRONJ) (historically "BRONJ") triggered by an extraction in a patient exposed to IV zoledronic acid.
• A small nuance: the *formal* MRONJ definition requires exposed bone (or bone probeable through a fistula) persisting ≥8 weeks in a patient with antiresorptive/antiangiogenic exposure and no jaw radiotherapy/metastasis. So "weeks after extraction" = highly suspicious/incipient MRONJ (or delayed healing) and should be managed as presumed MRONJ and followed closely—if it persists to 8 weeks, it meets criteria.
• Management plan (practical OMFS pathway)
• 1) Confirm risk + assess severity
• History: indication for zoledronic acid (oncology vs osteoporosis), timing of last dose, steroids/chemo, diabetes, smoking, oral hygiene.
• Clinical exam: size of exposure, fistula, purulence, mobility of sequestrum, vestibular swelling, neurosensory changes.
• Imaging: panoramic + consider CBCT if symptoms/signs suggest deeper involvement (sequestrum, cortical disruption, IAN proximity, pathologic fracture risk).
• Labs (optional, case-dependent): if systemic infection suspected (CBC/CRP), glycemic control, etc.
• Avoid routine biopsy unless the appearance is atypical or malignancy is in the differential.
• 2) Start with conservative (stage-based) care
• Because the case has pain and exposed bone, treat at least like AAOMS Stage 2 *if infection/inflammation is present*.
• Core measures (all suspected/confirmed stages):
• Chlorhexidine 0.12–0.2% rinse (gentle swish; no aggressive irrigation).
• Analgesia (NSAID ± acetaminophen as appropriate).
• Eliminate local trauma: smooth sharp bony edges only if they're traumatizing soft tissue; avoid irritating dentures/chewing on that side.
• Close follow-up (every 2–4 weeks) to document progression toward mucosal coverage.
• If pain and/or infection signs (common after extraction):
• Systemic antibiotics tailored to odontogenic flora and tolerance:
o Typical first line: amoxicillin-clavulanate (or penicillin VK)
o If penicillin allergy: doxycycline or clindamycin (regional resistance patterns matter; monitor C. difficile risk with clinda).
• Culture is usually unnecessary unless refractory/atypical.
• 3) Coordinate with the prescribing physician
• Inform the prescriber (oncology/endocrinology/rheumatology) that this is suspected MRONJ post-extraction.
• "Drug holiday" for IV zoledronic acid is not reliably proven to prevent/treat MRONJ and must be individualized.
o For oncology dosing, stopping can be risky for skeletal-related events.
o For osteoporosis dosing, temporary pause may be considered case-by-case, but you still manage the lesion locally the same way.
• 4) Decide when to operate (don't rush, but don't wait forever)
• Indications to escalate beyond conservative care:
• Persistent pain/infection despite adequate conservative therapy
• Increasing exposed bone, recurrent abscess, sinus tract, progressive radiographic necrosis/sequestrum
• Complications (IAN involvement, extraoral fistula, fracture risk)
• Surgical approach (when indicated):
• Sequestrectomy / superficial debridement if there is loose sequestrum causing irritation/infection.
• If disease is more extensive: resection to bleeding viable bone with primary closure can give better long-term control in selected cases (especially advanced/refractory lesions).
• Aim for tension-free mucosal closure; consider local flap advancement if needed.
• 5) Patient counseling (important)
• Explain that healing can be prolonged and goals are pain control + infection control + mucosal coverage.
• Reinforce meticulous oral hygiene and avoiding trauma.
• Document lesion size, photos, symptoms, and follow-up timeline (including the 8-week

diagnostic threshold).
• What this vignette most closely fits
• A 55-year-old with IV zoledronic acid and pain + exposed bone weeks after extraction is most consistent with suspected early MRONJ / evolving MRONJ. Manage immediately with conservative MRONJ protocol, treat infection/pain, image as needed, coordinate with prescriber, and reassess—if it persists to ≥8 weeks, it is MRONJ and you stage/continue accordingly (often Stage 2 in symptomatic cases).

15. In a 55-year-old patient with a history of radiotherapy, an unhealed area of exposed bone and pain develop weeks after extraction of tooth 38. Which complication is this associated with, and how should it be managed?

• This presentation is most consistent with osteoradionecrosis of the jaw (ORNJ)—radiation-injured mandibular bone that fails to heal after a trigger like a dental extraction, leading to persistent exposed bone + pain. (eviq.org.au)
• How to manage it (practical clinical pathway)
• 1) Treat as suspected ORN and refer early (OMFS ± oncology/ENT MDT).
• Management is often stage-dependent and multidisciplinary. (eviq.org.au)
• 2) Confirm extent + exclude tumor recurrence
• Verify the site is within the prior radiation field and review RT dose/field if available. (eviq.org.au)
• Imaging: start with dental radiographs as needed, but CT is more precise for bony extent (MRI for adjacent soft tissues). (eviq.org.au)
• Exclude recurrence when relevant (PET may help but can be false-positive from inflammation). (eviq.org.au)
• 3) First-line (conservative) treatment for early/stable disease
• Analgesia/anti-inflammatory meds for pain. (eviq.org.au)
• Antibiotics only if there is acute infection/cellulitis/suppuration (not automatically for "bone exposure"). (eviq.org.au)
• Meticulous oral hygiene (often with antiseptic rinses) and eliminate local trauma/irritants. (eviq.org.au)
• Sequestrectomy / removal of devitalized bone when indicated. (eviq.org.au)
• Many centers consider anti-radiation fibrosis medical therapy (e.g., pentoxifylline + tocopherol–based regimens) for established ORN as part of conservative management. (eviq.org.au)
• 4) Escalate when progressive / advanced (e.g., fistula, pathologic fracture, Notani III)
• If conservative care fails or disease progresses, surgical management (debridement → segmental resection with reconstruction as needed) is typically required. (eviq.org.au)
• 5) Hyperbaric oxygen (HBOT): selective, not routine
• Contemporary guidance notes HBOT for prevention/management is largely not justified overall, with limited supportive evidence. (PubMed)
• Some protocols suggest it *may* help selected high-risk subgroups or socket healing in irradiated tissue, but it isn't a blanket recommendation. (eviq.org.au)
• Bottom line: In this 55-year-old post-radiotherapy patient with exposed mandibular bone and pain weeks after 38 extraction, think ORN and manage via early specialist referral + staging/imaging + conservative measures first, escalating to surgery if progressive, with HBOT only in selected cases. (eviq.org.au)

16. In a 62-year-old patient taking warfarin, a mandibular right 48 extraction is performed without checking INR, followed by uncontrolled bleeding. What mechanism explains this bleeding, and what treatment should be applied?

• This is warfarin-related coagulopathy.
• Mechanism (why the bleeding won't stop)
• Warfarin inhibits vitamin K epoxide reductase (VKORC1) → vitamin K can't be recycled → impaired γ-carboxylation of vitamin K–dependent clotting factors II, VII, IX, X (and proteins C/S) → prolonged PT / elevated INR → insufficient fibrin clot formation after extraction → persistent oozing/bleeding.
• Treatment (what to do now)
• Manage in two layers: local control first, then systemic reversal only if needed.
• 1) Immediate local hemostasis (first-line in the dental chair)
• Suction + identify source (socket ooze vs. arterial spurting).
• Firm gauze pressure continuously 20–30 min (don't keep peeking).
• Local anesthetic with epinephrine (if appropriate) to aid vasoconstriction.
• Socket debridement/irrigation to remove loose clot, then:
o Pack socket with a local hemostatic: oxidized cellulose (Surgicel), gelatin sponge

(Gelfoam), collagen plug, etc.
o Figure-of-eight sutures to secure the pack.
• Tranexamic acid (TXA):
o Soak gauze in TXA and bite pressure, and/or
o TXA mouthwash (commonly used as 4.8–5%): hold in mouth then spit, several times daily for a few days (per local protocol).
• Post-op instructions: no spitting/rinsing for 24 h, soft/cool diet, avoid smoking/alcohol, head elevation.
• 2) Assess severity + check coagulation urgently
• Obtain INR ASAP (same day).
• If bleeding is uncontrolled despite proper local measures, the patient is unstable, or INR is markedly high → urgent medical/ED referral and coordinate with prescribing physician.
• 3) Systemic management (only when indicated)
• If significant / ongoing bleeding or supratherapeutic INR, medical team may give:
o Vitamin K (slower onset), and for serious bleeding:
o 4-factor prothrombin complex concentrate (PCC) (rapid reversal) ±
o Fresh frozen plasma (FFP) if PCC unavailable.
• This is typically hospital-based care.
• Practical key point
• The mistake here is extracting without knowing INR. In planned extractions, warfarin patients are usually treated safely when INR is within an acceptable range and with good local hemostasis—*but you must check it close to the procedure* and follow your institution's protocol.
• If you want, tell me what the patient's current status is (oozing vs spurting, vitals, estimated blood loss) and I'll map it to a "chairside vs immediate ED" pathway.

17. In a 30-year-old patient, after mandibular 48 extraction, painful oral erosions and cutaneous target lesions develop following use of ibuprofen and amoxicillin. Which drug reaction does this suggest, and how should it be treated?

• This presentation (painful oral erosions + classic *target* skin lesions soon after starting ibuprofen and amoxicillin) is most consistent with a drug-triggered erythema multiforme (EM)—often termed EM major when oral mucosa is involved.
What to do (management)
1. Immediately stop the suspected trigger drugs
a. Discontinue amoxicillin and ibuprofen (and avoid re-exposure until formally evaluated).
2. Assess severity to rule out SJS/TEN (the dangerous mimics)
a. EM: raised "target" lesions, often acral; mucosal involvement can occur (usually limited). b. SJS/TEN red flags: diffuse blistering/skin detachment, dusky or atypical targets, severe systemic symptoms (fever, malaise), multiple mucosal sites (eyes/genitals), rapidly progressive disease.
If any of these are present → urgent hospital referral.
3. Supportive care (core treatment)
a. Pain control: prefer paracetamol/acetaminophen (avoid NSAIDs until clarified). b. Oral care: bland rinses (saline / sodium bicarbonate), soft diet, hydration. c. Topical therapy for mouth: high-potency topical corticosteroid (e.g., dexamethasone mouth rinse or clobetasol gel in orabase) + topical anesthetic (e.g., lidocaine) as needed. d. Antihistamine can help itch if present.
4. Systemic therapy (if significant mucosal disease or extensive skin)
a. Many clinicians use a short course of systemic corticosteroid early in EM major (e.g., prednisone), especially when oral intake is compromised—ideally coordinated with dermatology. b. If severe dehydration/inability to eat → IV fluids and inpatient care.
5. Follow-up / safety
a. Document as suspected drug reaction; arrange dermatology/allergy follow-up. b. If antibiotics are still needed for the extraction site, choose an alternative only if clinically indicated and after clinician review (often they're not needed unless there's clear infection).
Immediate ER referral if: eye pain/redness, difficulty swallowing/breathing, genital erosions, fever/systemic illness, rapidly spreading rash, blisters, or any skin peeling.

18. In a 27-year-old patient, during surgical extraction of an impacted tooth 28, a portion of the maxillary tuberosity fractures and separates along with the tooth. What surgical and postoperative management should be performed?

• Plan it like a dentoalveolar fracture / maxillary tuberosity fracture with possible oroantral communication risk.
• Intra-operative (surgical) management
• Stop traction immediately
• Don't keep pulling—this can propagate the fracture posteriorly and tear soft tissue or open the sinus.
• Assess the fragment
o Size/mobility of the tuberosity segment
o Soft-tissue attachment (is it still vascularized via periosteum?)
o Sinus involvement: check for an oroantral communication (OAC) (Valsalva gently, visual inspection), and evaluate bleeding.
• Decision: remove vs reposition
• A) Small, non–strategic fragment (minimal tuberosity, not needed for denture support, no significant sinus involvement):
o Remove the tooth + small bone fragment if necessary
o Smooth sharp edges (alveoloplasty as needed)
o Irrigate, achieve hemostasis, primary closure.
• B) Large tuberosity segment (especially if it's a substantial piece, still attached to mucoperiosteum, important for posterior support / future prosthesis, or close to sinus):
o Preserve and reposition the segment back into place (treat as a fractured bone plate)
o Stabilize:
▪ Firm mucoperiosteal closure with interrupted sutures (often enough if stable), and/or
▪ Splinting to adjacent teeth (e.g., wire/composite splint) for ~2–4 weeks if mobile, and/or
▪ Rigid fixation (miniplate/screws) rarely, only if very unstable/large.
o If the tooth is still present and keeping it helps stability, you can section the tooth and remove it with minimal force; if removal would destabilize the segment, consider leaving the tooth/root temporarily and planning staged removal later once the segment is stable (case-dependent).
• If there is an oroantral communication
o Do not curette aggressively into the socket/sinus.
o Close primarily with a tension-free flap:
▪ Most commonly a buccal advancement flap, or
▪ Buccal fat pad flap if larger posterior defects or poor tissue.
o Consider placing resorbable collagen in the socket if helpful (avoid packing that prevents closure).
• Post-operative management
• Medications
o Analgesics/NSAIDs as appropriate
o Antibiotics if OAC/sinus exposure or large fracture (commonly amoxicillin-clavulanate; clindamycin if penicillin-allergic—local protocols apply)
o Nasal decongestant (short course) if OAC/sinus involvement
o Chlorhexidine mouth rinse (starting 24 hours post-op)
• Sinus precautions (if suspected/confirmed OAC or sinus proximity)
o No nose blowing, no forceful sneezing (sneeze with mouth open), avoid straws/smoking, avoid heavy lifting for ~10–14 days.
• Diet & function
o Soft diet, avoid chewing on that side
o If splinted/fixated: limit function until stable.
• Follow-up
o Early review in 48–72 hours (swelling, bleeding, flap integrity)
o Re-evaluate for sinus symptoms (fluid passage to nose, whistling, unilateral congestion, fetor)
o Suture removal typically 7–10 days (unless resorbable)
o If splinted, reassess and remove at 2–4 weeks depending on stability.
• Red flags requiring prompt reassessment
• Persistent epistaxis, increasing posterior maxillary pain/swelling, fever, purulent nasal discharge, clear "air/fluid" communication mouth↔nose, or a mobile painful tuberosity segment.
• If you want, tell me whether the fractured tuberosity piece is small vs large, and whether

there was any OAC, and I'll map the exact "best pathway" for that specific scenario.

19. In a 29-year-old patient, several weeks after extraction of tooth 18, unilateral nasal obstruction, foul-smelling yellow-green discharge, and facial pain develop. Which complication does this suggest, and how should it be managed?

• This presentation weeks after extracting tooth 18 (upper right third molar)— unilateral nasal obstruction + foul-smelling yellow-green discharge + facial/facial sinus pain— is most consistent with odontogenic maxillary sinusitis, very often due to an oroantral communication that has epithelialized into an oroantral fistula (OAF).

• What it suggests

• Chronic odontogenic maxillary sinusitis (right maxillary sinus), typically from:

o a persistent oroantral communication/fistula, and/or

o retained debris/root fragment/foreign body in the sinus.

• Management (practical pathway)

• Clinical confirmation

o Intraoral exam for a fistula at the extraction site (granulation "pinhole," fluid/air passage).

o Gentle tests (avoid aggressive Valsalva if it risks enlarging the defect).

• Imaging

o CBCT (best to identify: sinus opacification, bony defect, retained root/foreign body).

• Medical therapy (start early)

o Antibiotics with anaerobic coverage for odontogenic sinusitis (choice depends on local guidelines/allergy; common options include amoxicillin-clavulanate; clindamycin if penicillin-allergic).

o Nasal saline irrigation, topical nasal decongestant short course, and/or intranasal steroid.

o Sinus precautions: no nose-blowing, sneeze with mouth open, avoid straws/smoking.

• Definitive treatment (if OAF/persistent symptoms)

o If an oroantral fistula is present or symptoms persist beyond ~2–3 weeks, surgical closure is usually indicated:

▪ Buccal advancement flap (common first-line),

▪ Palatal rotational flap (good tissue quality),

▪ Buccal fat pad flap (especially for larger/posterior defects).

o Remove any foreign body/root fragment if present.

• ENT collaboration

o If there is significant chronic disease, blockage of the ostiomeatal complex, polyps, or failure of closure/medical therapy → ENT for functional endoscopic sinus surgery (FESS) alongside dental/oral surgical management.

• If you want, I can turn this into a short exam-style answer (diagnosis + 5-step management) like your other complication questions.

•

20. In a 24-year-old patient, during difficult extraction of a deeply impacted 48, the tooth slips from the elevator and is lost intraorally, followed by coughing and a foreign-body sensation in the throat. What condition does this most strongly suggest, and how should it be managed?

• This presentation most strongly suggests aspiration of the extracted tooth (or a fragment) into the airway (larynx/trachea/bronchus), rather than simple swallowing— because the tooth is "lost," followed immediately by coughing and a foreign-body sensation.

• Immediate management (chairside)

• Stop the procedure and keep the patient upright.

• Assess airway and breathing: SpO_2, respiratory rate, voice/stridor, wheeze, cyanosis.

• If the patient is actively choking / cannot speak / severe distress → treat as airway obstruction:

o Encourage forceful coughing if effective.

o If ineffective and obstruction is severe → abdominal thrusts (Heimlich); activate emergency response.

• If the patient is stable but aspiration is suspected (persistent cough, throat/foreign-body feeling, wheeze) → do not send home.

• Definitive evaluation and treatment

• Urgent imaging to localize the tooth:

o Chest radiograph (PA/lateral) ± neck soft-tissue radiograph.
o If not clearly seen (or high suspicion persists) → CT chest/neck.
• Urgent referral:
o Pulmonology / thoracic surgery (or ED) for suspected lower-airway aspiration.
o ENT if signs suggest upper-airway/hypopharyngeal lodgment.
• Removal:
o Bronchoscopy (rigid or flexible depending on setting and location) is the standard for airway foreign body retrieval.
• Aftercare:
o Monitor for complications (atelectasis, aspiration pneumonia, bronchospasm).
o Antibiotics are not automatic—given based on clinical findings (fever, infiltrate, purulence), plus supportive care as needed.
• Key practical point
• Even if the patient later feels "better," a tooth in the airway can migrate and cause delayed collapse/infection—so localize it and remove it urgently.
• If you want, tell me whether the coughing is persistent and whether there's any wheeze/stridor—I can help you decide whether ENT vs pulmonology should be the first call in your setting.

21. In a 27-year-old patient, during an inferior alveolar nerve block for mandibular right 48, the needle fractures and is lost within the pterygomandibular space. By what mechanism can this occur, and how should it be treated?

• This typically happens when a thin needle breaks at/near the hub (the weakest point) after it has been advanced deeply into tissue.
How it can occur (mechanism)
Most common contributing factors:
• Needle inserted all the way to the hub → highest bending stress concentrates at the hub and the needle can snap there.
• Sudden patient movement (startle, gag/cough, unexpected jaw closing) while the needle is engaged in tissue → the needle is forced to bend and fractures.
• Intentional pre-bending of the needle (or repeated redirection within tissue) → metal fatigue and stress concentration.
• Using very small-gauge needles (e.g., 30G) for IANB increases flexibility and risk of deformation; fracture is still rare, but risk is higher than with 25–27G.
• Less commonly: manufacturing defect or damage from prior handling.
What to do (treatment / management)
This is managed as a foreign body in a deep fascial space with potential migration risk.
Immediate chairside steps
• Stop the injection immediately.
• Do not blindly probe with instruments/fingers (can push it deeper).
• If the fragment is visible in the mouth, attempt gentle retrieval with a hemostat *only if you can clearly grasp it*.
• Document the event and arrange urgent OMFS referral.
Localization (same day if possible)
• Obtain imaging to localize precisely:
o CBCT is often most useful for 3D localization in the pterygomandibular space.
o Panoramic/periapical films can help but are less precise.
Definitive treatment
• Early surgical removal is usually recommended, especially if:
o the fragment is deep (not visible),
o there are symptoms (pain, trismus, infection),
o it's close to neurovascular structures,
o there's concern for migration.
Removal is typically performed by oral & maxillofacial surgery, often via an intraoral approach with careful blunt dissection; in difficult cases, surgeons may use image guidance / intraoperative radiography and sometimes general anesthesia.
When observation is considered
Occasionally, if the fragment is very small, deeply embedded, stable on imaging, and completely asymptomatic, a surgeon may consider close observation—but this is case-dependent, and many teams still prefer removal because migration/infection can occur.
If you want, tell me what gauge/length needle was used and whether the fragment was visible at any point—I'll map the most likely fracture point and the typical surgical access route.

22. In a 19-year-old patient, during impacted 48 surgery, cyanosis, fatigue, and low oxygen

saturation develop after administration of prilocaine and articaine. What does this indicate, and how should it be treated?

• This picture is most consistent with local-anesthetic–induced methemoglobinemia (classically from prilocaine; less commonly reported with other local anesthetics, including articaine). It causes cyanosis + fatigue/dyspnea with low SpO_2 that improves poorly despite oxygen, because hemoglobin is oxidized to methemoglobin and can't carry O_2 effectively.
• What it indicates
• Methemoglobinemia during/after LA injection
• Typical clues:
o Sudden gray/blue cyanosis and SpO_2 often ~80–88%
o Disproportionately normal PaO_2 on ABG (if measured)
o Chocolate-brown blood (sometimes noted)
o Diagnosis confirmed by co-oximetry (methemoglobin level)
• Treatment (clinical pathway)
• Stop the procedure and stop further local anesthetic.
• High-flow 100% oxygen + ABC support, monitor ECG/BP/SpO_2.
• Confirm with co-oximetry (preferred) and/or ABG if available (don't rely on pulse oximetry alone).
• If symptomatic (like your case) *or* metHb typically ≥20–30%:
o Methylene blue 1–2 mg/kg IV (0.1–0.2 mL/kg of a 1% solution) over ~5 minutes
o Recheck in 30–60 min; if needed, repeat 1 mg/kg (common max total ~7 mg/kg to avoid paradoxical worsening/hemolysis).
• If methylene blue is contraindicated (e.g., G6PD deficiency, or high risk of hemolysis; also be cautious with serotonergic drugs due to serotonin syndrome risk):
o Ascorbic acid (vitamin C) IV/PO as an alternative reducing agent
o Severe/refractory cases: exchange transfusion and/or hyperbaric oxygen in an ICU setting.
• After stabilization
• Document as methemoglobinemia from local anesthetic exposure and avoid high-risk agents in the future (especially prilocaine; caution with benzocaine/topicals too).
• Arrange follow-up and consider screening for G6PD deficiency if methylene blue is being considered or if hemolysis occurs.
• If you want, I can turn this into a short "exam-style" management algorithm (what to do first, what lab confirms it, exact dosing) for your question bank.

23. In a 45-year-old patient with uncontrolled diabetes, increasing pain, fistula formation, foul odor, and sequestrum formation develop a few weeks after extraction of mandibular left 37 and 38. What condition does this suggest, and how should it be treated?

• This presentation is most consistent with chronic suppurative osteomyelitis of the mandible (post-extraction osteomyelitis with sequestration)—uncontrolled diabetes is a major risk factor, and foul odor + fistula + sequestrum strongly point to necrotic infected bone.
• Treatment approach (practical OMFS pathway)
• 1) Stabilize the patient / control the cause
• Urgent glycemic control (coordinate with internal medicine/endocrinology). Antibiotics and healing will fail if hyperglycemia persists.
• Assess for systemic toxicity (fever, malaise), dehydration, ketoacidosis risk.
• 2) Confirm extent and guide surgery
• CBCT/CT to define sequestrum, involucrum, medullary involvement, cortical perforations.
• Culture and sensitivity from deep bone/pus (not just surface swab). Labs: CBC, CRP/ESR.
• 3) Start antimicrobial therapy (then tailor)
• Begin empiric IV antibiotics covering mixed oral flora (aerobes + anaerobes), then adjust to culture.
o Common empiric options (institution-dependent): ampicillin–sulbactam or piperacillin–tazobactam; add MRSA coverage only if risk/clinical suspicion.
• Plan for prolonged therapy (often 4–6+ weeks, IV then oral depending on response and cultures).
• 4) Definitive surgical management
• Incision & drainage if abscess/collections are present.
• Sequestrectomy + thorough debridement/curettage to bleeding viable bone.
• Remove any remaining local irritants (sharp spicules, foreign bodies, residual roots).

• If disease is extensive/refractory: decortication or segmental resection with reconstruction may be needed.
• 5) Adjuncts and follow-up
• Optimize nutrition, smoking cessation if relevant, meticulous oral hygiene and antiseptic rinses.
• Consider hyperbaric oxygen as an adjunct in selected refractory cases (center- and protocol-dependent).
• Close follow-up to ensure pain, drainage, odor, and inflammatory markers resolve; repeat imaging if not improving.
• Red flags needing urgent escalation: rapidly spreading swelling, trismus, dysphagia, airway symptoms, facial/neck cellulitis, sepsis signs—treat as a deep space infection emergency alongside osteomyelitis management.

24. In a 23-year-old patient, after local anesthesia for tooth 28, it is learned that the patient has a history of drug allergy and atopic disease . Shortly after injection, generalized pruritus–urticaria, lip/face swelling, wheezing, and dizziness develop. How would you evaluate this presentation in terms of the most likely diagnosis, and how would you plan the emergency management in a dental clinic?

• This is most consistent with an acute IgE-mediated anaphylaxis (systemic allergic reaction) triggered *around the time of* the local anesthetic injection.
• Why this fits best:
• Skin/mucosa: generalized pruritus–urticaria + lip/face swelling (angioedema)
• Respiratory: wheezing (bronchospasm)
• Circulatory/neurologic: dizziness (suggests hypotension/impending shock)
• Timing: “shortly after injection”
• Risk factors: atopy + prior drug allergy history
• Rapid differential (in case someone questions it)
• Vasovagal syncope: pallor, bradycardia, nausea; *not* urticaria/angioedema/wheezing.
• Local anesthetic toxicity: CNS symptoms (tinnitus, metallic taste, seizures) and arrhythmias; *not* urticaria/angioedema.
• Methemoglobinemia (e.g., prilocaine): cyanosis, low SpO_2; *not* urticaria/angioedema.
• So: treat as anaphylaxis until proven otherwise.
• Emergency management plan in a dental clinic (step-by-step)
• 1) Recognize + call for help immediately
• Stop dental treatment, remove instruments, call emergency medical services (EMS/112).
• Assign roles (airway/meds/vitals/documentation).
• 2) Position + oxygen
• Supine with legs elevated (to support blood pressure).
o If vomiting or pregnant → left lateral recovery position.
o If severe dyspnea → allow sitting position but watch for hypotension.
• High-flow oxygen via mask (10–15 L/min).
• 3) First-line medication: IM epinephrine (do not delay)
• Give IM epinephrine into the mid-anterolateral thigh.
• Adult dose (23-year-old):
• 0.5 mg IM = 0.5 mL of 1 mg/mL (1:1000)
• Repeat every 5 minutes if symptoms persist/worsen (airway, breathing, BP).
• Key points
• IM thigh is preferred for speed/reliability.
• Do not substitute antihistamines or steroids for epinephrine.
• If patient is on beta-blockers or is refractory, EMS/ED may use IV epinephrine and glucagon, but that’s typically beyond a dental office’s scope.
• 4) Airway and breathing support
• Look for stridor, voice change, progressive facial/tongue swelling, inability to speak full sentences, dropping SpO_2.
• If wheezing:
o Salbutamol (albuterol) via inhaler with spacer: 4–10 puffs, repeat as needed
• *(or nebulized 2.5–5 mg if available).*
• Prepare for airway deterioration; keep suction ready.
• 5) Circulation: monitor vitals + consider IV fluids
• Continuous observation: BP, HR, RR, SpO_2, mental status.
• If hypotension/shock signs:
o Establish IV access if trained and equipped.
o Rapid isotonic crystalloid bolus (e.g., normal saline) while awaiting EMS.

• 6) Adjuncts (after epinephrine)
• These help symptoms and reduce biphasic/protracted reactions, but they are not lifesaving first-line.
• H1 antihistamine (urticaria/itch):
o e.g., diphenhydramine 25–50 mg IM/IV *(or other available H1)*
• Corticosteroid (late-phase reaction):
o e.g., hydrocortisone 200 mg IV/IM *(or equivalent per local availability/protocol)*
• 7) Observation + transfer
• Even if the patient improves, they must be transferred to ED for monitoring due to risk of biphasic anaphylaxis.
• Typical observation guidance often falls in the 4–6+ hour range, longer for severe reactions, multiple epinephrine doses, hypotension, or asthma history—ED will decide.
• 8) After the event (documentation + prevention)
• Document: suspected trigger (drug name, preservative, latex, chlorhexidine, etc.), times, vitals, meds/doses, response.
• Advise follow-up with allergy/immunology for evaluation and future anesthetic planning (true allergy to amide local anesthetics is rare; reactions can be to preservatives like sulfites in epinephrine-containing solutions or to latex/chlorhexidine—specialist workup matters).
• If possible, ensure the patient leaves ED with guidance on an epinephrine auto-injector and an emergency action plan.
• Quick "clinic-ready" checklist
• Stop procedure + 112/EMS
• Supine legs up + O_2 10–15 L/min
• IM epinephrine 0.5 mg thigh; repeat q5 min if needed
• Salbutamol for wheeze
• Monitor vitals + IV fluids if hypotensive
• Antihistamine + steroid after epi
• ED transfer for observation
• If you want, I can format this as a one-page dental clinic anaphylaxis protocol (with drug concentrations and "mL to draw up" quick conversions) tailored to what's typically stocked in your setting.

25. A 29-year-old patient presents two weeks after extraction of tooth 18 with the complaint that liquids consumed come out through the nose. CBCT shows an oroantral communication measuring 6 mm. What complication does this represent, and how should it be treated?

This represents a persistent oroantral communication (OAC), likely evolving into an oroantral fistula (OAF) because the defect has persisted for approximately two weeks and the patient reports passage of fluids from the oral cavity into the nose. A 6-mm defect is unlikely to close spontaneously. Management consists of surgical closure of the communication after confirming that the maxillary sinus is free of active infection. Any epithelialized fistulous tract should be excised and the margins refreshed. For a defect of this size in the posterior maxilla, a buccal advancement flap (Rehrmann flap) is a commonly used first-line technique. Depending on the location and available tissue, a palatal rotational flap or buccal fat pad flap may also be used.
If maxillary sinusitis is present, the sinus infection should be treated before or in conjunction with closure, using appropriate antibiotics, nasal decongestants, and sinus drainage when indicated. Postoperatively, the patient should be instructed to follow sinus precautions: avoid nose blowing, sneezing with the mouth closed, smoking, use of straws, and activities that increase sinonasal pressure.
Diagnosis: Persistent oroantral communication / developing oroantral fistula after extraction of tooth 18.
Treatment: Surgical closure, most commonly with a buccal advancement flap, with management of any associated maxillary sinus infection.

26. In a 52-year-old patient with uncontrolled diabetes, five days after bilateral mandibular 38 and 48 extractions, tongue swelling, dyspnea, and "board-like" induration/swelling of the neck develop. Which life-threatening infection does this suggest, and what is the appropriate emergency management?

• I'll identify the most likely diagnosis from the red-flag symptoms and then lay out a step-by-step emergency management plan (airway first, then source control + antibiotics + ICU support).
• This presentation (uncontrolled diabetes + 5 days post–mandibular third molar extractions + tongue swelling, dyspnea, and "board-like" woody induration of the neck) most strongly

suggests Ludwig's angina — a rapidly spreading cellulitis of the submandibular/sublingual/submental spaces with high risk of acute airway obstruction.
• Emergency management (priorities)
• 1) Airway is the first emergency
• Treat as an impending difficult airway.
• Call anesthesia + ENT/OMFS and move to resuscitation bay/ICU-capable setting.
• Keep patient upright, give high-flow oxygen, continuous monitoring.
• Avoid sedation/paralysis until a secured plan is in place (can precipitate total obstruction).
• Definitive airway (choose the safest in expert hands):
• Awake fiber-optic intubation (often preferred if feasible).
• If intubation is unsafe or fails → awake tracheostomy (or emergency surgical airway per local protocol).
• 2) Immediate broad-spectrum IV antibiotics (don't wait for imaging)
• Cover polymicrobial oral flora: streptococci + anaerobes, and in diabetics consider broader gram-negative coverage; add MRSA coverage if risk factors/high local prevalence.
• Common empiric regimens (examples):
• Ampicillin–sulbactam IV *or* piperacillin–tazobactam IV
• If severe penicillin allergy: clindamycin + (e.g., levofloxacin/ceftriaxone alternative per allergy profile)
• Add vancomycin (or local equivalent) if MRSA coverage is needed.
• 3) Source control / surgical management
• Urgent OMFS/ENT evaluation for incision and drainage if abscess is present or if there's progression despite antibiotics.
• Remove the odontogenic source if not already controlled; irrigate/debride as indicated.
• Send cultures (aerobic/anaerobic) during drainage, then de-escalate antibiotics to culture results.
• 4) Imaging once airway is secure
• Contrast CT neck to define extent (submandibular spaces, parapharyngeal/retropharyngeal spread, gas, abscess).
• 5) Supportive critical care
• Sepsis bundle: IV fluids guided by vitals/lactate, blood cultures, labs.
• Tight glucose control (IV insulin protocol) — uncontrolled diabetes worsens outcomes.
• Analgesia, antipyretics; consider DVT prophylaxis once stable.
• Steroids are sometimes used to reduce edema, but never as a substitute for airway control + antibiotics + drainage, and should be decided by the treating team.
• Red flags that mandate "act now"
• Stridor, rapidly increasing tongue/floor-of-mouth swelling, inability to handle secretions, muffled "hot potato" voice, worsening dyspnea, hypoxia.
• Bottom line: This is Ludwig's angina until proven otherwise — manage as a life-threatening deep neck space infection with early airway control, immediate IV broad-spectrum antibiotics, and urgent surgical source control, with ICU-level monitoring.

27. In a 22-year-old patient, after an inferior alveolar nerve (IAN) block administered for mandibular right 48, difficulty closing the eye and drooping of the corner of the mouth are observed. How can the cause of this finding be explained, and how should it be managed?

• This presentation is most consistent with a transient facial nerve palsy (Bell's palsy–like weakness) caused by the local anesthetic reaching the facial nerve, not an injury to the IAN.
• Why it happens (mechanism)
• During an IAN block, if the needle is placed too posterior/lateral (toward the parotid gland) and anesthetic is deposited there, it can bathe the facial nerve trunk (or its branches) as it exits the stylomastoid foramen and passes through the parotid.
• That produces ipsilateral inability to close the eye (orbicularis oculi weakness) and drooping of the mouth corner (orbicularis oris/buccinator weakness).
• Key point: This is typically immediate and temporary, resolving as the anesthetic wears off (often within a few hours).
• Management (what to do)
• Stop the procedure (or postpone if needed) and reassure the patient: this is usually reversible.
• Protect the eye until function returns:

o Artificial tears/lubricating drops.
o Eye patch or tape the eyelid closed (especially if blinking is poor).
• Safety advice
o Avoid driving until facial control/vision is normal.
• Document the event (signs, time of onset, anesthetic used, batch/amount, vitals).
• Follow-up
o Recheck within the same day (or next day).
o If weakness doesn't start improving as anesthesia should be wearing off (e.g., persists beyond ~6–8 hours) or lasts >24 hours, or if there are atypical features (progressive deficits, severe pain, vesicles, systemic neuro signs), refer urgently (OMFS/ENT/neurology) to rule out true facial nerve palsy or other pathology.
• Prevention (for next time)
• Correct landmarking: stay at the proper medial ramus target, avoid drifting posterior into parotid.
• Aspirate and inject slowly; avoid deep/posterior deposition.
• If you want, tell me whether the weakness was immediate and whether the patient also had loss of forehead wrinkling on that side—those details help confirm it's anesthetic spread vs a different facial nerve issue.

28. In a 27-year-old patient, within a few days after a difficult 28 extraction, temporomandibular joint clicking, locking, and pain develop. By what mechanism can this be explained, and how should it be managed?

• I'll explain what's most likely happening in the TMJ after a prolonged/difficult upper 2nd molar (28) extraction, then give a practical step-by-step management plan (including what to do if there's an acute "closed lock").
• Mechanism (why it happens after a difficult extraction)
• A difficult extraction often involves prolonged maximal mouth opening, strong retraction, and sometimes the operator using the mandible as a "lever point." That can overload the TMJ and masticatory muscles and lead to:
• TMJ internal derangement (disc displacement)
o Clicking → commonly disc displacement with reduction (the disc slips forward then "reduces" back with opening/closing, producing a click).
o Locking (closed lock) → often disc displacement without reduction (disc stays displaced; the condyle can't translate normally, so the jaw "catches"/locks and range of motion drops).
• Capsulitis/synovitis (inflammation of the joint capsule/synovium) from overload/microtrauma → pain, tenderness, pain on function.
• Protective muscle spasm / myofascial pain (masseter/temporalis/pterygoids) from prolonged strain → pain + limited opening that can mimic locking.
• In the first few days after surgery, the most common explanation for *clicking + pain ± intermittent locking* is acute TMJ overload leading to inflammation and disc displacement rather than a primary "joint disease."
• Management (practical approach)
• 1) First: rule out things that change management
• True mandibular dislocation (mouth stuck *open* and cannot close) → needs urgent reduction.
• Infection spreading (fever, increasing swelling, worsening trismus day by day, dysphagia) → manage as infection.
• If it's mainly *clicking/locking/pain* without those red flags, proceed conservatively.
• 2) Initial conservative treatment (first 1–2 weeks)
• This is first-line for most post-extraction TMJ symptoms:
• Patient education / joint rest
o Soft diet, small bites
o Avoid wide opening (yawning, big sandwiches)
o No gum chewing, no clenching
• Analgesia / anti-inflammatory
o NSAID if not contraindicated (often best for capsulitis/synovitis)
o Add acetaminophen if needed (depending on your local protocol/contraindications)
• Local measures
o Warm compresses (or alternating warm/cold if acute soreness)
• Physiotherapy
o Gentle, pain-free range-of-motion exercises (controlled opening, lateral excursions) several times/day

o Posture and relaxation exercises
• Short course muscle relaxant at night can help if spasm is prominent (case-dependent).
• Occlusal splint (night guard) if symptoms persist or bruxism/clenching is suspected.
• 3) If there is an acute “closed lock” (disc displacement without reduction)
• Clues: sudden limited opening (often ~25–30 mm or less), deviation to the affected side, hard end-feel, patient says “it gets stuck.”
• Early manual manipulation (by someone experienced) can sometimes “unlock”/recapture function in the acute phase.
• If not improving quickly (days–1 week) or significant limitation persists:
o Consider MRI to assess disc position if diagnosis is uncertain or symptoms persist.
o Arthrocentesis (lavage) ± intra-articular medication is a common next step when conservative care fails and function is limited.
• 4) When to escalate / refer
• Refer to an OMFS/TMJ clinic if:
• Persistent locking or significant limitation beyond ~1–2 weeks despite conservative care
• Severe pain not responding to NSAIDs/rest
• Recurrent locking episodes
• Suspicion of structural injury, or you need imaging/arthrocentesis
• 5) What not to do
• Don’t force repeated wide opening (it can worsen inflammation/derangement).
• Avoid “hard” jaw stretching into sharp pain early on.
• What to document/assess in clinic (quick checklist)
• Max interincisal opening (mm), deviation/deflection pattern
• Joint sounds (click vs crepitus), palpation tenderness
• Muscle tenderness (masseter/temporalis)
• Occlusion changes (rare but important)
• Pain on loading (bite test) and functional movements
• If you want, I can convert this into a short exam-style answer (2–3 lines) or a more detailed clinical pathway (with decision points for click vs closed lock vs dislocation).

29. In a 28-year-old patient, during mandibular left 38 surgery, prolonged use of a bur with inadequate water cooling results in a localized area of necrotic bone. Which complication is this consistent with, and how should it be managed?

• I’ll identify the most likely diagnosis from the scenario and then lay out a practical OMFS-style management plan (immediate steps, conservative care, when to debride/resect, meds, and follow-up).
• This is most consistent with iatrogenic thermal bone necrosis (a localized osteonecrosis / sequestrum caused by heat injury from prolonged burring with insufficient irrigation). Clinically it can behave like a small, localized “post-surgical osteitis/osteonecrosis” that may later sequestrate and become secondarily infected.
• Management (practical pathway)
• 1) Assess severity
• Small, localized exposed necrotic area, mild symptoms, no spreading infection → start conservative care.
• Increasing pain, foul taste/odor, purulence, swelling, systemic signs, or enlarging exposed bone → treat as secondary infection/osteomyelitis risk and escalate.
• 2) Initial conservative treatment (first-line for localized lesions)
• Irrigation of the socket/wound and gentle debridement of loose debris (avoid aggressive curettage early).
• Antiseptic rinses: chlorhexidine 0.12–0.2% (commonly 2×/day for ~1–2 weeks).
• Analgesia (e.g., NSAID ± acetaminophen as appropriate).
• Avoid trauma to the area (no picking; soft diet; excellent oral hygiene).
• 3) If symptomatic or signs of secondary infection
• Culture if drainage is present (helpful if not responding).
• Antibiotics when there is cellulitis, purulence, fever, or progressive pain/swelling:
o Typical odontogenic coverage: amoxicillin–clavulanate or (if penicillin allergy) clindamycin (local protocols apply).
• Re-evaluate in 48–72 hours if infection suspected.
• 4) Definitive local management if necrotic bone persists
• If a sequestrum forms or there is persistent exposed necrotic bone after a short conservative period:
• Remove loose sequestrum / sequestrectomy (often minor).
• Debride to bleeding bone (limited saucerization) and smooth sharp margins.

• Copious irrigation.
• Primary closure with tension-free mucoperiosteal flap when feasible (or allow secondary healing if tiny and clean).
• 5) Imaging and escalation
• If pain is disproportionate, lesion enlarges, or symptoms persist >2–3 weeks, consider:
o CBCT to assess sequestrum extent / cortical involvement.
o Treat as evolving localized osteomyelitis if radiographic/clinical progression.
• Rarely, extensive cases need more aggressive debridement, prolonged antibiotics, and specialist follow-up.
• 6) Follow-up
• Close follow-up until mucosal coverage returns and symptoms resolve.
• Document exposed bone size, tenderness, discharge, and healing at each visit.
• Key point: This is not medication-related osteonecrosis (MRONJ) unless the patient has relevant risk factors (bisphosphonates/denosumab/antiangiogenics + exposed bone >8 weeks). In your scenario, the trigger is clearly thermal injury, so management is local hygiene + infection control + timely sequestrectomy/debridement if it doesn't self-resolve.

30. In a 23-year-old patient, intraosseous anesthesia is administered during mandibular 38 extraction. Ten days later, the patient presents with an unhealed area of exposed bone at the extraction site and increasing pain. Which complication is this most likely to indicate, and how should management be planned?

• I'd be most suspicious of localized ischemic osteonecrosis with secondary infection/sequestrum formation at the extraction site (i.e., post–intraosseous anesthesia–associated bone necrosis), rather than a routine delayed healing pattern.
• Dry socket (alveolar osteitis) can cause severe pain and an "empty socket," but it usually peaks 2–5 days post-extraction and doesn't typically present as a persistently unhealed exposed bony area at day ~10 with progressive worsening unless something else is going on (necrosis/infection).
• Most likely complication
• Aseptic/ischemic osteonecrosis of the alveolar bone (with possible early osteomyelitis) triggered by intraosseous injection factors (high local vasoconstrictor concentration/pressure → reduced perfusion) and then maintained by contamination/secondary infection.
• Management plan (practical pathway)
• 1) Reassess and triage (same visit)
• History: smoking, diabetes, steroids/immunosuppression, recent bisphosphonate/denosumab use (rare at 23 but ask), radiotherapy history, severe bruxism/trauma.
• Exam: size of exposed bone, surrounding erythema/pus, mobility of bony spicule, halitosis, fever, lymphadenopathy, worsening trismus.
• Red flags (fever, spreading swelling, systemic toxicity, progressive trismus, dysphagia) → treat as odontogenic infection/osteomyelitis and escalate urgently.
• 2) Imaging
• Start with periapical/panoramic.
• CBCT if pain is increasing, exposed bone persists, or you suspect sequestrum/cortical involvement.
• 3) Initial conservative care (first-line)
• Irrigate gently with saline (avoid aggressive curettage initially).
• Chlorhexidine 0.12–0.2% rinse (e.g., 2×/day for 7–14 days).
• Analgesia (NSAID ± acetaminophen as appropriate).
• Avoid traumatic manipulation of the socket; optimize oral hygiene.
• 4) Antibiotics — only if infection is likely
• Use if there is suppuration, cellulitis, fever, foul discharge, progressive swelling, or imaging suggests infection:
• Empiric options (adapt to local protocols/allergies): amoxicillin–clavulanate or amoxicillin + metronidazole; clindamycin if true penicillin allergy (weigh C. difficile risk).
• If not improving in 48–72 h, consider culture (if drainage present) and tailor therapy.
• 5) Sequestrum management (key point)
• If there's a loose bony spicule/sequestrum, remove it atraumatically (often immediate pain relief).
• If sequestrum is not loose, don't aggressively chase it early—wait for demarcation, then

perform limited sequestrectomy/superficial debridement.
• 6) Follow-up and escalation
• Review in 48–72 hours if significant pain/infection; otherwise weekly until mucosal coverage.
• If exposed bone persists beyond ~2–3 weeks, pain worsens, or radiographic changes progress → manage as localized osteomyelitis/osteonecrosis:
o Culture-directed antibiotics (longer course if osteomyelitis confirmed),
o Surgical debridement/sequestrectomy with minimal trauma,
o Consider referral to OMFS if not already in specialist care.
• 7) Patient instructions
• No smoking/vaping, avoid vigorous rinsing for 24 h after any dressing/debridement, soft diet, meticulous hygiene.
• Return immediately for swelling, fever, worsening trismus, dysphagia, or spreading pain.
• Bottom line: This presentation most strongly fits post–intraosseous injection– associated localized osteonecrosis (often with early secondary osteomyelitis). Start conservatively, image early if worsening, add antibiotics only when infection is evident, and remove loose necrotic bone—escalating to limited sequestrectomy/debridement if it persists or progresses.

GEMINI QUESTIONS AND ANSWERS:

1. In a 27-year-old patient, during surgical extraction of an impacted mandibular right third molar, an air-driven high-speed handpiece is used for bone removal. Shortly after the procedure, sudden swelling develops in the right cheek and submandibular region, with palpable crepitus on examination. What complication does this presentation suggest, by what mechanism does it occur, and how should it be managed?

Clinical Diagnosis
The sudden onset of swelling coupled with the pathognomonic sign of palpable crepitus (a crackling sensation under the skin) indicates Surgical Emphysema (also known as subcutaneous or tissue emphysema).
Mechanism of Injury
This complication is a direct iatrogenic result of utilizing inappropriate instrumentation for surgical bone removal.
Air Exhaust: Standard restorative air-driven high-speed handpieces exhaust compressed air forward directly into the cutting field to cool the bur.
Tissue Dissection: During the surgical extraction of an impacted third molar, a mucoperiosteal flap is elevated, exposing loose connective tissue and deep fascial planes.
Forced Injection: The high-speed turbine forcefully injects non-sterile compressed air under the flap and directly into the adjacent fascial spaces (such as the buccal, submandibular, pterygomandibular, and lateral pharyngeal spaces).
Crepitus: The palpable crepitus is the physical sensation of these trapped air pockets displacing and popping within the soft tissues.
Management Protocol
While surgical emphysema is often self-limiting, with the body resorbing the trapped nitrogen and oxygen over 3 to 10 days, it carries significant risks and requires careful, proactive management.
Airway Assessment: Immediately evaluate the patient for airway compromise. Severe cases can track inferiorly down the cervical fascial planes into the mediastinum (pneumomediastinum) or superiorly to the periorbital region.
Antibiotic Prophylaxis: Because the forced air acts as a vector, introducing oral microflora deep into the fascial spaces, there is a high risk of secondary cellulitis or deep space infection. A prophylactic course of broad-spectrum systemic antibiotics (e.g., Amoxicillin or Clindamycin) is strictly mandated.
Symptomatic Management: Provide standard analgesics for discomfort. Do not attempt to manually express or massage the swelling, as this can force the air deeper into adjacent anatomic spaces.
Observation and Escalation: Observe the patient closely. If signs of dyspnea, dysphagia, or chest pain develop, immediate referral to an emergency department for advanced airway management and chest radiography is required.
As a strict preventive standard of care, conventional air-driven handpieces must be avoided in oral surgery. Are you currently utilizing 45-degree rear-exhaust surgical handpieces or electric surgical motors to prevent this specific complication?

2. In a 25-year-old patient, during removal of a fractured apical root fragment after extraction of tooth 28, the fragment is displaced apically and separated from the socket. Radiographs show that the fragment has been displaced into the maxillary sinus. What clinical pathway should be followed in this situation?

• This scenario describes a classic complication involving the displacement of a root fragment into the maxillary sinus (antrum of Highmore) during the extraction of an upper third molar (Tooth 28 in FDI notation).

• Because the fragment has separated from the socket and is confirmed inside the sinus, attempting to retrieve it blindly through the extraction socket is contraindicated, as this risks enlarging the Oroantral Communication (OAC) or pushing the fragment further into the sinus ostium.

• Here is the recommended clinical pathway:

• 1. Immediate Intraoperative Management

• Cease Manipulation: Stop all attempts to retrieve the fragment through the socket. Blind suctioning or digging can damage the sinus membrane further or displace the fragment into the ostium (middle meatus).

• Document and Inform: Inform the patient of the complication.

• Referral: Unless the operator is an experienced Oral and Maxillofacial Surgeon (OMFS), the patient should be referred to an OMFS immediately.

• 2. Surgical Management (Root Retrieval)

• The standard of care for a young patient (25 years old) is removal of the fragment to prevent future chronic sinusitis or foreign body reaction. The surgeon will typically choose one of two approaches:

• Caldwell-Luc Procedure (Lateral Window Approach):

• An incision is made in the vestibule (gum line above the premolars/canine), and a small window is created in the lateral wall of the maxillary sinus. This allows excellent visibility to locate and remove the root fragment without traumatizing the extraction socket further.

• Functional Endoscopic Sinus Surgery (FESS):

• Increasingly, surgeons may use an endoscope introduced through the nose (transnasal) or through the small lateral window to visualize and retrieve the fragment with less morbidity.

• 3. Management of the Oroantral Communication (OAC)

• Since the root entered the sinus, there is a communication between the mouth and the sinus that must be sealed to prevent an Oroantral Fistula (OAF).

• Primary Closure: The extraction socket must be closed water-tight. This is most commonly achieved using a Buccal Advancement Flap (Rehrmann flap).

• Alternative: If the defect is large, a Buccal Fat Pad graft may be used.

• 4. Post-Operative Medical Management

• To ensure healing and prevent sinus infection, the patient must be placed on a strict regimen:

• Antibiotics: Broad-spectrum antibiotics (e.g., Amoxicillin/Clavulanate) to prevent sinusitis.

• Decongestants: Nasal sprays (e.g., Oxymetazoline) and/or systemic decongestants (pseudoephedrine) to keep the sinus ostium patent and allow drainage.

• Sinus Precautions: The patient must be instructed to avoid creating negative or positive pressure in the sinus for 2–4 weeks:

o No nose blowing.
o Sneeze with mouth open.
o No drinking through straws.
o No smoking.

3. In a 29-year-old patient, during extraction of a deeply impacted tooth 48 that is related to the inferior alveolar nerve, a fractured apical root fragment is displaced apically and separated from the socket during attempted retrieval. Radiographs show that the fragment has been displaced into the inferior alveolar nerve canal. What clinical pathway should be followed, and what should the treatment approach be?

• This is a complex complication requiring a cautious, strategic approach to minimize the risk of permanent injury to the inferior alveolar nerve (IAN). The primary goal is to avoid further displacement or nerve damage.

• Here is the recommended clinical pathway and treatment approach:

• 1. Immediate Intraoperative Management

• Stop Manipulation: Immediately cease any attempts to retrieve the fragment blindly. Further instrumentation can push the fragment deeper into the canal or compress the

nerve, causing mechanical and chemical injury.
• Visualize (if possible): If the fragment is clearly visible and loose without deeper instrumentation, careful retrieval can be attempted. However, since the prompt states it is already displaced and separated, do not probe blindly.
• Assess the Site: Gently irrigate the socket to clear debris, but avoid high-pressure irrigation that could force the fragment further.
• Close the Wound: If retrieval is not feasible immediately, suturing the extraction site loosely is often the safest interim step to protect the socket while a definitive plan is made.
• 2. Advanced Diagnostic Imaging
• Cone Beam CT (CBCT): Standard 2D radiographs (periapical or panoramic) are insufficient for determining the precise 3-D relationship between the root tip and the neurovascular bundle. A CBCT scan is mandatory to:
o Confirm the fragment is inside the canal.
o Determine if the fragment is compressing the nerve.
o Assess the integrity of the lingual cortical plate (displacement into the submandibular space is a differential to rule out).
• 3. Surgical Decision Making
• The treatment decision generally falls into two categories based on the risk-benefit analysis:
• Option A: Observation (Leave in Situ)
• This is often the preferred choice if the fragment is small (< 2-3 mm), uninfected, and not compressing the nerve.
• Rationale: The surgical trauma required to retrieve the tip may cause more damage (e.g., permanent paresthesia/anesthesia) than leaving the root tip alone.
• Protocol:
o Prescribe antibiotics and anti-inflammatory medication (corticosteroids) to reduce perineural edema.
o Monitor for infection or neural symptoms (pain, numbness) regularly.
o Inform the patient that the root tip may eventually migrate or become encased in bone (fibrosis/ossification).
• Option B: Surgical Retrieval
• This is indicated if the fragment is large, infected, mobile, or causing active compression/neuralgia.
• Referral: This procedure should strictly be performed by an Oral and Maxillofacial Surgeon (OMS).
• Technique: The surgeon may employ a lateral window approach (sagittal split or localized osteotomy) to access the canal and decompress the nerve without working through the socket, which offers poor visibility.
• Timing: If retrieval is necessary, it is often best done sooner (within 24-48 hours) to prevent fibrosis, provided the surgeon has proper imaging and expertise.
• 4. Patient Communication & Legal Documentation
• Inform the Patient: Be transparent. Explain that a piece of the root has moved and that "fishing" for it carries a high risk of permanent lip/chin numbness.
• Documentation: Record the incident in detail in the clinical notes, including the sequence of events, the decision to stop, and the radiographic findings.
• Follow-up: Check for signs of neurosensory deficit (paresthesia, hypoesthesia, or anesthesia) of the lip and chin the next day. If the nerve was crushed, referral for nerve repair (microsurgery) might be required.
• Summary of Clinical Pathway
• Stop procedure immediately.
• Verify position with CBCT.
• Refer to an Oral and Maxillofacial Surgeon.
• Monitor for sensory deficit.
• Would you like me to outline the specific details that should be included in the referral letter to the Oral Surgeon?

4. In a 28-year-old patient using oral contraceptives, severe pain and a foul odor develop a few days after extraction of tooth 38. Which postoperative complication is this most consistent with, and how should treatment be planned?

• Based on the clinical presentation and history, the most consistent postoperative complication is Alveolar Osteitis, commonly known as a Dry Socket.
• Diagnosis: Alveolar Osteitis (Dry Socket)
• This diagnosis is supported by the specific combination of symptoms and risk factors

presented:
• Timing: The onset of severe pain occurring 2–4 days after extraction is the hallmark timing for a dry socket.
• symptoms: The "foul odor" (halitosis) and bad taste result from the disintegration of the blood clot and the accumulation of food debris in the empty socket.
• Site: Tooth 38 (the lower left third molar) is the most common site for this complication.
• Risk Factor (Oral Contraceptives): This is a critical detail. Patients taking oral contraceptives have a significantly higher risk of developing dry socket due to elevated estrogen levels, which stimulate the fibrinolytic system (specifically causing lysis of the blood clot).
• Treatment Planning
• The primary goal of treatment is palliative pain relief, as the condition is self-limiting and healing will eventually occur by secondary intention (granulation tissue forming from the walls of the socket).
• 1. Examination and Irrigation
• Gently irrigate the socket with warm sterile saline or a chlorhexidine solution.
• This removes necrotic tissue, broken down clot remnants, and accumulated food debris that are contributing to the pain and foul odor.
• Do not induce bleeding or curette the socket walls aggressively, as this may expose more bone and increase pain.
• 2. Placement of Intrasocket Medicament
• Place a resorbable or non-resorbable sedative dressing into the socket.
• Common materials include Alvogyl (containing eugenol, iodoform, and butamben) or plain iodoform gauze soaked in a eugenol-based medication.
• This provides almost immediate pain relief by covering the exposed bone and soothing the nerve endings.
• 3. Pain Management
• Prescribe or recommend potent non-steroidal anti-inflammatory drugs (NSAIDs) such as Ibuprofen or Naproxen to manage the inflammatory pain.
• Systemic analgesics alone are often insufficient without the local sedative dressing.
• 4. Follow-up
• If a non-resorbable dressing (gauze) is used, the patient must return in 24–48 hours for removal or replacement.
• If a resorbable paste (like Alvogyl) is used, it does not need to be removed, but the patient may still need to return if pain persists.
• Important Note on Antibiotics: Systemic antibiotics are generally not indicated for alveolar osteitis unless there are signs of spreading infection (e.g., fever, lymphadenopathy, or facial swelling). Dry socket is a disturbance of healing, not a pyogenic infection.
• Would you like me to elaborate on the specific physiology of how oral contraceptives affect clot stability?

5. In a 24-year-old patient, persistent numbness of the lower lip and chin region for three months following a difficult mandibular right 48 extraction suggests which neurological complication, and how should this condition be managed?

• Based on the clinical presentation, the patient is suffering from Inferior Alveolar Nerve (IAN) Paresthesia (or neuropathy).
• The persistent numbness of the lower lip and chin specifically indicates damage to the mental nerve, a terminal branch of the IAN, which supplies sensation to the skin of the chin and the mucous membrane of the lower lip.
• 1. Diagnosis and Mechanism
• The condition is a traumatic neuropathy of the Inferior Alveolar Nerve resulting from the extraction of the mandibular right third molar (tooth 48).
• Anatomy: The roots of the mandibular third molars often lie in close proximity to, or even wrap around, the mandibular canal where the IAN resides.
• Mechanism: During a "difficult" extraction, the nerve can be damaged via compression (crushing), stretching (traction), or direct severance (laceration) by surgical instruments or bone fragments.
• Chronicity: Numbness persisting for three months is a critical clinical marker. While simple compression (neurapraxia) often resolves within days or weeks, symptoms lasting 3 months suggest more severe damage (axonotmesis or neurotmesis) and a lower likelihood of spontaneous complete recovery.
• 2. Management Protocol

• At the three-month mark, the window for "wait and see" is closing. Management should shift from passive observation to active assessment and potential intervention.
• A. Clinical Assessment (Neurosensory Testing)
• A thorough mapping of the affected area is required to determine the degree of nerve damage. This usually involves:
• Mapping: Delineating the area of numbness on the chin/lip to see if it is shrinking (sign of recovery) or static.
• Mechanoceptive Testing: Two-point discrimination and brush directional stroke tests to assess large fiber function.
• Nociceptive Testing: Pin-prick tests (sharp vs. dull) and thermal testing to assess small fiber function.
• B. Radiographic Evaluation
• Standard panoramic X-rays (OPG) are insufficient for detailed nerve mapping. A Cone Beam CT (CBCT) is essential to:
• Visualize the inferior alveolar canal.
• Identify any bony compressions, retained root tips, or debris impinging on the nerve.
• C. Surgical Referral and Intervention
• This is the most critical step for a patient at the 3-month mark.
• Referral: The patient should be referred immediately to an oral and maxillofacial surgeon specializing in nerve microsurgery.
• The "3-Month Rule": If there is no sign of neurosensory improvement by 3 months, exploratory microsurgery is often indicated.
• Procedure: Surgery may involve:
o Decompression: Removing bone or scar tissue pressing on the nerve.
o Direct Neurorrhaphy: Stitching the nerve ends back together if severed.
o Nerve Grafting: Using a donor nerve (often the sural nerve or great auricular nerve) to bridge a gap if the nerve ends cannot be re-approximated without tension.
• Summary of Prognosis
• Outcomes for nerve repair are time-sensitive. Repairs performed within 3 to 6 months of the injury yield significantly better functional results than those performed later. If left untreated beyond 6–9 months, central cortical changes and distal nerve atrophy make successful surgical recovery highly unlikely.
• Would you like me to detail the specific steps for performing the neurosensory "mapping" test in a clinical setting?

6. In a 26-year-old patient, following lingual retraction during extraction of tooth 38, numbness and loss of taste develop on the left half of the tongue. Which nerve injury does this suggest, and how should it be treated?

• Based on the clinical presentation, here is the assessment of the nerve injury and the recommended management protocol.
• Diagnosis: Injury to the Left Lingual Nerve
• The symptoms described—anesthesia (numbness) combined with ageusia (loss of taste) on the anterior two-thirds of the tongue—are pathognomonic for injury to the Lingual Nerve.
• Anatomical Explanation
• Numbness: The Lingual Nerve provides general sensory innervation (touch, pain, temperature) to the anterior two-thirds of the tongue and the lingual soft tissues.
• Loss of Taste: At the level of the mandibular third molar (tooth 38), the Lingual Nerve carries fibers from the Chorda Tympani (a branch of the Facial Nerve, CN VII). The Chorda Tympani provides special sensory taste fibers to the anterior two-thirds of the tongue.
• Mechanism: During the extraction of lower third molars, a lingual flap retraction can compress or stretch the nerve against the lingual cortical plate, resulting in neuropraxia (bruising) or axonotmesis (damage to nerve fibers).
• Management and Treatment
• Management depends on the severity and duration of the injury. Since traction injuries often result in neuropraxia (temporary conduction block), spontaneous recovery is common.
• 1. Immediate Management (Acute Phase)
• Documentation: Perform and document basic neurosensory testing (e.g., light touch, two-point discrimination, pinprick) to establish a baseline. map the affected area.
• Pharmacotherapy: If the injury is suspected immediately post-op (or within 1 week), a course of systemic corticosteroids is often prescribed to reduce neural edema and

compression.
o *Example:* Dexamethasone or Prednisolone (tapering dose) for 5–7 days.
o Vitamin B Complex: Often prescribed for neurotrophic support, though clinical evidence varies.
• 2. Observation (Subacute Phase)
• Wait-and-See Protocol: Most traction injuries resolve spontaneously within 6 to 8 weeks.
• Monitoring: The patient should be monitored monthly to track the return of sensation. Signs of paresthesia (tingling) often precede the return of normal sensation.
• 3. Surgical Intervention (Chronic/Non-Resolving)
• Referral: If there is no sign of improvement after 3 months, or if the anesthesia is profound and persistent, referral to a microneurosurgeon or oral and maxillofacial surgeon is indicated.
• Microsurgery: Procedures may include external neurolysis (freeing the nerve from scar tissue), direct neurorrhaphy (suturing), or nerve grafting. Outcomes are significantly better if surgery is performed within 3–6 months of the injury.
• *Disclaimer: I am an AI, not a doctor. This information is for educational purposes regarding clinical scenarios and does not constitute medical advice or a treatment plan for a specific patient.*

7. In a 26-year-old patient, a tooth is lost during extraction of tooth 28, and two days later CBCT shows the tooth in the infratemporal fossa. What does this finding indicate, and what is the appropriate treatment approach?

• The displacement of a maxillary third molar (tooth 28) into the infratemporal fossa is a rare but serious complication of extraction, typically resulting from excessive force or improper elevator positioning during the luxation of an impacted tooth.
• What This Finding Indicates
• Displacement into Tissue Spaces: The tooth has been pushed posteriorly and superiorly through the periosteum of the distal maxillary tuberosity.
• Anatomic Location: The infratemporal fossa is a deep space located inferior to the zygomatic arch, posterior to the maxilla, and medial to the ramus of the mandible. It contains critical structures, including:
o The pterygoid venous plexus.
o The maxillary artery (and its branches).
o The mandibular nerve (V3) and its branches (e.g., lingual and inferior alveolar nerves).
o Pterygoid muscles.
• Risk of Infection/Pathology: A displaced tooth acts as a foreign body and can lead to severe deep space infections, fibrosis, or migration further into the skull base if left untreated.
• Appropriate Treatment Approach
• Because the displacement occurred two days ago, immediate retrieval in a standard clinic setting is generally contraindicated due to the risk of pushing the tooth deeper, hemorrhage from the pterygoid plexus, or nerve injury.
• 1. Referral to Oral and Maxillofacial Surgeon (OMFS)
• This case requires management by a specialist, often in a hospital setting where general anesthesia and advanced imaging are available.
• 2. Watchful Waiting vs. Surgical Retrieval
• Immediate Action (First 24 hours): Usually, if the tooth is high in the fossa and not visible, the procedure is aborted, the patient is placed on antibiotics, and retrieval is delayed to allow fibrosis to stabilize the tooth.
• Delayed Retrieval (Current Scenario - 2 days post-op):
o Fibrosis formation: Waiting 2–4 weeks allows a fibrous capsule to form around the tooth, stabilizing it and making retrieval safer and more predictable.
o Antibiotic Prophylaxis: The patient should be maintained on broad-spectrum antibiotics to prevent infection in the infratemporal space.
o Monitoring: The patient should be monitored for signs of infection, trismus (lockjaw), or ocular symptoms.
• 3. Surgical Retrieval
• Imaging: The CBCT you already have is critical for 3D localization. Intraoperative navigation systems may be used in complex cases.
• Approach:
o Intraoral Approach: Most common. An incision is made high in the mucobuccal fold (similar to a Caldwell-Luc approach or extended third molar incision) to

access the posterior maxilla. Dissection is carried out carefully to locate the tooth.
o Extraoral Approach: Rarely required, usually reserved for cases where the tooth has migrated significantly superiorly or laterally (e.g., Gillies temporal approach or hemicoronal approach).
• Anesthesia: General anesthesia is strictly required to ensure patient immobility and airway protection.
• 4. Post-Operative Management
• Strict observation for hematoma formation (due to the pterygoid plexus).
• Management of trismus, which is common after manipulation of the pterygoid muscles.
• Summary of Steps
• Inform the patient honestly about the complication.
• Refer immediately to an Oral and Maxillofacial Surgeon.
• Prescribe antibiotics and analgesics.
• Do not attempt blindly retrieving the tooth in the chair, as this often worsens the displacement.
• Would you like me to find the specific referral guidelines or typical recovery timelines for this type of surgical retrieval?

8. In a 34-year-old patient, a mandibular basal (inferior border) fracture occurs during extraction of a deeply impacted tooth 38 due to excessive elevator force. What should the emergency management approach be?

• This is a serious intraoperative complication that requires immediate cessation of the extraction and a shift to fracture management protocols. Because the fracture involves the inferior border (basal bone) and occurred during the elevation of an impacted third molar (tooth 38), it is likely an angle fracture, which is frequently "unfavorable" due to muscle pull.
• The emergency management approach should follow these steps:
• 1. Immediate Intraoperative Measures
• Stop the Procedure: Immediately cease all attempts to elevate the tooth. Continued force will displace the fracture segments further and may damage the Inferior Alveolar Nerve (IAN).
• Manual Stabilization: If the segments are mobile, manually support the mandible to prevent displacement and patient discomfort.
• Assess Neurological Status: Quickly document the presence or absence of lip and chin sensation (V3 distribution) to establish a baseline for IAN injury.
• Wound Care: If there is active bleeding from the socket or soft tissue, achieve hemostasis with pressure or packing.
• 2. Management of the Tooth in the Fracture Line
• Do Not Chase the Tooth: Unless the tooth is completely loose and can be removed with tweezers without force, do not attempt to remove it at this stage.
• Rationale: The impacted tooth effectively acts as a "splint" or wedge between the segments. Removing it without fixation hardware ready can cause the proximal segment (the ramus/angle) to collapse superiorly due to the pull of the masseter and medial pterygoid muscles. The tooth is typically removed during the definitive surgical repair (Open Reduction and Internal Fixation).
• 3. Radiological Assessment
• You must obtain imaging to visualize the extent of the fracture and the position of the tooth.
• OPG (Orthopantomogram): The standard screening view to see the fracture line relative to the inferior border and the angle.
• CBCT/CT: Often required to assess the buccolingual displacement of the segments and the precise location of the IAN canal relative to the fracture and the tooth roots.
• 4. Stabilization and Referral
• Temporary Immobilization:
o If the patient has a stable occlusion (bite feels normal), advise them to restrict jaw movement.
o If the mandible is mobile or hanging, a Barton bandage (a head bandage supporting the jaw) should be applied to limit movement and reduce pain during transport.
• Referral: This injury requires management by a Maxillofacial Surgeon. The standard of care is usually Open Reduction and Internal Fixation (ORIF) with mini-plates to rigidly fix the fracture and allow for the safe removal of the impacted tooth.
• 5. Medical Management (Prescriptions)
• Since the fracture communicates with the oral cavity through the extraction socket, it is

considered an open (compound) fracture.
• Antibiotics: Prophylactic antibiotics (e.g., Amoxicillin/Clavulanate or Clindamycin) are mandatory to prevent infection of the fracture site.
• Analgesics: Strong pain relief (NSAIDs + Opioids if necessary).
• Corticosteroids: (e.g., Dexamethasone) may be prescribed to manage the anticipated edema.
• Instructions: Soft/liquid diet and absolute avoidance of chewing.
• Summary Table: Action Plan
Ste p Action Key Consideration
1 STOP Do not apply further elevator force.
2 Assess Check occlusion and IAN (lip/chin) sensation.
3 Image OPG or CBCT to confirm fracture type (favorable vs. unfavorable).
4 Medicate Antibiotics (critical for open fracture) and Analgesics.
5 Refer Transfer to Maxillofacial Surgeon for ORIF.
• Would you like me to detail the surgical considerations for the definitive Open Reduction and Internal Fixation (ORIF) for this specific type of fracture?
9. In a 28-year-old patient, one week after extraction of tooth 48, progressively worsening severe pain, trismus, fever, and cervical lymphadenopathy develop. The patient also has dysphagia/odynophagia, diffuse neck swelling, and chest pain–shortness of breath, raising suspicion of spread of infection through deep neck spaces to the mediastinum. Which diagnosis does this presentation most strongly suggest?
• Based on the clinical presentation, the most likely diagnosis is Descending Necrotizing Mediastinitis (DNM).
• While the patient likely began with a deep neck infection (such as Ludwig's Angina) originating from the tooth extraction, the presence of chest pain and shortness of breath indicates the infection has spread beyond the neck and into the chest cavity, creating a life-threatening emergency.
• Key Clinical Indicators
• The progression of symptoms in this scenario follows a classic "red flag" pattern for DNM:
• Odontogenic Origin: The history of a recent lower third molar (Tooth 48) extraction is the most common cause of deep neck infections.
• Deep Neck Space Involvement: Symptoms like trismus (lockjaw), dysphagia (difficulty swallowing), and diffuse neck swelling suggest the infection has breached the primary spaces (submandibular, sublingual) and moved into the deep cervical spaces.
• Mediastinal Involvement (The Critical Sign): The development of chest pain and shortness of breath distinguishes this from a localized neck abscess. These symptoms suggest the infection has traveled inferiorly into the mediastinum (the space in the chest between the lungs).
• Pathophysiology: The "Danger Space"
• The spread of infection likely followed a specific anatomical pathway:
• Origin: Infection starts at the extraction site of the mandibular third molar.
• Primary Spread: It moves into the submandibular or pterygomandibular spaces.
• Deep Spread: It enters the retropharyngeal space or the "Danger Space" (a potential space posterior to the retropharyngeal space).
• Mediastinal Entry: The Danger Space extends from the skull base continuously down to the diaphragm. Once an infection enters this channel, gravity and negative intrathoracic pressure can facilitate the rapid spread of purulent material into the posterior mediastinum.
• Differential Diagnosis & Severity
• Ludwig's Angina: This is a firm, bilateral cellulitis of the submandibular, sublingual, and submental spaces. While this patient likely *has* Ludwig's Angina (indicated by the neck swelling and trismus), the diagnosis of DNM supersedes it because the infection has advanced into the chest.
• Lemierre's Syndrome: This involves septic thrombophlebitis of the internal jugular vein and can cause chest symptoms (due to septic emboli in the lungs). However, the prompt specifically describes direct "spread through deep neck spaces," which favors DNM.
• Immediate Implications:
• Descending Necrotizing Mediastinitis has a high mortality rate. Management typically requires immediate broad-spectrum antibiotics and aggressive surgical drainage of both the cervical and mediastinal collections (often requiring thoracotomy).
• Would you like to examine the specific anatomical boundaries of the "Danger Space" versus the Retropharyngeal Space to understand why one leads directly to the

diaphragm?

10. In a 25-year-old patient, six weeks after mandibular 48 extraction, the patient reports food impaction and bleeding distal to tooth 47. Clinical examination reveals a ≥6 mm periodontal pocket distal to 47 with bleeding on probing; radiographs show localized bone loss distal to 47. What surgical and postoperative approach should be implemented in this case?

• Based on the clinical presentation—a young patient (25 years old) with a deep periodontal pocket ($\ge$6 mm), bleeding on probing, and localized bone loss distal to the second molar (47) following the recent extraction of an impacted third molar (48)—the standard surgical approach is Guided Tissue Regeneration (GTR).

• Simple soft tissue reduction (distal wedge) is generally contraindicated when there is underlying vertical bone loss, as it removes tissue needed for primary closure over a graft. Scaling alone is unlikely to resolve a vertical osseous defect of this magnitude.

• Here is the detailed surgical and postoperative management plan:

• 1. Surgical Approach: Guided Tissue Regeneration (GTR)

• The goal is to regenerate the lost periodontal attachment (bone, periodontal ligament, and cementum) distal to tooth 47 to prevent future tooth loss.

• Flap Design and Elevation:

o A full-thickness mucoperiosteal flap is elevated to expose the defect distal to 47.

o Incisions should be designed to preserve as much keratinized tissue as possible to ensure primary closure over the membrane. Sulcular incisions are made around the distal of 47.

• Debridement:

o Thorough removal of all granulation tissue from the osseous defect is critical.

o The distal root surface of 47 must be carefully scaled and planed to remove plaque, calculus, and any endotoxins.

• Root Conditioning (Optional but common):

o Chemical conditioning of the root surface (e.g., with EDTA or tetracycline) may be used to remove the smear layer and expose collagen fibers to enhance attachment.

• Regenerative Materials:

o Bone Graft: An osteoconductive or osteoinductive bone graft material (e.g., freeze-dried bone allograft or bovine xenograft) is placed into the bony defect to act as a scaffold.

o Barrier Membrane: A resorbable membrane (e.g., collagen) is placed over the graft and the defect. This prevents the faster-growing gingival epithelial cells from migrating into the defect, allowing the slower-growing bone and PDL cells to populate the area.

• Closure:

o The flap is repositioned coronally or to its original position.

o Primary closure is essential to protect the membrane and graft. Tension-free suturing (e.g., with mattress sutures) is required.

• 2. Postoperative Approach

• Proper postoperative care is vital to the success of GTR, as the initial clot and membrane stability determine the regenerative outcome.

• Pharmaceutical Management:

o Antibiotics: Systemic antibiotics (e.g., Amoxicillin 500mg tid for 7 days) are frequently prescribed to prevent infection of the membrane/graft.

o Analgesics: NSAIDs (e.g., Ibuprofen) for pain and inflammation control.

o Antiseptic Rinse: Chlorhexidine gluconate (0.12%) mouth rinse twice daily is prescribed.

• Hygiene Instructions:

o No mechanical brushing or flossing at the surgical site for at least 2–3 weeks to avoid disturbing the sutures and membrane. The patient should rely on the Chlorhexidine rinse for plaque control in that specific area.

o Soft diet to prevent mechanical trauma to the distal site.

• Follow-up Schedule:

o Suture Removal: Typically at 10–14 days.

o Weekly/Bi-weekly checks: To ensure plaque control is maintained (supragingival professional cleaning may be needed).

o NO Probing: Do not probe the site for at least 6 months to allow the periodontal attachment to mature. Early probing can disrupt the healing attachment.

• Summary of Clinical Rationale
• The extraction of mesioangular or horizontally impacted mandibular third molars often results in an osseous defect on the distal aspect of the second molar. In a 25-year-old patient, the regenerative potential is high. Leaving a 6mm pocket untreated will likely lead to chronic periodontitis, further bone loss, and eventual loss of the second molar.
• Would you like me to detail the specific risk factors (like 3rd molar angulation) that likely caused this defect initially?

11. In a 29-year-old patient, recurrent bleeding from the extraction site begins approximately one week after extraction of mandibular left 38. What mechanism could explain this delayed bleeding, and how should it be managed?

• This clinical presentation is characteristic of secondary hemorrhage. Unlike immediate or reactionary bleeding, which occurs within the first 24 hours, secondary hemorrhage typically presents 7–10 days post-operatively.
• Mechanism: Infection and Clot Lysis
• The delayed onset (one week later) is the key diagnostic clue. The primary mechanism is almost invariably infection at the surgical site.
• Fibrinolysis: Bacteria present in the socket produce enzymes (or induce an inflammatory response) that cause the lysis (breakdown) of the fibrin clot that had previously formed.
• Erosion of Vessels: The chronic inflammation and infection can erode the granulation tissue or small blood vessels within the socket wall, leading to new bleeding.
• Anatomy: In the case of the mandibular left third molar (38), the surgical site is often close to the Inferior Alveolar neurovascular bundle. While rare, erosion near this bundle due to deep infection can cause significant bleeding, though capillary oozing from granulation tissue is more common.
• Management Protocol
• Management focuses on controlling the acute bleeding and treating the underlying infection.
• 1. Immediate Hemostasis
• Visualization: Administer local anesthesia (with vasoconstrictor) to ensure the patient is comfortable and to help visualize the source of bleeding.
• Debridement: Gently irrigate the socket with saline to remove the old, infected, or lytic clot and food debris. Suction carefully to identify the specific bleeding point.
• Curettage: Gently curette the socket walls. This serves two purposes: it removes the infected granulation tissue and stimulates fresh bleeding to form a new, healthy clot.
• 2. Local Measures
• Packing: If bleeding persists after curettage, pack the socket with a resorbable hemostatic agent (e.g., Oxidized Regenerated Cellulose like Surgicel, or a gelatin sponge like Gelfoam).
• Suturing: Place a suture (usually 3-0 or 4-0 silk or resorbable) to hold the hemostatic agent in place and approximate the gingival margins.
• Pressure: Have the patient bite firmly on a gauze pack for at least 30–60 minutes.
• 3. Systemic Treatment
• Antibiotics: Because the root cause is infection, a course of systemic antibiotics is required. Amoxicillin (500mg tid) or Metronidazole (400mg tid) are commonly prescribed, depending on the patient's allergy status and local antibiotic stewardship guidelines.
• Tranexamic Acid (Optional): In cases of persistent oozing, the patient can be instructed to use a 5% tranexamic acid mouthwash or soak the gauze in it before biting.
• Summary Table
Clinical Stage Timeframe Primary Cause Management Focus
Primary During surgery Vessel severance Ligation, cautery, bone wax
Reactionary < 24 hours Dislodged clot / BP rise Pressure, re-suturing
Secondary 7–10 days Infection Antibiotics, debridement, packing
• Would you like me to outline the specific antibiotic dosage regimens typically used for treating secondary socket infections?

12. In a 22-year-old patient, soft tissue trauma–related tissue hemorrhage occurs during local anesthetic injection prior to mandibular 38 extraction. How would you proceed with the procedure in this situation?

• This scenario describes a hematoma formation caused by needle trauma to a blood vessel (likely the Inferior Alveolar Artery, Vein, or the Pterygoid Venous Plexus) during an Inferior Alveolar Nerve Block (IANB).
• This is a known complication, but it requires immediate management to prevent trismus, infection, or airway compromise. Here is the clinical protocol for how to proceed.
• 1. Immediate Management (The First 5 Minutes)

• Before making any decision about the extraction, you must control the hemorrhage.
• Stop and Withdraw: Immediately terminate the injection and withdraw the needle. Do not deposit more solution into the area of hemorrhage.
• Apply Direct Pressure: This is the most critical step.
o Place gauze or a gloved finger firmly against the medial aspect of the mandibular ramus (the injection site).
o Maintain firm, continuous pressure for at least 2 to 5 minutes. Do not release pressure to "check" the bleeding repeatedly, as this disrupts clot formation.
• Cold Application: Apply an ice pack or cold compress extra-orally to the affected side of the jaw. This induces vasoconstriction and limits the size of the hematoma.
• 2. Clinical Assessment
• Once pressure has been maintained and the initial expansion has stopped, assess the site to determine the feasibility of the procedure.
• Size and Expansion: Is the swelling localized and stable, or is it large and continuing to expand into the lateral pharyngeal space?
• Trismus: Ask the patient to open their mouth. Is their range of motion significantly restricted by the swelling?
• Anesthetic Effect: Did any anesthetic reach the nerve before the hematoma formed? Is the patient numb?
• Patient Status: Is the patient anxious, distressed, or in pain?
• 3. Decision Matrix: Proceed or Abort?
• Whether you proceed with the extraction of tooth 38 depends on the severity of the hematoma.
Scenario Clinical Presentation Action Plan
• Hematoma is large or rapidly expanding.
• Do not proceed.
• Significant trismus (patient cannot open wide enough for access).
• The blood in the tissues will neutralize the pH of local anesthetics, making re-anesthesia difficult.
Abort Procedur e
• Signs of airway distress (rare but critical).
• Trauma from surgery increases infection risk in the hematoma.
• Patient is highly distressed/uncooperative.
• Reschedule for 10–14 days later.
• Hematoma is small/minor and stable.
• Re-anesthetize carefully.
• No trismus; access is clear.
• Avoid the hematoma: Use a different technique (e.g., Gow-Gates or Vazirani-Akinosi) to bypass the pooled blood and inflamed tissue.
Proceed with Caution
• Patient is calm and compliant.
• Administer antibiotic prophylaxis if the hematoma is significant (to prevent secondary infection of the clot).
• Note: Proceed only if the tooth is symptomatic/urgent or if the patient insists after informed consent.
• 4. Patient Management & Post-Op Instructions
• Regardless of whether you extract the tooth or reschedule, the patient requires specific care for the hematoma:
• Documentation: Record the size, location, and management of the hematoma in the clinical notes.
• R.I.C.E Protocol (Modified):
o Ice: Apply ice extra-orally for the first 24 hours (20 mins on, 20 mins off) to reduce swelling.
o Heat: After 24 hours, switch to warm moist heat (e.g., warm towel) to vasodilate and help the body reabsorb the blood. Do not use heat on the first day.
• Analgesics: Avoid NSAIDs (like Aspirin or Ibuprofen) immediately if possible, as they can inhibit platelet aggregation; Acetaminophen (Paracetamol) is preferred for the first day.

• Warning Signs: Instruct the patient to go to the ER if they experience difficulty breathing, swallowing, or speaking (dysphagia/dyspnea).
• Expectation Management: Warn the patient that they will likely develop bruising (ecchymosis) on the face and neck over the next 7–14 days as the blood tracks downward due to gravity.
• Would you like me to detail the technique for the Gow-Gates block as an alternative to the IANB for this scenario?

13. A 25-year-old patient presents two days after mandibular 48 extraction with limited mouth opening and difficulty eating. How should the patient be assessed and managed?

• Disclaimer: *The following information is for educational purposes and does not constitute medical advice. Assessment and management should be performed by a qualified dental or medical professional.*
• A presentation of trismus (limited mouth opening) and dysphagia (difficulty eating) 48 hours post-extraction of a mandibular third molar (Tooth 48) is a common clinical scenario. This timeframe coincides with the peak of the post-operative inflammatory phase, but pathological complications must be ruled out.
• I. Assessment Phase
• The primary goal is to distinguish between physiological post-operative inflammation and developing infection (specifically involving the fascial spaces).
• 1. History & Symptom Review
• Systemic Signs: Ask about fever, chills, or malaise.
• Pain History: Is the pain throbbing (suggestive of infection) or a dull ache? Is it controlled by analgesics?
• Swallowing/Breathing:
o *Dysphagia (difficulty swallowing):* Indicates pharyngeal involvement.
o *Dyspnea (difficulty breathing):* RED FLAG indicating potential airway compromise (e.g., Ludwig's Angina or lateral pharyngeal space involvement).
• Taste: Any foul taste or discharge (purulence) in the mouth?
• 2. Clinical Examination
• Extra-Oral:
• Swelling: Palpate the angle of the mandible.
o *Soft/Oedematous:* Likely normal inflammation.
o *Firm/Indurated or Fluctuant:* Suggests abscess or deep space infection (Submasseteric or Pterygomandibular space).
• Lymphadenopathy: Check for tender, palpable cervical lymph nodes.
• Trismus Measurement: Measure the inter-incisal opening. Normal post-op inflammation may restrict opening to 20–30mm; severe restriction (<10–15mm) often indicates infection of the masticator space.
• Intra-Oral:
• Site Inspection: Irrigate debris. Check for a disintegrating clot (Alveolar Osteitis/Dry Socket), though Dry Socket typically presents with *pain* rather than severe trismus.
• Pus Discharge: Palpate the vestibule and distal to the extraction site for exudate.
• Pharyngeal Assessment: Look for medial displacement of the lateral pharyngeal wall or uvular deviation. This is a critical sign of deep neck space infection requiring immediate escalation.
• II. Differential Diagnosis
• Post-operative Inflammation (Most Likely): Trauma to the medial pterygoid or masseter muscles during surgery causes reactive spasms. Peak swelling occurs at 48–72 hours.
• Submasseteric/Pterygomandibular Abscess: Infection tracking into the spaces between the muscles of mastication.
• Hematoma: Collection of blood in the muscle planes, leading to fibrosis or secondary infection.
• Needle Track Trauma: Inflammation from the Inferior Alveolar Nerve (IAN) block injection site.
• III. Management Strategies
• Scenario A: Inflammation (No systemic signs, no fluctuation, airway patent)
• If the assessment suggests normal post-operative trauma:
• Reassurance: Explain that day 2–3 is typically the peak for swelling and discomfort.
• NSAIDs: Prescribe or optimize anti-inflammatory medication (e.g., Ibuprofen 400mg–600mg) to reduce muscle inflammation.
• Thermotherapy: Switch from cold packs (used in first 24h) to moist heat packs externally to relax the muscles.

• Soft Diet: Strictly non-chew diet to rest the jaw.
• Physiotherapy: Gentle active range-of-motion exercises. The patient should attempt to open their mouth slightly past the point of pain for short intervals.
• Review: Re-assess in 48 hours. If worsening, treat as infection.
• Scenario B: Infection (Fever, lymphadenopathy, severe trismus, pus)
• Antibiotics: Prescribe broad-spectrum antibiotics covering oral flora (e.g., Amoxicillin or Metronidazole; Clindamycin if penicillin-allergic).
• Drainage: If an intra-oral fluctuant swelling is palpable, incision and drainage (I&D) are necessary.
• Debridement: Irrigate the socket with saline or chlorhexidine to remove food debris.
• Scenario C: Emergency Referral
• Immediate referral to an Oral & Maxillofacial Surgeon is required if:
• The patient has difficulty breathing or swallowing liquids.
• There is inability to open the mouth sufficiently to examine the throat.
• Temperature >38.5°C with systemic toxicity.
• Swelling extends significantly into the neck or crosses the midline.
• Next Step
• Would you like me to detail the specific antibiotic regimens suitable for odontogenic infections, or should we discuss the red flags for airway compromise in more depth?

14. In a 55-year-old patient with a history of four doses of intravenous bisphosphonate (zoledronic acid) use, an unhealed area of exposed bone and pain develop weeks after extraction of mandibular 38. Which complication is most likely, and how should management be planned?

• Based on the clinical presentation and history, the diagnosis and management plan are as follows:
• Most Likely Complication: Medication-Related Osteonecrosis of the Jaw (MRONJ)
• The patient presents with the classic triad for MRONJ (formerly known as Bisphosphonate-Related Osteonecrosis of the Jaw, or BRONJ):
• Current or previous treatment with antiresorptive or antiangiogenic agents: The patient has a history of IV Zoledronic Acid (a highly potent bisphosphonate).
• Exposed bone in the maxillofacial region: Specifically in the mandible, which is more prone to necrosis than the maxilla due to lower vascularity.
• No history of radiation therapy to the jaws: (Implied, as this distinguishes it from Osteoradionecrosis).
• Pathophysiology:
• Zoledronic acid inhibits osteoclast function and bone resorption, which impairs normal bone remodeling. Following an invasive dental procedure like an extraction (a major risk factor), the bone is unable to heal, leading to necrosis. The addition of pain suggests secondary infection of the exposed bone.
• Management Planning
• Management is guided by the staging of the disease. Based on the American Association of Oral and Maxillofacial Surgeons (AAOMS) guidelines:
• Staging: The patient likely has Stage 2 MRONJ.
o *Stage 0:* No clinical evidence of necrotic bone, but non-specific symptoms.
o *Stage 1:* Exposed necrotic bone, but asymptomatic and no infection.
o *Stage 2:* Exposed necrotic bone associated with infection, pain, and erythema (matches this patient).
o *Stage 3:* Exposed bone with extensive destruction, pathologic fracture, or extra-oral fistula.
• Treatment Protocol (Stage 2 Focus)
• Since the patient is symptomatic (pain), the treatment goal is to control infection and pain. The approach is primarily conservative medical management.
• Antimicrobial Mouth Rinses:
o Use of 0.12% Chlorhexidine gluconate mouthwash to reduce local bacterial load and prevent plaque accumulation over the exposed bone.
• Systemic Antibiotics:
o Because pain and inflammation indicate infection, oral systemic antibiotics are indicated.
o First-line: Penicillin V-K or Amoxicillin (often combined with Metronidazole).
o If Penicillin-allergic: Clindamycin, Doxycycline, or Azithromycin.
o *Note:* Culture and sensitivity testing may be needed if the infection does not resolve.

• Pain Control:
o Prescription of appropriate analgesics (NSAIDs or stronger if necessary) to manage the patient's discomfort.
• Limited Surgical Debridement:
o Superficial debridement to relieve soft tissue irritation (e.g., removing sharp bony spicules).
o Avoid aggressive surgery (like extensive resection) initially, as it may expose more bone and worsen the condition, unless the conservative approach fails or the bone is clearly a loose sequestrum.
• Oncology Consultation:
o Coordinate with the prescribing physician/oncologist. While discontinuing the bisphosphonate (a "drug holiday") is a theoretical option, it is controversial and often not recommended for oncology patients receiving IV bisphosphonates due to the high risk of skeletal-related events, unless the necrosis is unmanageable.
• Summary Table
Component Action
Diagnosis MRONJ (Stage 2)
Primary Goal Control infection and pain
Medication Penicillin/Amoxicillin + Chlorhexidine 0.12%
Surgery Limited superficial debridement only
• Would you like me to explain the specific risk differences between oral bisphosphonates and IV bisphosphonates regarding extraction safety?

15. In a 55-year-old patient with a history of radiotherapy, an unhealed area of exposed bone and pain develop weeks after extraction of tooth 38. Which complication is this associated with, and how should it be managed?

• Based on the clinical presentation—specifically the history of radiotherapy, the location (mandible/tooth 38), and the presence of exposed bone with pain persisting weeks after extraction—the associated complication is Osteoradionecrosis (ORN).
• Below is a breakdown of the condition and its management.
• Diagnosis: Osteoradionecrosis (ORN)
• ORN is a severe complication of radiation therapy characterized by exposed irradiated bone that fails to heal over a period of usually 8 weeks or more, without evidence of persistent or recurrent tumor.
• Pathophysiology: It is caused by radiation-induced hypocellularity, hypovascularity, and hypoxia (Marx’s "3 H" principle). The tissue becomes unable to repair itself after trauma (like a tooth extraction).
• Risk Factors:
o Total radiation dose > 60 Gy.
o Mandibular extractions (the mandible has poorer blood supply than the maxilla).
o Posterior sites (like tooth 38).
o Poor oral hygiene or continued tobacco/alcohol use.
• Management Strategies
• Management depends on the clinical stage (e.g., Marx Staging I, II, or III), but generally follows a step-wise approach.
• 1. Conservative Management (Early Stage)
• For patients with exposed bone but no severe infection, pathological fracture, or fistula:
• Local Irrigation: Daily irrigation with saline or 0.12% Chlorhexidine to keep the area clean.
• Antibiotics: Systemic antibiotics (e.g., Penicillin V, Clindamycin, or Metronidazole) are prescribed if there is acute infection, swelling, or drainage.
• Debridement: Gentle removal of loose bony sequestra (dead bone fragments) that are irritating the soft tissue.
• Avoidance: Avoiding irritants like smoking, alcohol, and traumatic prostheses.
• 2. Pharmacological Therapy (PENTOCLO Protocol)
• Increasingly used as a first-line or adjunctive therapy to improve blood flow and reduce fibrosis:
• Pentoxifylline: A vasodilator that improves blood flow.
• Tocopherol (Vitamin E): An antioxidant that reduces scarring and fibrosis.
• (Clodronate): Bisphosphonates may occasionally be used to reduce bone turnover (controversial and case-dependent).
• 3. Hyperbaric Oxygen Therapy (HBO)
• Traditionally considered the "gold standard" for prevention and treatment (Marx Protocol),

though modern efficacy is sometimes debated:
• Involves breathing 100% oxygen at high pressure to stimulate angiogenesis (new blood vessel formation).
• Often used in conjunction with surgery (e.g., 30 dives before surgery, 10 dives after).
• 4. Surgical Management (Advanced Stage)
• If conservative measures fail, or if there is a pathological fracture or orocutaneous fistula:
• Sequestrectomy: Removal of the necrotic bone segment.
• Resection and Reconstruction: Removal of the affected jaw section followed by reconstruction with a vascularized free flap (e.g., fibula free flap) to bring healthy, vascularized tissue to the area.
• Summary Table
Feature Details
Complication Osteoradionecrosis (ORN) of the jaw.
Primary Cause
Radiation-induced tissue damage (Hypoxia, Hypovascularity, Hypocellularity).
Key Symptoms Exposed bone, pain, fetid breath, potential infection/fistula.
First-Line Care Chlorhexidine rinses, antibiotics (if infected), gentle debridement.
Adjuncts Pentoxifylline/Tocopherol, Hyperbaric Oxygen (HBO).
• Would you like me to detail the specific pre-extraction protocols (such as the "Marx Protocol") used to prevent this condition in future cases?

16. In a 62-year-old patient taking warfarin, a mandibular right 48 extraction is performed without checking INR, followed by uncontrolled bleeding. What mechanism explains this bleeding, and what treatment should be applied?

• This clinical scenario presents a classic case of coagulopathy induced by a Vitamin K antagonist (VKA) complicated by a surgical procedure without prior monitoring.
• Here is the breakdown of the physiological mechanism causing the bleeding and the appropriate treatment protocol.
• 1. Mechanism of Bleeding
• The uncontrolled bleeding is caused by the inhibition of the Vitamin K cycle, leading to the depletion of functional coagulation factors.
• Target Enzyme: Warfarin inhibits the enzyme Vitamin K epoxide reductase (VKOR).
• Physiological Impact: Normally, VKOR recycles oxidized Vitamin K epoxide back into its active reduced form (Vitamin K hydroquinone). This active form is an essential cofactor for the enzyme *gamma-glutamyl carboxylase*.
• The Defect: Without active Vitamin K, the liver cannot perform gamma-carboxylation of glutamic acid residues on the "Vitamin K-dependent" clotting factors: II (Prothrombin), VII, IX, and X.
• Result: These "decarboxylated" factors are biologically inactive because they cannot bind Calcium (Ca^{2+}) or phospholipid membranes. This halts the coagulation cascade (specifically the extrinsic and common pathways), resulting in a significantly prolonged Prothrombin Time (PT) and elevated INR, preventing the formation of a stable fibrin clot at the extraction site.
• 2. Management and Treatment
• Management involves a tiered approach: assessing the severity (INR), controlling the local site, and reversing the systemic anticoagulation if necessary.
• A. Immediate Assessment
• Check INR: An urgent INR test is required to determine the degree of anticoagulation.
• B. Local Hemostatic Measures (First-Line)
• For most dental extractions, even with elevated INR, local measures are often sufficient to stop bleeding.
• Mechanical Pressure: Apply firm, continuous pressure with gauze for 20–30 minutes.
• Antifibrinolytics: Apply gauze soaked in Tranexamic Acid (5%) or use it as a mouthwash.
• Packing & Suturing: Place a resorbable hemostatic agent (e.g., oxidized cellulose, gelatin sponge, or collagen plug) into the socket and secure it with sutures to mechanically close the wound.
• C. Systemic Reversal (If bleeding is severe or INR is supratherapeutic)
• If local measures fail or the patient is hemodynamically unstable, systemic reversal of warfarin is required.
Agent Indication Mechanism Onset of Action
Stimulates production of functional factors.
Vitamin K (Phytomenadione)
INR > 5.0 or significant bleeding.

Slow (6–24 hours).
Prothrombin Complex Concentrate (PCC)
Life-threatening or uncontrolled major bleeding.
Directly replaces Factors II, VII, IX, and X.
Rapid (Minutes).
Preferred over FFP.
Replaces factors but requires large volume.
Slower/Less
effective than PCC.
Fresh Frozen Plasma (FFP) If PCC is unavailable.
• Summary Recommendation
• Mechanism: Inhibition of Vitamin K epoxide reductase leading to non-functional Factors II, VII, IX, and X.
• Treatment: Immediate local hemostasis (Surgicel/Gelfoam + Sutures + Tranexamic Acid). If bleeding persists or INR is critically high, administer Vitamin K (oral/IV) and/or PCC for rapid reversal.
• Would you like me to detail the specific dosage protocols for Tranexamic Acid in a dental setting?

17. In a 30-year-old patient, after mandibular 48 extraction, painful oral erosions and cutaneous target lesions develop following use of ibuprofen and amoxicillin. Which drug reaction does this suggest, and how should it be treated?

• Based on the clinical presentation of cutaneous target lesions and painful oral erosions following the administration of ibuprofen (an NSAID) and amoxicillin (a penicillin), the clinical picture is most suggestive of Erythema Multiforme (EM), specifically Erythema Multiforme Major due to the mucosal involvement.
• Here is a breakdown of the diagnosis and management:
• 1. Diagnosis: Erythema Multiforme (EM) Major
• Key Indicator: The hallmark of EM is the target (iris) lesion, which consists of three concentric zones: a central dusky/dark area (or blister), a pale edematous ring, and a peripheral erythematous halo.
• Mucosal Involvement: The presence of painful oral erosions distinguishes EM Major from EM Minor (which affects only the skin).
• Etiology: While Herpes Simplex Virus (HSV) is the most common cause of EM overall, drug reactions are a well-documented etiology, particularly for EM Major. Both NSAIDs (ibuprofen) and penicillins (amoxicillin) are known triggers.
• 2. Differential Diagnosis
• While the "target lesions" strongly point to EM, clinicians must always monitor for severe cutaneous adverse reactions (SCARs), such as:
• Stevens-Johnson Syndrome (SJS): Often involves more widespread mucosal damage and skin detachment. Lesions in SJS are typically "atypical targets" (flat, only two zones).
• Toxic Epidermal Necrolysis (TEN): A more severe variant of SJS with greater skin detachment (>30% body surface area).
• 3. Management and Treatment
• The immediate and primary step in treatment is identifying and removing the antigenic stimulus.
• Discontinue Offending Drugs: Immediately stop both ibuprofen and amoxicillin.
• Supportive Care (Standard of Care):
o Pain Control: Systemic analgesics (avoiding NSAIDs due to the reaction) and topical anesthetics (e.g., viscous lidocaine) for oral lesions to facilitate eating and drinking.
o Hydration: Oral lesions often limit fluid intake, so IV fluids may be necessary to prevent dehydration.
o Nutritional Support: Soft or liquid diet.
o Wound Care: Local care for skin lesions to prevent superinfection.
• Pharmacologic Therapy (Controversial):
o Systemic Corticosteroids: Their use is debated. Some clinicians prescribe them early in the course of severe drug-induced EM to halt progression, while others avoid them due to concerns about infection or delayed wound healing.
o Antihistamines: May be used for symptomatic relief of pruritus (itching).
• *Disclaimer: I am an AI, not a doctor. This information is for educational purposes and should not replace professional medical evaluation or treatment.*
• Would you like me to generate a comparison table distinguishing Erythema

Multiforme from Stevens-Johnson Syndrome?

18. In a 27-year-old patient, during surgical extraction of an impacted tooth 28, a portion of the maxillary tuberosity fractures and separates along with the tooth. What surgical and postoperative management should be performed?

• This scenario describes a fracture of the maxillary tuberosity during the extraction of the upper left third molar (FDI tooth #28). Note that in the Universal Numbering System, #28 is a lower premolar; however, the mention of the "maxillary tuberosity" confirms the location is the upper posterior maxilla.
• This is a significant complication that requires immediate decision-making based on the size of the bony fragment and its attachment to the periosteum (soft tissue).
• 1. Immediate Intraoperative Assessment
• Before proceeding, the surgeon must stop and assess the extent of the fracture.
• Is the bone fragment attached to the periosteum? (Crucial for blood supply and healing).
• How large is the fragment? (Small rim vs. massive tuberosity involving the hamular notch or sinus floor).
• Is the tooth infected or symptomatic? (Determines if extraction can be delayed).
• 2. Surgical Management Options
• Management depends on the mobility and size of the fragment.
• Scenario A: Small Fragment
• If the fractured bone is small and the maxillary sinus is not compromised:
• Remove: Dissect the fragment from the soft tissue and remove it along with the tooth.
• Smooth: File down any sharp bony edges (alveoloplasty).
• Check Sinus: verify if an Oroantral Communication (OAC) exists.
• Closure: Achieve primary closure of the soft tissue to prevent infection or OAC formation.
• Scenario B: Large Fragment (Still Attached to Soft Tissue)
• This is the most critical scenario. Losing a large portion of the tuberosity results in a significant bony defect, making future denture retention difficult.
• Option 1: Rigid Fixation (Delayed Extraction) — The Preferred Conservative Approach
• If the tooth is not acutely infected and the patient can tolerate it:
• Stop the extraction.
• Reposition: Push the tooth and the fractured bone segment back into their original position.
• Stabilize: Splint the mobile segment to the adjacent stable teeth using composite and wire or an arch bar.
• Wait: Allow the bone to heal for 6–8 weeks.
• Re-entry: After healing, return for a surgical extraction. The tooth should be sectioned (cut into pieces) with a surgical bur to remove it with minimal force, preserving the now-healed tuberosity.
• Option 2: Surgical Dissection (Immediate Extraction)
• If the tooth *must* be removed (e.g., acute infection, pathology) or the patient cannot return:
• Dissect: Carefully separate the tooth from the fractured bone fragment using a high-speed handpiece and elevators.
• Preserve Periosteum: It is vital not to reflect the periosteum from the bone fragment. The soft tissue provides the blood supply required for the bone to reattach.
• Stabilize: Once the tooth is out, stabilize the bone fragment with sutures. In severe cases, rigid fixation (screws/wires) may be needed.
• Scenario C: Large Fragment (Completely Detached/Avulsed)
• If the bone has completely separated from the soft tissue (no blood supply):
• Remove: The bone fragment must be removed to prevent necrosis and infection.
• Closure: This often creates a large defect or an Oroantral Communication (OAC).
o Perform a tension-free closure.
o This may require a buccal advancement flap or a buccal fat pad graft to cover the defect and close the sinus.
• 3. Postoperative Management
• Because the maxillary tuberosity is effectively the floor of the maxillary sinus, sinus precautions are mandatory to prevent Oroantral Fistula formation.
• Sinus Precautions (10–14 days):
o Do not blow the nose. Wipe gently if needed.
o Sneeze with the mouth open to reduce sinus pressure.
o Avoid drinking through a straw or smoking (negative pressure).
• Medication:
o Antibiotics: Broad-spectrum (e.g., Amoxicillin or Augmentin; Clindamycin if allergic) to prevent sinus infection.

o Decongestants: Systemic (e.g., Pseudoephedrine) or nasal sprays (e.g., Oxymetazoline) to keep the sinus ostium patent.

• Diet: Soft, cool diet to prevent trauma to the surgical site.

• Follow-up: Monitor for signs of sinus infection (foul taste, discharge) or breakdown of the wound closure.

• *Disclaimer: I am an AI, not a doctor or surgeon. This information is for educational purposes only and should not replace professional medical training or clinical judgment.*

• Would you like me to detail the specific suture techniques used for closing an Oroantral Communication?

19. In a 29-year-old patient, several weeks after extraction of tooth 18, unilateral nasal obstruction, foul-smelling yellow-green discharge, and facial pain develop. Which complication does this suggest, and how should it be managed?

• Based on the clinical presentation—specifically the history of a recent upper molar extraction combined with unilateral foul-smelling discharge—this suggests Chronic Odontogenic Maxillary Sinusitis, likely secondary to an Oroantral Fistula (OAF).

• The "foul-smelling" quality is distinctively characteristic of anaerobic bacteria from the oral cavity infecting the sinus.

• 1. Pathophysiology and Diagnosis

• The Complication: Oroantral Fistula (OAF)

• Tooth 18 (using the FDI system) refers to the Upper Right Third Molar (wisdom tooth). The roots of maxillary molars are often separated from the maxillary sinus floor by only a thin layer of bone or mucous membrane.

• During extraction, this barrier can be disrupted. If the resulting communication (hole) between the oral cavity and the sinus does not heal immediately, it epithelializes into a fistula. This acts as a pathway for food and oral bacteria to enter the sterile sinus, causing chronic infection.

• Diagnostic Steps:

• Clinical Examination: An intraoral exam may reveal a small opening or granulation tissue at the extraction site. A Valsalva maneuver (nose blowing test) might show air bubbling from the socket, though this is often avoided to prevent further damage.

• Imaging: A CBCT (Cone Beam CT) is the gold standard. It allows the surgeon to measure the size of the bony defect and assess the extent of the sinus opacification (fluid/mucosal thickening).

• Endoscopy: Nasal endoscopy can confirm purulent drainage from the middle meatus.

• 2. Management Strategy

• Management requires addressing both the sinus infection and the physical defect. Because this has persisted for "several weeks," it is unlikely to heal spontaneously.

• Phase 1: Medical Management (Pre-operative)

• Before surgery, the active infection must be controlled to improve tissue quality for repair.

• Antibiotics: Broad-spectrum antibiotics covering oral anaerobes and aerobes are required (e.g., Amoxicillin-Clavulanate or Clindamycin combined with Metronidazole).

• Nasal Decongestants: To encourage sinus drainage.

• Saline Irrigation: To help clear purulence.

• Phase 2: Surgical Intervention

• A combined approach is often the most successful standard of care for chronic cases.

• A. Sinus Surgery (FESS)

• If the sinusitis is severe or irreversible despite antibiotics, Functional Endoscopic Sinus Surgery (FESS) is performed first. This enlarges the natural ostium of the sinus to allow drainage into the nose.

• B. Fistula Closure

• The oral defect must be surgically sealed. Common flap techniques include:

• Buccal Advancement Flap (Rehrmann Flap): The gingiva from the cheek is mobilized and stretched over the extraction site.

• Buccal Fat Pad Flap: Ideally used for larger defects or recurrent cases; the buccal fat pad is accessed and gently teased out to cover the hole, where it epithelializes rapidly.

• Summary Table

Feature	Details
Primary Diagnosis	Chronic Odontogenic Maxillary Sinusitis (secondary to OAF)
Key Indicator	Unilateral foul-smelling rhinorrhea (indicates anaerobic oral bacteria)
Imaging of Choice	Cone Beam CT (CBCT)
Primary Treatment	Antibiotics followed by Surgical Closure (FESS + Flap Repair)

• Would you like me to detail the specific post-operative care instructions typically

required after an OAF repair surgery?

20. In a 24-year-old patient, during difficult extraction of a deeply impacted 48, the tooth slips from the elevator and is lost intraorally, followed by coughing and a foreign-body sensation in the throat. What condition does this most strongly suggest, and how should it be managed?

• This scenario most strongly suggests Foreign Body Aspiration, specifically that the tooth has been inhaled into the tracheobronchial tree rather than swallowed.
• The immediate coughing and foreign-body sensation are protective reflexes indicating the object has passed the vocal cords and entered the airway.
• Most Likely Location
• Due to anatomical differences, the aspirated tooth is statistically most likely to lodge in the right main bronchus.
• As shown in the diagram, the right main bronchus is wider, shorter, and more vertical than the left, making it the path of least resistance for falling objects.
• Management Protocol
• 1. Immediate Chairside Measures
• Stop the procedure immediately and remove any remaining debris or packs.
• Position the patient: If the patient is conscious and coughing effectively, encourage them to cough forcefully. Do not slap the back if they are upright and coughing, as this might dislodge the object deeper.
• Monitor Vitals: Assess airway patency and oxygen saturation. If the patient has total airway obstruction (cannot speak or cough, cyanosis), initiate Basic Life Support (Heimlich maneuver) immediately.
• 2. Diagnostic Imaging
• If the tooth is not expelled immediately by coughing, imaging is mandatory to locate it.
• Chest X-ray (PA and Lateral views): This is the gold standard for locating a radiopaque object like a tooth. It helps differentiate between aspiration (airway) and ingestion (esophagus/stomach).
• Abdominal X-ray: If the chest X-ray is negative, an abdominal film may be taken to rule out ingestion (swallowing), though the coughing symptoms make this less likely.
• 3. Definitive Treatment
• Bronchoscopy: If the tooth is identified in the airway on the X-ray, the patient requires an urgent referral to a pulmonologist or thoracic surgeon for a bronchoscopy (usually rigid bronchoscopy) to retrieve the tooth.
• Medical Emergency: This is considered an urgent situation because a retained tooth can lead to lung collapse (atelectasis), pneumonia, or a lung abscess.
• Summary of Difference: Aspiration vs. Ingestion
• Aspiration (Lungs): Violent coughing, stridor, dyspnea. Requires Bronchoscopy.
• Ingestion (Stomach): Often asymptomatic or mild dysphagia. Usually passes spontaneously; monitored via serial stool checks or abdominal X-rays.
• Would you like me to explain the specific anatomical boundaries of the deep spaces (like the submandibular space) where a wisdom tooth might be displaced if it is *not* aspirated?

21. In a 27-year-old patient, during an inferior alveolar nerve block for mandibular right 48, the needle fractures and is lost within the pterygomandibular space. By what mechanism can this occur, and how should it be treated?

• This is a known complication of the inferior alveolar nerve block (IANB), typically occurring when a needle is inserted too deeply or manipulated incorrectly.
• Below is the mechanism of how this fracture likely occurred and the protocol for management.
• 1. Mechanism of Fracture
• Needle breakage in this context is rarely due to a manufacturing defect. It is almost always caused by a combination of the following factors:
• Sudden Patient Movement (Primary Cause): The patient likely moved unexpectedly (jerked their head) causing a reflex muscle contraction or physical stress on the needle shaft.
• Insertion to the Hub: The needle was likely inserted all the way to the hub (the plastic adaptor). The hub is the weakest point of the needle. If the needle breaks here and the hub is pressed against the mucosa, the elasticity of the tissue causes it to "rebound" and cover the broken fragment, causing it to disappear.
• Needle Gauge: A 30-gauge (short or long) needle was likely used. 30-gauge needles are significantly thinner and more prone to breakage than 25-gauge or 27-gauge needles. They are generally contraindicated for IANBs for this reason.

• Bending or Redirecting: The operator may have bent the needle prior to injection or attempted to change the direction of the needle while it was deeply embedded in the tissue (forceful redirection).
• 2. Immediate Management (Chairside)
• If the needle is not visible, do not attempt to probe or cut into the tissue to find it. This can push the needle deeper into the pterygomandibular space.
• Remain Calm: Do not alarm the patient.
• Keep Mouth Open: Instruct the patient to keep their mouth wide open. Do not allow them to close, as the pterygoid muscle contraction can draw the needle deeper. Place a bite block if possible.
• Visual Inspection: Look for the proximal end of the needle.
o If visible: Grasp it firmly with a hemostat (locking forceps) or cotton pliers and remove it.
o If NOT visible (Lost): Stop the procedure immediately. Do not attempt to incise or probe.
• Inform the Patient: Explain clearly what has happened and that you are referring them to a specialist for removal.
• Documentation: Thoroughly document the incident in the patient's notes (needle gauge used, patient movement, length of remaining fragment, etc.).
• 3. Specialist Treatment (Oral & Maxillofacial Surgeon)
• Refer the patient immediately to an Oral and Maxillofacial Surgeon (OMS). The treatment protocol typically involves:
• Imaging: The surgeon will order 3D imaging, typically a CBCT (Cone Beam CT) or CT scan, to triangulate the exact position of the needle in three dimensions. 2D radiographs (like Panorex) are often insufficient for surgical retrieval.
• Surgical Retrieval:
o Removal is generally required because the needle can migrate toward vital structures (carotid sheath, base of skull, or deeper spaces) and cause trismus, infection, or pain.
o This is usually performed under General Anesthesia in an operating room to ensure patient immobility and adequate access.
o Intraoperative navigation or radiopaque markers may be used to locate the fragment.
• Summary of Prevention
• Use a 25-gauge or 27-gauge long needle for IANBs (avoid 30-gauge).
• Never insert the needle to the hub (always leave 5mm of shaft visible).
• Do not bend the needle.
• Withdraw the needle almost entirely before changing direction.
22. In a 19-year-old patient, during impacted 48 surgery, cyanosis, fatigue, and low oxygen saturation develop after administration of prilocaine and articaine. What does this indicate, and how should it be treated?
• Based on the clinical presentation and the specific anesthetic agents used, this scenario indicates Acquired Methemoglobinemia.
• The following outlines the diagnosis, mechanism, and treatment protocol.
• 1. Diagnosis: Acquired Methemoglobinemia
• This is a condition where the iron within the hemoglobin molecule is oxidized from the ferrous (Fe^{2+}) state to the ferric (Fe^{3+}) state. Hemoglobin in the ferric state (methemoglobin) cannot effectively bind and transport oxygen, leading to functional anemia and tissue hypoxia.
• Key Indicator: The combination of cyanosis (bluish discoloration) and low oxygen saturation that does not improve significantly with supplemental oxygen is a hallmark sign.
• The Culprit: Prilocaine is the primary causative agent in this scenario. When metabolized, prilocaine produces o-toluidine, an oxidizing agent that stresses the red blood cells' reducing pathways. While Articaine can theoretically cause this, Prilocaine is classically associated with the condition in dental settings.
• 2. Immediate Management and Treatment
• The treatment depends on the severity of symptoms and the methemoglobin level, but given the presence of fatigue and cyanosis in a surgical setting, intervention is typically required.
• A. Initial Steps
• Stop Administration: Immediately cease the administration of any further local anesthetic.

• Supplemental Oxygen: Administer 100% oxygen. While this will not correct the methemoglobinemia itself, it maximizes the oxygen saturation of the remaining normal hemoglobin.
• Monitor: Assess vital signs and cardiac rhythm.
• B. Pharmacologic Treatment (The Antidote)
• If the patient is symptomatic (dyspnea, chest pain, altered mental status) or methemoglobin levels are usually $>30\%$:
• Primary Treatment: Methylene Blue
o Dosage: 1 to 2 mg/kg administered intravenously (IV) over 5 minutes.
o Mechanism: Methylene blue acts as a cofactor for the enzyme NADPH-methemoglobin reductase, accelerating the reduction of methemoglobin back to functional hemoglobin.
o Response: Improvement is usually rapid, often seen within 20 to 60 minutes.
• 3. Critical Contraindication: G6PD Deficiency
• Before administering Methylene Blue, it is vital to consider if the patient has a known Glucose-6-Phosphate Dehydrogenase (G6PD) deficiency.
• Risk: Methylene blue requires adequate G6PD levels to function. In G6PD-deficient patients, methylene blue is ineffective and can induce severe hemolysis (destruction of red blood cells).
• Alternative Treatment: If G6PD deficiency is known or strongly suspected, Ascorbic Acid (Vitamin C) can be used, though its action is much slower. In severe, life-threatening cases where Methylene Blue is contraindicated, hyperbaric oxygen therapy or exchange transfusion may be necessary.
• Disclaimer: *This information is for educational purposes and does not constitute medical advice. Medical emergencies require immediate management by qualified professionals.*
• Would you like me to detail the specific dosage calculations for the Methylene Blue or explain the mechanism of o-toluidine accumulation?

23. In a 45-year-old patient with uncontrolled diabetes, increasing pain, fistula formation, foul odor, and sequestrum formation develop a few weeks after extraction of mandibular left 37 and 38. What condition does this suggest, and how should it be treated?

• Based on the clinical presentation—particularly the combination of uncontrolled diabetes, delayed healing, and the specific presence of a sequestrum—the condition suggests Chronic Suppurative Osteomyelitis of the Mandible.
• Here is a breakdown of the diagnosis and the recommended management protocol.
• Diagnosis: Chronic Suppurative Osteomyelitis
• Osteomyelitis is an inflammation of the bone and bone marrow, usually caused by bacterial infection. In this case, the extraction sites (teeth 37 and 38) likely served as the portal of entry for bacteria.
• Why this diagnosis fits:
• Sequestrum Formation (Hallmark Sign): A sequestrum is a piece of necrotic (dead) bone that has become separated from the healthy bone. This occurs because the infection increases intramedullary pressure, causing blood vessel compression, ischemia, and bone death.
• Uncontrolled Diabetes: This is the critical predisposing factor. Hyperglycemia impairs leukocyte function (chemotaxis and phagocytosis), reduces blood flow (microvascular angiopathy), and delays collagen synthesis, making the patient highly susceptible to spreading infection.
• Fistula and Foul Odor: The foul odor typically indicates an anaerobic bacterial infection, while the fistula represents the body's attempt to drain the pus and necrotic debris from the bone.
• Mandibular Location: The mandible is significantly more prone to osteomyelitis than the maxilla because it has a poorer blood supply (centrifugal vs. centripetal) and dense cortical plates that confine the infection.
• Management and Treatment
• Treatment for chronic osteomyelitis is difficult because the "sequestrum" (dead bone) has no blood supply; therefore, systemic antibiotics cannot reach the bacteria trapped inside it.
• A combined surgical and medical approach is mandatory.
• 1. Surgical Intervention (Primary Treatment)
• Surgery is required to remove the source of infection.
• Sequestrectomy: The removal of the loose, dead bone (sequestrum). This is often the definitive treatment if the sequestrum is small and clearly separated.

• Debridement and Curettage: Removal of granulation tissue and necrotic debris from the extraction socket and surrounding marrow spaces.
• Saucerization: If the defect is large, the surgeon may smooth the sharp bony edges to eliminate "dead space" and allow the overlying soft tissue to collapse and heal over the bone.
• Extraction of adjacent teeth: If the infection has compromised the stability or bone support of adjacent teeth, they may also need to be extracted.
• 2. Medical Management (Antibiotic Therapy)
• Culture and Sensitivity (C&S): Pus or a swab from the deep tissue (not just the superficial fistula) must be sent for culture to identify the specific bacteria.
• Regimen: While waiting for culture results, empiric broad-spectrum antibiotics are started (often covering anaerobes, e.g., Clindamycin, Penicillin + Metronidazole, or Amoxicillin/Clavulanate).
• Duration: Antibiotic therapy is usually prolonged in chronic cases, often continuing for 4 to 6 weeks after surgical debridement to ensure eradication of the infection.
• 3. Systemic Management
• Glycemic Control: This is critical. The infection will be difficult or impossible to clear if the patient's blood glucose levels remain high. The patient should be referred to their physician or endocrinologist to stabilize their diabetes/HbA1c levels immediately.
• 4. Adjunctive Therapy (Case Dependent)
• Hyperbaric Oxygen Therapy (HBO): If the patient does not respond to standard surgery and antibiotics, or if the vascularity is severely compromised, HBO may be used to improve tissue oxygenation and stimulate angiogenesis (new blood vessel formation).
• Summary of Next Steps
• Referral: The patient requires immediate management by an Oral and Maxillofacial Surgeon.
• Imaging: A panoramic radiograph (OPG) or CBCT is needed to assess the extent of the bone destruction and locate the sequestrum.
• Surgery: Schedule for sequestrectomy and debridement.
• Would you like me to create a checklist for the patient's pre-operative assessment?

24. In a 23-year-old patient, after local anesthesia for tooth 28, it is learned that the patient has a history of drug allergy and atopic disease . Shortly after injection, generalized pruritus–urticaria, lip/face swelling, wheezing, and dizziness develop. How would you evaluate this presentation in terms of the most likely diagnosis, and how would you plan the emergency management in a dental clinic?

• Based on the clinical presentation described, here is an evaluation of the diagnosis and a step-by-step emergency management plan.
• I. Most Likely Diagnosis: Anaphylaxis
• The clinical picture strongly indicates Anaphylaxis, a severe, potentially life-threatening systemic hypersensitivity reaction.
• Diagnostic Reasoning:
• The diagnosis is supported by the rapid onset of symptoms involving two or more body systems immediately after exposure to a likely allergen (local anesthetic or preservatives like metabisulfite):
• Dermatological/Mucosal: Generalized pruritus (itching), urticaria (hives), and lip/face swelling (angioedema).
• Respiratory: Wheezing indicates bronchospasm and lower airway obstruction.
• Cardiovascular/Neurological: Dizziness suggests hypotension (low blood pressure) and cerebral hypoperfusion.
• Differential Diagnosis Note:
• Syncope (Fainting): While common, syncope typically presents with pallor and clammy skin, *not* urticaria, itching, or swelling.
• Local Anesthetic Toxicity: This usually manifests as CNS excitation (tremors, seizures) followed by depression, rather than the cutaneous signs (hives/swelling) seen here.
• II. Emergency Management in the Dental Clinic
• Speed is critical. The progression to cardiovascular collapse or complete airway obstruction can happen within minutes.
• 1. Immediate Stabilization (The "ABCDE" Approach)
• Stop the Procedure: Immediately cease injection and remove all materials from the patient's mouth.
• Positioning: Place the patient in a supine position with legs elevated (Trendelenburg) to improve venous return to the heart and brain.
o *Exception:* If the patient is struggling significantly to breathe or vomiting, a semi-

reclined position may be necessary, but avoid sitting them upright if they are hypotensive to prevent "empty ventricle syndrome" (cardiac arrest).
• Call for Help: Instruct a staff member to call Emergency Medical Services (911 or local equivalent) immediately. State "Anaphylaxis."
• 2. First-Line Pharmacological Intervention (Crucial)
• Epinephrine is the only life-saving medication for anaphylaxis. Do not delay its administration to look for antihistamines.
• Drug: Epinephrine (Adrenaline).
• Concentration: 1:1,000 (1 mg/mL).
• Dose: 0.3 mg to 0.5 mg for an adult.
• Route: Intramuscular (IM) injection into the anterolateral aspect of the thigh (vastus lateralis muscle). This site provides the fastest absorption.
o *Note:* If symptoms do not improve, the dose can be repeated every 5–15 minutes.
• 3. Respiratory Support
• Oxygen: Administer 100% oxygen via a non-rebreather mask at a high flow rate (10–15 L/min).
• Bronchodilators: If wheezing persists *after* epinephrine, administer a β_2-agonist inhaler (e.g., Albuterol/Salbutamol, 2–4 puffs) to help relieve bronchospasm.
• 4. Circulatory Support
• Monitor Vitals: Attach pulse oximeter and blood pressure cuff. Monitor heart rate, blood pressure, and oxygen saturation continuously.
• CPR: If the patient becomes unconscious and pulseless, begin Basic Life Support (BLS) immediately (compressions and rescue breaths).
• 5. Secondary (Adjunctive) Medications
• These should *only* be administered after Epinephrine and after the patient is stabilized or while waiting for EMS. They prevent biphasic (recurrent) reactions but do not treat the acute attack.
• Antihistamines: Diphenhydramine (Benadryl) 25–50 mg IM or IV.
• Corticosteroids: Hydrocortisone 100 mg IV or Prednisone orally (if the patient can swallow safely).
• Summary of Action Plan
• STOP procedure & POSITION patient (Supine, legs up).
• INJECT EPINEPHRINE (0.3 mg IM, Thigh).
• CALL EMS (911).
• GIVE OXYGEN.
• MONITOR & prepare for CPR.
• Would you like me to detail the specific dosage calculations for pediatric patients or discuss the prevention protocols for patients with known atopic history?

25. A 29-year-old patient presents two weeks after extraction of tooth 18 with the complaint that liquids consumed come out through the nose. CBCT shows an oroantral communication measuring 6 mm. What complication does this represent, and how should it be treated?

As an oral and maxillofacial surgeon, here is the clinical assessment and textbook-based management for your patient's presentation.

Clinical Diagnosis

The complication presented is an Oroantral Fistula (OAF). While it originated as an acute Oroantral Communication (OAC) following the extraction of the maxillary third molar, the two-week timeframe is the critical diagnostic factor. Epithelialization of a communication tract typically begins within 48 to 72 hours. By the two-week mark, a mature, epithelialized fistulous tract has formed. The classic pathognomonic symptom of liquid regurgitation through the nose confirms the patency of this tract.

Pre-Surgical Management

A 6 mm defect that is two weeks old is highly unlikely to close spontaneously and requires definitive surgical closure. However, immediate surgery is contraindicated if the maxillary sinus is actively infected.

Evaluate the CBCT for signs of maxillary sinusitis, such as mucosal thickening, opacification, or fluid levels.

If infection or heavy inflammation is present, prescribe a pre-operative regimen of antibiotics (typically Amoxicillin/Clavulanate), systemic decongestants, and saline nasal sprays.

Ensure the sinus is healthy and well-ventilated before proceeding with surgical closure to prevent flap failure.

Surgical Treatment Options

During surgery, the epithelialized lining of the fistulous tract must be completely excised before

closure, or the fistula will inevitably recur. Textbook surgical options for a 6 mm defect in the posterior maxilla include:
Buccal Advancement Flap (Rehrmann Flap): The most traditional approach. A broad-based trapezoidal mucoperiosteal flap is raised from the buccal aspect. The periosteum is horizontally scored at the base to release tension, allowing the flap to be advanced coronally over the defect and sutured over healthy bone.
Buccal Fat Pad Flap: Highly indicated for posterior maxillary defects. The buccal fat pad is mobilized and sutured over the bony defect. This technique has an excellent success rate, rapid epithelialization, and the major advantage of preserving vestibular depth.
Palatal Pedicled Flap: A full-thickness flap rotated from the palate, based on the greater palatine artery. It provides excellent tissue thickness but leaves a painful donor site that must heal by secondary intention.
Post-Operative Protocols
Strict adherence to sinus precautions is mandatory for 10 to 14 days to prevent pressure differentials from disrupting the flap.
Instruct the patient to completely avoid blowing their nose, smoking, or using straws.
Advise the patient to sneeze with their mouth open.
Prescribe prophylactic antibiotics and nasal decongestants to maintain sinus ostium patency.
Considering the location at the tooth 18 extraction site, which of these surgical flap techniques would you lean toward utilizing in your practice?

26. In a 52-year-old patient with uncontrolled diabetes, five days after bilateral mandibular 38 and 48 extractions, tongue swelling, dyspnea, and "board-like" induration/swelling of the neck develop. Which life-threatening infection does this suggest, and what is the appropriate emergency management?

• Based on the clinical presentation, the diagnosis is Ludwig's Angina.
• This is a rapidly spreading, life-threatening cellulitis of the floor of the mouth that involves the submandibular, sublingual, and submental spaces bilaterally.
• 1. Diagnosis: Ludwig's Angina
• The specific combination of symptoms points directly to this diagnosis:
• "Board-like" Induration: Unlike a simple abscess which is fluctuant (soft/fluid-filled), Ludwig's Angina presents as a firm, "woody" or board-like cellulitis because the infection is trapped within the fascial spaces.
• Tongue Swelling/Elevation: The infection involves the sublingual space, pushing the tongue up and backward against the palate.
• Dyspnea: The posterior displacement of the tongue causes acute airway obstruction, which is the primary cause of mortality.
• Etiology (Teeth 38 & 48): The roots of the mandibular second and third molars often lie below the mylohyoid line. Infection here bypasses the sublingual space and goes directly into the submandibular space, facilitating rapid spread.
• Risk Factor: Uncontrolled diabetes impairs neutrophil function, allowing the infection to spread aggressively.
• 2. Emergency Management Protocol
• This is a medical and surgical emergency. Management must be simultaneous and aggressive.
• A. Airway Management (Primary Priority)
• The greatest threat is airway occlusion.
• Assessment: Do not wait for stridor or cyanosis. Early security is vital.
• Intubation: Blind oral intubation is contraindicated due to anatomy distortion and bleeding risk. Fiberoptic nasal intubation (awake) is often the preferred method.
• Surgical Airway: If intubation is not possible due to severe edema, an emergency tracheostomy or cricothyrotomy must be performed immediately.
• B. Surgical Decompression
• Antibiotics alone are insufficient because of the pressure within the fascial compartments.
• Incision and Drainage (I&D): Wide exposure is required to release tension, even if frank pus is not yet present.
• Technique: Incisions are typically made in the submental and bilateral submandibular regions. The mylohyoid muscle may need to be divided to connect the spaces and allow decompression.
• C. Aggressive Antibiotic Therapy
• High-dose intravenous antibiotics are required immediately to cover oral flora (streptococci, staphylococci, and anaerobes like *Bacteroides*).
• Common Regimens:

o Ampicillin-Sulbactam (Unasyn)
o Penicillin G + Metronidazole
o Clindamycin (if penicillin-allergic)
o *Note:* In a diabetic patient, coverage for Klebsiella or other gram-negatives may also be considered.
• D. Systemic Support
• Glycemic Control: Aggressive management of blood glucose is essential to restore immune function and aid healing.
• Fluid Resuscitation: To manage sepsis and dehydration (due to dysphagia).
• Summary Table

Feature	Details
Involved Spaces	Bilateral Submandibular, Sublingual, Submental
Key Sign	"Woody" or "Board-like" neck swelling (no fluctuance)
Primary Risk	Asphyxia due to airway obstruction
Immediate Action	Secure Airway (Fiberoptic/Tracheostomy) + Surgical Drainage

• Would you like me to detail the specific anatomical boundaries of the submandibular spaces to explain why the infection spreads so easily in this region?

27. In a 22-year-old patient, after an inferior alveolar nerve (IAN) block administered for mandibular right 48, difficulty closing the eye and drooping of the corner of the mouth are observed. How can the cause of this finding be explained, and how should it be managed?

• This clinical presentation—difficulty closing the eye and drooping of the mouth corner immediately following an Inferior Alveolar Nerve (IAN) block—is characteristic of Transient Facial Nerve Palsy.
• This occurs when the facial nerve (Cranial Nerve VII) is inadvertently anesthetized. Below is the explanation of why this happened and the specific protocol for managing it.
• 1. Anatomical Explanation of the Cause
• The cause is a procedural error related to the depth and angulation of the needle insertion.
• The Mechanism: The facial nerve exits the skull via the stylomastoid foramen and travels forward, branching out within the substance of the parotid gland. The parotid gland wraps around the posterior border of the mandibular ramus.
• The Error: During the IAN block, if the needle is inserted too posteriorly (too deep) or deflected, it can bypass the mandibular ramus and penetrate the capsule of the parotid gland.
• The Result: The local anesthetic solution is deposited into the parotid gland rather than the pterygomandibular space. This anesthetizes the branches of the facial nerve (specifically the temporal, zygomatic, and buccal branches), leading to unilateral paralysis of the muscles of facial expression.
o Drooping mouth: Paralysis of the buccal/mandibular branches affects the orbicularis oris.
o Inability to close eye: Paralysis of the temporal/zygomatic branches affects the orbicularis oculi, causing a loss of the blink reflex.
• 2. Management Protocol
• While the condition is temporary and will resolve as the anesthetic wears off, immediate management is critical to prevent injury to the eye and to calm the patient.
• A. Immediate Communication & Reassurance
• Stop the procedure: Do not administer more anesthetic immediately.
• Reassure the patient: The patient may fear they are having a stroke. Calmly explain that this is a known, temporary side effect of the anesthesia affecting a nearby nerve and that it will return to normal when the numbing wears off (typically 2–4 hours).
• B. Eye Protection (Critical Step)
• The primary medical risk is corneal desiccation (drying) or abrasion because the patient has lost the blink reflex (lagophthalmos).
• Manual Closure: Ask the patient to manually close their eyelid periodically with their finger to keep the eye moist.
• Taping/Patching: If the procedure will continue or if the anesthesia is long-acting, you should tape the eye shut or apply an eye patch to protect the cornea from dust and debris.
• C. Evaluate Anesthesia
• Since the solution was deposited into the parotid gland, the IAN block itself often fails (the tooth #48 will likely *not* be numb).
• If you decide to proceed with treatment, you must re-administer the block, correcting the technique (contact bone earlier to ensure you are not too deep), but generally, it is often

prudent to postpone treatment if the patient is highly distressed.
• D. Documentation
• Clearly document the incident, the specific symptoms (facial weakness, loss of blink reflex), the instructions given to the patient (eye care), and the resolution time in the patient's chart.
• Summary Table

Feature	Detail
Diagnosis	Transient Facial Nerve Palsy (CN VII Anesthesia)
Procedural Error	Needle insertion was too deep/posterior; entered Parotid Gland.
Key Risk	Corneal abrasion due to loss of blink reflex (Orbicularis Oculi paralysis).
Primary Action	Protect the eye (patch/tape) and reassure the patient.

• Would you like me to detail the specific landmarks to look for to prevent injecting too posteriorly in future IAN blocks?

28. In a 27-year-old patient, within a few days after a difficult 28 extraction, temporomandibular joint clicking, locking, and pain develop. By what mechanism can this be explained, and how should it be managed?

• *Disclaimer: I am an AI, not a doctor or dentist. The following information is for educational purposes only and does not constitute medical advice. Please consult with an oral and maxillofacial surgeon or a dentist specializing in Temporomandibular Disorders (TMD) for a proper diagnosis and treatment plan.*
• In clinical practice, the development of temporomandibular joint (TMJ) symptoms—such as clicking, locking (trismus or closed lock), and pain—following a difficult extraction is a known complication. This is often categorized as an acute TMJ injury resulting from "macrotrauma."
• Here is the explanation of the mechanism and the standard management protocols.
• Mechanism of Injury
• The symptoms described (clicking, locking, pain) suggest an Internal Derangement of the TMJ, likely precipitated by the mechanics of the extraction procedure.
• Acute Macrotrauma & Hyperextension: A "difficult" extraction often implies prolonged wide mouth opening or the application of significant force to the mandible. This can overstretch the ligaments and capsule of the TMJ.
• Anterior Disc Displacement: The most likely specific mechanism is the anterior displacement of the articular disc.
o During the extraction, if the condyle was pulled too far forward or subjected to heavy downward force, the posterior attachment of the disc may have been stretched or torn.
o Clicking: Indicates the condyle is slipping on and off the displaced disc (displacement *with* reduction).
o Locking: If the disc becomes stuck in front of the condyle and prevents full opening, this is displacement *without* reduction (closed lock).
• Traumatic Synovitis/Capsulitis: The physical stress causes acute inflammation of the synovial tissues and the retrodiscal pad (the tissue behind the disc), leading to significant pain and swelling within the joint space.
• Muscle Spasm (Myospasm): The trauma can trigger a protective reflex spasm in the muscles of mastication (particularly the lateral pterygoid), which can physically pull the disc out of alignment and restrict movement.
• Management Strategies
• Management for acute TMJ injury post-extraction is typically conservative and focused on reducing inflammation and restoring normal joint function.
• 1. Immediate Conservative Therapy (Home Care)
• Soft Diet: The patient should strictly adhere to a non-chew diet (liquids, purees, soft foods) for 2–4 weeks to minimize joint loading.
• Restricted Opening: Advise the patient to limit mouth opening. They should support their chin with a hand when yawning to prevent hyperextension.
• Thermal Therapy:
o Ice: Applied for the first 24–48 hours to reduce acute inflammation and swelling.
o Moist Heat: Applied after the acute phase (after 48 hours) to relax stiff muscles and increase blood flow.
• 2. Pharmacological Management
• NSAIDs: Non-steroidal anti-inflammatory drugs (e.g., Ibuprofen, Naproxen) are the first line of defense to treat the capsulitis/synovitis.
• Muscle Relaxants: If muscle spasm is a major component contributing to the locking, a

short course of muscle relaxants (e.g., cyclobenzaprine) may be prescribed.
• 3. Professional Interventions
• If home care does not resolve the locking or pain within a short period:
• Manual Reduction: If the patient is experiencing an acute "closed lock" (unable to open), an oral surgeon may perform a manual manipulation to recapture the disc.
• Occlusal Splint Therapy: A stabilization splint (bite guard) may be fabricated to unload the joint, reduce muscle activity, and position the condyle in a more stable relationship with the disc.
• Physical Therapy: Gentle range-of-motion exercises, ultrasound, or laser therapy can help rehabilitate the joint.
• Arthrocentesis: In persistent cases where the joint remains "stuck" due to inflammatory pressure (suction cup effect), arthrocentesis (joint lavage/washing) is a minimally invasive procedure used to wash out inflammatory mediators and lubricate the joint.
• Summary
• The condition is likely acute anterior disc displacement with inflammation caused by the trauma of the extraction. The immediate goal is to reduce inflammation and unload the joint to allow the tissues to heal.
• Would you like me to create a patient instructions checklist that summarizes these home care steps?

29. In a 28-year-old patient, during mandibular left 38 surgery, prolonged use of a bur with inadequate water cooling results in a localized area of necrotic bone. Which complication is this consistent with, and how should it be managed?

• Based on the scenario described, the clinical presentation is consistent with Thermal Osteonecrosis (also known as "Bone Burn"), which typically results in the formation of a Sequestrum.
• This occurs when the temperature of the bone exceeds the critical threshold (approximately 47°C for one minute) during drilling, causing the death of osteocytes and subsequent necrosis of the osseous tissue.
• Complication: Thermal Osteonecrosis / Sequestrum Formation
• Unlike Alveolar Osteitis (Dry Socket), which is primarily the disintegration of the blood clot, thermal osteonecrosis involves actual structural damage to the jawbone. The localized area of necrotic bone will eventually separate from the healthy living bone; this detached piece of dead bone is called a sequestrum.
• Management Protocol
• The management strategy depends on the size of the necrotic area and the presence of infection, but generally follows these stages:
• 1. Conservative Management (Initial Phase)
• If the area is small and not clinically infected, the body may heal on its own or the sequestrum may spontaneously exfoliate.
• Irrigation: Frequent irrigation with saline or 0.12% chlorhexidine to keep the wound clean and reduce bacterial load.
• Analgesics: Management of pain, which can be significant.
• Antibiotics: Typically prescribed only if there are signs of secondary infection (swelling, purulence, systemic fever). They are generally ineffective for the dead bone itself as it has no blood supply.
• 2. Surgical Intervention (Sequestrectomy)
• If the necrotic bone does not dissolve or exfoliate spontaneously, it acts as a foreign body and prevents soft tissue healing.
• Waiting Period: The clinician usually waits until the sequestrum has fully demarcated (separated) from the healthy bone. Attempting to remove it too early can damage healthy bone and cause bleeding.
• Removal: Once the sequestrum is mobile, a minor surgical procedure is performed to remove the dead bone fragment (sequestrectomy).
• Debridement: The underlying healthy bone is lightly curetted to promote fresh bleeding, which brings healing cells to the area.
• 3. Long-term Healing
• Once the dead bone is removed, the defect typically heals by secondary intention (granulation tissue fills the void).
• Summary
• Diagnosis: Thermal Osteonecrosis (leading to Sequestrum).
• Key Cause: Frictional heat from the bur without sufficient irrigation.
• Treatment: Pain control, infection prevention, and eventual removal of the dead bone

fragment (sequestrectomy).
• Would you like me to outline the key preventative measures to avoid thermal necrosis in future surgeries?

30. In a 23-year-old patient, intraosseous anesthesia is administered during mandibular 38 extraction. Ten days later, the patient presents with an unhealed area of exposed bone at the extraction site and increasing pain. Which complication is this most likely to indicate, and how should management be planned?

Based on the clinical presentation and the specific procedure history, here is the assessment of the complication and the appropriate management plan.

Likely Diagnosis: Focal Osteonecrosis (Bone Sequestrum)

While Alveolar Osteitis (Dry Socket) is a common complication involving exposed bone, the timing (10 days post-op) and the specific use of intraosseous anesthesia strongly point to Focal Osteonecrosis leading to the formation of a Sequestrum (a piece of dead bone that has become separated from sound bone).

Pathophysiology

This complication is directly related to the intraosseous anesthesia technique. It typically occurs due to one of two mechanisms:

1. Ischemia: The high concentration of vasoconstrictor (often epinephrine) deposited directly into the cancellous bone causes localized lack of blood supply, leading to bone death.
2. Thermal Injury: The perforation of the cortical plate required for the intraosseous needle can generate excessive heat if performed without adequate coolant or with a dull drill, resulting in thermal necrosis of the surrounding bone.

The "increasing pain" at Day 10 is caused by the body's inflammatory reaction as it attempts to separate the necrotic bone fragment (the sequestrum) from the healthy living bone. The soft tissue cannot heal over the dead bone, leaving it exposed.

Management Plan

The goal of management is to facilitate the removal of the necrotic bone and promote soft tissue closure.

1. Conservative Management (Initial Phase)

If the exposed bone is firm and not yet mobile, aggressive surgical removal can damage healthy surrounding bone.

• Irrigation: Gently irrigate the area with saline or chlorhexidine to remove food debris and reduce bacterial load.
• Analgesia: Prescribe NSAIDs (e.g., Ibuprofen) for pain management.
• Topical Antiseptics: Prescribe a 0.12% Chlorhexidine gluconate rinse to keep the site clean.

2. Sequestrectomy (Intervention Phase)

Once the sequestrum has spontaneously separated (usually indicated by mobility of the bone fragment), it acts as a foreign body and must be removed.

• Assessment: Probe the exposed bone gently. If it is loose, it can often be lifted out easily with forceps.
• Debridement: If the bone acts as a sharp irritant but is not fully loose, the sharp edges may be smoothed (alveoloplasty) to prevent trauma to the tongue or cheek.
• Removal: Once the dead bone is removed, the healthy granulation tissue underneath is exposed, and healing typically proceeds rapidly.

Differential Diagnosis Note

• Alveolar Osteitis (Dry Socket): Usually presents earlier (Days 3–5) and involves the disintegration of the blood clot. While the pain is severe, it typically does not present with a distinct, unhealed bony spicule persisting at Day 10.
• Osteomyelitis: Would typically present with systemic signs (fever, lymphadenopathy) and more diffuse swelling/pain, rather than a localized defect.

Would you like me to explain the specific protocols for performing intraosseous anesthesia to minimize the risk of thermal necrosis in future cases?

COPILOT QUESTIONS AND ANSWERS:

1. In a 27-year-old patient, during surgical extraction of an impacted mandibular right third molar, an air-driven high-speed handpiece is used for bone removal. Shortly after the procedure, sudden swelling develops in the right cheek and submandibular region, with palpable crepitus on examination. What complication does this presentation suggest, by what mechanism does it occur, and how should it be managed?

Complication: The sudden swelling with palpable crepitus in the cheek and submandibular region following use of an air-driven high-speed handpiece strongly suggests subcutaneous

emphysema.
Mechanism: During bone removal with an air-turbine handpiece, compressed air can be forced into the soft tissues through the surgical site. In mandibular third molar surgery, air may dissect along fascial planes into the buccal, submandibular, and even cervical regions. The presence of crepitus on palpation is pathognomonic.
Clinical Features:
Rapid onset swelling during or immediately after surgery.
Soft, diffuse swelling with palpable crackling sensation (crepitus).
Usually painless, but may cause discomfort or anxiety.
In severe cases, air may spread to the parapharyngeal or mediastinal spaces.
Management Principles (Based on Standard Oral Surgery Texts):
Immediate Measures:
Stop use of the air-driven handpiece.
Reassure the patient; the condition is usually self-limiting.
Monitor for airway compromise if swelling extends to deeper spaces.
Medical Management:
Prophylactic antibiotics to prevent secondary infection (air can introduce oral flora into deeper tissues).
Analgesics as needed.
Advise the patient to avoid activities that increase intraoral pressure (nose blowing, forceful coughing, using straws).
Observation:
Most cases resolve spontaneously within 2–7 days as the air is resorbed.
Follow-up to ensure resolution and exclude progression to deeper spaces.
Prevention:
Avoid air-turbine handpieces for surgical bone removal in the mandible; use electric surgical handpieces or piezoelectric units instead.
Summary: This presentation represents subcutaneous emphysema caused by the inadvertent introduction of compressed air into fascial planes during mandibular third molar surgery with an air-driven handpiece. Management is conservative: discontinue air use, prescribe antibiotics, reassure the patient, and monitor for resolution. Prevention relies on using alternative instruments for bone removal.
Would you like me to expand on the clinical signs of subcutaneous emphysema, the management protocol, or the preventive measures in more detail?Clinical Interpretation (Textbook-Based):
Complication: The sudden swelling with palpable crepitus in the cheek and submandibular region following use of an air-driven high-speed handpiece strongly suggests subcutaneous emphysema.
Mechanism: During bone removal with an air-turbine handpiece, compressed air can be forced into the soft tissues through the surgical site. In mandibular third molar surgery, air may dissect along fascial planes into the buccal, submandibular, and even cervical regions. The presence of crepitus on palpation is pathognomonic.
Clinical Features:
Rapid onset swelling during or immediately after surgery.
Soft, diffuse swelling with palpable crackling sensation (crepitus).
Usually painless, but may cause discomfort or anxiety.
In severe cases, air may spread to the parapharyngeal or mediastinal spaces.
Management Principles (Based on Standard Oral Surgery Texts):
Immediate Measures:
Stop use of the air-driven handpiece.
Reassure the patient; the condition is usually self-limiting.
Monitor for airway compromise if swelling extends to deeper spaces.
Medical Management:
Prophylactic antibiotics to prevent secondary infection (air can introduce oral flora into deeper tissues).
Analgesics as needed.
Advise the patient to avoid activities that increase intraoral pressure (nose blowing, forceful coughing, using straws).
Observation:
Most cases resolve spontaneously within 2–7 days as the air is resorbed.
Follow-up to ensure resolution and exclude progression to deeper spaces.
Prevention:
Avoid air-turbine handpieces for surgical bone removal in the mandible; use electric

surgical handpieces or piezoelectric units instead.
Summary: This presentation represents subcutaneous emphysema caused by the inadvertent introduction of compressed air into fascial planes during mandibular third molar surgery with an air-driven handpiece. Management is conservative: discontinue air use, prescribe antibiotics, reassure the patient, and monitor for resolution. Prevention relies on using alternative instruments for bone removal.

2. In a 25-year-old patient, during removal of a fractured apical root fragment after extraction of tooth 28, the fragment is displaced apically and separated from the socket. Radiographs show that the fragment has been displaced into the maxillary sinus. What clinical pathway should be followed in this situation?

• Immediate action: confirm location with a CBCT, start sinus precautions and antibiotics, and arrange prompt surgical retrieval (either intraoral approach/Caldwell-Luc or endoscopic transnasal FESS depending on accessibility and symptoms) with an oral-maxillofacial surgeon and/or ENT — early removal is preferred to prevent acute or chronic maxillary sinusitis. pmc.ncbi.nlm.nih.gov International Online Medical Council (IOMC)
• Clinical pathway (step-by-step)
• 1. Confirm and localize
• Obtain a cone-beam CT (CBCT) or maxillary CT to precisely locate the fragment within the sinus, assess its relation to the ostium, and look for sinus mucosal reaction or oroantral communication. Accurate 3D imaging guides choice of approach. ResearchGate International Online Medical Council (IOMC)
• 2. Immediate conservative measures
• Advise sinus precautions: no nose-blowing, avoid Valsalva, sneeze with mouth open, no heavy lifting.
• Start empirical antibiotics covering oral flora (commonly amoxicillin-clavulanate unless contraindicated) and analgesics; consider nasal decongestant spray short term. These reduce risk of acute sinus infection while definitive management is arranged. International Online Medical Council (IOMC)
• 3. Decide timing and surgical approach
• Early removal (ideally within 24–72 hours) is generally recommended to reduce risk of infection, foreign-body reaction, and chronic sinus disease. pmc.ncbi.nlm.nih.gov International Online Medical Council (IOMC)
• If fragment is accessible through the extraction socket or alveolus and small, an intraoral approach with careful elevation and irrigation may succeed. If an oroantral communication exists, plan simultaneous closure (e.g., buccal advancement flap). ResearchGate
• If fragment is deep, adherent to sinus floor, near ostium, or not retrievable intraorally, refer for endoscopic transnasal removal (FESS) — this is less morbid than an open Caldwell-Luc and allows inspection/clearance of sinus disease. Open (Caldwell-Luc) approaches are reserved for cases where endoscopic access is not feasible. Choice should be made jointly by OMFS/dental surgeon and ENT. pmc.ncbi.nlm.nih.gov ResearchGate
• 4. Operative and postoperative care
• Coordinate care with ENT/OMFS; intraoperative irrigation and removal of any infected mucosa if present.
• If oroantral fistula is present, close it at the same session or plan repair soon after retrieval. ResearchGate
• Continue antibiotics, nasal care, and follow-up imaging or endoscopic review until sinus symptoms resolve.
• Decision points and red flags
• Immediate referral if the patient has fever, purulent nasal discharge, severe facial pain, or signs of spreading infection. pmc.ncbi.nlm.nih.gov
• Consider observation only in very select, asymptomatic cases with tiny fragments deeply embedded and high surgical risk — but this is uncommon and requires informed consent and close ENT follow-up. International Online Medical Council (IOMC)
• Risks, complications, and patient counseling
• Risks if left in situ: acute or chronic maxillary sinusitis, foreign-body granuloma, persistent oroantral fistula, mucocele formation. pmc.ncbi.nlm.nih.gov ResearchGate
• Procedure risks: bleeding, persistent fistula, sensory disturbance, need for secondary sinus surgery. Explain rationale for imaging and timely referral.
• Bottom line: confirm with CBCT, start antibiotics and sinus precautions, and arrange

prompt retrieval via the least-invasive effective route (intraoral if accessible; otherwise endoscopic FESS; Caldwell-Luc only if necessary) in coordination with ENT/OMFS to minimize sinus complications. pmc.ncbi.nlm.nih.gov International Online Medical Council (IOMC)

3. In a 29-year-old patient, during extraction of a deeply impacted tooth 48 that is related to the inferior alveolar nerve, a fractured apical root fragment is displaced apically and separated from the socket during attempted retrieval. Radiographs show that the fragment has been displaced into the inferior alveolar nerve canal. What clinical pathway should be followed, and what should the treatment approach be?

• Immediate priority: stop further manipulation, obtain a CBCT to precisely localize the fragment, assess and document inferior alveolar nerve (IAN) function, inform the patient, and urgently refer to an oral and maxillofacial surgeon (or tertiary centre in Ankara) for joint decision-making; if the patient has new or worsening neurosensory deficit or infection, plan prompt surgical removal (endoscopic-assisted or microsurgical) — if asymptomatic and removal poses high risk to the nerve, conservative retention with close follow-up is an acceptable option.

• Clinical pathway (stepwise)

• Stop further attempts at blind retrieval immediately to avoid additional displacement or nerve injury. Document the event in the chart and take intraoperative radiographs.

• Neurological assessment: perform and record baseline IAN sensory testing (light touch, two-point discrimination, pinprick) for lower lip/chin. If new paresthesia, anesthesia, or dysesthesia is present, treat as urgent. MDPI

• Imaging: obtain a CBCT (cone-beam CT) as soon as possible to define exact position relative to the mandibular canal and cortical plates. CBCT guides whether a safe intraoral approach is feasible.

• Specialist referral and informed consent: refer immediately to an oral & maxillofacial surgeon (or ENT/neuronal microsurgeon if available). Discuss risks/benefits with the patient and document alternatives (immediate removal vs observation).

• Treatment approach — decision framework

Option Indications Advantages Risks / Notes
Removes foreign body, may relieve
compression; endoscopic assistance
improves visualization and reduces bone
removal.
Neurological deficit,
Risk of further IAN
injury; requires
specialist surgery.
Immediate surgical removal (intraoral ± endoscopic-assisted)
infection, fragment protruding through canal cortex, patient
symptoms or
medicolegal/psychological
concern
Requires long-term
follow-up, patient
counselling, and
imaging; intervene if
symptoms develop.
Conservative retention (leave fragment in canal + monitor)
No neurosensory deficit, fragment fully intracanalar
Avoids immediate surgical trauma to IAN;
many fragments remain asymptomatic.
but stable, removal judged
higher risk than benefit

• Practical management steps and perioperative care

• If removal chosen: plan a controlled surgical approach under appropriate anaesthesia; use CBCT-guided planning and consider endoscopic assistance to minimize bone removal and nerve traction. Springer

• Medical therapy: short course of systemic corticosteroid may be considered for acute nerve inflammation (specialist decision); antibiotics if contamination/infection suspected. MDPI

• Follow-up: serial neurosensory exams and repeat imaging; if persistent or progressive

deficit after removal, consider microsurgical nerve exploration/repair referral. MDPI
• Communication, consent, and medicolegal points
• Fully inform the patient about risks (permanent IAN injury, altered sensation), alternatives, and expected follow-up; obtain written consent for any operative plan. Document all discussions and imaging.
• If you'd like, I can summarize this into a short checklist you can use in the clinic (CBCT timing, referral wording, consent points, and follow-up schedule).

4. In a 28-year-old patient using oral contraceptives, severe pain and a foul odor develop a few days after extraction of tooth 38. Which postoperative complication is this most consistent with, and how should treatment be planned?

• Most likely diagnosis: this presentation is classic for alveolar osteitis (dry socket) after a mandibular third molar extraction; treatment is focused on local socket management (irrigation and placement of a medicated dressing), analgesia, antiseptic rinses, and patient counselling rather than routine systemic antibiotics. dentalrx.ca Cochrane
• Key considerations and decision points
• Typical timing: pain and foul odor usually begin 2–4 days after extraction, especially after mandibular third molar removal. dentalrx.ca
• Major risk factors: oral contraceptive use, traumatic extraction, female sex, smoking, and poor clot formation or premature clot loss. Europe PMC dentalrx.ca
• Primary goals: relieve severe pain, protect exposed bone, control local contamination, and promote healing while avoiding unnecessary antibiotics. Cochrane dentalrx.ca
• Clinical diagnosis
• Most consistent complication: Alveolar osteitis (dry socket) — absence or disintegration of the post-extraction blood clot with exposed bone, intense localized pain, and fetid odor. Diagnosis is clinical based on history and exam. dentalrx.ca
• Recommended treatment plan
• Immediate chairside management
o Irrigate the socket gently with warm saline to remove debris and food. dentalrx.ca
o Debride necrotic tissue or loose clot carefully if present. dentalrx.ca
o Place a medicated intra-socket dressing (e.g., eugenol-containing dressings such as Alvogyl or other analgesic/antiseptic dressings) to provide immediate pain relief and protect exposed bone. Evidence supports pain reduction with some dressings though quality varies. Cochrane dentalrx.ca
• Analgesia
o Systemic analgesics: start or continue NSAIDs (ibuprofen) as first line; add short course opioids only if pain is severe and not controlled. Consider adjunct paracetamol. dentalrx.ca
• Antiseptic measures
o Chlorhexidine 0.12% rinse twice daily can reduce microbial load and help healing; in some protocols a chlorhexidine gel placed in socket is used for prevention and adjunctive care. Cochrane
• Antibiotics
o Do not prescribe systemic antibiotics routinely for uncomplicated dry socket. Reserve antibiotics for signs of spreading infection, systemic involvement, or immunocompromise. dentalrx.ca Cochrane
• Follow up
o Reassess in 48–72 hours; replace dressing as needed until pain subsides and granulation begins. Expect symptom improvement within a few days. dentalrx.ca
• Patient counselling and prevention
• Advise avoidance of smoking, vigorous rinsing, straws, and spitting until healed. dentalrx.ca
• For patients on oral contraceptives, consider scheduling elective extractions during the inactive pill week (low estrogen) when feasible to reduce risk; discuss risks but do not abruptly stop OCPs without medical advice. Europe PMC dentalrx.ca
• Red flags requiring escalation
• Fever, spreading swelling, trismus, lymphadenopathy, or systemic signs → treat as possible infection and consider antibiotics and urgent review. dentalrx.ca
• If you want, I can draft a short script for what to tell the patient at the chairside and a checklist for dressing materials and analgesic prescriptions.

5. In a 24-year-old patient, persistent numbness of the lower lip and chin region for three months following a difficult mandibular right 48 extraction suggests which neurological

complication, and how should this condition be managed?
• Persistent numbness of the lower lip and chin after a difficult lower right third-molar (48) extraction most likely represents an inferior alveolar/mental nerve injury (traumatic neuropathy); because symptoms have lasted three months, urgent specialist assessment is indicated and surgical exploration/repair should be considered if there is no clear recovery or if objective testing/imaging suggests nerve transection or compression. MDPI journalonsurgery.org
• Diagnosis
• Likely lesion: Inferior alveolar nerve (IAN) injury with involvement of its terminal branch, the mental nerve, producing lower-lip and chin numbness. MDPI
• Type: Traumatic neurosensory deficit ranging from neurapraxia (temporary conduction block) to axonotmesis or neurotmesis (partial or complete transection). MDPI
• Immediate actions (now)
• Refer promptly to an oral and maxillofacial surgeon or a peripheral nerve specialist for formal assessment; if you are in Ankara, seek evaluation at a tertiary dental/maxillofacial unit.
• Document baseline: detailed history, map sensory deficit, and perform standardized neurosensory tests (light touch, two-point discrimination, pinprick, thermal). MDPI
• Obtain imaging: CBCT or panoramic radiograph to look for retained root fragments, hematoma, or bony impingement on the canal. MDPI
• Conservative management (first 6–12 weeks, if partial deficit)
• Watchful waiting with close follow-up because many injuries improve within weeks to a few months. journalonsurgery.org
• Adjunctive measures: short course of systemic corticosteroids early (if within days), vitamin B complex, pain control, and counseling about prognosis; consider low-level laser therapy or neuromodulatory medications for dysesthesia under specialist guidance. MDPI
• When to consider surgical intervention
• No objective improvement by 3 months, progressive worsening, or evidence of nerve entrapment/transection on imaging or intraoral exam → surgical exploration and microsurgical repair (neurolysis, direct repair, or grafting) should be discussed. MDPI journalonsurgery.org
• Immediate exploration may be indicated earlier when there is clear iatrogenic transection, displaced root fragment within the canal, or severe persistent anesthesia with high risk of permanent deficit. MDPI
• Prognosis and counseling
• Partial injuries often recover over weeks–months; complete transections have a poorer prognosis without timely repair. journalonsurgery.org MDPI
• Explain expected timelines, possible outcomes (partial recovery, persistent numbness, or dysesthesia), and medicolegal aspects if relevant. MDPI
• Practical next steps for the patient
• Arrange urgent specialist referral (oral & maxillofacial surgery).
• Get CBCT/panoramic imaging and formal neurosensory testing.
• If no improvement or imaging suggests compression/transection, plan surgical exploration within weeks rather than further delay. MDPI journalonsurgery.org
• If you want, I can outline the specific neurosensory tests the specialist will perform, or draft questions to take to the referral appointment.

6. In a 26-year-old patient, following lingual retraction during extraction of tooth 38, numbness and loss of taste develop on the left half of the tongue. Which nerve injury does this suggest, and how should it be treated?
• Answer: This presentation indicates injury to the left lingual nerve (a branch of V3) with involvement of taste fibers carried by the chorda tympani (CN VII) that hitchhike on the lingual nerve; initial management is conservative observation and symptomatic care, but urgent referral to an oral/maxillofacial surgeon for early assessment is required because persistent or complete deficits may need microsurgical repair (ideally considered within the first 3 months). biologyinsights.com scienceinsights.org
• Key facts (diagnosis)
• Nerve involved: *Lingual nerve* (branch of the mandibular division of trigeminal nerve, V3) supplying general sensation to the anterior two-thirds of the tongue. Taste loss occurs because chorda tympani (facial nerve) taste fibers run with the lingual nerve to the tongue; both can be affected by lingual retraction or laceration. biologyinsights.com

• Pathophysiology
• Mechanism: traction, compression, contusion, or laceration during lingual retraction for lower third-molar (tooth 38) extraction.
• Clinical signs: immediate numbness/paresthesia of the ipsilateral anterior two-thirds of tongue and loss or alteration of taste on that side. biologyinsights.com
• Immediate management (first 0–2 weeks)
• Document baseline: perform sensory testing (light touch, pinprick, two-point discrimination) and taste testing if possible; photograph and record findings.
• Conservative measures: reassure the patient; observe for spontaneous recovery (many traction/neuropraxic injuries improve over weeks to months). Consider short course of systemic corticosteroids only if recommended by treating surgeon for acute inflammatory neuropathy (evidence variable). Supportive measures: oral hygiene, soft diet, topical agents for mucosal protection, and neuropathic pain control if painful dysesthesia develops. scienceinsights.org
• When to refer and surgical indications
• Urgent referral to an oral and maxillofacial surgeon or peripheral nerve specialist is indicated if there is complete anesthesia, severe dysesthesia, or no improvement within 4–8 weeks, or if intraoperative transection is suspected.
• Surgical options: early microsurgical exploration and primary neurorrhaphy (direct repair) or grafting if a segmental loss is found. Best outcomes are reported when repair is performed within the first 3 months after injury; delayed repairs have progressively worse sensory recovery. biologyinsights.com scienceinsights.org
• Prognosis & follow-up
• Prognosis: partial/traction injuries often recover partially or fully over weeks to months; complete transections have a guarded prognosis without timely repair. Taste fibers may recover more slowly and incompletely. Regular follow-up with serial sensory testing is essential. biologyinsights.com
• Practical next steps (actionable)
• Refer now to an oral/maxillofacial surgeon for clinical assessment and documentation.
• Start conservative care (protective oral measures, analgesia for neuropathic pain if needed) while awaiting specialist review.
• If no improvement by 4–8 weeks or if severe deficit was immediate and complete, plan for surgical exploration and possible repair within 3 months. biologyinsights.com scienceinsights.org
• If you want, I can summarize the typical sensory tests and a sample timeline for follow-up visits to bring to the specialist.

7. In a 26-year-old patient, a tooth is lost during extraction of tooth 28, and two days later CBCT shows the tooth in the infratemporal fossa. What does this finding indicate, and what is the appropriate treatment approach?

• Summary — This CBCT finding means the extracted upper third molar was iatrogenically displaced into the infratemporal fossa (a deep facial space); this is a potentially serious complication that usually requires prompt surgical retrieval by an oral and maxillofacial surgeon (intraoral approach preferred when feasible) after careful radiologic planning. pmc.ncbi.nlm.nih.gov DergiPark
• What the CBCT finding indicates
• Anatomy: The infratemporal fossa (ITF) is a deep space posterior to the maxilla containing muscles (pterygoids), the maxillary artery branches, pterygoid venous plexus, and branches of the mandibular nerve. A tooth located here is outside the alveolus and in close relation to vital structures. pmc.ncbi.nlm.nih.gov
• Clinical implication: The tooth is displaced into a deep soft-tissue compartment rather than “lost” in the mouth; it can cause pain, trismus, infection, hemorrhage, or further migration if not managed correctly. DergiPark
• Appropriate treatment approach (practical, stepwise)
• Confirm and map location with CBCT (already done). Use the CBCT to define exact position relative to the lateral pterygoid plate, zygomatic buttress, and vascular structures. Do not attempt blind probing or forceful retrieval in the clinic. pmc.ncbi.nlm.nih.gov
• Urgency and referral. Refer immediately to an experienced oral and maxillofacial surgeon (or an ENT/head-and-neck surgeon with maxillofacial experience). Early specialist management reduces risk of migration, infection, and vascular injury. DergiPark
• Decide timing: immediate vs delayed retrieval.
o Immediate retrieval under appropriate anesthesia is generally recommended when the tooth is accessible or the patient is symptomatic (pain, swelling, trismus,

bleeding). Early removal prevents infection and migration. pmc.ncbi.nlm.nih.gov
Taylor & Francis Online
o Delayed retrieval (short delay of 1–2 weeks) is sometimes chosen to allow fibrosis to stabilize the fragment if retrieval would be high-risk; this must be a deliberate specialist decision and requires close monitoring. pmc.ncbi.nlm.nih.gov
• Surgical approach and technique.
o Preferred: intraoral approach with an extended posterior maxillary/vestibular flap and careful blunt dissection to the ITF; removal under general anesthesia is common. Use CBCT guidance and gentle retraction of soft tissues; control for bleeding from the pterygoid plexus or maxillary artery branches.
pmc.ncbi.nlm.nih.gov DergiPark
o Alternatives: transoral endoscopic assistance or extraoral approaches are reserved for inaccessible or migrated fragments and are chosen by the surgeon based on imaging. Taylor & Francis Online
• Perioperative care. Start antibiotics if infection is suspected or as perioperative prophylaxis; provide analgesia, tetanus status check if indicated, and plan short-interval follow-up to monitor for infection, nerve or vascular complications. DergiPark
• Risks, patient counseling, and red flags
• Risks: hemorrhage, hematoma, persistent trismus, infection, injury to branches of V3, and need for more extensive surgery. pmc.ncbi.nlm.nih.gov DergiPark
• Red flags requiring urgent action: expanding swelling, severe pain, fever, progressive trismus, or signs of bleeding — these require immediate surgical review.
• Bottom line: This is a deep-space iatrogenic displacement that usually warrants specialist surgical retrieval planned from the CBCT; avoid blind attempts, refer urgently to an oral and maxillofacial surgeon, and manage perioperative infection/bleeding risks.
pmc.ncbi.nlm.nih.gov DergiPark Taylor & Francis Online

8. In a 34-year-old patient, a mandibular basal (inferior border) fracture occurs during extraction of a deeply impacted tooth 38 due to excessive elevator force. What should the emergency management approach be?

• Immediate priority: stop the extraction, protect the airway, control bleeding, and stabilize the mandible — do not continue forceful manipulation; arrange urgent assessment and definitive care by an oral and maxillofacial surgeon (likely requiring imaging and surgical fixation). jcda eMedicine
• First 5–10 minutes — emergency actions
• Stop the procedure immediately and remove the elevator; avoid further levering or attempts to force the tooth out. jcda
• Assess airway and breathing; if there is any airway compromise (swelling, bleeding, inability to open/close mouth), manage airway per emergency protocols and call for emergency help. eMedicine
• Control hemorrhage with direct pressure (gauze) and local measures; keep the patient upright and calm. Do not pack deep into the fracture site. eMedicine
• Stabilize the mandible manually (support the jaw) to limit fragment movement until definitive fixation is available. Avoid further manipulation of fragments. jcda
• Immediate assessment and investigations
• Obtain plain radiography (panoramic/OPG) as first-line to confirm fracture location and displacement; if available and the fracture is complex or near the inferior border, CT (CBCT/CT) is preferred for surgical planning. IntechOpen eMedicine
• Document the event (informed consent discussion, what happened, clinical findings) and inform the patient clearly about next steps. jcda
• Short-term medical management (while arranging definitive care)
• Analgesia (IV/IM or oral as appropriate) and anti-inflammatory medication. eMedicine
• Start broad-spectrum antibiotics if there is mucosal perforation, contamination, or open fracture risk. IntechOpen
• Tetanus status: update if indicated. eMedicine
• Definitive management (oral and maxillofacial surgery)
• Non-displaced, minimally mobile inferior border fractures may be managed conservatively with soft diet, immobilization (intermaxillary fixation [IMF] or elastic guiding), and close follow-up. IntechOpen
• Displaced or unstable inferior border fractures usually require open reduction and internal fixation (ORIF) with plates and screws to restore continuity and function. Early referral to OMFS is indicated. IntechOpen eMedicine
• Practical points, risks, and patient communication

• Do not attempt further extraction maneuvers once a basal fracture is suspected — this increases displacement and complicates repair. jcda
• Explain risks: infection, malocclusion, need for surgery, sensory disturbance (inferior alveolar nerve), and possible prolonged recovery. IntechOpen
• Arrange urgent transfer to a hospital with OMFS capability; if you are in Ankara, present to the nearest tertiary hospital emergency/OMFS unit for imaging and operative planning. eMedicine
• Quick checklist to hand to the receiving team
• Time and mechanism of injury; intraoperative notes; current vitals; radiographs if taken; antibiotics/analgesics given; tetanus status; photos of occlusion if possible. jcda eMedicine
• If you want, I can summarize this as a one-page handout for the emergency department or draft the exact wording to communicate to the patient and the OMFS team.

9. In a 28-year-old patient, one week after extraction of tooth 48, progressively worsening severe pain, trismus, fever, and cervical lymphadenopathy develop. The patient also has dysphagia/odynophagia, diffuse neck swelling, and chest pain–shortness of breath, raising suspicion of spread of infection through deep neck spaces to the mediastinum. Which diagnosis does this presentation most strongly suggest?

• Most likely diagnosis
• Descending necrotizing mediastinitis secondary to an odontogenic deep neck space infection (spread from the infected lower third molar).
• Why this fits
• Recent lower molar extraction with progressive severe pain, trismus, fever, cervical lymphadenopathy, dysphagia/odynophagia, and diffuse neck swelling points to a deep neck space infection (sublingual/submandibular/parapharyngeal/retropharyngeal).
• New chest pain and shortness of breath indicate extension inferiorly into the mediastinum, which is characteristic of descending necrotizing mediastinitis.
• Immediate actions (urgent)
• Secure the airway and arrange urgent transfer to hospital.
• Contrast CT scan of neck and chest to define extent of neck and mediastinal involvement.
• Start broad-spectrum IV antibiotics and obtain prompt surgical consultation (ENT/maxillofacial and thoracic surgery) for drainage/debridement.

10. In a 25-year-old patient, six weeks after mandibular 48 extraction, the patient reports food impaction and bleeding distal to tooth 47. Clinical examination reveals a ≥6 mm periodontal pocket distal to 47 with bleeding on probing; radiographs show localized bone loss distal to 47. What surgical and postoperative approach should be implemented in this case?

• Key decision factors for treatment
Attribute Resective surgery Regenerative surgery
Defect morphology
Non-contained, horizontal bone loss;
Contained vertical intrabony defect
thin or irregular bone
(1–3 walls or 3-wall)
Recreate lost attachment and bone
Primary goal Eliminate pocket by reshaping bone
and soft tissue
height
Predictability in young patient
High for pocket elimination and
High if defect is well contained and
long-term stability
patient compliant
Typical procedures
Distal wedge, apically positioned flap,
Flap access, debridement, bone graft
osteoplasty/ostectomy
± membrane (GTR)
More strict plaque control,
membrane care, longer
healing
Postop demands Standard wound care and plaque

control
• Recommended surgical approach
• 1. Preoperative assessment
• Re-evaluate the defect under local anaesthesia with a full periodontal chart and periapical radiograph or CBCT if needed to define defect walls.
• Decide based on defect morphology: if the defect distal to 47 is a non-contained or horizontal defect, plan resective surgery; if it is a deep, well-contained vertical intrabony defect, plan regenerative therapy.
• 2. If defect is non-contained or primarily horizontal (most common after third molar extraction)
• Perform a full-thickness mucoperiosteal flap with a distal wedge incision to remove redundant tissue and gain access.
• Thorough granulation tissue removal and root planing of the distal surface of 47.
• Perform osteoplasty/ostectomy to recontour the distal alveolar bone and reestablish a physiologic architecture that allows a shallow sulcus.
• Reposition flap apically if needed to reduce pocket depth and suture for primary closure.
• 3. If defect is a deep, contained vertical intrabony defect
• Raise a full-thickness flap, debride thoroughly.
• Place an appropriate bone graft (autograft/allograft/xenograft) and consider GTR with a resorbable membrane to promote regeneration of bone and attachment.
• Achieve tension-free primary closure with sutures.
• Intraoperative details and materials
• Flap: full-thickness mucoperiosteal; distal wedge to remove excess tissue.
• Bone work: osteoplasty for resective cases; careful ostectomy if necessary to remove undermining bone.
• Grafts/membranes: use when defect morphology is favorable for regeneration.
• Sutures: interrupted or mattress sutures; non-resorbable or resorbable depending on surgeon preference; plan removal at 7–10 days.
• Postoperative protocol
• Medications
o Analgesics: NSAID as needed (e.g., ibuprofen).
o Antibiotics: only if indicated (systemic infection risk, extensive surgery, or local protocol); commonly amoxicillin if prescribed.
• Oral hygiene and rinses
o Chlorhexidine 0.12% rinse twice daily for 1–2 weeks.
o Avoid mechanical cleaning of the surgical site for 7–10 days; resume gentle brushing after suture removal as instructed.
• Diet and activity
o Soft diet for 3–7 days; avoid chewing on the operated side until healing is adequate.
o No smoking; smoking impairs healing and compromises regenerative outcomes.
• Wound care
o Ice intermittently first 24 hours to reduce swelling.
o Avoid vigorous rinsing or spitting for 24 hours.
• Follow up and maintenance
• Suture removal at 7–10 days.
• Clinical review at 1 month, 3 months, and 6 months with periodontal probing and radiographic reassessment as indicated.
• If resective surgery performed expect pocket depth reduction and stable architecture; if regenerative therapy performed, monitor for membrane exposure and evaluate radiographic bone fill over 6–12 months.
• Enroll patient in periodontal maintenance with professional cleaning every 3 months initially, then individualized interval.
• Practical summary and recommendation
• First step: re-enter and inspect the defect to determine morphology.
• If non-contained: perform distal wedge + apically positioned flap with osteoplasty/ostectomy to eliminate the pocket.
• If contained vertical defect: perform regenerative surgery with bone graft and membrane (GTR).
• Postop care: chlorhexidine, analgesics, limited chewing, suture removal at 7–10 days, and close follow-up with periodontal maintenance.
• This plan balances predictable pocket elimination and the potential for regeneration when anatomy allows.

11. In a 29-year-old patient, recurrent bleeding from the extraction site begins approximately one week after extraction of mandibular left 38. What mechanism could explain this delayed bleeding, and how should it be managed?

• Delayed, recurrent bleeding one week after a lower third-molar extraction is most commonly due to secondary (infective) lysis of the clot or, less commonly, a developing arterial pseudoaneurysm; treat immediately with local haemostasis and antibiotics if infection is suspected, check coagulation/medication status, and refer for surgical/angiographic management if bleeding persists or is arterial.
• Key considerations and quick questions
• Was there fever, foul discharge, or increasing pain (suggests infection)?
• Is the patient on anticoagulants or has a bleeding disorder?
• Is the bleeding brisk/arterial (pulsatile) or oozing?
• Likely mechanisms
• Secondary haemorrhage from infection: bacterial enzymes and local inflammation can lyse the clot and prevent stable haemostasis, producing delayed bleeding about 5–10 days after extraction. Infection with clot breakdown is a common cause of late post-extraction bleeding. MSD Manuals
• Pseudoaneurysm or delayed arterial erosion: trauma to a vessel at extraction can form a contained arterial lesion that ruptures days later, producing recurrent or pulsatile bleeding; this is less common but important to recognise. Oral and Maxillofacial Surgery Clinics
• Systemic factors: resumed anticoagulant/antiplatelet therapy, undiagnosed coagulopathy, or medications that increase fibrinolysis can also present as delayed bleeding.
• Immediate management (first aid, same visit)
• Apply firm local pressure with gauze for 15–20 minutes; seat the patient upright and lean forward.
• If pressure fails, suture the socket to approximate tissues and protect the clot; place a haemostatic agent (oxidized cellulose, gelatin sponge) into the socket. Topical tranexamic acid mouthwash or soaked gauze can help stabilize clot by inhibiting fibrinolysis. Frontiers
• Irrigate gently to remove debris if infection suspected, then pack with antiseptic/haemostatic material.
• Assess for signs of infection (fever, purulence, swelling) and start empiric oral antibiotics if present. MSD Manuals
• Check medication and coagulation status (INR, platelet count) and liaise with the prescribing physician if anticoagulants were recently restarted.
• Definitive management and referral
• If bleeding is controlled with local measures and infection treated, follow up in 24–48 hours.
• If bleeding is recurrent, pulsatile, or uncontrolled, suspect arterial source/pseudoaneurysm — arrange urgent referral to oral and maxillofacial surgery or interventional radiology for angiography and embolization or operative ligation. Oral and Maxillofacial Surgery Clinics
• Document events, medications, and interventions; give clear return instructions for rebleeding or systemic symptoms.
• Risks and patient advice
• Untreated infection or arterial bleeding can lead to significant blood loss or sepsis. Encourage prompt evaluation in Ankara emergency dental/oral surgery services if bleeding recurs or is heavy. If the patient is hemodynamically unstable, call emergency services immediately.
• If you want, tell me whether the bleeding is pulsatile, whether there are infection signs, and the patient's medication list — I'll give a tailored step-by-step plan.

12. In a 22-year-old patient, soft tissue trauma–related tissue hemorrhage occurs during local anesthetic injection prior to mandibular 38 extraction. How would you proceed with the procedure in this situation?

• If the hemorrhage is small and controllable, stop the injection, apply firm intraoral pressure and ice, achieve hemostasis, and proceed only when bleeding and swelling are stable; if the hematoma is expanding, large, or obscures the field, postpone the extraction and manage the hematoma (cold then heat, analgesia, monitor) until it resolves (usually 7–14 days). This approach is standard in outpatient oral surgery settings in Ankara and elsewhere.
• Immediate steps (first 10 minutes)

• Stop the injection immediately and withdraw the needle. الجامعة المستنصرية
• Apply firm direct pressure with sterile gauze over the bleeding site for 10–15 minutes without interruption; maintain pressure rather than repeated checks. This is the single most effective immediate measure. Dr. Chetan
• Apply an external cold compress (ice pack) to the cheek over the area for 10–20 minutes on, 10 minutes off for the first 4–6 hours to limit hematoma expansion. Seattle Smiles Dental
• Assess airway, vision, and neurologic signs (facial nerve palsy, visual disturbance) and vital signs; if any compromise or rapidly expanding swelling occurs, escalate care and consider urgent referral. Springer
• Decision: proceed with extraction or postpone
• Proceed with extraction only if: bleeding is controlled, the surgical field is visible, the patient is hemodynamically stable, and you can obtain adequate anesthesia without further trauma. *Proceed cautiously.* Dr. Chetan Seattle Smiles Dental
• Postpone extraction if: hematoma is large or expanding, bleeding cannot be controlled with pressure, the field is obscured, trismus or significant swelling limits access, or the patient is anxious/unstable. Typical delay is 7–14 days to allow clot organization and resolution. Dr. Chetan Seattle Smiles Dental
• Subsequent management if hematoma present
• First 24–48 hours: continue cold compresses and firm pressure as needed; avoid heat. Avoid NSAIDs that impair platelet function if bleeding risk is a concern; use acetaminophen for pain when appropriate. Seattle Smiles Dental Springer
• After 48–72 hours: switch to warm moist heat to promote resorption and reduce trismus; begin gentle jaw exercises if trismus present. Seattle Smiles Dental
• Medications: analgesics as needed; antibiotics only if secondary infection risk or planned surgical intervention. Consider tetanus status per routine wound care. Springer Seattle Smiles Dental
• Anesthesia strategy for future attempt
• Avoid repeating the same block through the hematoma; wait until swelling resolves. Use alternative techniques (local infiltration, Gow-Gates or Akinosi approaches) or a different needle path once the hematoma has settled. Reassess bleeding risk and counsel the patient. Dr. Chetan الجامعة المستنصرية
• Key practical points
• Immediate firm pressure and ice are essential and often sufficient to control injection-related hematoma. Dr. Chetan Seattle Smiles Dental
• Do not proceed if visualization or access is compromised; postpone 7–14 days for resolution. Dr. Chetan Seattle Smiles Dental
• Escalate care for rapidly expanding hematoma, airway compromise, or neurologic/ocular symptoms. Springer
• If you want, I can convert this into a short chairside checklist you can print for your clinic in Ankara.

13. A 25-year-old patient presents two days after mandibular 48 extraction with limited mouth opening and difficulty eating. How should the patient be assessed and managed?

• Short answer: This is most consistent with postoperative trismus (limited mouth opening) after a lower third-molar extraction; assess for infection, hematoma, nerve or TMJ injury, and alveolar osteitis, start analgesia, soft diet, warm compresses and guided jaw-opening exercises, and give antibiotics only if clinical infection is present — refer urgently to oral & maxillofacial surgery (OMFS) if worsening, systemic signs, or inability to maintain nutrition. Spandidos Publications MDPI
• Quick checklist to guide your next steps
• Key questions to ask now: onset and progression of trismus; fever, swelling, pus, increasing pain; ability to swallow/maintain fluids; any bleeding after extraction; prior antibiotics or medical conditions.
• Immediate decision points: signs of systemic infection or spreading cellulitis → urgent referral/admission; severe inability to eat/drink → consider IV fluids and urgent OMFS review.
• 1) Initial assessment (history + exam)
• History: time since extraction (2 days), pain severity, fever, swelling, odour, prior antibiotics, medical history (immunosuppression, bleeding disorders).
• Examination: measure interincisal opening (normal ~35–55 mm); inspect socket for clot, food debris, pus; palpate for localized swelling/hematoma; check regional lymph nodes and temperature; assess TMJ pain and mandibular nerve function. Document interincisal

distance in mm. Spandidos Publications
• Red flags requiring urgent action: fever >38°C, spreading facial swelling, rapidly worsening trismus, dysphagia, airway compromise, systemic toxicity.
• 2) Differential diagnosis (most likely → less likely)
• Postoperative inflammatory trismus from muscle spasm (masseter/medial pterygoid) or local edema.
• Local infection (alveolar osteitis or abscess) — look for foul smell, exposed bone, pus.
• Hematoma or iatrogenic TMJ injury or coronoid impingement.
• Less likely: mandibular fracture, tetanus (rare).
• 3) Immediate management (outpatient if no red flags)
• Analgesia: regular NSAID (e.g., ibuprofen) ± paracetamol; consider short course opioid if severe pain. Start analgesics immediately.
• Local care: warm moist compresses to the cheek 10–15 min every 2–3 hours; soft/liquid diet; maintain oral hygiene with gentle saline rinses after 24 hours.
• Jaw exercises: gentle, graded mouth-opening exercises (hold maximal comfortable opening for 10–30 sec, repeat 5–10 times, 3–4 times/day) to reduce muscle spasm.
• Antibiotics: only if clinical infection (cellulitis/abscess) is present — choose according to local guidelines (e.g., amoxicillin ± metronidazole in many settings). Avoid routine prophylactic antibiotics for isolated trismus. MDPI Spandidos Publications
• 4) When to refer and follow-up
• Refer to OMFS within 24–48 hours if trismus persists/worsens despite measures, signs of abscess, systemic illness, or inability to maintain nutrition. Consider imaging (panoramic radiograph or CT) if suspected fracture/abscess. Persistent trismus >2 weeks may need physiotherapy or surgical drainage. Spandidos Publications
• Risks, counselling, and prevention
• Risks: untreated infection → abscess, spread to deep neck spaces; prolonged trismus → fibrosis.
• Counsel: expected improvement over 7–14 days with conservative care; return earlier for fever, spreading swelling, severe pain, or worsening mouth-opening. Spandidos Publications MDPI
• If you want, tell me the interincisal opening in mm, presence/absence of fever or pus, and whether the patient is tolerating fluids — I'll give a tailored next-step plan.

14. In a 55-year-old patient with a history of four doses of intravenous bisphosphonate (zoledronic acid) use, an unhealed area of exposed bone and pain develop weeks after extraction of mandibular 38. Which complication is most likely, and how should management be planned?

• Most likely diagnosis: Medication-Related Osteonecrosis of the Jaw (MRONJ) after IV zoledronic acid; management should be staged (confirm with imaging, conservative local care, antibiotics and pain control for symptomatic disease, and timely referral to an oral/maxillofacial surgeon for possible debridement or resection if disease progresses), coordinated with the patient's prescribing physician. AAOMS British Journal of Oral and Maxillofacial Surgery
• Key facts and immediate actions
• Likely complication: *MRONJ* presenting as exposed, non-healing mandibular bone with pain weeks after extraction in a patient who received intravenous zoledronic acid. IV bisphosphonates carry higher MRONJ risk than oral agents. AAOMS
• Immediate steps: stop local trauma, maintain oral hygiene, start chlorhexidine 0.12% rinse twice daily, control pain, and arrange urgent imaging and specialist referral. AAOMS
• Diagnostic workup (first 48–72 hours)
• Clinical exam: document size/location of exposed bone, presence of purulence, fistula, paresthesia, and systemic signs.
• Imaging: panoramic radiograph and CBCT to assess extent of necrosis, sequestra, and cortical involvement. AAOMS
• Medical review: confirm bisphosphonate dosing, indication (oncologic vs osteoporosis), other antiresorptives or antiangiogenics, comorbidities (diabetes, steroids), and current oncologic plan. Coordinate with the prescribing physician. AAOMS
• Staging and management summary (evidence-based)
Stage / Presentation
Typical findings Recommended management
No exposed
At risk / Stage 0

bone; nonspecific
Preventive care, monitor. AAOMS
symptoms
Stage 1 Exposed bone, no infection or Conservative: chlorhexidine rinse, observation, patient education.
pain AAOMS
Antibiotics (culture-guided; empiric amoxicillin-clavulanate or
clindamycin if penicillin allergy), oral antimicrobial rinses, local
debridement as needed; analgesia. Refer to OMFS. AAOMS
British Journal of Oral and Maxillofacial Surgery
Exposed bone with pain and
Stage 2
infection
Extensive
necrosis, pathologic
Surgical resection plus antibiotics and reconstruction as indicated.
Urgent OMFS care. AAOMS
Stage 3
fracture, extraoral
fistula

- Specific management plan for this patient (practical steps)
- Urgent referral to an oral and maxillofacial surgeon (within days). Document IV zoledronate use and dates. AAOMS
- Start conservative therapy now: chlorhexidine 0.12% rinse twice daily; gentle local debridement only if loose sequestra; analgesics as needed. AAOMS
- If signs of infection or systemic involvement: begin empiric antibiotics (e.g., amoxicillin-clavulanate), adjust per culture. British Journal of Oral and Maxillofacial Surgery
- Coordinate with the patient's physician/oncologist about the possibility and risks/benefits of interrupting bisphosphonate therapy—this is individualized and controversial because bisphosphonates persist in bone; decisions must be multidisciplinary. AAOMS
- Plan definitive care after staging: limited debridement for small symptomatic lesions; more extensive surgical resection for progressive or stage 3 disease. AAOMS British Journal of Oral and Maxillofacial Surgery
-
- Risks, trade-offs, and patient counseling
- Important: IV bisphosphonate–related MRONJ can be persistent and may require repeated interventions; early specialist involvement improves outcomes. AAOMS
- Drug holiday is not uniformly effective because bisphosphonates bind bone long-term; discuss with prescribing physician. AAOMS
- If you'd like, I can draft a concise referral note (history, meds, exam, suggested urgent actions) you can give to the oral surgery team in Ankara.

15. In a 55-year-old patient with a history of radiotherapy, an unhealed area of exposed bone and pain develop weeks after extraction of tooth 38. Which complication is this associated with, and how should it be managed?

This presentation is most consistent with osteoradionecrosis (ORN) of the mandible after tooth extraction in a previously irradiated field; urgent referral to an oral and maxillofacial surgeon (and the patient's radiation/oncology team) is required — initial management is conservative (local care, antibiotics if infected, analgesia, chlorhexidine) while definitive therapy ranges from medical therapy (PENTO) and hyperbaric oxygen in selected cases to surgical sequestrectomy or segmental resection with vascularized reconstruction for advanced disease. Frontiers MDPI

Key facts and immediate actions

- Diagnosis: Exposed necrotic bone in a previously irradiated jaw after dental extraction = osteoradionecrosis (ORN). Typical timing can be weeks to years after radiation; onset weeks after extraction is classic. Confirm with clinical exam and imaging (panoramic radiograph, CT). Frontiers
- Urgency: Refer urgently to oral/maxillofacial surgery and coordinate with radiation oncology. Early specialist input improves outcomes. MDPI

Initial (conservative) management — do this first

- Oral hygiene and local wound care: gentle debridement of loose bone, saline rinses, 0.12%–0.2% chlorhexidine mouthwash. Frontiers
- Treat infection and pain: systemic antibiotics targeted to clinical infection (empiric choices

per local protocols), analgesics, and topical measures. Obtain cultures if purulent drainage.
Frontiers
• Imaging and staging: panoramic X-ray and CT to define extent; staging guides therapy. MDPI
Adjunctive and disease-modifying options
• PENTO protocol (pentoxifylline + tocopherol ± clodronate): increasingly used for ORN with evidence of benefit in many series and trials; consider in consultation with specialists.
ijoms.com BMJ Open
• Hyperbaric oxygen (HBO): historically used for prevention/management; evidence is mixed and use is individualized based on severity, availability, and multidisciplinary decision.
Frontiers
Surgical management (when indicated)
• Sequestrectomy / conservative resection for localized necrotic bone not responding to conservative care.
• Segmental mandibulectomy with vascularized free-tissue reconstruction for extensive ORN, pathological fracture, or persistent infection — definitive and often necessary for advanced cases. Coordinate reconstruction with experienced head & neck/maxillofacial teams.
MDPI Frontiers
Prevention and counseling
• Pre-radiation dental assessment and extraction of non-restorable teeth before radiotherapy reduces ORN risk. After radiotherapy, avoid extractions if possible; if necessary, plan with oncology and surgical teams. MDPI
Risks, limitations, and next steps
• Risks: progression to pathologic fracture, chronic infection, fistula, need for major resection.
• Limitations: HBO availability and variable evidence; PENTO promising but requires specialist oversight.
• Next steps for this patient: urgent specialist referral, imaging, start local care and antibiotics if infected, discuss PENTO/HBO options, and plan surgical intervention if no improvement.
Frontiers ijoms.com
If you want, I can summarize a short referral checklist (imaging to order, wording for the referral, and suggested empiric antibiotics based on local guidelines).

16. In a 62-year-old patient taking warfarin, a mandibular right 48 extraction is performed without checking INR, followed by uncontrolled bleeding. What mechanism explains this bleeding, and what treatment should be applied?

• Mechanism
• Vitamin K antagonism causing deficient clotting factors.
• Warfarin inhibits vitamin K epoxide reductase, preventing gamma-carboxylation of vitamin K–dependent clotting factors II, VII, IX, and X (and proteins C and S). This reduces thrombin generation and fibrin formation, prolongs the prothrombin time/INR, and impairs primary hemostasis at the extraction site, producing uncontrolled bleeding.
• Immediate management and definitive treatment
• 1. Local hemostasis now
• Apply firm local pressure with gauze; place sutures if needed.
• Use topical hemostatic agents (oxidized cellulose, gelatin sponge), electrocautery, and a tranexamic acid mouthwash or soaked gauze (antifibrinolytic) to stabilize the clot.
• 2. Stop anticoagulant and assess
• Hold warfarin immediately and obtain an urgent INR.
• Assess hemodynamic status and estimate blood loss; give IV fluids and transfuse blood if indicated.
• 3. Reverse anticoagulation based on severity
• Major or life-threatening bleeding: give prothrombin complex concentrate (PCC) for rapid INR correction plus IV vitamin K (phytonadione) to sustain reversal. PCC is preferred because it corrects INR quickly.
• Significant but not life-threatening bleeding: give IV or slow-infusion vitamin K (commonly 5–10 mg IV) and consider FFP if PCC unavailable or if rapid correction is required.
• Minor bleeding with mildly elevated INR: local measures plus oral vitamin K and close INR monitoring may suffice.
• 4. Follow up
• Recheck INR after reversal and before restarting warfarin; resume anticoagulation only after bleeding is controlled and with appropriate bridging if needed.
• Document the event and coordinate with the patient's physician or anticoagulation clinic for long-term management.

17. In a 30-year-old patient, after mandibular 48 extraction, painful oral erosions and cutaneous target lesions develop following use of ibuprofen and amoxicillin. Which drug reaction does this suggest, and how should it be treated?

• Likely diagnosis: Stevens-Johnson syndrome (SJS) triggered by recent amoxicillin and/or ibuprofen; immediate management is to stop the offending drugs and admit for urgent supportive care (often in a burn/ICU setting) with specialist input (dermatology, ophthalmology), plus consideration of systemic immunomodulatory therapy such as IVIG or cyclosporine in selected cases. Stevens-Johnson Syndrome Foundation Mayo Clinic

• Diagnosis — why SJS fits

• Key features: acute painful oral mucosal erosions plus cutaneous targetoid lesions after new medications are classic for SJS (a severe mucocutaneous drug reaction). Antibiotics (including penicillins) and NSAIDs are well-recognized triggers. Stevens-Johnson Syndrome Foundation

• Spectrum: SJS and toxic epidermal necrolysis (TEN) are a continuum defined by body-surface area (BSA) of epidermal detachment: SJS <10% BSA; SJS/TEN overlap 10–30%; TEN >30%. Severity and prognosis correlate with BSA and comorbidities. MDPI

• Immediate management (first 24 hours)

• Stop all suspected drugs immediately (amoxicillin, ibuprofen, and any other recent new meds). Mayo Clinic

• Urgent hospital admission — ideally to a burn unit, intensive care unit, or high-dependency dermatology ward for fluid balance, wound care, and monitoring. Mayo Clinic

• Supportive care (cornerstone of treatment):

o Fluid and electrolyte management; monitor renal function and electrolytes.

o Wound care as for partial-thickness burns (nonadherent dressings, sterile technique).

o Pain control and nutritional support (enteral feeding if oral intake impossible).

o Infection surveillance and early treatment of sepsis if it occurs. Mayo Clinic

• Specialist consultations: dermatology, ophthalmology (urgent), ENT/dental for mucosal care, and critical care as needed. Ocular complications can be sight-threatening. Mayo Clinic

• Systemic therapies — evidence and options

• Systemic corticosteroids: used in some centers early in disease but evidence is mixed and risks (infection) must be weighed. MDPI

• Intravenous immunoglobulin (IVIG): widely used historically; randomized evidence is limited and results variable. Consider in severe cases, especially early, but benefit is not definitive. Frontiers JAMA Network

• Cyclosporine: emerging evidence suggests cyclosporine may reduce mortality and halt progression in some patients and is considered a reasonable option in selected severe cases. JAMA Network MDPI

• Practical points and prognosis

• Immediate drug cessation and supportive care are the most important actions and improve outcomes. Mayo Clinic

• Mortality risk varies with extent of skin loss, age, and comorbidities; early transfer to an appropriate unit and multidisciplinary care reduce complications. MDPI JAMA Network

• Actionable summary: Stop amoxicillin and ibuprofen now, arrange urgent hospital admission (burn/ICU or dermatology ward), start supportive/burn-style care, obtain dermatology and ophthalmology review, and discuss systemic immunomodulation (IVIG or cyclosporine) with specialists. Mayo Clinic JAMA Network

18. In a 27-year-old patient, during surgical extraction of an impacted tooth 28, a portion of the maxillary tuberosity fractures and separates along with the tooth. What surgical and postoperative management should be performed?

• Management of a fractured maxillary tuberosity during extraction

• Immediate surgical assessment

• Assess size, vascularity and mobility of the fractured tuberosity fragment and check for communication with the maxillary sinus.

• If the fragment is large, viable and well vascularized: attempt to preserve it — reposition and stabilize rather than remove.

• If the fragment is small, comminuted, non-viable, or prevents adequate closure: remove the loose bone, smooth sharp edges (alveoloplasty) and prepare for primary closure.

• Surgical techniques
• Reposition and stabilize (preferred when feasible):
o Gently reposition the tuberosity fragment into its anatomic position.
o Stabilize with tension-free suturing (interrupted or figure-of-eight) using 3-0 or 4-0 resorbable sutures to approximate mucoperiosteal flaps and hold the fragment in place.
o If interdental support is needed and fragment is large, consider temporary fixation (e.g., interdental wiring or splinting) or referral to an oral and maxillofacial surgeon.
• If an oroantral communication (OAC) is present or sinus floor is involved:
o Small OAC (<5 mm): attempt primary closure with a tension-free mucoperiosteal flap (buccal advancement flap) and layered closure.
o Larger OAC or unstable fragment: consider buccal fat pad flap or palatal rotational flap, or refer for specialist closure.
o Avoid forcing bone fragments into the sinus; remove nonviable bone and close the defect.
• If fragment must be removed: smooth bone margins, achieve primary soft-tissue closure.
• Postoperative management
• Medications
o Antibiotics: broad-spectrum cover (e.g., amoxicillin-clavulanate) for several days; if penicillin allergic, use clindamycin.
o Analgesia: NSAID ± acetaminophen as appropriate.
o Short course nasal decongestant and/or topical steroid may be advised if sinus involvement is suspected (follow local protocols).
• Local care and instructions
o Sinus precautions: no nose blowing, avoid sneezing with mouth closed, avoid sucking through straws, no smoking, avoid vigorous rinsing for 24 hours.
o Oral hygiene: gentle rinsing with warm saline or chlorhexidine after 24 hours; maintain soft diet.
o Ice packs first 24 hours to reduce swelling; then warm compresses if needed.
• Activity and diet: soft diet, avoid heavy exertion for several days.
• Follow-up and complications to watch for
• Early review within 3–7 days to check wound stability, suture integrity and signs of infection or persistent OAC.
• Radiographic follow-up if sinus involvement suspected or to confirm healing.
• If persistent OAC, chronic sinusitis, wound dehiscence, or nonunion: refer to oral and maxillofacial surgery or ENT for definitive management (flap revision, sinus surgery, fixation).
• Document the event and discuss prosthetic implications (tuberosity loss may affect future denture support).
• Key principle (one line)
• Preserve and stabilize a viable tuberosity fragment and achieve a tension-free primary closure; remove and smooth nonviable bone and close the defect, then institute sinus precautions, antibiotics, analgesia and close follow-up.

19. In a 29-year-old patient, several weeks after extraction of tooth 18, unilateral nasal obstruction, foul-smelling yellow-green discharge, and facial pain develop. Which complication does this suggest, and how should it be managed?

• Likely diagnosis: an oroantral communication progressing to odontogenic maxillary sinusitis (oroantral fistula if persistent). Immediate management is medical (sinus care and antibiotics) and, if the communication persists beyond ~2–3 weeks or is larger than ~5 mm, surgical closure with sinus clearance and a mucosal flap is indicated. Springer MDPI
• Key considerations and initial assessment
• Probable complication: tooth 18 (maxillary third molar) extraction followed by unilateral purulent nasal discharge, foul smell, nasal obstruction and facial pain strongly suggests an oroantral communication (OAC) with secondary maxillary sinusitis or an established oroantral fistula (OAF) if chronic. MDPI
• Urgency: treat infection and close the communication to prevent chronic sinus disease, foreign-body contamination, and persistent drainage. Springer IntechOpen
• Immediate management (first 48–72 hours)
• Medical therapy: start broad-spectrum antibiotics targeting oral flora (commonly amoxicillin–clavulanate or equivalent), analgesics, and nasal decongestants; advise

saline nasal irrigation to clear purulence. MDPI
• Local measures: instruct the patient to avoid nose-blowing, sneezing with closed mouth, and sucking through a straw; soft diet and good oral hygiene.
• ENT/dental coordination: arrange prompt evaluation by an oral surgeon and/or ENT specialist for endoscopic assessment and imaging. Springer
• Diagnostic workup
• Clinical tests: Valsalva test (air escape through socket), inspection of extraction site for visible communication.
• Imaging: panoramic radiograph or periapical films initially; CBCT or CT sinus if sinusitis suspected or to plan surgery. Nasal endoscopy helps assess sinus mucosa and purulence. MDPI Springer
• Definitive management — when and how to close
• Conservative healing: small OACs <5 mm may close spontaneously with conservative care if sinus is healthy. Monitor closely for 1–2 weeks. IntechOpen
• Indications for surgical closure: defect >5 mm, persistent drainage beyond 2–3 weeks, recurrent/persistent sinusitis, or visible epithelialized tract (fistula). Springer IntechOpen
• Surgical options: buccal advancement flap, palatal rotational flap, or buccal fat pad flap are commonly used to close the oral side; choice depends on defect size, location, and dentition. Before closure, sinus must be cleared of infection—either by intraoral irrigation, endoscopic sinus surgery (FESS) if chronic sinus disease is present, or combined approaches. Springer MDPI
• Risks, follow-up, and patient counseling
• Risks if untreated: chronic sinusitis, recurrent infections, impaired healing, need for more extensive sinus surgery.
• Follow-up: reassess within 48–72 hours after antibiotics start; if symptoms persist or imaging shows sinus disease, proceed to ENT for FESS ± surgical closure. Document and photograph the socket and communicate plan between dentist and ENT. MDPI Springer
• If you want, I can outline a step-by-step surgical closure plan (flap choice, suturing technique, postoperative regimen) tailored to the defect size and whether the patient is dentate or edentulous.

20. In a 24-year-old patient, during difficult extraction of a deeply impacted 48, the tooth slips from the elevator and is lost intraorally, followed by coughing and a foreign-body sensation in the throat. What condition does this most strongly suggest, and how should it be managed?

• Likely diagnosis
• Immediate management (first minutes)
• Keep the patient upright and encourage effective coughing if the patient is able; do not perform blind finger sweeps.
• Call for urgent help (oral/maxillofacial surgeon, anesthesiology, ENT/pulmonology, and theatre staff).
• Obtain a chest and neck radiograph (AP and lateral) immediately to localize a radiopaque tooth. If radiographs are inconclusive but suspicion remains high, proceed to endoscopic evaluation. British Orthodontic Society OAText
• Definitive management
• If the tooth is lodged high in the oropharynx or hypopharynx and visible, it may be removed under direct vision; otherwise do not attempt blind retrieval. British Orthodontic Society
• Practical steps to arrange care (context for Ankara / local setting)
• Avoid blind attempts that may push the tooth deeper or cause aspiration into a main bronchus.

21. In a 27-year-old patient, during an inferior alveolar nerve block for mandibular right 48, the needle fractures and is lost within the pterygomandibular space. By what mechanism can this occur, and how should it be treated?

• A fractured inferior alveolar block needle most commonly occurs when a thin/short needle is bent or engages soft tissue and the patient moves or the operator withdraws against resistance; immediate management is localization with imaging and prompt referral to an oral and maxillofacial surgeon for retrieval (early surgical removal is preferred), although in rare, deeply embedded asymptomatic cases conservative observation may be considered after informed consent. If you are in Ankara, arrange urgent referral to a local oral and maxillofacial surgery service for imaging and removal. Nature Annex Publishers

• Mechanism (how fracture/loss occurs)
• Mechanical stress at a weakened point: Needles can fracture where they were previously bent, at the hub, or where a manufacturing defect exists. Repeated bending or using very short, thin needles increases risk. Nature MDPI
• Patient movement during injection: Sudden head movement while the needle is engaged in the pterygomandibular tissues can produce shear forces that snap the needle. Nature
• Improper technique: Inserting the needle to the hub, changing direction while embedded, or attempting to withdraw a bent needle can cause breakage. Reuse of needles or using inappropriate gauge/length contributes. MDPI
• Immediate steps at the chairside (first actions)
• Stop further manipulation — do not probe blindly or attempt blind retrieval. Nature
• Keep the patient calm and immobilize the head to prevent further migration. Nature
• Document the event (time, needle type/gauge/length, depth of insertion, patient movement) and inform the patient clearly. Annex Publishers
• Diagnostic workup (localize the fragment)
• Plain radiographs (periapical, panoramic) can often show metallic fragments.
• CBCT or CT is preferred for precise 3-D localization in the pterygomandibular space and surgical planning. Do not delay imaging if retrieval is planned. Annex Publishers MDPI
• Definitive management options
• Early surgical retrieval (recommended): Referral to an oral and maxillofacial surgeon for intraoral approach under local or general anesthesia is standard; early removal reduces risk of migration, infection, and fibrosis. Nature Annex Publishers
• Image-guided/minimally invasive retrieval: Use of CBCT guidance, fluoroscopy, or surgical navigation when available improves accuracy. MDPI
• Conservative management (rare): If the fragment is deeply embedded, asymptomatic, and retrieval risk is high, some teams may elect observation with informed consent and periodic imaging; this is a case-by-case decision. MDPI Annex Publishers
• Additional considerations and prevention
• Do not attempt blind probing—this risks further migration and neurovascular injury. Nature
• Tetanus status and antibiotics: Assess tetanus immunization; antibiotics are considered case-by-case if contamination or infection risk exists. Annex Publishers
• Prevention: Use appropriate needle gauge/length, avoid bending needles, never insert to the hub, use single-use needles, and instruct patients to remain still. Nature MDPI
• Practical recommendation for you now
• Obtain immediate radiographic localization (panoramic + CBCT if available) and refer urgently to an oral and maxillofacial surgeon in Ankara for planned removal. Document and counsel the patient about risks, timing, and alternatives. Annex Publishers Nature
• If you want, I can outline a step-by-step surgical approach (intraoral vs extraoral), list imaging parameters for CBCT, or draft patient counseling language and documentation notes.

22. In a 19-year-old patient, during impacted 48 surgery, cyanosis, fatigue, and low oxygen saturation develop after administration of prilocaine and articaine. What does this indicate, and how should it be treated?
• Likely diagnosis: acute methemoglobinemia from local anesthetic (prilocaine); treat immediately with high-flow oxygen, stop the offending drugs, confirm with co-oximetry, and give intravenous methylene blue 1–2 mg/kg (repeat if needed) unless contraindicated. DergiPark Drugs.com
• Clinical interpretation
• What this indicates: Central cyanosis, fatigue, and a low pulse-ox reading shortly after prilocaine (and less commonly other amide anesthetics) strongly suggest methemoglobinemia — hemoglobin iron oxidized to Fe^{3+} (methemoglobin) which cannot carry oxygen. Pulse oximetry often reads ~85% and does not correct with supplemental O_2. ARC Publications DergiPark
• Immediate management steps
• Stop further local anesthetic exposure and remove any ongoing source. DergiPark
• Give high-flow oxygen and support airway/ventilation as needed. Austin Health
• Obtain arterial blood gas with co-oximetry (to measure methemoglobin fraction) and look for chocolate-brown blood on sampling. ARC Publications
• Administer methylene blue if methemoglobin is clinically significant or symptomatic (see dosing below). Drugs.com

• Specific treatment and dosing
• First-line antidote: Methylene blue IV 1–2 mg/kg given over 3–5 minutes (commonly 1 mg/kg initially). If symptoms or MetHb fraction persist, repeat 1 mg/kg after 30–60 minutes. Do not exceed a cumulative dose ~7 mg/kg because higher doses can paradoxically induce methemoglobinemia. Drugs.com Austin Health
• When to treat: Treat symptomatic patients or MetHb fraction >20–30% (lower thresholds if cardiac/pulmonary disease or infants). Austin Health
• Important precautions and alternatives
• Check for G6PD deficiency before or as soon as possible: methylene blue is relatively contraindicated in G6PD deficiency because it can cause hemolysis and be ineffective. If G6PD deficiency is present or methylene blue fails, consider exchange transfusion, hyperbaric oxygen, or high-dose ascorbic acid in consultation with a toxicologist/hematologist. Austin Health turkishjic.org
• Drug interactions: Methylene blue has MAOI activity and can precipitate serotonin syndrome in patients on serotonergic drugs; weigh risks and consult toxicology if relevant. Austin Health
• Monitoring and follow-up
• Continuous pulse oximetry and cardiac monitoring.
• Repeat co-oximetry to document falling MetHb levels after treatment.
• Observe for rebound methemoglobinemia (especially with long-acting oxidant exposure) and for adverse effects of methylene blue. Drugs.com Austin Health
• If you want, I can summarize a concise emergency checklist you can print or paste in the operating room.

23. In a 45-year-old patient with uncontrolled diabetes, increasing pain, fistula formation, foul odor, and sequestrum formation develop a few weeks after extraction of mandibular left 37 and 38. What condition does this suggest, and how should it be treated?

• Likely diagnosis
• Chronic suppurative osteomyelitis of the mandible (post-extraction osteomyelitis with sequestrum formation).
• Key supporting features
• Occurred weeks after extraction
• Sequestrum formation (dead bone)
• Persistent/increasing pain, foul odor, draining fistula
• Predisposing factor: uncontrolled diabetes (impaired host defense)
• Management
• Confirm diagnosis
o Panoramic radiograph and CT (CBCT) to define extent and locate sequestra.
o Microbiologic sampling of pus or tissue for culture and sensitivity.
• Definitive surgical treatment
o Surgical debridement: removal of all necrotic bone (sequestrectomy) and adequate curettage/decortication.
o Establish drainage and excise sinus tracts if present.
o Leave healthy bleeding bone margins.
• Antibiotic therapy
o Start empiric broad-spectrum antibiotics covering oral flora, then tailor to culture results.
o Typical empiric choices: IV ampicillin–sulbactam or IV ceftriaxone + metronidazole, or IV clindamycin if penicillin allergic.
o Continue prolonged therapy (often several weeks; IV then oral) guided by clinical response and labs.
• Medical optimization
o Strict glycemic control and management of other systemic risk factors.
o Nutritional support.
• Adjuncts when indicated
o Hyperbaric oxygen therapy for refractory cases or extensive osteoradionecrosis-like presentations.
o Regular follow-up with imaging and clinical review until resolution.
• Prognosis
• Good if necrotic bone is completely removed, infection is culture-directed, and diabetes is controlled; delayed or inadequate treatment risks persistent infection and pathologic fracture.

24. In a 23-year-old patient, after local anesthesia for tooth 28, it is learned that the patient has a history of drug allergy and atopic disease . Shortly after injection, generalized pruritus–urticaria, lip/face swelling, wheezing, and dizziness develop. How would you evaluate this presentation in terms of the most likely diagnosis, and how would you plan the emergency management in a dental clinic?

• Immediate conclusion: This presentation is most consistent with acute anaphylaxis triggered by a dental exposure (local anaesthetic or excipient) — characterized by generalized urticaria, angioedema, bronchospasm, and circulatory compromise. Treat immediately with intramuscular adrenaline (epinephrine) 0.3–0.5 mg (1:1,000) and call emergency medical services. Nature Springer

• 1. Diagnostic evaluation (most likely diagnosis and differential)

• Most likely diagnosis: Anaphylaxis — rapid onset multisystem allergic reaction with skin (pruritus/urticaria, lip/face swelling), respiratory (wheezing), and cardiovascular (dizziness → presyncope/hypotension) signs. Nature

• Key differential diagnoses (brief):

Local anaesthetic systemic toxicity
Feature Anaphylaxis Vasovagal
reaction
(LAST)
Urticaria, generalized
Skin
Pallor, sweating Usually no urticaria
pruritus, angioedema
Respiratory Wheeze, stridor,
bronchospasm Rare Possible respiratory depression if
severe
Hypotension, tachycardia or
Hypertension/tachyarrhythmia then
Bradycardia,
Cardiovascular
seizures, then cardiovascular
hypotension
collapse
collapse
During/after injection, often
Minutes after intravascular injection
Timing Minutes after
exposure
or overdose
with prodrome

• (Comparison synthesized from dental/anaphylaxis reviews.) Nature anesthesiaprogress.kglmeridian.com

• 2. Immediate emergency management in the dental clinic (step-by-step)

• A. Recognize and call for help

• Call emergency services immediately and summon trained staff. Nature

• B. First-line treatment — adrenaline (epinephrine)

• Give intramuscular (IM) adrenaline 0.3–0.5 mg of 1:1,000 (1 mg/mL) into the mid-outer thigh immediately. Repeat every 5 minutes if there is no improvement. This is the single most life-saving action. Nature

• C. Positioning and airway

• Place patient supine with legs elevated unless vomiting or severe respiratory distress; maintain airway and prepare for advanced airway if swelling progresses. Administer high-flow oxygen. Springer Nature

• D. Circulation and monitoring

• Establish IV access, give IV fluids (crystalloid bolus) for hypotension, monitor pulse, BP, SpO_2, and ECG. Prepare for advanced resuscitation if needed. Springer

• E. Adjunctive therapies (after adrenaline)

• H1 antihistamine (e.g., chlorphenamine/clemastine IV/IM) and corticosteroid (e.g., hydrocortisone IV) as adjuncts to reduce prolonged or biphasic reactions (they do not replace adrenaline). For bronchospasm, give nebulized salbutamol. Nature

• F. If severe airway compromise or cardiac arrest

• Follow advanced life support algorithms; be ready for endotracheal intubation or

cricothyrotomy if needed. Springer
• 3. After-stabilization and follow-up
• Observe in hospital (often 4–24 hours depending on severity) for biphasic reactions. Nature
• Document event, suspected trigger, and medications given.
• Refer to an allergist/immunologist for confirmatory testing (skin tests or specific IgE) and consideration of an epinephrine auto-injector prescription and written action plan. anesthesiaprogress.kglmeridian.com
• 4. Practical clinic preparedness (brief checklist)
• Stock: adrenaline ampoules (1 mg/mL), syringes/needles, oxygen, IV fluids, antihistamines, steroids, bronchodilator nebuliser. Nature
• Train staff in recognition and IM adrenaline administration and run regular emergency drills. Nature
• If you want, I can convert this into a one-page clinic emergency checklist (no file export) or a short script for staff roles during anaphylaxis.

25. A 29-year-old patient presents two weeks after extraction of tooth 18 with the complaint that liquids consumed come out through the nose. CBCT shows an oroantral communication measuring 6 mm. What complication does this represent, and how should it be treated?

Clinical Interpretation :Complication: The patient presents with an oroantral fistula (OAF), which is a pathological epithelialized communication between the oral cavity and the maxillary sinus. It has developed following extraction of the maxillary third molar (tooth 18). The clinical symptom of fluids passing into the nasal cavity is characteristic of this complication.
Pathophysiology: Oroantral communications larger than 5 mm, if left untreated, often fail to close spontaneously and epithelialize over time, forming a chronic fistula. This allows persistent passage of fluids and bacteria, leading to recurrent sinusitis.
Diagnosis: Confirmed by clinical history (fluid leakage through the nose) and radiographic imaging (CBCT showing a 6 mm defect). The size is significant enough to require surgical closure.
Management Principles (Based on Standard Oral Surgery Textbooks):
Initial Considerations:
Eliminate any sinus infection before closure (antibiotics, nasal decongestants, sinus irrigation if indicated).
Ensure the sinus is free of purulent discharge and obstruction.
Surgical Closure Options:
Buccal advancement flap (Rehrmann flap): Most commonly used technique; a trapezoidal mucoperiosteal flap is advanced to cover the defect.
Palatal rotational flap: Useful for posterior defects; provides robust tissue with good blood supply.
Buccal fat pad flap: Particularly effective for larger defects (>5 mm) and posterior maxilla; provides well-vascularized tissue.
Combination techniques: In complex or recurrent cases, buccal fat pad with mucosal advancement may be employed.
Postoperative Care:
Antibiotics (covering oral flora, e.g., amoxicillin-clavulanate).
Nasal decongestants to reduce sinus pressure.
Avoidance of nose blowing, sneezing with mouth closed, or using straws.
Soft diet and meticulous oral hygiene.
Summary: This case represents an oroantral fistula secondary to extraction of tooth 18. Given the 6 mm size and persistence beyond two weeks, spontaneous closure is unlikely. The recommended treatment is surgical closure using a local flap technique (most commonly buccal advancement flap or buccal fat pad flap), preceded by management of any sinus infection.

26. In a 52-year-old patient with uncontrolled diabetes, five days after bilateral mandibular 38 and 48 extractions, tongue swelling, dyspnea, and “board-like” induration/swelling of the neck develop. Which life-threatening infection does this suggest, and what is the appropriate emergency management?

• Ludwig’s angina is the most likely diagnosis — a rapidly spreading cellulitis of the floor of mouth that causes tongue elevation and life-threatening airway compromise; immediate airway protection (early definitive airway or emergency tracheostomy/cricothyrotomy if needed), broad-spectrum IV antibiotics, urgent surgical drainage when indicated, and ICU admission with glycemic control are required. Cleveland Clinic CHEST

• Key diagnosis
• Likely infection: Ludwig's angina — a rapidly progressive, bilateral cellulitis of the submandibular, sublingual and submental spaces classically following lower molar extractions, presenting with tongue swelling, dysphagia, dyspnea, drooling, and "board-like" neck induration. This is a true airway emergency. Cleveland Clinic IOSR Journals
• Immediate emergency management (stepwise)
• 1. Secure the airway without delay
• Priority: protect the airway early — do not wait for complete obstruction.
• Preferred: awake fiberoptic naso/orotracheal intubation performed by an experienced anesthesiologist/ENT under topical anesthesia and sedation if anatomy and cooperation permit. IOSR Journals CHEST
• If intubation fails or is impossible: perform emergency surgical airway (tracheostomy or cricothyrotomy) immediately. Prepare for difficult airway equipment and ENT backup. CHEST Cleveland Clinic
• 2. Start empiric broad-spectrum IV antibiotics immediately
• Empiric regimen examples: IV ampicillin-sulbactam or IV penicillin plus metronidazole, or IV clindamycin (for penicillin allergy) to cover oral anaerobes and streptococci/staphylococci. Adjust based on cultures. Start high-dose therapy. Cleveland Clinic IOSR Journals
• 3. Surgical evaluation and drainage
• Assess for fluctuant collections with bedside exam and imaging; urgent surgical drainage of involved fascial spaces if abscess or loculated infection is present. Early ENT/oral-maxillofacial surgical consultation is mandatory. IOSR Journals
• 4. Supportive care and monitoring
• Admit to ICU for airway monitoring, hemodynamic support, and serial exams.
• Obtain CT neck with contrast if patient is stable to define extent and guide drainage. Cleveland Clinic
• 5. Treat underlying risk factors
• Aggressive glycemic control for uncontrolled diabetes to improve infection control and outcomes. Consider blood glucose management and endocrinology input. Cleveland Clinic
• Why this is urgent
• Ludwig's angina can progress rapidly to complete airway obstruction, mediastinitis, sepsis, and death; early airway control and combined medical-surgical therapy reduce mortality. Cleveland Clinic CHEST
• Practical checklist for the team now
• Call ENT/anesthesia/ICU immediately.
• Prepare difficult airway cart and plan A/B/C.
• Start IV broad-spectrum antibiotics now.
• Arrange urgent imaging and surgical consultation.
• Control blood glucose and admit to ICU. Cleveland Clinic IOSR Journals
• If you want, I can summarize this as a one-page emergency checklist you can print for the team.

27. In a 22-year-old patient, after an inferior alveolar nerve (IAN) block administered for mandibular right 48, difficulty closing the eye and drooping of the corner of the mouth are observed. How can the cause of this finding be explained, and how should it be managed?

• Brief answer: This is most consistent with a transient facial nerve (VII) palsy caused by inadvertent deposition of local anaesthetic into/near the parotid region (styloid/parotid capsule) during the IAN block; it is usually immediate and self-limiting (hours) — protect the eye, reassure the patient, observe, and refer if signs persist beyond 24–72 hours. MDPI IJRASET
• Explanation of cause
• Mechanism: If the needle is advanced too far posteriorly or superiorly during an inferior alveolar nerve block, the tip can enter the parotid region and deposit anaesthetic around the facial nerve branches (extracranial facial nerve at the stylomastoid/parotid area). This produces acute, unilateral facial weakness (inability to close the eye, drooping of mouth) that mimics Bell palsy but has immediate onset after injection. Onset is usually immediate and correlates with the anaesthetic taking effect. MDPI IJRASET
• Other possible but less likely causes: intravascular injection with spread to facial nerve, direct needle trauma to a peripheral branch, or local haematoma compressing the nerve.

These are rarer than simple local deposition. Anesthesia Experts
• How to manage (practical steps)
• Immediate (in clinic):
• Reassure the patient that this is commonly transient and related to the block technique. MDPI
• Protect the cornea: if the patient cannot fully close the eye, apply lubricating eye drops or ointment and cover the eye with a patch or protective shield to prevent exposure keratitis until eyelid function returns. This is the single most important immediate action. MDPI
• Document the event (time of onset, signs, block technique, anaesthetic used, volume, aspiration result).
• Advise rest and observation in the clinic for a short period (30–60 minutes) to confirm stability.
• Follow-up and escalation:
• Expect spontaneous recovery as the local anaesthetic wears off — typically within a few hours (duration depends on agent and dose). MDPI
• If weakness persists beyond 24 hours or if symptoms worsen (increasing pain, sensory loss, systemic signs), arrange urgent review by oral/maxillofacial surgery, neurology, or ENT and consider imaging or specialist nerve assessment. If corneal protection remains needed, refer to ophthalmology. MDPI IJRASET
• If there is any sign of systemic local anaesthetic toxicity (CNS or cardiovascular symptoms), treat per advanced life support/local anaesthetic systemic toxicity protocols and transfer to emergency care. Anesthesia Experts
• Prevention (for future IAN blocks)
• Avoid over-advancing the needle posteriorly; know anatomical landmarks and use measured depth.
• Always aspirate before injection and inject slowly with minimal effective volume.
• Consider alternative techniques (e.g., modified IAN approaches) if anatomy or prior complications suggest risk. IJRASET
• When to seek urgent care (Ankara context)
• If facial weakness persists >24–48 hours, or if eye symptoms (pain, redness, vision change) occur, go to the nearest emergency department or see an ophthalmologist/ENT/neurology clinic in Ankara for assessment and corneal protection. If you want, I can list nearby hospitals or ophthalmology clinics in Ankara and their contact details.
• Key takeaways: Immediate facial weakness after IAN block is most often a transient facial nerve palsy from anaesthetic spread to the parotid/facial nerve; protect the eye, observe, document, and refer if it does not resolve within hours to a couple of days. MDPI IJRASET

28. In a 27-year-old patient, within a few days after a difficult 28 extraction, temporomandibular joint clicking, locking, and pain develop. By what mechanism can this be explained, and how should it be managed?

• Acute TMJ symptoms after a difficult lower third-molar (tooth 28) extraction are most commonly caused by prolonged mouth opening and direct joint/muscle trauma leading to disc displacement, capsular inflammation, and muscle spasm; initial treatment is conservative (jaw rest, analgesics/NSAIDs, soft diet, local heat/ice, guided jaw exercises and short-term muscle relaxants), with referral for imaging and specialist care if locking or pain persists beyond 2–4 weeks or if there is true mechanical blockage. pmc.ncbi.nlm.nih.gov Taylor & Francis Online
• Mechanism
• Prolonged maximal mouth opening and forceful manipulation during a difficult extraction strains the lateral pterygoid, masseter and temporomandibular joint (TMJ) capsule, which can produce anterior disc displacement, capsulitis, synovitis, or muscle spasm. This explains clicking (disc recapture), intermittent locking (partial displacement), or fixed locking (mechanical entrapment) and pain. pmc.ncbi.nlm.nih.gov mdpub.net
• Local inflammation or hematoma in the joint or masticatory muscles after surgery amplifies pain and limits movement. pmc.ncbi.nlm.nih.gov
• Initial management (first 48–72 hours)
• Rest the jaw: avoid wide opening (no yawning, large bites). Soft diet.
• Analgesia and anti-inflammatory therapy: NSAIDs as first line (unless contraindicated); consider short course of acetaminophen or combined therapy. pmc.ncbi.nlm.nih.gov

• Local measures: alternating warm compresses to relax muscles and cold packs for acute swelling/pain.
• Short course of muscle relaxant if severe trismus or muscle spasm (prescribed by clinician).
• Gentle, guided jaw exercises started after acute pain improves to prevent stiffness and promote disc mobility (open-close in a controlled manner, lateral excursions as instructed). pmc.ncbi.nlm.nih.gov• When to escalate or refer
• Urgent referral to an oral and maxillofacial surgeon or TMJ specialist if: true inability to close or open the mouth (locked open/closed), worsening neurologic signs, fever, spreading infection, or severe uncontrolled pain. Taylor & Francis Online
• Imaging: if symptoms persist >2–4 weeks or mechanical locking recurs, obtain MRI to assess disc position and soft tissues; CT if bony injury suspected. pmc.ncbi.nlm.nih.gov
• Interventions for persistent cases: supervised physical therapy, occlusal splint/nightguard, intra-articular corticosteroid injection or arthrocentesis for refractory synovitis, and surgical options only after conservative measures fail. pmc.ncbi.nlm.nih.gov Taylor & Francis Online
• Practical points and risks
• Most post-extraction TMJ problems are self-limited and respond to conservative care within 2–4 weeks. mdpub.net
• Avoid aggressive manipulation of a locked jaw at home; seek professional reduction to prevent worsening disc injury.
• If you are in Ankara, seek evaluation at a local dental/oral-maxillofacial clinic or hospital emergency department if locking is complete, pain is severe, or oral intake is compromised.
• If you'd like, I can outline a simple 7-day home care plan and exercise set tailored to your current pain and opening range.

29. In a 28-year-old patient, during mandibular left 38 surgery, prolonged use of a bur with inadequate water cooling results in a localized area of necrotic bone. Which complication is this consistent with, and how should it be managed?

• Diagnosis
• Thermal osteonecrosis (heat-induced bone necrosis) of the mandibular socket from bur overheating, which may progress to sequestration and secondary infection (osteomyelitis) if not managed.
• Key clinical features to expect
• Localized exposed, non-bleeding bone in the surgical site
• Delayed healing and persistent pain beyond the normal postoperative course
• Possible foul odor, purulent discharge, or loosening of a bone fragment if infection/sequestrum develops
• Immediate management
• Assess extent and infection signs — examine for exposed necrotic bone, pus, systemic signs.
• Conservative debridement/sequestrectomy — remove loose necrotic bone under local anesthesia; irrigate copiously with saline.
• Control infection if present — prescribe antibiotics guided by clinical severity (e.g., amoxicillin ± metronidazole or alternative if allergic) and give analgesics.
• Local care — chlorhexidine mouthwash, gentle irrigation, and regular follow-up to monitor healing and detect further sequestration.
• Histology/culture — consider sending removed bone for culture/histopathology if atypical course or concern for deeper infection.
• When to escalate
• Persistent or spreading infection, systemic signs, large sequestra, or failure to heal → refer to oral and maxillofacial surgery for formal surgical debridement, possible curettage, and further management (imaging and longer antibiotic therapy).
• Prevention (brief)
• Use adequate water cooling, intermittent cutting, sharp burs, and appropriate speed/pressure during bone cutting to avoid thermal injury.

30. In a 23-year-old patient, intraosseous anesthesia is administered during mandibular 38 extraction. Ten days later, the patient presents with an unhealed area of exposed bone at the extraction site and increasing pain. Which complication is this most likely to indicate, and how should management be planned?

• Most likely this represents localized osteonecrosis of the alveolar bone (anesthesia-related osteonecrosis/sequestration) rather than a routine dry socket; urgent

assessment and removal of necrotic bone with targeted infection control and referral to an oral/maxillofacial surgeon are indicated. Journal of Endodontics thecomfortdentistry.com

• Key diagnostic considerations
• Primary diagnosis: Osteonecrosis of the jaw (localized bone necrosis/sequestrum) after intraosseous injection is a recognized, though rare, complication and fits the timeline of persistent exposed bone at 10 days with worsening pain. Journal of Endodontics
• Important differentials: Alveolar osteitis (dry socket) usually presents earlier (48–72 hours) with exposed socket pain and foul odor; acute osteomyelitis or infected sequestrum is possible if systemic or local infection signs are present. thecomfortdentistry.com biologyinsights.com
• Immediate clinical assessment
• History: confirm anesthesia type (intraosseous), any systemic disease, smoking, medications (bisphosphonates, antiresorptives), and progression of symptoms.
• Examination: inspect size/location of exposed bone, presence of pus, soft-tissue inflammation, lymphadenopathy, and trismus.
• Imaging: obtain periapical radiograph and panoramic radiograph; consider CBCT to define extent of necrosis/sequestrum. Journal of Endodontics
• Management plan (stepwise)
• Conservative initial measures
o Chlorhexidine 0.12% mouth rinse twice daily to reduce surface contamination.
o Analgesia (NSAID ± paracetamol) and local wound care (gentle irrigation).
o Systemic antibiotics only if clinical infection (fever, purulence, cellulitis) or immunocompromise — choose based on local protocols (e.g., amoxicillin ± metronidazole) and culture if available. thecomfortdentistry.com biologyinsights.com
• Definitive surgical management
o If a mobile sequestrum is present or pain persists despite conservative care, plan surgical removal (sequestrectomy) and debridement under local or general anesthesia. This both relieves pain and promotes mucosal healing. Journal of Endodontics
o Send removed tissue for histopathology and microbiology to exclude other causes and guide antibiotics.
• Referral and follow-up
o Refer promptly to an oral and maxillofacial surgeon or tertiary dental clinic for operative management and imaging review.
o Schedule close follow-up (48–72 hours after starting treatment, then weekly) until mucosal coverage and symptom resolution.
• Red flags requiring urgent action
• Spreading cellulitis, fever, systemic signs, progressive numbness, or rapidly enlarging lesion — admit and treat aggressively.
• Failure to improve after sequestrectomy or atypical features — consider biopsy to exclude other pathology.
• Bottom line: treat as likely anesthesia-related osteonecrosis with prompt imaging, conservative infection control, and timely surgical removal of sequestrum by an oral surgeon to relieve pain and allow mucosal healing. Journal of Endodontics thecomfortdentistry.com
