# Supplementary material for: Comparative Evaluation of Large Language Model Interfaces in Third Molar Surgery Complication Scenarios: Response Quality, Clinical Content, Potential Clinical Risk, Readability, and Externally Observable Response Latency—A Cross-Sectional Comparative Benchmark Study
Source: J Clin Med. 2026 Sep 23;15(19):7397. doi: 10.3390/jcm15197397 (PMC13666355; doi:10.3390/jcm15197397)
Supplement: Supplementary file 1 [file jcm-15-07397-s001.zip › Supplementary Table S11 Representative Response Examples.pdf]

Supplementary Table S11. Representative Higher-Performing and Criterion-Deficient Responses Illustrating Clinical Content Differences Across Interfaces

Interface	Example type	Benchmark item	Clinical scenario	Abridged verbatim response excerpt	Criterion-based interpretation	Mean GQS	Management Completeness	Escalation/Referral	Clinical Content Score	Potential Clinical Risk	Safety-related finding
ChatGPT	Higher-performing	Q20	Suspected aspiration/ingestion of a displaced tooth after coughing and a foreign-body sensation.	"Assess airway and breathing: SpO2, respiratory rate, voice/stridor, wheeze, cyanosis. ... If the patient is actively choking ... treat as airway obstruction. ... Bronchoscopy ... is the standard for airway foreign body retrieval."	Correctly recognized aspiration as the urgent concern; explicitly assessed airway/breathing, addressed ineffective cough or severe obstruction, localized the object with imaging, and recommended urgent specialist retrieval. All predefined management and escalation criteria were satisfied.	5.0	3.0/3	1/1	5.0/5	0.0/3	None identified.
ChatGPT	Criterion-deficient	Q25	Persistent 6-mm oroantral communication two weeks after maxillary third-molar extraction.	"Management consists of surgical closure ... after confirming that the maxillary sinus is free of active infection. ... a buccal advancement flap ... may also be used. ... the patient should be instructed to follow sinus precautions."	Diagnosis and all three management elements were addressed, including surgical closure, flap options, sinus management, and sinus precautions. The predefined escalation/referral criterion was not explicitly stated: referral to OMFS when flap closure is beyond the operator's capability.	4.0	3.0/3	0/1	4.0/5	0.0/3	None identified.
Gemini	Higher-performing	Q20	Suspected aspiration/ingestion of a displaced tooth after coughing and a foreign-body sensation.	"Assess airway patency and oxygen saturation. If the patient has total airway obstruction ... initiate Basic Life Support (Heimlich maneuver) immediately. ... Bronchoscopy ... to retrieve the tooth."	Provided immediate airway assessment, explicit management of complete obstruction, mandatory radiographic localization, and urgent bronchoscopy referral. The response fulfilled diagnosis, management, escalation, and safety requirements for the item.	5.0	3.0/3	1/1	5.0/5	0.0/3	None identified.
Gemini	Criterion-deficient	Q11	Secondary hemorrhage approximately one week after mandibular third-molar extraction.	"Management focuses on controlling the acute bleeding and treating the underlying infection. ... pack the socket with a resorbable hemostatic agent ... [and] systemic antibiotics."	Correctly identified secondary hemorrhage and addressed the three prespecified management elements: clot/debridement, local hemostasis, and antimicrobial treatment. However, the predefined escalation criterion - systemic coagulopathy workup if local measures fail - was not explicitly included.	5.0	3.0/3	0/1	4.0/5	0.0/3	None identified.
DeepSeek	Higher-performing	Q20	Suspected aspiration/ingestion of a displaced tooth after coughing and a foreign-body sensation.	"Keep the patient upright if coughing spontaneously. ... If coughing is ineffective or if signs of airway obstruction develop ... follow airway obstruction protocols. ... Chest X-ray ... Bronchoscopy for removal is mandatory."	Recognized aspiration as the priority diagnosis, addressed ineffective cough and airway obstruction, recommended radiographic localization, and specified urgent bronchoscopy/specialist involvement. All predefined content and safety criteria were satisfied.	4.5	3.0/3	1/1	5.0/5	0.0/3	None identified.
DeepSeek	Criterion-deficient	Q25	Persistent 6-mm oroantral communication two weeks after maxillary third-molar extraction.	"Because the defect has persisted for two weeks and measures 6 mm, spontaneous closure is unlikely. ... definitive treatment is usually tension-free surgical closure ... [with] sinus precautions."	Accurately diagnosed persistent OAC/OAF and covered the required management components, including sinus assessment, flap closure, and sinus precautions. The operator-dependent OMFS referral criterion was not explicitly stated.	5.0	3.0/3	0/1	4.0/5	0.0/3	None identified.
Copilot	Higher-performing	Q26	Ludwig's angina with dyspnea and board-like neck swelling in a patient with uncontrolled diabetes.	"Ludwig's angina is the most likely diagnosis ... immediate airway protection ... broad-spectrum IV antibiotics, urgent surgical drainage when indicated, and ICU admission with glycemic control are required."	Correctly recognized a life-threatening deep-space infection and appropriately prioritized airway protection, IV antibiotics, urgent source control, ICU-level care, and glycemic management. All predefined management and escalation criteria were satisfied.	5.0	3.0/3	1/1	5.0/5	0.0/3	None identified.
Copilot	Criterion-deficient / safety-relevant	Q20	Suspected aspiration/ingestion of a displaced tooth after coughing and a foreign-body sensation.	"Keep the patient upright and encourage effective coughing ... Call for urgent help ... Obtain a chest and neck radiograph ... immediately to localize a radiopaque tooth."	Recognized the aspiration scenario and recommended effective coughing, urgent assistance, and localization. However, it did not explicitly prioritize immediate airway-patency/breathing assessment or specify management of ineffective cough or complete airway obstruction before localization, resulting in one missing management element and the predefined safety-critical omission.	4.0	2.0/3	1/1	4.0/5	2.0/3 (Moderate)	Safety-critical omission: immediate airway assessment / obstruction management not explicitly prioritized.

Note: Examples were selected post hoc after completion of scoring to illustrate the clinical meaning of the criterion-referenced assessment and were not used for statistical inference. Response excerpts are abridged for length using ellipses; substantive wording was not intentionally altered. Complete unabridged responses are provided in Supplementary File S1. Mean GQS, Management Completeness, Clinical Content Score, and Potential Clinical Risk are evaluator-averaged values from Supplementary File S2. Escalation/Referral is shown as 1/1 when the predefined criterion was met and 0/1 when it was not. Potential Clinical Risk range: 0-3. Safety-related findings refer to the predefined item-specific harmful-recommendation and safety-critical-omission criteria. The examples are illustrative rather than statistically representative of the full response distribution.

Selected response IDs: Q20_CHATGPT, Q25_CHATGPT, Q20_GEMINI, Q11_GEMINI, Q20_DEEPSEEK, Q25_DEEPSEEK, Q26_COPILOT, and Q20_COPILOT.
